# Megastudy shows that reminders boost vaccination but adding free rides does not

**DOI:** 10.1038/s41586-024-07591-x

**Published:** 2024-06-26

**Authors:** Katherine L. Milkman, Sean F. Ellis, Dena M. Gromet, Youngwoo Jung, Alex S. Luscher, Rayyan S. Mobarak, Madeline K. Paxson, Ramon A. Silvera Zumaran, Robert Kuan, Ron Berman, Neil A. Lewis, John A. List, Mitesh S. Patel, Christophe Van den Bulte, Kevin G. Volpp, Maryann V. Beauvais, Jonathon K. Bellows, Cheryl A. Marandola, Angela L. Duckworth

**Affiliations:** 1 Department of Operations, Information and Decisions, The Wharton School, University of Pennsylvania, Philadelphia, PA USA; 2 Behavior Change for Good Initiative, The Wharton School and the School of Arts and Sciences, University of Pennsylvania, Philadelphia, PA USA; 3 Department of Agricultural and Resource Economics, University of Maryland, College Park, MD USA; 4 Department of Marketing, The Wharton School, University of Pennsylvania, Philadelphia, PA USA; 5 Department of Communication, Cornell University, Ithaca, NY USA; 6 Department of Economics, University of Chicago, Chicago, IL USA; 7 Clinical Transformation and Behavioral Insights, Ascension Health, St Louis, MO USA; 8 Penn Center for Health Incentives and Behavioral Economics, Departments of Medical Ethics and Health Policy and Medicine, Perelman School of Medicine, University of Pennsylvania, Philadelphia, PA USA; 9 CVS Health, Woonsocket, RI USA; 10 Department of Psychology, University of Pennsylvania, Philadelphia, PA USA

**Keywords:** Human behaviour, Economics, Health policy, Decision making

## Abstract

Encouraging routine COVID-19 vaccinations is likely to be a crucial policy challenge for decades to come. To avert hundreds of thousands of unnecessary hospitalizations and deaths, adoption will need to be higher than it was in the autumn of 2022 or 2023, when less than one-fifth of Americans received booster vaccines^1,2^. One approach to encouraging vaccination is to eliminate the friction of transportation hurdles. Previous research has shown that friction can hinder follow-through^3^ and that individuals who live farther from COVID-19 vaccination sites are less likely to get vaccinated^4^. However, the value of providing free round-trip transportation to vaccination sites is unknown. Here we show that offering people free round-trip Lyft rides to pharmacies has no benefit over and above sending them behaviourally informed text messages reminding them to get vaccinated. We determined this by running a megastudy with millions of CVS Pharmacy patients in the United States testing the effects of (1) free round-trip Lyft rides to CVS Pharmacies for vaccination appointments and (2) seven different sets of behaviourally informed vaccine reminder messages. Our results suggest that offering previously vaccinated individuals free rides to vaccination sites is not a good investment in the United States, contrary to the high expectations of both expert and lay forecasters. Instead, people in the United States should be sent behaviourally informed COVID-19 vaccination reminders, which increased the 30-day COVID-19 booster uptake by 21% (1.05 percentage points) and spilled over to increase 30-day influenza vaccinations by 8% (0.34 percentage points) in our megastudy. More rigorous testing of interventions to promote vaccination is needed to ensure that evidence-based solutions are deployed widely and that ineffective but intuitively appealing tools are discontinued.

## Abstract (web summary)

Contrary to expectations, offers of free transportation to vaccine sites did not increase COVID-19 vaccine uptake more than text message reminders, a finding that has implications for future policy decision-making to promote vaccination.

## Main

In the first 10 months after COVID-19 vaccines became available, they prevented an estimated 235,000 deaths and averted 1.6 million hospitalizations in the United States alone^5^. However, as of April 2023, at least 19% of Americans had still not received their first, free COVID-19 vaccine dose^6^, and 65% had not received all recommended, free COVID-19 booster immunizations to avert the waning efficacy of vaccines after 6–8 months^1,7,8^. This lack of uptake helps explain why nearly 500 Americans were still dying every day from COVID-19 in early 2023 (ref. ^9^). The US Food and Drug Administration has announced that reformulated COVID-19 vaccines may be recommended annually for all Americans^10^. To avert hundreds of thousands of unnecessary hospitalizations and deaths in the decades to come, booster vaccine adoption will need to be higher than it was in the autumn of 2022 or 2023, when less than 20% of Americans received bivalent boosters^1,2^.

This raises the question of how COVID-19 booster vaccination can be increased. Although vaccine mandates are effective^11–13^, they are not always popular or feasible^12–14^. Moreover, in the United States, cash incentives for vaccination have proven surprisingly ineffective^15–18^. By contrast, nudges sent by text message from a healthcare provider reminding Americans to get vaccinated have yielded measurable benefits, and the reminders that work are remarkably cost-effective^19–21^. A promising untested approach to encouraging vaccination is to eliminate transportation hurdles. This could add value, given that small amounts of friction can hinder follow-through^3^. Indeed, people who live farther from COVID-19 vaccination sites have proven less likely to get vaccinated^4^. Alternatively, it could be as unproductive as offering other vaccination rewards or inducements.

Here we evaluate whether providing free transportation to vaccination sites can increase vaccination. A cardinal finding from the choice architecture literature is that small transaction costs have an outsized impact on behaviour^3^. For example, changing the default choice on a form so that it is frictionless to enrol in a savings plan or to become an organ donor substantially increases the number of savers and organ donors, respectively^22,23^. Meanwhile, reducing friction in the college financial aid application process by helping senior high school students to complete paperwork markedly increases college enrolment 2 years later^24^. In the context of COVID-19, it has been shown that individuals who happen to live farther from vaccination sites are less likely to get vaccinated^4^, whereas employees who walked by work-site flu vaccination clinics for other reasons were more likely to get their flu vaccination^25^. Vaccine accessibility is a challenge for many populations, and it has received widespread media attention^26–28^. Thus, making vaccines more accessible through free round-trip rides to appointments would seem to be a good investment.

However, if lack of transportation were a key barrier to vaccination, then cash rewards could be used to fund rides to vaccination sites, and cash rewards have had no measurable impact on the Americans’ decisions to receive COVID-19 vaccines^15–18^. Furthermore, cash rewards offer a more flexible solution to accessibility hurdles than a free ride, which cannot facilitate childcare, eldercare or time off from work. The ineffectiveness of cash rewards as a means of encouraging COVID-19 vaccinations in the United States therefore suggests that free rides might not boost vaccination rates, particularly among those who have previously successfully obtained their primary COVID-19 immunization series. Notably, large investments were made in free rides to and from vaccination sites in mid-to-late 2021 (refs. ^29–32^) on the assumption that complementary transportation could help people to overcome a key logistical hurdle to vaccination. In fact, the free-ride intervention we test here is modelled on a similar program that was deployed by the White House from May to July of 2021 in partnership with Uber and Lyft (the effectiveness of this programme was not evaluated)^33^.

Because we know reminders direct attention to goals that may otherwise be forgotten^34,35^ and strongly increase immunization rates^19–21,36^, as well as many other policy-relevant outcomes^36–40^, we embedded our free-ride offering in a reminder message. Past research suggests that incorporating behavioural insights into reminders can increase their impact^19,41–43^. For example, conveying to people that a vaccine belongs to them (that is, they can ‘claim’ it or it is ‘reserved’ or ‘waiting’ for them) can increase immunization rates under certain conditions^19–21,44^. Offering people default appointments through reminders boosts vaccination uptake^41^. Moreover, prompting people to plan the date and time when they will get vaccinated increases the effectiveness of vaccine reminders^42^. Therefore, alongside our test of the value of free rides (shared through a behaviourally informed reminder message), we assess the impact of various other behaviourally informed reminders on encouraging booster vaccination uptake.

Here we present a megastudy—a field experiment testing many interventions at once^45,46^—in which we compare seven different behaviourally informed text reminders encouraging the receipt of a bivalent COVID-19 booster vaccine against text reminders offering individuals a free round-trip Lyft ride to their vaccination appointment. Our megastudy was conducted with more than 3.66 million patients of CVS Pharmacy in the United States who had previously received their primary COVID-19 vaccination series. We compare the actual impact of the interventions tested in our megastudy on vaccination uptake with predictions by both laypeople and PhD behavioural scientists. We find that offering patients free round-trip Lyft rides to and from their pharmacy has no measurable benefit over and above sending them two text reminders that follow best practices from previous research and encourage the receipt of a bivalent COVID-19 booster that is ‘recommended’ and ‘waiting’ for them^19–21,44,47^. We find slight variation in the performance of different reminder texts. The three text reminders that emerged as top performers (1) encouraged patients to make a vaccination plan and suggested a specific day of the week, time of day and CVS Pharmacy location for an appointment that matched when and where a patient had received their last vaccination at a CVS Pharmacy; (2) communicated that there were high current infection rates in a patient’s county; or (3) appeared to be sent directly by the pharmacy team at the patient’s most frequently visited CVS Pharmacy location. Notably, all of the bivalent COVID-19 booster reminders tested had a spillover benefit, whereby flu vaccination rates were increased.

Both laypeople and behavioural science experts proved to be poorly calibrated forecasters of what works to promote bivalent COVID-19 booster adoption. Both groups incorrectly predicted that offering individuals a free, round-trip ride to vaccination appointments would produce larger benefits than sending other types of reminder. These findings highlight the need for more experiments like the one we conducted to inform optimal policy decisions.

## Megastudy to promote COVID-19 vaccination

In our megastudy, we focused on encouraging adoption by adults of the bivalent COVID-19 booster vaccine in the autumn of 2022. This vaccine was recommended by the Centers for Disease Control (CDC) for all adults who had completed any primary COVID-19 vaccination series or received a monovalent booster^48^. As of mid-November 2022 (shortly after the launch of our study), only 11% of Americans had received this recommended bivalent booster vaccine^1^. We partnered with CVS Pharmacy—a large US pharmacy chain with nearly 10,000 locations across the United States—to test eight interventions among their patients.

Our megastudy included 3,662,548 CVS Pharmacy patients deemed eligible for participation in our study by CVS Pharmacy on 18 October 2022 (see Methods for more details). For all analyses of the effectiveness of our interventions, we relied on records from CVS Pharmacy to assess which patients received a bivalent booster at any CVS Pharmacy within 30 days of the start of their intervention (or control) period.

We worked with CVS Pharmacy and a team of nine behavioural science experts to develop eight different intervention messages that were sent to patients by text message in early November 2022 to encourage adoption of the bivalent COVID-19 booster (see Table 1 for a summary of the interventions). All eight interventions consisted of an initial set of reminder texts with a follow-up set of reminder texts sent 7 days later, and all the tested text message reminders conveyed to patients that a vaccine was ‘recommended’ and ‘waiting for you’, language that was built on past research^19–21^. The intervention of focal interest was designed to test the value of free round-trip rides to vaccination sites and it included the aforementioned standard reminder language but also provided people with one free round-trip ride by Lyft (a popular ride-sharing app) to and from a CVS Pharmacy in the month ahead.

**Table 1:** Text messages sent to patients by intervention condition

Intervention (sample size)	Launch day text messages	Follow-up texts sent 7 days after launch
1. Baseline message (*n* = 492,572)	CVS Pharmacy: Hi [Patient First Name]! Updated COVID boosters are recommended to help prevent infection & severe illness. Your booster is waiting for you at CVS. Schedule: cvs.co/8981004	CVS Pharmacy: Remember, a COVID booster is waiting for you at CVS. Schedule: cvs.co/9810048
2. Free ride (*n* = 50,000)	CVS Pharmacy: Hi [Patient First Name]! Updated COVID boosters are recommended to help prevent infection & severe illness. Your booster is waiting for you at CVS.	CVS Pharmacy: Remember, a COVID booster is waiting for you at CVS. Schedule: cvs.co/7473148
	A free ride to and from CVS has been reserved for your booster appointment until 12/8/22 with support from the Mercury Project. Schedule: cvs.co/8747314	As a reminder, a free ride to and from CVS has been reserved for your booster appointment until 12/8/22 with support from the Mercury Project.
	You can claim your free rides to or from any CVS near you by entering your personal code VAXBR4QKHVQBRKLM in the Lyft app https://lyft.com/lp/VAXBR4QKHVQBRKLM	You can claim your free rides to or from any CVS near you by entering your personal code VAXBR4QKHVQBRKLM in the Lyft app https://lyft.com/lp/VAXBR4QKHVQBRKLM
3. Default plan (*n* = 492,573)	CVS Pharmacy: Hi [Patient First Name]! Updated COVID boosters are recommended to help prevent infection & severe illness. Your booster is waiting for you at CVS.	CVS Pharmacy: Remember, a COVID booster is waiting for you. Many find it helps to plan ahead. If you haven’t yet, consider planning when you’ll get yours.
	Many find it helps to make a plan. Would Tuesday at 2:00 PM at 1 Main Street work?	How would Tuesday at 2:00 PM at 1 Main Street work?
	To try to book that time, or another that works better for you, schedule here: cvs.co/5822335	To try to book that time, or another that works better for you, schedule here: cvs.co/8223355
4. Infection rates (*n* = 492,573)	CVS Pharmacy: Hi [Patient First Name]! CDC data show significant current COVID transmission in Washington County. [Infection rates are in the top X% in the US].	CVS Pharmacy: Washington County currently has significant COVID transmission. [Infection rates are in the top X% in the US].
	Updated COVID boosters are recommended to help prevent infection & severe illness. Your booster is waiting for you at CVS. Schedule: cvs.co/5462339	Remember, to keep you safe a COVID booster is waiting for you at CVS. Schedule: cvs.co/4623395
5. Pharmacy team message (*n* = 492,573)	CVS Pharmacy: Hi [Patient First Name]! This is a message from your Pharmacy Team at 1 Main Street.	Pharmacy: Hi again [Patient First Name]! This is a message from your Pharmacy Team at 1 Main Street.
	Updated COVID boosters are recommended to help prevent infection & severe illness. We have a booster waiting for you at CVS. Schedule: cvs.co/8917615	As a reminder, we have a COVID booster waiting for you at CVS. Schedule: cvs.co/9176158
6. CDC recommended (*n* = 492,573)	CVS Pharmacy: Hi [Patient First Name]! The CDC recommends updated COVID boosters to help prevent infection & severe illness. Your booster is waiting for you at CVS. Schedule: cvs.co/6011623	CVS Pharmacy: Remember, a COVID booster is recommended by the CDC & waiting for you at CVS. Schedule: cvs.co/0116236
7. Holiday protection (*n* = 328,285)	CVS Pharmacy: Hi [Patient First Name]! The holiday season is just a few weeks away & updated COVID boosters are recommended to help prevent infection & severe illness.	CVS Pharmacy: Remember, a COVID booster is waiting for you at CVS. Get your booster now so you can more safely gather with loved ones over the holidays.
	Get your booster now so you can more safely gather with loved ones over the holidays. Your booster is waiting for you at CVS. Schedule: cvs.co/1065105	Schedule: cvs.co/0651051
8. Misinformation resources (*n* = 328,826)	CVS Pharmacy: Hi [Patient First Name]! Updated COVID boosters are recommended to help prevent infection & severe illness. Your booster is waiting for you at CVS.	CVS Pharmacy: Remember, a COVID booster is waiting for you at CVS. And here are some important facts about boosters: www.CDC.gov.
	Here are some important facts about why boosters are recommended: www.CDC.gov. You can also call (555) 867-5309 to speak with a pharmacist if you have questions. Schedule: cvs.co/6846120	You can also call (555) 867-5309 anytime to speak with a pharmacist if you have any questions. Schedule: cvs.co/6846120

Our seven other interventions did not offer free round-trip Lyft rides to a CVS Pharmacy. These interventions instead layered a range of different strategies for encouraging immunization on top of the standard reminder, from conveying current (high) rates of infection in a patient’s county to providing resources to combat misinformation (Table 1).

**Fig. 1:**
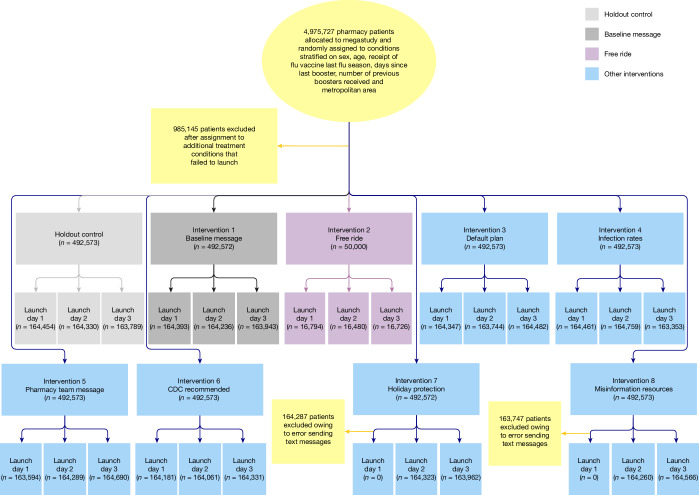
Megastudy CONSORT flow diagram. Note that after randomization, two planned interventions failed to deploy, which led 985,145 patients to be excluded from the originally planned sample. The two interventions that failed to deploy are not discussed as part of this megastudy. In addition, interventions 7 and 8 failed to deploy on launch day 1; therefore, only patients randomized to interventions 7 and 8 on launch days 2 and 3 were included in the megastudy.

As shown in the CONSORT diagram in Fig. 1, and following our pre-registration (see Methods for more details), eligible patients were randomly assigned to one of eight different intervention conditions or a holdout control condition in which they did not receive any reminder messages to get vaccinated during the duration (30 days) of the study.

Extended Data Table 1 and Supplementary Table 1 demonstrate that our nine study conditions were balanced on age, sex and vaccinations for flu in the previous flu season (*P* values from all three undirected *F*-tests > 0.064; all three Bayes factor values ⪞ 1.119 × 10^23^ in support of no difference). Patients were randomly assigned to receive their first reminder message on one out of three dates in November (day 1, day 2 or day 3; see Methods for details). However, conditions 7 and 8 were not administered on day 1 (as described in Methods, on day 1, conditions 7 and 8 failed to launch and these conditions therefore have no day 1 patients). This left 25 condition–day combinations (see Methods for details). Age, sex and vaccinations for flu the previous flu season were all balanced across these 25 condition–day combinations (three undirected *F*-tests all have *P* > 0.290; Bayes factor values ⪞ 6.869 × 10^72^ in support of no difference; Extended Data Table 1 and Supplementary Table 2). The nine study conditions were also balanced on a measure of total previous COVID-19 booster vaccinations provided by CVS Pharmacy in October 2022 (undirected *F*-test, *P* = 0.082; Bayes factor ≈ 1.646 × 10^23^ in support of no difference). However, a measure of the total COVID-19 vaccinations obtained by individuals prior to the launch of our first wave of text message reminders on day 1 that was extracted in December 2022 showed some imbalance (undirected *F*-test, *P* < 0.001; Extended Data Table 1 and Supplementary Table 1). This unexpected imbalance helped us to determine and confirm with CVS Pharmacy that the historical vaccination records of patients were sometimes updated after the fact (that is, when our interventions brought patients into the pharmacy, see Supplementary Information, section 3). Thus, several control variables we had pre-registered, including in our primary analyses—variables extracted in December 2022—were likely to have been influenced by the condition assignment of the patient (that is, individuals in megastudy conditions that produced more CVS Pharmacy visits for bivalent booster vaccinations had more previous vaccines ‘updated’, so they appeared to receive more vaccinations pre-treatment than other individuals). To address this, we adjusted our primary analysis strategy to an intent-to-treat strategy including all patients deemed eligible for inclusion in our study as of 18 October 2022 (except those assigned to intervention conditions in which reminders failed to send; Fig. 1), and we relied on a pre-registered robustness check that excluded control variables as our primary regression specification (Methods and Table 2, model 1). Notably, our results were robust to our original, problematic analysis strategy (in which a key control variable was likely to have been influenced by the condition assignment of the individual; Table 2, model 2).

**Table 2:** Regression-estimated impact of each of the eight intervention conditions in our megastudy

	COVID bivalent booster uptake								Flu vaccination uptake			
	Within 30 days				Within 90 days				Within 30 days			
	Model 1		Model 2		Model 3		Model 4		Model 5		Model 6	
	*β*	*P*	*β*	*P*	*β*	*P*	*β*	*P*	*β*	*P*	*β*	*P*
Intervention 1: baseline message	1.005 (0.046)	<0.001	0.914 (0.044)	<0.001	0.868 (0.061)	<0.001	0.735 (0.057)	<0.001	0.309 (0.043)	<0.001	0.316 (0.042)	<0.001
Intervention 2: free ride	0.968 (0.111)	<0.001	0.913 (0.106)	<0.001	0.810 (0.143)	<0.001	0.732 (0.134)	<0.001	0.422 (0.101)	<0.001	0.437 (0.100)	<0.001
Intervention 3: default plan	1.204 (0.047)	<0.001	1.124 (0.045)	<0.001	1.026 (0.061)	<0.001	0.915 (0.057)	<0.001	0.392 (0.043)	<0.001	0.401 (0.042)	<0.001
Intervention 4: infection rates	1.105 (0.046)	<0.001	1.053 (0.044)	<0.001	0.830 (0.061)	<0.001	0.758 (0.057)	<0.001	0.367 (0.043)	<0.001	0.377 (0.042)	<0.001
Intervention 5: pharmacy team message	1.098 (0.046)	<0.001	1.030 (0.044)	<0.001	0.954 (0.061)	<0.001	0.858 (0.057)	<0.001	0.374 (0.043)	<0.001	0.383 (0.042)	<0.001
Intervention 6: CDC recommended	1.079 (0.046)	<0.001	0.992 (0.044)	<0.001	1.045 (0.061)	<0.001	0.921 (0.057)	<0.001	0.318 (0.043)	<0.001	0.316 (0.042)	<0.001
Intervention 7: holiday protection	0.978 (0.052)	<0.001	0.910 (0.050)	<0.001	0.817 (0.068)	<0.001	0.720 (0.064)	<0.001	0.310 (0.048)	<0.001	0.326 (0.047)	<0.001
Intervention 8: misinformation resources	0.949 (0.052)	<0.001	0.877 (0.050)	<0.001	0.878 0.069	<0.001	0.771 (0.064)	<0.001	0.263 (0.048)	<0.001	0.265 (0.047)	<0.001
*F*-statistic for *F*-test of whether all eight treatments had the same effect	4.848	<0.001	5.609	<0.001	3.543	<0.001	3.432	0.001	1.687	0.107	1.961	0.056
Are controls included?	No		Yes		No		Yes		No		Yes	
Observations (*n*)	3,662,548		3,662,548		3,662,548		3,662,548		3,662,548		3,662,548	
*R*^2^	3.64 × 10^–4^		8.99 × 10^–2^		3.66 × 10^–4^		1.27 × 10^–1^		2.22 × 10^–4^		2.13 × 10^–2^	
Vaccination rate of control group (%)	5.09		5.09		9.65		9.65		4.52		4.52	

Regression-estimated impact of each of the eight intervention conditions in our megastudy on bivalent COVID-19 booster uptake at a CVS Pharmacy within 30 days of a patient’s study launch day (models 1–2), bivalent COVID-19 booster uptake at a CVS Pharmacy within 90 days of a patient’s study launch day (models 3–4) and flu vaccination uptake at a CVS Pharmacy within 30 days of a patient’s study launch day (models 5–6).

Note that this table reports the results of six ordinary least squares (OLS) regressions to predict whether a given individual received a given vaccine at a CVS Pharmacy. The primary predictor variables in these regressions are eight indicators for assignment to each of our megastudy’s eight intervention conditions (the holdout control condition is the comparison group). All regression models also include indicators for whether the individual received their first text message on launch day 1 or launch day 2 (an indicator for receiving a message on launch day 3 is omitted). In models 2, 4 and 6, additional controls are included for the patient’s age as of October 2022; an indicator for whether the patient’s age was greater than or equal to 50 years in October 2022; an indicator for whether a patient is male; and indicators for the patient’s insurance status (Medicare, Medicaid or unknown; commercial insurance is omitted) as of December 2022. Models 2 and 4 also control for the patient’s number of previous COVID-19 boosters before the start of the study according to the records of CVS Pharmacy as of October 2022 and their number of previous COVID-19 vaccinations before the start of the study according to records of CVS Pharmacy as of December 2022 (which were potentially affected by our interventions, as reported above and in Supplementary Information, section 3). Model 6 includes an indicator for whether a patient received a flu vaccine at any CVS Pharmacy during the 2021–2022 flu season. The control variables in all models are mean-centred using the mean of the holdout control so the constant term is identical to the vaccination rate estimated by the corresponding model without additional control variables. All regression coefficients and standard errors were multiplied by 100 to improve interpretability (and therefore reflect a percentage point change induced in vaccination uptake). Standard errors reported in parentheses are robustly estimated using heteroskedasticity-consistent (HC1) standard errors, and *P* values are adjusted for multiple comparisons using the BH procedure. Statistical tests of whether an individual regression coefficient is zero are all two-sided. Statistical tests involving multiple regression coefficients are all undirected.

**Fig. 2:**
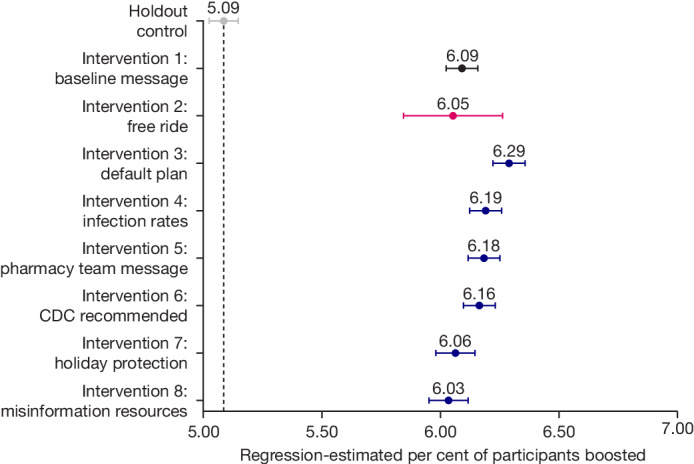
Regression-estimated percentage of patients who received a bivalent COVID-19 booster at a CVS Pharmacy within 30 days of this megastudy’s launch, by condition. These estimates are derived from a variant of our main regression model (Table 2, model 1) in which we include an additional binary indicator for assignment to the holdout control condition and exclude the intercept. The number of patients in each megastudy condition depicted here are as follows: holdout control (492,573), baseline message (492,572), free ride (50,000), default plan (492,573), infection rates (492,573), pharmacy team message (492,573), CDC recommended (492,573), holiday protection (328,285), misinformation resources (328,826). Whiskers depict 95% CI.

In our holdout control condition, 5.09% of CVS Pharmacy patients received a bivalent COVID-19 booster within 30 days of their megastudy launch date. The results of our primary regression analysis (described in Methods) estimating the impact of each intervention condition are presented in Table 2, model 1. Figure 2 presents estimated vaccination rates across conditions. As Fig. 2 shows, when a vaccination reminder message indicated that a vaccine was ‘recommended’ and ‘waiting for you’ and offered patients a free round-trip Lyft ride to a local CVS Pharmacy, it produced no more vaccination uptake than our baseline text reminder without a free-ride offer, which simply indicated that a vaccine was recommended and waiting for you (two-sided Wald test Benjamini–Hochberg (BH)-adjusted *P* = 0.739; Bayes factor ≈ 1806.514 in support of no difference; Supplementary Table 3).

On average, the eight interventions we tested increased bivalent booster vaccination rates by 20.63% (1.05 percentage points) within 30 days of deployment, and each intervention significantly increased bivalent COVID-19 booster vaccination rates of patients during this time period (all BH-adjusted *P* values < 0.001). We reject the null hypothesis that all eight interventions had the same effect (undirected *F*-test, *P* < 0.001; Table 2, model 1). Also, the difference in regression-estimated vaccination rates between the best-performing and the worst-performing intervention was only 0.26 percentage points (a 4.13% difference; two-sided Wald test, *P* < 0.001; Supplementary Table 3). Our results are robust to including pre-registered controls in our regression (Table 2, model 2), even though some of these controls were likely to be differentially influenced by a patient’s condition assignment. The results were also generally robust to excluding data from launch day 1, although doing so slightly decreased the estimated benefits of most interventions (average estimated decrease in intervention effectiveness when data from day 1 is excluded = 3.93%; Table 2, model 1, and Extended Data Table 2, model 1).

We analysed which intervention was the true top performer and how the free-ride intervention ranked against others tested. Confidence sets for ranks^49^ (Extended Data Table 3, model 1) showed that three reminder interventions cannot be ruled out (95% confidence) from being the true top performer, and the free-ride intervention was not one of them. The first encouraged a patient to ‘make a plan’ for getting a vaccine and specifically suggested scheduling an appointment at the same time and location when and where the patient received their last vaccination at a CVS Pharmacy (boosting vaccination rates by 23.65%, or 1.20 percentage points, BH-adjusted *P* < 0.001). The second conveyed that there was significant COVID-19 transmission in the patient’s county, and if infection rates in the patient’s county were in the top 50% of US counties, these reminders also conveyed the exact infection decile (for example, ‘infection rates are in the top 20% in the United States across all counties’; these messages boosted vaccination rates by 21.71%, or 1.11 percentage points, BH-adjusted *P* < 0.001). The final top-performing message was ostensibly sent by the pharmacy team at the patient’s most frequently visited CVS Pharmacy location (for example, ‘This message is from your Pharmacy Team at 1 Main Street’; these messages boosted vaccination rates by 21.57%, or 1.10 percentage points, BH-adjusted *P* < 0.001; see Supplementary Information, section 7, for more details on the test of ranks). Two-sided Wald tests confirmed that our ‘make a plan’ message significantly outperformed our baseline message (BH-adjusted *P* < 0.001; Supplementary Table 3), whereas our ‘infection rates’ reminder only significantly outperformed our baseline message without adjusting for multiple comparisons (unadjusted *P* = 0.039, BH-adjusted *P* = 0.126; Supplementary Table 3). The ‘pharmacy team message’ reminder only marginally outperformed our baseline message without adjusting for multiple comparisons (unadjusted *P* = 0.054, BH-adjusted *P* = 0.126; Supplementary Table 3).

After applying a James–Stein shrinkage procedure, our best-performing reminder intervention boosted vaccination uptake by an estimated 1.18 percentage points, whereas our worst-performing reminder intervention boosted vaccinations by an estimated 0.96 percentage points^50^. This result means that even our worst-performing message, which was designed following best practices, achieved roughly 80% of the benefits of our top-performing message (0.96/1.18 = 0.81).

We also examined two pre-registered, secondary outcomes: (1) receipt of bivalent boosters within 90 days of a patient’s launch date (instead of 30 days) and (2) flu vaccinations within 30 days of launch. Bivalent booster uptake within 90 days after launch exhibited similar, if diluted, treatment responsiveness to what is documented in the main analysis (Table 2, models 3–4). This result suggests that the interventions did not merely accelerate vaccination uptake but also increased the total number of individuals vaccinated. On average, the eight interventions increased bivalent booster vaccination rates by 9.36% (0.90 percentage points) within 90 days of deployment (from 9.65% in the holdout control to 10.55%, on average, in the intervention conditions; Table 2, model 3). This is a similar point estimate to the 1.05 percentage point increase in vaccination rates produced by the interventions during our 30-day follow-up period, but it is a smaller percent change because the 30-day baseline vaccination rate during our study was 5.09%, whereas the 90-day vaccination rate was 9.65%. All eight interventions significantly increased bivalent COVID-19 booster vaccination rates during the 90-day follow-up period (all BH-adjusted *P* values <0.001). Furthermore, we again reject the null hypothesis that all eight interventions had the same effect (undirected *F*-test *P* < 0.001; Table 2, model 3).

Examining the adoption of flu vaccinations showed that on average, our eight interventions increased flu vaccination rates by 7.62% within 30 days of deployment (up 0.34 percentage points from 4.52% in the holdout control group to 4.86%, on average, in the intervention groups; Table 2, model 5). Furthermore, all eight interventions significantly increased patients’ flu vaccination rates during this time period (all BH-adjusted *P* values < 0.001). In our primary model (Table 2, model 5), all eight interventions had the same effect (undirected *F*-test, *P* = 0.107; Bayes factor ≈ 1.860 × 10^20^ in support of no difference).

The efficacy of reminder text messages and the failure of free round-trip Lyft rides to boost vaccination rates over and above sending multiple vaccination reminders suggest that attentional hurdles are likely to play a greater role than transportation accessibility hurdles in preventing previously vaccinated individuals from pursuing COVID-19 booster vaccines.

## Forecasts of the effects of interventions

The large outlays of resources to support free rides to vaccination sites (including the high-profile 2021 White House partnership with Lyft and Uber^29,51,52^) suggest that policy makers were bullish on the benefits of this approach, but the lack of benefit produced by free rides in the population we studied does not support this optimism. To determine whether this mismatch could be driven in part by inaccurate beliefs about the benefits of free rides, we conducted two follow-up forecasting surveys to measure the accuracy of people’s expectations about which interventions most effectively promote vaccination.

In our first forecasting study, lay participants (*n* = 199, Prolific sample) were presented with information about (1) our megastudy, (2) the patients included in it, and (3) the bivalent COVID-19 booster vaccination rate among patients in our holdout control condition during the 30-day megastudy period. Lay participants were then shown the exact messages sent to individuals in each of our megastudy’s intervention conditions (one at a time, in random order) and asked to forecast the bivalent COVID-19 booster vaccination rate in the intervention group in the 30 days after receiving their first message (see Methods for more details).

**Fig. 3:**
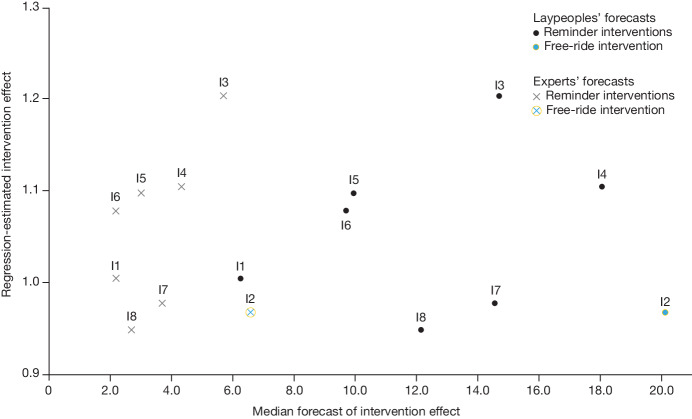
Forecasted intervention effects by laypeople and experts and actual, regression-estimated intervention effects. Scatterplots showing median forecasted intervention effects on the *x*-axis and actual, regression-estimated intervention effects on the *y*-axis with forecasts from laypeople and experts. I1, baseline message; I2, free ride; I3, default plan; I4: infection rates; I5: pharmacy team message; I6, CDC recommended; I7, holiday protection; I8, misinformation resources.

Lay forecasters predicted that the offer of free round-trip Lyft rides to the pharmacy would spur the most vaccinations of all interventions tested, proffering a median forecasted 30-day vaccination rate of 25.42% in the population assigned to this condition (or a 378.72% increase from baseline). At the time of these forecasting studies, we had not yet determined that data on vaccination rates of patients before 3 November 2022 were unreliable (because they were likely to be differentially influenced by individuals’ condition assignments). Therefore, when calculating summary statistics to share with forecasters, we excluded data from participants who appeared to have received a vaccine before the 3 November 2022 launch of our experiment. We told survey forecasters that 5.31% of patients in our control condition had been vaccinated, but our current analysis reports that 5.09% of patients in our control condition were vaccinated (because we did not exclude any patients from our analysis who were randomly assigned to conditions). To analyse the accuracy of the forecasters’ estimates in light of this issue, we calculated the absolute change in vaccination rates they forecasted (for example, if they predicted a treatment would produce a 6.31% vaccination rate, we would call that a predicted 1.00 percentage point boost from baseline). When describing the percentage changes in vaccination rates that were forecasted, we use a similar approach (for example, if they predicted a treatment would produce a 6.31% vaccination rate, we would call that a predicted 18.83% boost from baseline). All results described here are robust to analysing the percentage change in vaccination rates that were forecasted (see Supplementary Information, section 11). The lay forecasters predicted that this intervention would significantly outperform all other interventions tested (two-sided Wilcoxon signed-rank tests, all BH-adjusted *P* values < 0.010). On average, lay forecasters expected our seven reminder-only interventions to produce a median increase in vaccinations of 229.51% over baseline (a 17.50% 30-day vaccination rate). Notably, lay forecasters were substantially overoptimistic about the effects of both free rides and reminders (their estimates were 6–21 times too high for every single intervention). Lay forecasters were also poorly calibrated regarding the relative performance of the interventions: the Pearson correlation between their eight (median) forecasts and the actual regression-estimated performance of each intervention was −0.01 (Fig. 3).

To assess whether experts were better calibrated than lay forecasters, we conducted the same forecasting study with PhD behavioural scientists (*n* = 163; see Methods for more details). Similar to the lay forecasters, experts predicted that the offer of free round-trip Lyft rides to the pharmacy would outperform all other interventions tested (forecasting a median 30-day vaccination rate of 11.89% in the population assigned to this condition or a 123.92% increase from baseline). Experts predicted that this intervention would significantly outperform all other reminder-only interventions tested (two-sided Wilcoxon signed-rank tests, all BH-adjusted *P* values < 0.05). It is worth noting that this forecasted absolute change in vaccinations of 6.58 percentage points is not outside the realm of possibility when considering boosts in vaccination uptake that have been reported in recent articles, such as a 2010 study that found defaulting individuals into vaccination appointments increased vaccination rates by 11.72 percentage points^41^.

On average, expert forecasters expected our seven reminder interventions to produce a median increase in vaccinations of 63.97% over baseline (an 8.71% 30-day vaccination rate). Similar to the lay forecasters, experts were also overly optimistic about the effects of specific reminders (their estimates were 2–7 times too high for every single intervention), but they were significantly less optimistic about the performance of every intervention than lay forecasters (all BH-adjusted *P* values from two-sided Wilcoxon rank sum tests <0.001). Moreover, their forecast of a roughly 3 percentage point boost in vaccinations from reminder messages was in line with several recent studies that have shown that such messages can produce up to 4 percentage point increases in vaccinations when baseline vaccination rates are in the double digits^19,20^. Expert forecasters were also directionally better calibrated regarding the relative performance of all interventions tested than lay forecasters (consistent with considerable past research^53,54^), although they were still poorly calibrated overall: the correlation between their eight (median) forecasts and actual intervention effects was 0.24 (Fig. 3).

In summary, both experts and lay people incorrectly anticipated that providing patients with free rides to and from pharmacies would generate substantial unrealized value relative to sending reminders, which may help explain the large past investments made in free rides to and from vaccination sites. Forecasters were also an order of magnitude too bullish about the benefits of all of our interventions on vaccination rates, although experts were substantially less bullish than lay people. Notably, estimates of absolute changes in vaccination in response to our interventions by both groups were in line with changes that have been measured in the recent literature when baseline vaccination rates were higher^19,20,41^. Therefore, the overall effect size miscalibration detected may primarily reflect a failure to recognize that low vaccination base rates shift the absolute effect sizes that interventions are likely to achieve in this context.

## Heterogeneity in the effects of the interventions

COVID-19 is particularly dangerous for elderly populations, making it an urgent policy priority to ensure that older Americans receive recommended booster vaccinations^55–57^. The virus has also taken a relatively greater toll on Americans with fewer resources^58^, and vaccine take-up has lagged in some political subcultures more than others^14^. In pre-registered exploratory analyses, we investigated the relative effects of our interventions on different subpopulations.

**Fig. 4:**
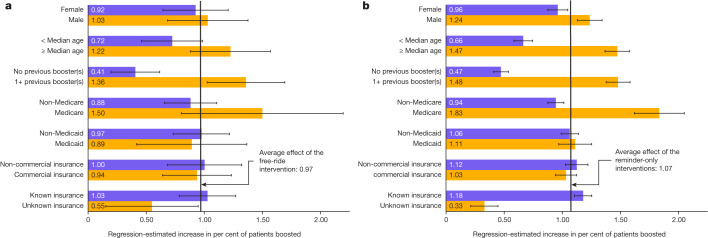
Regression-estimated effects by subpopulation of assignment to the free-ride intervention and, separately, to the reminder-only interventions. **a**,**b**, Regression-estimated effects of assignment to the free-ride intervention for different subpopulations (**a**) and for all other reminder-only interventions pooled again by subpopulation (**b**) on COVID-19 bivalent booster vaccination rates at a CVS Pharmacy within 30 days (the reference group is the holdout control group). Note that these estimates are drawn from 14 new regressions (one for each subpopulation shown in the two graphs; Supplementary Tables 4–17, model 2). Each new regression uses the same specification as our main regression model (Table 2, model 1) but includes only patients from the relevant subpopulation (for example, female patients only) and includes different primary predictors (in place of eight indicators for experimental condition, the models all include one indicator for whether a patient received our free-ride intervention (to inform **a**), and one indicator for whether a patient received any reminder-only intervention (to inform **b**). For **a**, the number of patients in subpopulations is as follows: female (29,684), male (20,316), <median age, (24,734) ≥median age, (25,266), no previous booster(s) (20,249), 1+ previous booster(s) (29,751), non-Medicare (42,659), Medicare (7,341), non-Medicaid (42,598), Medicaid (7,402), non-commercial insurance (21,031), commercial insurance (28,969), known insurance (43,712), unknown insurance (6,288). For **b**, the number of patients in each subpopulation is as follows: female (1,858,359), male (1,261,616), <median age (1,536,715), ≥median age (1,583,260), no previous booster(s) (1,261,166), 1+ previous booster(s) (1,858,809), non-Medicare (2,661,549), Medicare (458,426), non-Medicaid (2,651,800), Medicaid (468,175), non-commercial insurance (1,319,882), commercial insurance (1,800,093), known insurance (2,726,694), unknown insurance (393,281). Whiskers depict 95% CI.

**Extended Data Fig. 1:**
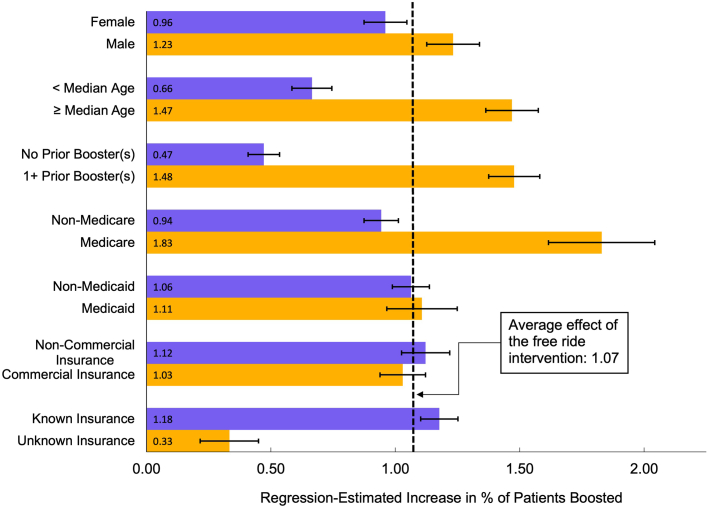
Regression-estimated effect of all intervention arms (pooled) vs. the holdout control on bivalent COVID-19 booster vaccination rates by patient subpopulation. *Note:* This figure presents the average regression-estimated effect of assignment to any intervention condition for different patient sub-populations. These estimates were obtained from fourteen separate regressions (one for each bar, see Tables S4–S17, Model 3). Each regression relied on the same specification as our main regression model (Table 2, Model 1) but included only a single pooled treatment indicator predictor and only patients from the relevant subpopulation. Whiskers depict 95% confidence intervals around each intervention effect estimate.

First, we assessed whether the effects of our interventions varied depending on specific characteristics of the individual, including their age, sex, past adoption of the booster vaccine (or vaccines) as measured in October 2022, and insurance coverage as measured in December 2022 (Supplementary Tables 4–24). Notably, the performance of our eight different interventions correlated highly across different subpopulations. Specifically, the average correlation between our eight treatment effect estimates across all 16 subpopulations examined was 0.72 (see Extended Data Table 4 for the correlations between our eight intervention effect estimates by subgroup). Because subgroup analyses looked so similar across interventions, Fig. 4 shows the effects of our free-ride intervention (*β*_free ride_) by subgroup (Fig. 4a) but pools the effects of our seven reminder-only interventions (*β*_reminder-only_) when showing their performance by subgroup (Fig. 4b). As illustrated in Fig. 4 and Extended Data Fig. 1, our free-ride and reminder-only interventions were generally more effective for the following individuals: (1) older recipients (Supplementary Table 7, model 2; *β*_free ride_ = 1.23, 95% confidence intervals (CI) = 0.88–1.57, BH-adjusted *P* < 0.001; *β*_reminder-only_ = 1.47, 95% CI = 1.37–1.58, BH-adjusted *P* < 0.001); (2) recipients with Medicare coverage (Supplementary Table 11, model 2; *β*_free ride_ = 0.88, 95% CI = 0.80–2.19, BH-adjusted *P* < 0.001; *β*_reminder-only_ = 0.94, 95% CI = 1.62–2.05, BH-adjusted *P* < 0.001); (3) men (Supplementary Table 5, model 2; *β*_free ride_ = 1.03, 95% CI = 0.68–1.37, BH-adjusted *P* < 0.001; *β*_reminder-only_ = 1.24, 95% CI = 1.34–1.13, BH-adjusted *P* < 0.001); and (4) individuals who had received at least one previous booster according to CVS Pharmacy records (Supplementary Table 9, model 2; *β*_free ride_ = 1.36, 95% CI = 1.02–1.69, BH-adjusted *P* < 0.001; *β*_reminder-only_ = 1.48, 95% CI = 1.38–1.58, BH-adjusted *P* < 0.001).

Next, we explored whether the impact of our interventions varied on the basis of patients’ neighbourhood characteristics (based on the zip code of the nearest CVS Pharmacy of the patient). Specifically, we explored whether there are differences in the impact of our interventions by the wealth, education, density, racial composition and 2020 Republican presidential vote share of patients’ neighbourhoods (Supplementary Tables 25–57). The interventions were generally more effective for the following populations: (1) individuals from lower-income neighbourhoods (Supplementary Table 35, model 3; *β*_below median income × any intervention_ = 1.17, 95% CI = 1.08–1.25, BH-adjusted *P* < 0.001); (2) patients in neighbourhoods where fewer residents had earned Bachelor’s degrees (Supplementary Table 37, model 3; *β*_below median Bachelor’s × any intervention_ = 1.10, 95% CI = 1.01–1.18, BH-adjusted *P* < 0.001); and (3) individuals from neighbourhoods where a lower proportion of residents are white (Supplementary Table 25, model 3; *β*_below median white × any intervention_ = 1.10, 95% CI  = 1.00–1.19, BH-adjusted *P* < 0.001). We did not observe substantial or systematic heterogeneity in treatment effects on the basis of population density, CVS Pharmacy density, 2020 Republican presidential vote share, previous COVID-19 vaccination rates or the proportion of Black, Hispanic or Asian residents in the neighbourhoods of the patients in our megastudy (Supplementary Tables 48–50 and 53–57).

Older and lower-income Americans are at particularly high risk for complications from COVID-19, and both older individuals and those from lower-income neighbourhoods responded particularly well to vaccination reminder interventions. Therefore, the benefits of implementing reminder interventions such as those studied here may be particularly substantial in these populations.

## Discussion

Our megastudy demonstrated that offering previously vaccinated individuals free Lyft rides to and from a pharmacy for booster vaccines does not produce measurable benefits over and above reminding people to get vaccinated. This finding was contrary to the high expectations of both expert and lay forecasters. On a more positive note, our megastudy suggested that pharmacies and other vaccination providers in the United States have a cost-effective opportunity to increase vaccination rates by sending people a series of behaviourally informed text reminders to receive their COVID-19 booster each autumn. We identified three types of personalized text reminder that produced slightly increased expected benefits over and above other text reminders, which would be good candidates for widespread deployment. Notably, however, we estimated that a behaviourally informed text message reminder (designed to follow best practices in the academic literature by describing a vaccine as ‘recommended’ and ‘waiting for you’) achieved approximately 80% of the benefits obtained by our best-performing intervention. Moreover, using new behavioural insights to reword these text reminders can be credited with only approximately 20% of the measured benefit of our best-performing intervention.

We estimated that altogether, the text interventions we tested produced an additional 33,864 COVID-19 booster vaccinations (95% CI = 30,838–36,889) and 10,756 flu vaccinations (95% C = 7,985–13,527) in the autumn of 2022. Because COVID-19 booster vaccinations reduce infections by at least 43%^59^, compared with individuals who received their last monovalent COVID-19 dose at least 8 months before, these extra vaccinations probably prevented an estimated 1,857 infections (see Supplementary Information, section 6, for detailed calculations). It is likely that the benefits we see in our over 3.66-million-patient megastudy would generalize to other populations, which suggests that the scale of these benefits could potentially be increased by nearly two orders of magnitude with a national rollout of reminder messaging.

Even before the COVID-19 pandemic, the World Health Organization identified vaccine hesitancy as one of the top ten global public health threats^60^. The importance of finding effective ways to encourage vaccinations among populations that might otherwise neglect them has only grown. That is because vaccinations against COVID-19, flu, pneumonia, shingles, polio and other debilitating diseases avert millions of unnecessary deaths each year^61,62^, and vaccinations prevent even more unnecessary hospitalizations^63,64^, as well as staving off chronic health problems such as long COVID^65,66^. Our results point to the need for more rigorous testing of interventions to promote vaccination, which will help ensure that evidence-based solutions are deployed widely and that ineffective tools are discontinued. This is particularly important in light of our finding that scientific experts cannot accurately forecast what actually works to encourage vaccination nor are they well-calibrated when predicting how well interventions work.

Overall, our findings indicated that despite the optimism of lay and expert forecasters about free rides and simple reminder messages, most interventions tested produced relatively small absolute increases in vaccination rates. This means that work is needed to identify more potent methods of encouraging vaccine adoption to prevent more unnecessary hospitalizations and death from COVID-19, particularly in high-risk populations.

Our megastudy’s strengths include its large, diverse, national sample, its analysis of an objective measure of COVID-19 vaccination both 30 days and 90 days after intervention, its ability to measure spillover effects to flu vaccination decisions, and its simultaneous comparison of the impact of eight different interventions. However, a number of limitations are worth noting. First, our research focused on the benefits of providing patients in the United States with free Lyft rides to pharmacies for vaccination appointments, but our findings might have been different if we had offered free rides through another ridesharing app, by taxi or through some other service or if our test had been run in another country. Our findings also speak only to the benefit of providing free rides to vaccination sites and not to the benefits of finding other ways to increase the ease of vaccine access, such as bringing mobile vaccination clinics to remote communities^67^, which proved efficacious in Sierra Leone. Another limitation of our work is that Federal Communication Commission (FCC) regulations compelled us to conduct our megastudy only among individuals who had agreed to receive SMS messages from CVS Pharmacy. This sample of patients, although demographically and geographically diverse, is not necessarily representative of all populations that would benefit from the interventions tested here. It is important to note that our finding that free Lyft rides did not increase vaccinations might not hold in a sample of patients who had not previously found their way to a CVS Pharmacy for a COVID-19 vaccine. Ideally, future tests of the interventions evaluated here would be conducted with even more diverse populations and more diverse modes of transport. Another limitation of our study is that we were unable to measure the adoption of COVID-19 vaccines (or flu vaccines) at locations besides CVS Pharmacies. Although past work has found neither crowd-in nor crowd-out from reminder messages encouraging vaccination in a specific setting^42^, we are unable to rule out either possibility. Finally, although we were able to conduct heterogeneity analyses based on a patient’s age, sex, insurance type and previous vaccination history, one key unknown variable was the race of the patient. We obtained information about the racial composition of patients’ neighbourhoods to conduct heterogeneity analyses, but in light of racial disparities in vaccination, it would be ideal for future research studies with data on participant race to explore the relative impact of the interventions tested here on individuals from minority ethnic groups.

## Methods

### Ethics approval

The designs of our megastudy and our forecasting studies were reviewed and approved by the Institutional Review Board of the University of Pennsylvania. A waiver of informed consent was granted for our megastudy because of the following reasons: (1) it was deemed to pose minimal risk to patients; (2) it could not be practically carried out otherwise; and (3) only CVS Pharmacy patients who had already consented to receive SMS communications were included in the study.

### Megastudy participants

Megastudy participants were CVS Pharmacy patients who (1) were 18 years or older, (2) resided in one of 65 US metropolitan areas selected for study inclusion (see Supplementary Information, section 4 for a complete list), (3) had previously received at least their primary COVID-19 vaccine series but not the bivalent booster according to CVS Pharmacy records (only patients who had completed their primary COVID-19 vaccination series were eligible for a bivalent booster according to the US Food and Drug Administration), and (4) had consented in writing to receive text messages from CVS Pharmacy (this requirement was imposed to comply with the FCC’s Telephone Consumer Protection Act, which outlaws sending communications by text without an individual’s consent).

The average age of CVS Pharmacy patients in our megastudy was 47.30 years (s.d. = 17.15), and 40.43% of patients were male. Information on the race of a patient was not available. CVS Pharmacy used SMS short codes to contact all patients in our study, and for roughly 60% of patients, the SMS short code used to contact them was familiar—meaning it had been used to send that same patient one or more pharmacy-related messages (for example, about prescription refills) in the previous 22 months.

### Megastudy conditions and randomization procedures

Our megastudy included nine different conditions: eight intervention conditions and a holdout control condition. A patient’s condition determined which (if any) text messages they received from CVS Pharmacy reminding them to obtain a COVID-19 bivalent booster vaccine as part of this megastudy.

**Extended Data Fig. 2:**
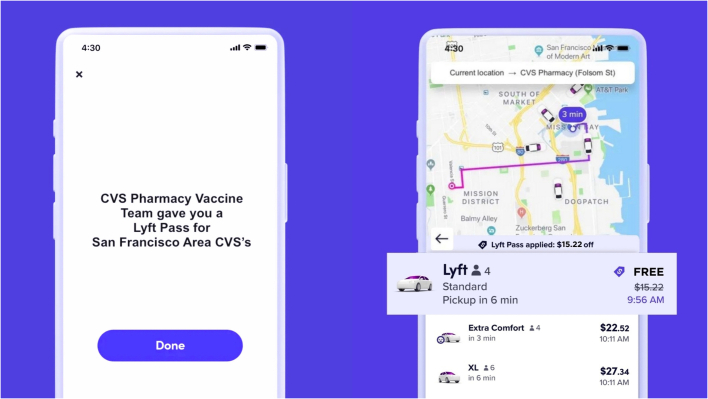
Illustration of the Lyft Pass programs’ interface displayed to patients who clicked the link in our reminder texts to claim (Panel A) and then use (Panel B) a free round-trip ride to CVS Pharmacy. *Note*: This illustration is for a hypothetical patient in the San Francisco-Oakland-Berkeley metropolitan area. Image of the Lyft app courtesy of Lyft, Inc. When patients clicked on the link in their text message to claim their round-trip Lyft ride, it opened their Lyft app and automatically stored our Lyft Pass as a payment option for a qualifying ride (see Panel A). When patients then booked a ride to a location that was within a 0.1 square mile radius of a CVS Pharmacy in their metropolitan area, the Lyft Pass was shown as their first payment option (see Panel B). Free rides could also be claimed by manually entering the code patients were supplied within our text message as a payment method within the Lyft app.

All intervention messages consisted of an initial set of reminder texts sent on 3 November (hereafter called day 1), 5 November (hereafter called day 2) or 8 November (hereafter called day 3) with a follow-up set of reminder texts sent 7 days later. All text reminders conveyed to patients that a vaccine was ‘recommended’ and ‘waiting for you’, building on past research^19–21^. Our key intervention—which was designed to test the value of free round-trip rides to vaccination sites—included this standard reminder language but also provided patients with one free round-trip ride to and from a CVS Pharmacy in the month ahead. The free ride was provided by Lyft (Extended Data Fig. 2), a popular ride-sharing company supporting over one quarter of all rideshare rides in the United States^68^. The Lyft codes provided were geofenced so that patients could only take the round-trip ride to and from the CVS Pharmacy locations in their metropolitan area (or subregion). CVS Pharmacy did not cover the cost of the Lyft rides or provide any incentive to patients in this study. All costs of the free Lyft rides to and from CVS Pharmacies were funded by the Social Science Research Council’s Mercury Project.

Our free-ride offering was designed to emulate the 2021 White House programme that offered people free rides by Lyft and Uber to and from vaccination sites for a limited time. Specifically, the free rides offered by the White House were available from 24 May to 4 July 2021 and required customers of Lyft (or Uber) to enter a claim code into their app to receive a free round trip ride (worth up to US$15 per ride at Lyft or up to $25 per ride at Uber^29,51,52^). Our programme arguably made claiming free rides slightly easier than the White House programme because it simply required one click on a link in our text message to accept our offer code (the code was also supplied directly for manual entry if preferred), and our offer was then automatically applied to the next qualifying ride taken to or from a CVS Pharmacy in the patient’s metropolitan area.

Patients encountered a price cap of $25 per ride only if they attempted to book a ride that exceeded this price limit, at which point they would be billed for spending in excess of $25. We estimated that in the zip codes where our test was conducted, the median resident lived 1.70 miles (2.7 km) from a CVS Pharmacy, such that the cost of a Lyft to or from the pharmacy would typically be under $10. Even patients living at the estimated 99th percentile distance from a CVS Pharmacy were only 9.40 miles (15.1 km) from a CVS Pharmacy, such that the cost of a Lyft to or from the pharmacy would typically be under $25 (see Supplementary Information, section 5 for calculation details and a complete distribution of distance and ride cost estimates).

The interventions tested that did not offer free round-trip Lyft rides to CVS Pharmacy in a standard reminder message instead layered a range of different strategies for encouraging immunization on top of the standard reminder, from conveying current (high) rates of infection in a patient’s county to providing resources to combat misinformation (see Table 1 for a summary of our eight interventions).

Randomization of each eligible participant to one of our nine megastudy conditions was conducted using data obtained from CVS Pharmacy on 18 October 2022 with the splitsample routine in Stata (v.17.0)^69^. Patients were assigned with equal probability to one of nine megastudy conditions except the intervention offering free round-trip Lyft rides to CVS Pharmacy—this intervention was capped at 50,000 people to ensure study costs would not exceed our budget. Owing to a technical error, reminder messages in two megastudy conditions (interventions 7 and 8 in Table 1) were not successfully sent on 3 November (day 1), and thus no follow-up reminder messages were sent to these intended study patients 1 week later either. These intended participants simply were not messaged or included in the megastudy. As a result, an average of 328,556 patients were included in two megastudy conditions (interventions 7 and 8), whereas an average of 492,573 patients were included in the megastudy’s remaining six conditions. See Fig. 1 for a CONSORT flow diagram depicting randomization.

### Megastudy data

All megastudy data supplied by CVS Pharmacy were de-identified through the Safe Harbor method pursuant to 45 Code of Federal Regulations 164.514(b)(2). Supplied data for each patient included sex, age, dates of all previous COVID-19 and flu vaccinations at CVS Pharmacy since 2020, primary insurance type and the zip codes of the CVS Pharmacy locations that were closest to the patient’s home, the most frequently visited and the site of the patient’s last COVID-19 vaccination. We merged in several additional variables that describe the composition of residents of the zip code or county of the CVS Pharmacy closest to the patient’s home address (see Supplementary Information sections 9 and 10 for details).

### Calculation of Bayes factors in support of null results

Throughout this article, we support null results by reporting approximate Bayes factors. For all nulls derived from linear probability models estimated using OLS regression, we first estimated the corresponding generalized linear model for binary data with the identity link function^70^ using Maximum Likelihood, then obtained the Bayesian information criterion (BIC) from the likelihood for both the null (that is, restricted) and the non-null (that is, full) model, and then tightly approximated the Bayes factor in support of the null hypothesis as Bayes factor ≈ exp([BIC_Full_ – BIC_Restricted_]/2)^71–73^. For a null result involving a continuous dependent variable, we tightly approximated the Bayes factor directly from the sums of squared errors of the null and non-null models^73^.

### Megastudy data analysis

We evaluated the impact of the eight interventions tested in our megastudy using a pre-registered OLS regression to predict vaccination within 30 days of the start of a patient’s intervention period (or control period). The start of a patient’s intervention period was defined as the (randomly assigned) date when they received their first reminder message (day 1, day 2 or day 3). The start of a patient’s control period was defined as the (randomly assigned) control period start date selected for purposes of comparison with the intervention conditions (day 1, day 2 or day 3). The key predictors in our regression were eight indicator variables for assignment to each intervention condition with an indicator for assignment to the holdout control condition omitted. We also included indicators for the date on which patients were assigned to receive their first reminder text (day 1 or day 2; day 3 was omitted). We estimated this regression with HC1 robust standard errors and adjusted all *P* values for multiple comparisons using the BH procedure^74^.

As noted above, a technical error prevented interventions 7 and 8 from deploying on day 1 of our study (so no patients were actually assigned to these interventions on day 1). To assess the ability of our pre-registered OLS regression to produce unbiased results despite the absence of patients in interventions 7 and 8 on launch day 1, we followed a method laid out in our second pre-registration (which was posted after this launch error became apparent but before any outcome data had been received by our research team). Specifically, we ran our standard OLS regression to predict vaccination within 30 days and added interaction terms between indicators for interventions 1–6 and launch day 1. We then conducted an undirected *F*-test assessing whether these interaction terms were jointly equal to zero. We failed to reject the null hypothesis (*P* = 0.140), which indicated a lack of heterogeneous treatment effects between launch day 1 and launch days 2 and 3 pooled (Extended Data Table 5). Furthermore, we found strong support for the null hypothesis that the effects of interventions 1–6 were identical on day 1 and days 2 and 3 pooled (Bayes factor ≈ 4.051 × 10^17^). Following our second pre-registration, we therefore proceeded with analysing data from all 25 available intervention-by-launch day combinations jointly and including indicators for the eight intervention conditions and launch days but no interaction terms.

In addition, after conducting our pre-registered OLS regression (outlined above) to evaluate the impact of our eight interventions on COVID-19 vaccinations, we ran a robustness check that included the following pre-registered additional controls: (1) age (as of October 2022); (2) an indicator for being 50 years or older; (3) an indicator for being male; (4) indicators for insurance type as of December 2022 (Medicare, Medicaid or unknown; commercial insurance omitted); (5) total number of previous COVID-19 vaccinations received at any CVS Pharmacy (as measured in December 2022); and (6) total number of previous COVID-19 boosters received at any CVS Pharmacy (as measured in December 2022). However, these robustness tests have the limitation that variables extracted in December 2022 were likely to be influenced patients’ condition assignment (that is, patients in megastudy conditions that produced more CVS Pharmacy visits for bivalent booster vaccinations apparently had more previous vaccinations ‘updated’, making it appear that they received more vaccinations pre-treatment than other patients; see discussion in the section ‘Megastudy to promote COVID-19 vaccination’).

In further robustness checks presented in Extended Data Table 2 and Supplementary Fig. 1, we also re-ran both regression specifications excluding all data from patients assigned to launch day 1 (because interventions 7 and 8 were not deployed on launch day 1).

### Forecasting experiment with laypeople

We recruited 216 US residents who were 18 years and older from Prolific (48.15% male; average age = 35.69 years, s.d. = 13.39 years) and paid them $1.40 to complete a 7-min forecasting survey. All participants were required to take our survey on a desktop computer or tablet rather than a mobile device to ensure images would display properly. All participants were told: “We’ll ask you to review nine different sets of text messages that encouraged pharmacy patients to get their bivalent COVID-19 booster in November 2022. We’ll ask you to predict the impact each message set had on bivalent booster vaccination uptake.” They then all learned about the inclusion criteria for patients in our vaccination megastudy and were told what fraction of patients in our holdout control condition received a vaccine within 30 days of our megastudy’s launch. Because we had not yet determined that data on patients’ vaccinations before 3 November 2022 were unreliable (because it was probably differentially influenced by patients’ condition assignment) at the time of these studies, we told survey forecasters that 5.31% of patients in our control condition had been vaccinated. At this point, the forecasters were required to pass a comprehension check before proceeding—17 laypeople did not do so, leaving 199 forecasters who completed our survey and are therefore included in all analyses (52.26% male; average age = 35.69, s.d. = 13.39).

Next, the forecasters were separately shown each of the different text messaging interventions that patients in our study could have received from their pharmacy. These messages were displayed overlaid on a mobile phone screen (as they would have appeared to recipients). After viewing each set of messages and being reminded what fraction of patients in our holdout control group received a booster vaccine within 30 days of our study’s launch, the forecasters were asked: “For patients who did receive the above text messages from their pharmacy—what percentage of them do you think got the bivalent COVID-19 booster at their pharmacy within 30 days of receiving the first message above? Please enter your response to the hundredth decimal place (for example, X.XX% or XX.XX%)”. For complete study stimuli, which were closely modelled on those used in past forecasting studies^21,46,75^, see Supplementary Information, section 12.

Although there were only eight intervention conditions in our megastudy, one of our interventions (intervention 4: infection rates) displayed a different message to patients who lived in US counties with above median infection rates in late October 2022. To simplify the way this was communicated to survey respondents, we showed forecasters each of these two message separately and then created a weighted average of their two forecasts (weighted proportionally to the number of megastudy patients who saw each version of intervention 4) to estimate the forecasts of the impact of intervention 4 on vaccination rates.

When depicting the free-ride intervention, we did not show forecasters the Lyft app screens they would have seen had they been in the megastudy and clicked the link in their intervention message to claim a free ride to CVS Pharmacy (see Supplementary Information, section 12, screen 7). Because so few individuals in our megastudy clicked the link to claim a free ride (see Supplementary Information, section 4), giving forecasters the information shown to this small subpopulation would have provided them with a nonrepresentative experience of our stimuli.

These forecasting procedures followed standard practices in the literature^21,46,75^. Although incentives are sometimes provided for forecasting accuracy, they often are not^21,46,75–77^.

To analyse the accuracy of the estimates of the forecasters, we calculated the absolute change in vaccination rates they forecasted. All results we describe are robust to instead analysing the percentage change in vaccination rates that were forecasted (Supplementary Information, section 11).

Extended Data Table 6 presents the median, mean and standard deviation of the predicted effectiveness of each intervention provided by lay forecasters. Extended Data Table 7 presents the average rank order of intervention performance based on laypeople’s forecasts of intervention efficacy as well as the fraction of laypeople who forecasted each intervention would be the top performer.

### Forecasting experiment with experts

We recruited 215 volunteer participants who held a PhD in psychology, economics, business or a related field in the social sciences (37.21% male; average age = 41.86 years, s.d. = 10.73 years) to complete our second forecasting survey. Participants were recruited by posting invitations on the Society for Judgement and Decision Making and the Economic Science Association listservs to anyone with the aforementioned qualifications to make predictions about “a study testing the efficacy of eight different sets of text messages encouraging people to get bivalent COVID-19 boosters”. Invitations to participate in the forecasting study were also posted on social media (Twitter and LinkedIn) in early 2023 by the study’s principal investigators with the message: “Can you predict what text messages worked best to increase bivalent COVID-19 booster vax rates this past fall? Do you have a PhD in #psych, #econ, #business, or a related field?”

The first question in our survey asked respondents to confirm that they held the requisite PhD. The remainder of the study procedures were identical to those described above for lay forecasters. Fifty-two individuals failed our attention check or dropped out of our survey before reaching it, leaving 163 participants who completed our survey and are therefore included in all analyses (49.07% male; average age = 41.86 years, s.d. = 10.73 years). For complete study stimuli, see Supplementary Information, section 12.

Extended Data Table 6 presents the median, mean and standard deviation of the predicted effectiveness of each intervention provided by expert forecasters. Extended Data Table 8 presents the average rank order of intervention performance based on the forecasts by experts of intervention efficacy as well as the fraction of experts who forecasted each intervention would be the top performer.

### Reporting summary

Further information on research design is available in the Nature Portfolio Reporting Summary linked to this article.

## Online content

Any methods, additional references, Nature Portfolio reporting summaries, source data, extended data, supplementary information, acknowledgements, peer review information; details of author contributions and competing interests; and statements of data and code availability are available at 10.1038/s41586-024-07591-x.

### Supplementary information

Supplementary Information This supplementary information file contains 67 display items (5 figures and 62 tables). It also includes additional information about the following: (1) our megastudy’s implementation, (2) forecasting study stimuli, (3) subgroup and heterogeneity analyses, and (4) robustness checks. These materials supplement our main article by providing further details on methodology and analyses

Reporting Summary

## Figures and Tables

**Extended Data Table 1:**
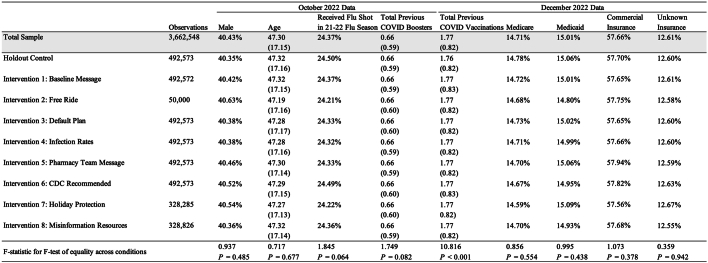
Patient summary statistics and balance test results

*Note*. This table reports means for patient-level variables provided by CVS Pharmacy (and standard deviations in parentheses for continuous variables) for our full patient sample and for the subset of patients in each study condition. Statistical tests involving multiple regression coefficients are all undirected.

**Extended Data Table 2:**
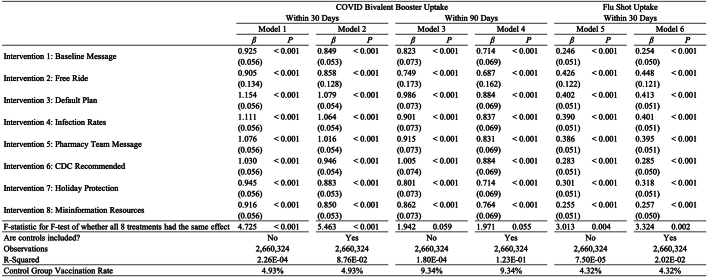
Regression-estimated impact of each of our megastudy’s eight intervention conditions including only patients who were assigned to launch day 2 or 3

*Note:* This table reports the results of six OLS regressions; the primary predictor variables are eight indicators for assignment to each of our eight intervention conditions. All models also include an indicator for whether the patient received their first text message on launch day 2. In Models 2, 4 and 6, controls are also included for the patient’s age in October 2022; whether the patient’s age was ≥ 50 in October 2022; whether the patient was male; and indicators for the patient’s insurance status (Medicare, Medicaid, or unknown; commercial insurance is omitted) in December 2022. Models 2 and 4 also control for the patient’s number of previous COVID-19 boosters prior to the start of the study according to records as of October 2022 and their number of previous COVID-19 vaccinations prior to the start of the study according to records as of December 2022. Model 6 includes an indicator for whether a patient received a flu shot at CVS Pharmacy during the 2021–2022 flu season. The control variables in all models are mean-centered using the mean of the holdout control. Regression coefficients and standard errors have been multiplied by 100 to improve interpretability. Standard errors reported in parentheses are estimated robustly using HC1. P-values are adjusted for multiple comparisons using the Benjamini-Hochberg procedure. Statistical tests of whether an individual regression coefficient is zero are all two-sided. Statistical tests involving multiple regression coefficients are all undirected.

**Extended Data Table 3:**
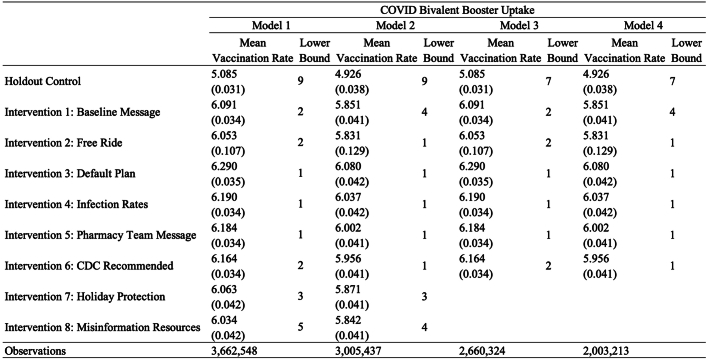
Tests of ranks of interventions

*Note:* This table presents the estimated mean vaccination rates and standard errors from 4 variants of our main regression model (Table 2, Model 1). The number in the column “Lower Bound” is the lowest value in each condition’s one-sided 95% confidence set of its true rank, based on the set of nine regression-estimated bivalent COVID-19 booster vaccination rates. Model 1 is estimated on the full study sample. Model 2 excludes data from patients assigned to launch day 1. Model 3 excludes data from patients assigned to conditions 7 or 8. And Model 4 excludes data from patients assigned to launch day 1 as well as patients assigned to conditions 7 or 8. The results for Models 1 and 3 indicate that interventions 3, 4, and 5 are the only interventions for which we cannot reject the null hypothesis (at 95% confidence) that they are the true best-performing intervention for the data including launch day 1. Results for Models 2 and 4 indicate that interventions 2, 3, 4, 5, and 6 are the only interventions for which we cannot reject the null hypothesis (at 95% confidence) that they are the true best-performing intervention for the data excluding launch day 1.

**Extended Data Table 4:**
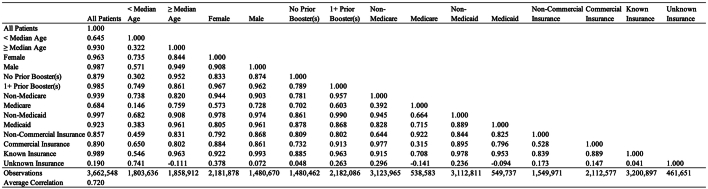
Correlations between the eight estimated intervention effects for all patients in our main regression model (Column 1) and the same eight estimated intervention effects for different patient subgroups (e.g., males) based on individual patient characteristics

*Note:* This table reports the Pearson correlation coefficients between the eight treatment effects estimated in our main regression model (see Table 2, Model 1) and the eight treatment effects estimated in patient-level subgroup analyses (see Tables S4–S17, Model 1) following the same regression specification as our main regression model but including only patients from the subgroup of interest.

**Extended Data Table 5:**
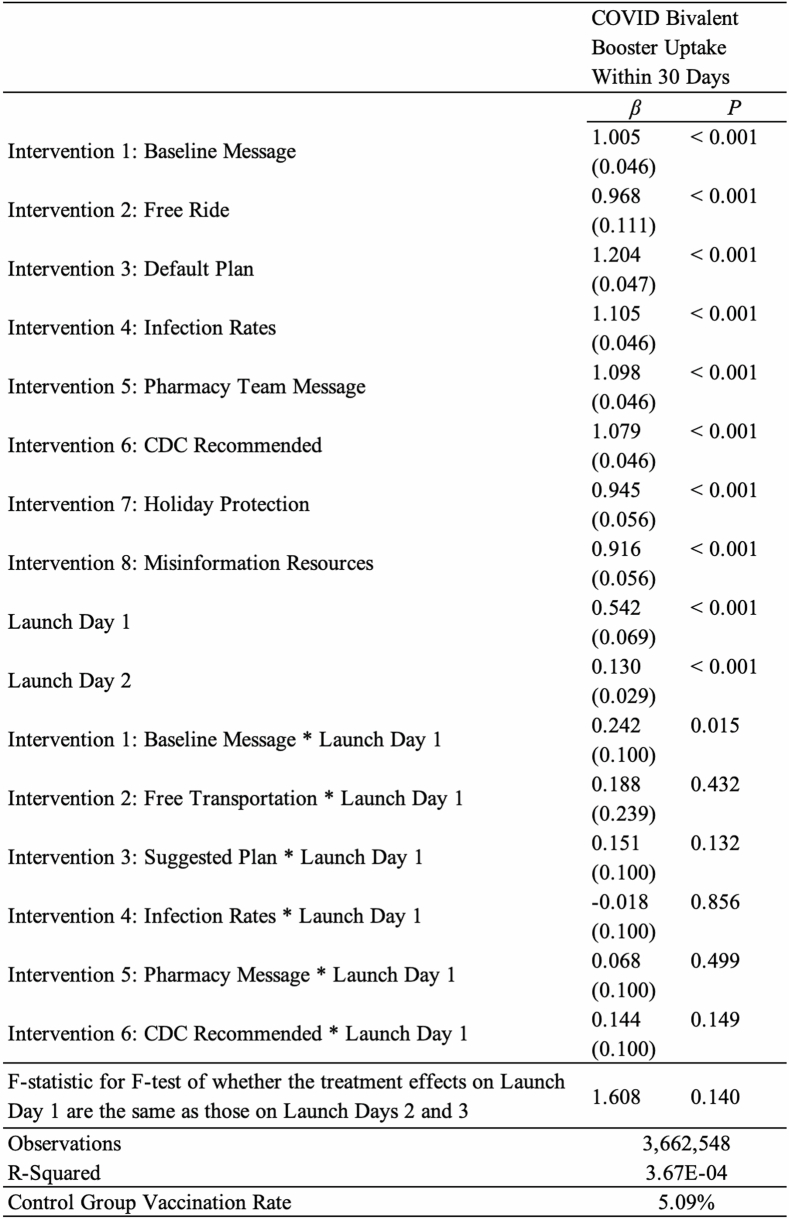
Regression-estimated impact of each of our megastudy’s eight intervention conditions allowing for treatment effect heterogeneity by patient assignment to launch day 1

*Note*. This table reports the results of an ordinary least squares (OLS) regression that predicts whether a given patient received a bivalent COVID-19 booster at a CVS Pharmacy within 30 days of a patient’s study launch day. The primary predictor variables in the regression are eight indicators for assignment to each of our megastudy’s eight intervention conditions (omitting the holdout control condition), indicators for whether a patient’s first message was sent on launch day 1 and launch day 2 (omitting launch day 3) mean-centered using the mean of the holdout control, and interactions between the mean-centered launch day 1 indicator and each of the six indicators for assignment to an intervention condition that launched successfully on launch day 1. The reported two-sided F-statistic tests the null hypothesis that all of the interaction terms are jointly equal to 0. All regression coefficients and standard errors have been multiplied by 100 to improve interpretability (and thus reflect percentage point change(s) induced in vaccination uptake). Standard errors reported in parentheses are estimated robustly using HC1. Statistical tests of whether an individual regression coefficient is zero are all two-sided. Statistical tests involving multiple regression coefficients are all undirected.

**Extended Data Table 6:**
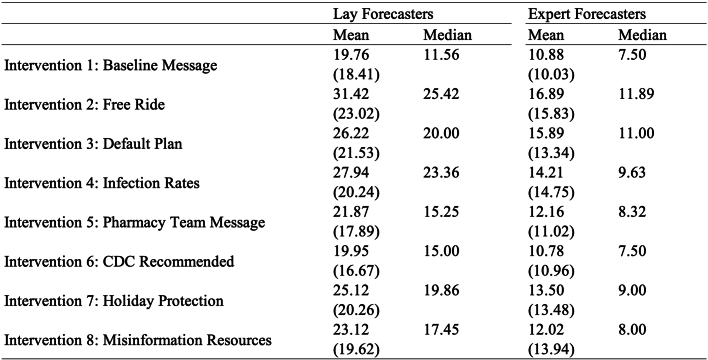
Median, mean, and standard deviation of predicted rate of vaccination of each intervention by lay and expert forecasters

*Note:* Standard deviations are reported in parenthesis.

**Extended Data Table 7:**
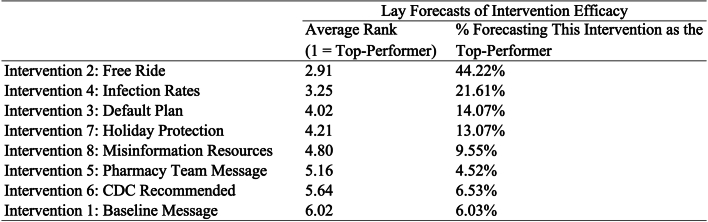
The rank order of interventions’ forecasted performance according to laypeople

*Note*. The first column is based on laypeople’s average forecasts of intervention efficacy, and the second is based on the fraction of laypeople who forecasted each intervention would be the top-performer in the set.

**Extended Data Table 8:**
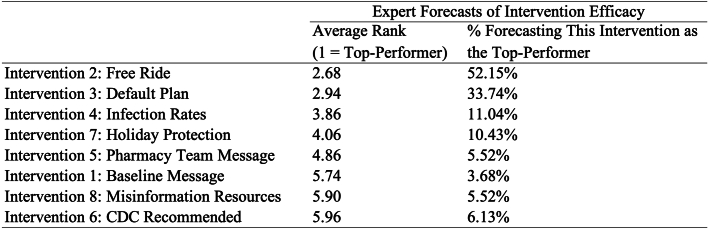
The rank order of intervention’s forecasted performance according to experts

*Note*. The first column is based on experts’ average forecasts of intervention efficacy, and the second is based on the fraction of experts who forecasted each intervention would be the top-performer in the set.

## Data Availability

The experimental data analysed in this article were provided by CVS Pharmacy. The data were de-identified pursuant to 45 CFR 164.514(b)(2). Our study’s analysis plan was pre-registered on the Open Science Framework (OSF; pre-registration 1: bit.ly/3n3KUh2; pre-registration 2: bit.ly/3ZRPzBk). Pre-registration 2 updates part of pre-registration 1 to address unexpected problems stemming from the failure to send messages, as planned, in interventions 7 and 8 on 3 November. This second pre-registration was posted before any outcome data were received or analysed. Fully anonymized and de-identified data on each study participant’s intervention condition, launch date and bivalent COVID-19 booster vaccination decision during our follow-up period are available on the OSF as are aggregated summary statistics (https://bit.ly/3MhRHgm). However, to protect patient privacy, we cannot publicly post individual-level data on patients’ covariates. Source data are provided with this paper.

## Code availability

All codes used to analyse study data can also be found at the OSF (https://bit.ly/3MhRHgm).
