# Supplementary material for: Megastudy shows that reminders boost vaccination but adding free rides does not
Source: Nature. 2024 Jun 26;631(8019):179–88. doi: 10.1038/s41586-024-07591-x (PMC11222156; doi:10.1038/s41586-024-07591-x)
Supplement: Supplementary file 1 — This supplementary information file contains 67 display items (5 figures and 62 tables). It also includes additional information about the following: (1) our megastudy’s implementation, (2) forecasting study stimuli, (3) subgroup and heterogeneity analyses, and (4) robustness checks. These materials supplement our main article by providing further details on methodology and analyses [file 41586_2024_7591_MOESM1_ESM.docx]

Supplementary Materials:

# **TITLE:** Megastudy shows reminders boost vaccination but adding free rides doesn’t

**AUTHORS:** Katherine L. Milkman^1,*^, Sean F. Ellis^2^, Dena M. Gromet^2^, Youngwoo Jung^2^, Alex S. Luscher^2^, Rayyan S. Mobarak^3^, Madeline K. Paxson^2^, Ramon A. Silvera Zumaran^2^, Robert Kuan^1^, Ron Berman^4^, Neil A. Lewis Jr.^6^, John A. List^7^, Mitesh S. Patel^8^, Christophe Van den Bulte^4^, Kevin G. Volpp^9^, Maryann V. Beauvais^10^, Jonathon K. Bellows^10^, Cheryl A. Marandola^10^, Angela L. Duckworth^1,5^

**AFFILIATIONS:**

^1^ Department of Operations, Information and Decisions, The Wharton School, University of Pennsylvania; Philadelphia, PA 19104, USA

^2^ Behavior Change for Good Initiative, The Wharton School and the School of Arts & Sciences, University of Pennsylvania; Philadelphia, PA 19104, USA

^3^ Department of Agricultural and Resource Economics, University of Maryland, College Park, MD 20742, USA

^4^ Department of Marketing, The Wharton School, University of Pennsylvania; Philadelphia, PA 19104, USA

^5^ Department of Psychology, University of Pennsylvania; Philadelphia, PA 19104, USA

^6^ Department of Communication, Cornell University; Ithaca, NY 14853, USA

^7^ Department of Economics, University of Chicago; Chicago, IL 60637, USA

^8^ Clinical Transformation and Behavioral Insights, Ascension Health; St. Louis, MO 63105, USA

^9^ Penn Center for Health Incentives and Behavioral Economics, Departments of Medical Ethics and Health Policy and Medicine, Perelman School of Medicine, University of Pennsylvania; Philadelphia, PA 19104, USA

^10^ CVS Health; Woonsocket, RI 02895, USA

*Corresponding author, email: kmilkman@wharton.upenn.edu.

**Table of Contents**

[1. Clarification of University of Pennsylvania and CVS Pharmacy agreement to conduct this study 3](#_qrersi499hy7)

[2. Sample Size 3](#_1fob9te)

[3. Balance tests demonstrating a change in measurement of prior vaccinations over time 4](#_enmtrnaft7fh)

[4. Information about how free Lyft rides were provided 7](#_3znysh7)

[5. Estimating (A) the Distance between Patients in Our Study and their Nearest CVS Pharmacy as well as (B) the Implied Cost of Lyft Rides from Patients’ Homes to their Nearest CVS Pharmacy 12](#_y56upxwm1qvi)

[6. Information on estimates of how many infections our interventions prevented 14](#_2et92p0)

[7. Details of test ranking intervention performance using confidence sets 15](#_tyjcwt)

[8. Information about de-identified patient neighborhood data that was incorporated into patient subgroup or heterogeneity analyses 16](#_3dy6vkm)

[9. Subgroup analyses 17](#_1t3h5sf)

[10. Heterogeneity analyses 18](#_tp3juexkgois)

[11. Robustness checks of analysis of experts’ and laypeople’s forecasts of our interventions’ effects 19](#_4d34og8)

[12. Forecasting study stimuli 23](#_17dp8vu)

[13. Figures 37](#_35nkun2)

[14. Tables 42](#_1ksv4uv)

[15. Supplementary Materials References 104](#_44sinio)

# **1. Clarification of University of Pennsylvania and CVS Pharmacy agreement to conduct this study**

The University of Pennsylvania’s Behavior Change for Good Initiative and CVS Pharmacy entered into an agreement to work on this study to better understand whether different text verbiages impacted patients’ decision to receive the bivalent COVID-19 booster vaccine. All costs incurred were borne by each organization separately and CVS Pharmacy did not receive funding to share de-identified data. CVS Pharmacy shares data under the security and privacy guidelines of Code of Federal Regulations (CFR) regarding public welfare (Title 45) with administrative data standards and related requirements of the Department of Health and Human Services, which states implementation specification requirements pertaining to use of protected health information. The data was de-identified pursuant to 45 Code of Federal Regulations 164.514(b)(2) prior to being shared with the University of Pennsylvania. No personal identifiable information (PII) or protected health information (PHI) was exchanged. The individuals who received the text messages in this study had Telephone Consumer Protection Act (TCPA) consent to receive text messages noted in their CVS Pharmacy profiles at the time of patient selection and outreach.

# **2. Sample Size**

CVS Pharmacy initially told us to assume we would only be able to include 2.6 million participants in our study, although they allowed us to randomize 4.9 million participants for possible inclusion. The estimate that we would be able to include 2.6 million people was based on (1) the maximum number of text messages that CVS Pharmacy thought they would be permitted to send during the three days allocated to our study (given capacity constraints), and (2) a very upwardly biased (and thus conservative) forecast of the maximum total number of patients from our randomized sample who would (a) get COVID booster shots after the date of randomization and before treatment deployment and (b) who would become ineligible for study inclusion for some other reason. Fortunately, it turned out that CVS Pharmacy was able to send reminder messages to all randomized patients who were eligible on the day of treatment deployment. Because of this, we ended up with a far larger final sample than we expected and pre-registered.

#

# **3. Balance tests demonstrating a change in measurement of prior vaccinations over time**

Prior to the launch of our megastudy in October 2022, we conducted checks to ensure that the (de-identified) patients who were assigned to different experimental conditions and launch days were balanced on observable features. The observable patient features available to us in October 2022 included patient gender, age, total number of prior COVID-19 booster vaccinations, and vaccination for flu in the previous flu season. To check for balance across our experiment’s nine conditions, we regressed each covariate on indicators for our eight intervention conditions, with the holdout control condition omitted, using an ordinary least squares (OLS) regression model with HC1 robust standard errors. We then conducted undirected F-tests to test the null hypothesis that the coefficients from the eight intervention conditions were jointly equal to zero (see Extended Data Table 1). Results from these undirected F-tests suggest that all covariates were balanced (all p’s > 0.05).

To check for balance across our experiment’s 25 experimental condition and launch day- combinations (interventions 1 through 6 and the holdout control condition across all 3 launch days and interventions 7 and 8 across launch days 2 and 3), we regressed each covariate on indicators for our 24 condition-by-launch-day pairs with the holdout control condition on launch day 3 omitted, again using an OLS regression with HC1 robust standard errors. We then conducted undirected F-tests to test the null hypothesis that the coefficients from the 24 indicators were jointly equal to zero (see Extended Data Table 1). Results of these undirected F-tests suggest that all covariates were balanced (all p’s > 0.05).

In December 2022 after the launch of our megastudy, we received the first round of de-identified study data from CVS Pharmacy, which included updated data pulls on patients’ insurance status (Medicare, Medicaid, unknown, or commercial) and on patients’ total number of previous COVID-19 vaccinations. We ran the same balance checks described above on these two new covariates and found that while insurance status was balanced (all p’s > 0.05), the total number of prior COVID-19 vaccinations was not balanced (undirected F-test p < 0.001): patients in our study’s eight intervention conditions appeared to have received significantly more COVID-19 vaccinations prior to the start of our intervention than patients in the holdout control condition. This imbalance was systematic enough to suggest that patients who were nudged to receive November vaccinations and thus made more pharmacy visits to receive vaccines may have been more likely to ask a pharmacist to update any incomplete historical records of vaccines received at other (non-CVS Pharmacy) locations in the CVS Pharmacy records system.

When we discussed this with CVS Pharmacy representatives, we were told that this was possible for the following reasons. CVS Pharmacy does not necessarily have a complete record of a patient’s COVID-19 vaccine history. Since the roll out of the first COVID-19 vaccines, medical organizations have strived to vaccinate all clinically eligible patients as quickly as possible at any viable location (e.g. a patient may have received their first and/or second dose from their primary care physician and then their monovalent booster from a CVS Pharmacy). When a patient comes into a pharmacy to get a COVID-19 vaccine, they can update their record to include the COVID-19 vaccines they received elsewhere (e.g. from their primary care physician or another pharmacy).

Data on vaccinations can also change over time because it is difficult to track if a dose is a primary series vaccine or a monovalent booster because the National Drug Code (NDC) was the same in most cases. Also, CVS Pharmacy’s process of ingesting data from one system to another and then cleaning it takes time, which can result in delays that impact the analysis of cross-sectional data.

The possibility that these issues affected the data on patients’ total number of previous COVID-19 vaccinations is confirmed by further examination of the data on patients’ total number of prior COVID-19 booster vaccinations, which we determined changed depending on the date of the data pull (we received data in October 2022, December 2022, and February 2023; see Table S58). We observe that the regression-estimated average within-person increase in COVID-19 booster vaccinations received before September 1, 2022 is +1.21 percentage points between October 2022 to December 2022 and this quantity increases by an additional +0.43 percentage points between December 2022 and February 2023 (all p’s < 0.001). We infer the imbalance in patients’ total number of COVID-19 vaccinations prior to September 1, 2022 is the result of patient-driven updating of historical COVID-19 vaccination records given that randomization was successfully balanced on all observable patient characteristics measured in October 2022 and there is a channel through which patients could update incomplete prior vaccination histories when visiting a CVS Pharmacy after the start of our study. Our study results show that patients reminded to get bivalent COVID-19 booster vaccines go to the pharmacy for those vaccines at a higher rate than others. It is natural that patients who make additional visits to CVS pharmacy for additional vaccines are more likely to update their past vaccine records (which offers a natural explanation for why those who were reminded to get COVID-19 boosters appeared, after-the-fact, to have more prior COVID vaccines than those who weren’t reminded to visit the pharmacy).

As a result of the challenges in tracking patients’ historical COVID-19 vaccinations prior to the launch of our study encouraging bivalent COVID-19 booster adoption, the measures available to us of patients’ prior vaccinations are actually biased proxies for prior vaccination decisions. We therefore reverted to our secondary pre-registered regression specification as our main analysis because it does not include any control variables. This is preferable to controlling for historical vaccination rates, the measurement of which was likely affected by assignment to one of our intervention conditions (although we also report our analyses including all pre-registered covariates to demonstrate the robustness of our findings).

# **4. Information about how free Lyft rides were provided**

We focused our study on the 65 largest metropolitan statistical areas (MSA) in the fifty U.S. states in which Lyft operates. According to the U.S. Census Bureau, “the general concept of a metropolitan or micropolitan statistical area is that of a core area containing a substantial population nucleus, together with adjacent communities having a high degree of economic and social integration with that core”^1^. Only CVS Pharmacy patients whose CVS Pharmacy was located in or around one of the 65 largest MSAs where Lyft operates were eligible to participate in our study.

To provide some patients in this megastudy with access to free round-trip Lyft rides, 70 regional “Lyft Pass programs” were created, and patients could receive a free ride to and from any CVS Pharmacy within the boundaries of the program. The boundaries of these programs were determined by first looking at the “core” or “principal city” of each MSA included in our study (MSAs can have more than one principal city). If an MSA had less than 150 pharmacy locations in it (the maximum number of specific destinations addresses that could be tagged as “free ride” destinations in a Lyft Pass program), we included all of them in the Lyft Pass program for a given patient in the MSA region and then added additional locations from its bordering zip codes until we reached 150 locations. When adding bordering zip codes, we started with the surrounding county with the most CVS Pharmacy locations and chose the bordering zip code with the most CVS Pharmacy locations in it. If we added all of a bordering county’s zip codes that bordered an MSA and were still not at 150 locations, we moved onto the surrounding county with the second most CVS Pharmacy locations, repeating this process and radiating outward until we reached 150 locations. If two surrounding counties or bordering zip codes had the same number of CVS Pharmacy locations, a coin was flipped to determine which would be chosen first. This rule applied to 54 of the 65 MSAs.

If an MSA had more than 150 pharmacy locations, which the eleven largest did, we first added all the CVS Pharmacy locations in the principal city to the Lyft Pass program. We then added pharmacy locations in bordering zip codes following the same process described above for adding pharmacy locations to the MSAs with less than 150 pharmacy locations. Three of the 11 largest MSAs had to be divided into multiple Lyft Pass programs because their principal cities, when combined, had more than 150 pharmacy locations. This increased the number of Lyft Pass programs from 65 to 70. The creation of these programs followed the procedure described above.

Using the metropolitan divisions defined by the United States Office of Management and Budget, the New York-Newark-Jersey City MSA was divided into its four metropolitan divisions of New York, Newark, Nassau-Suffolk, and New Brunswick-Lakewood. The Los Angeles-Long Beach Anaheim MSA was divided into its two metropolitan divisions of Los Angeles-Long Beach-Glendale and Anaheim Santa Ana-Irvine. The Dallas-Fort Worth-Arlington MSA was divided into its two metropolitan divisions of Dallas-Plano-Irving and Fort Worth-Arlington-Grapevine^2^.

The 65 MSAs included in our experiment were:

1. New York-Newark-Jersey City, NY-NJ-PA MSA
2. Los Angeles-Long Beach-Anaheim, CA MSA
3. Chicago-Naperville-Elgin, IL-IN-WI MSA
4. Dallas-Fort Worth-Arlington, TX MSA
5. Houston-The Woodlands-Sugar Land, TX MSA
6. Washington-Arlington-Alexandria, DC-VA-MD-WV MSA
7. Philadelphia-Camden-Wilmington, PA-NJ-DE-MD MSA
8. Miami-Fort Lauderdale-Pompano Beach, FL MSA
9. Atlanta-Sandy Springs-Alpharetta, GA MSA
10. Boston-Cambridge-Newton, MA-NH MSA
11. Phoenix-Mesa-Chandler, AZ MSA
12. San Francisco-Oakland-Berkeley, CA MSA
13. Riverside-San Bernardino-Ontario, CA MSA
14. Detroit-Warren-Dearborn, MI MSA
15. Seattle-Tacoma-Bellevue, WA MSA
16. Minneapolis-St. Paul-Bloomington, MN-WI MSA
17. San Diego-Chula Vista-Carlsbad, CA MSA
18. Tampa-St. Petersburg-Clearwater, FL MSA
19. Denver-Aurora-Lakewood, CO MSA
20. Baltimore-Columbia-Towson, MD MSA
21. St. Louis, MO-IL MSA
22. Orlando-Kissimmee-Sanford, FL MSA
23. Charlotte-Concord-Gastonia, NC-SC MSA
24. San Antonio-New Braunfels, TX MSA
25. Portland-Vancouver-Hillsboro, OR-WA MSA
26. Sacramento-Roseville-Folsom, CA MSA
27. Pittsburgh, PA MSA
28. Austin-Round Rock-Georgetown, TX MSA
29. Las Vegas-Henderson-Paradise, NV MSA
30. Cincinnati, OH-KY-IN MSA
31. Kansas City, MO-KS MSA
32. Columbus, OH MSA
33. Indianapolis-Carmel-Anderson, IN MSA
34. Cleveland-Elyria, OH MSA
35. San Jose-Sunnyvale-Santa Clara, CA MSA
36. Nashville-Davidson-Murfreesboro-Franklin, TN MSA
37. Virginia Beach-Norfolk-Newport News, VA-NC MSA
38. Providence-Warwick, RI-MA MSA
39. Milwaukee-Waukesha, WI MSA
40. Raleigh-Cary, NC MSA
41. Richmond, VA MSA
42. Louisville/Jefferson County, KY-IN MSA
43. New Orleans-Metairie, LA MSA
44. Salt Lake City, UT MSA
45. Hartford-East Hartford-Middletown, CT MSA
46. Buffalo-Niagara Falls, NY MSA
47. Birmingham-Hoover, AL MSA
48. Rochester, NY MSA
49. Grand Rapids-Kentwood, MI MSA
50. Tucson, AZ MSA
51. Urban Honolulu, HI MSA
52. Tulsa, OK MSA
53. Fresno, CA MSA
54. Worcester, MA-CT MSA
55. Omaha-Council Bluffs, NE-IA MSA
56. Bridgeport-Stamford-Norwalk, CT MSA
57. Greenville-Anderson, SC MSA
58. Albuquerque, NM MSA
59. Bakersfield, CA MSA
60. Albany-Schenectady-Troy, NY MSA
61. Knoxville, TN MSA
62. McAllen-Edinburg-Mission, TX MSA
63. Baton Rouge, LA MSA
64. El Paso, TX MSA
65. Allentown-Bethlehem-Easton, PA-NJ MSA

CVS Pharmacy did not cover the costs of any Lyft rides or provide any vaccine incentives to patients in our study. All costs associated with the free Lyft rides to and from CVS Pharmacies offered to patients in this experiment were covered by the Social Science Research Council’s Mercury Project funding.

Extended Data Figure 2 provides a rough illustration of what the Lyft interface looked like for patients who clicked the link in our reminder texts to claim a free round-trip ride to CVS Pharmacy. Only 0.1% of patients offered a free round-trip Lyft ride to a CVS Pharmacy claimed one. We see that 2.42% of patients clicked on the CVS Pharmacy scheduler link in the Lyft messages, a click-through rate that fell slightly below the range of click through rates observed in other intervention conditions of 2.91% to 5.09% (see Table S59). In order to claim our Lyft pass, patients needed to have or create a Lyft account. Visit <https://www.lyft.com/rider/lyftpass> for more details on how Lyft Pass programs of the type we set up for our megastudy provide riders with easy access to free rides.

# **5. Estimating (A) the Distance between Patients in Our Study and their Nearest CVS Pharmacy as well as (B) the Implied Cost of Lyft Rides from Patients’ Homes to their Nearest CVS Pharmacy**

To estimate how far the average patient in a given zip code in our study lived from a vaccine dispensing CVS Pharmacy, we followed this procedure:

1. For every zip code in our study with at least 1,000 addresses present in the U.S. Department of Transportation’s National Address Database (National Address Database, 2023), we pulled 25 randomly selected addresses. We were able to follow this procedure for 50.9% of the 4,390 zip codes in our study (including 50.3% of the patients in our study).
2. For the remaining zip codes represented in our study (without at least 1,000 addresses present in the US DOT National Address Database), we randomly generated latitude and longitude points that fell between the zip code’s maximum and minimum latitude and longitude and selected the closest physical address to each point using Google Maps’ API in November 2023. If a randomly generated address fell outside of the relevant zip code, it was discarded and another latitude and longitude point was generated. This process was repeated until we either identified 25 addresses in the zip code of interest, or tried 50 times. (We limited our number of attempts to 50 due to the cost of each request of Google Maps’ API.) We followed this procedure successfully in 46.7% of the 4,390 zip codes in our study (which included 47.4% of the patients in our study).
3. For 2.3% of the zip codes in our study, after 50 tries with the latitude/longitude method detailed above, we had successfully identified 0 real addresses in the zip code. In these cases, we used Google Maps’ API to find the address geographically closest to the center of the zip code.

Once we had identified a set of 1–25 addresses using the aforementioned procedure for all zip codes in our study (25 for the median zip code) we calculated the driving distance between each address in each zip code and every CVS Pharmacy in that zip code to identify the nearest within-zip code CVS Pharmacy (according to its driving distance assessed using Google Maps’ API).^^[[1]](#footnote-1)^^

Figure S2 shows the distribution of all the address data collected. Using our sampling method, the median distance to a CVS Pharmacy for a patient living at a randomly selected address in a given zip code from our study is 1.70 miles, the 75th percentile distance is 2.80 miles, and the 90th percentile distance is 4.50 miles, suggesting most patients in our study would not need to take long Lyft rides to reach a CVS Pharmacy.

We estimated the price for the median ride, the 75th percentile ride, and the 90th percentile ride in each of the MSAs included in our study based on available fall 2023 pricing^3^ (which should be a bit higher than fall 2022 pricing due to inflation), and we report these data in Table S60, which illustrate that rides to a CVS Pharmacy that exceeded our $25 price cap should be incredibly rare in the locales we studied.

Note that Lyft uses some surge pricing (particularly during rush hour according to the member of our team who was Lyft’s Chief Economist from 2018–2022, which notably is not the most popular timeframe for vaccinations; the most popular time for getting a vaccine at CVS Pharmacy is midday). However, a case study of Lyft prices in Pittsburgh in 2017 found that less than 10% of rides involved surge pricing (Battifarano & Qian, 2019). Since then, Lyft's CEO has expressed a desire to get rid of surge pricing entirely^14^. As a result, our estimates focus on standard pricing.

# **6. Information on estimates of how many infections our interventions prevented**

We estimate that our interventions resulted in an additional 33,864 patients getting the bivalent COVID-19 booster by taking the product of each experimental condition’s sample size and its corresponding regression-estimated treatment effect and then summing across conditions (e.g., in the suggested plan intervention, 492,573 patients * 1.204% treatment effect = 5,931 additional vaccinations). We then use this number as an input to estimate the number of COVID-19 infections we could expect were prevented in the 8 months after our study launched. We begin with the Centers for Disease Control and Prevention (CDC)’s estimate of the average weekly percent of the U.S. population infected with COVID-19 that had received at least their COVID-19 primary series vaccinations but not their bivalent booster vaccine. For the week starting on November 6, 2022 through the week ending on February 18, 2023, the average weekly COVID-19 cases per 100,000 people in this population was 92^4^. The CDC estimates that the rate of unreported COVID-19 cases is at least 4 times that of confirmed cases^5^, meaning the average weekly rate of COVID-19 in the U.S. population during this period was actually 92*4 = 368 people per 100,000. Making the rough estimate that infection rates would, on average, remain at this level over the eight months after our intervention, this roughly translates to an 8-month COVID-19 infection probability of (368/100,000)*(34.66 weeks in eight months) = 12.75%. In the 8 months after our megastudy, 12.75% of the 33,864 patients we caused to get boosted would therefore have been expected to contract COVID-19 absent our intervention, or 4,318 patients. However, because the bivalent COVID-19 booster reduces the likelihood of infection by at least 43% ^6^, compared to patients who received their last monovalent COVID-19 dose at least eight months prior, we estimate that these interventions prevented 43% of the 4,318 expected infections among unboosted patients, or 1,857 infections.

# **7. Details of test ranking intervention performance using confidence sets**

To determine which of the interventions tested were the likely top-performer, we applied the confidence sets procedure for ranks developed by Mogstad et al. (2023)^7^. Marginal, one-sided, 95% confidence intervals were constructed for the rank of each megastudy condition (all eight interventions as well as the holdout control condition) based on the distribution of each condition’s regression-estimated bivalent COVID-19 booster vaccination rate during our study’s 30-day follow-up period. In Extended Data Table 4, we report the lower bounds of the confidence sets of the ranks. The COVID-19 booster vaccination rates in all models were estimated by using a variant of our main regression model in which we suppressed the intercept and added an indicator for the holdout control condition. Model 1 includes all eight interventions and all 3 launch days, Model 2 includes all 8 interventions and launch days 2 and 3, Model 3 includes interventions 1 through 6 and all 3 launch days, and Model 4 includes interventions 1 through 6 and launch days 2 and 3. The results from Models 1 and 3 show that the true top performing intervention is likely interventions 3, 4 or 5. The other interventions can be ruled out, at 95% confidence, from being the true top performer. Results for Models 2 and 4 (which omit data from launch day 1) indicate that the true top performing intervention is likely interventions 2, 3, 4, 5, or 6. The other interventions can be ruled out, at 95% confidence, from being the true top performer. Note that this widening of the confidence set in Models 2 and 4 after omitting data from launch day 1 is not driven by changes in point estimates but rather by a reduction in the precision of estimates when substantial data (from launch day 1) is omitted from Models 2 and 4.

# **8. Information about de-identified patient neighborhood data that was incorporated into patient subgroup or heterogeneity analyses**

In the subgroup and heterogeneity analyses, we analyzed several variables that describe the composition of patients’ neighborhoods based on the zip code or county of their closest CVS Pharmacy. Estimates of the percentage of residents of a specific race (White, Black, Asian or Hispanic) from each zip code were supplied by CVS Pharmacy in October 2022. We obtained zip code level data on median income^8^ and the percent of the population with a Bachelor’s degree^9^ from the U.S. Census Bureau. To create a measure of the density around the relevant CVS Pharmacy, we calculated the number of residents per square mile in a zip code using U.S. Census Bureau data on population^10^ and geographic size^11^. Data on the percent of residents in a relevant CVS Pharmacy’s county with a completed primary COVID-19 vaccine series and the percent of residents in a relevant CVS Pharmacy’s county who had received at least one COVID-19 booster was acquired from the CDC^12^. The percent of voters in a relevant CVS Pharmacy’s county who voted for the Republican presidential candidate in the 2020 election was obtained from the Massachusetts Institute of Technology (MIT) Election Data + Science Lab^13^. To create a measure of the number of CVS Pharmacies per square mile in a given zip code, we divided the number of CVS Pharmacy locations in that zip code (using data provided by CVS Pharmacy) by the zip code’s geographic size^11^.

# **9. Subgroup analyses**

To assess differences in the effectiveness of a typical intervention from our megastudy across different subpopulations, we estimated all intervention treatment effects by subgroup. The subgroups were defined by creating an indicator that was coded as 1 for patients who belonged to a given group (e.g., females) and 0 for those who did not (e.g., males). For continuous variables like age, we took the same approach, but subgroups were defined by a median split (i.e., “at or above median age” vs. “below median age”). No values for patient covariates used to determine subgroup assignment were missing in the deidentified data provided by CVS Pharmacy.

To estimate the treatment effects for each subgroup of interest, we estimated three different OLS regression models, each only including patients from a single subpopulation (e.g., only female patients; see Tables S4–S17). In the first model estimated for a given subgroup (always labeled Model 1), we used the same specification as our main regression model. In the second model estimated for a given subgroup (always labeled Model 2), we replaced our eight treatment indicators from Model 1 with just two treatment indicators: one indicator for whether a patient received any reminder-only intervention (interventions 1 and 3–8) and one indicator for whether a patient received our free ride intervention (intervention 2). In the third model estimated for a given subgroup (always labeled Model 3), we replaced our eight treatment indicators from Model 1 with a single pooled treatment indicator for all of our megastudy’s eight intervention conditions.

We followed this procedure to examine each subgroup of interest. Some subgroups of interest were determined by neighborhood-level data rather than individual-level data (see Tables S25–S57). Notably, some of the neighborhood data obtained for use in the subgroup analyses reported in Tables S25–S57 contained missing values (e.g., % Republican vote share in 2020 in a given county). In such cases, we dropped the patients with missing values from subgroup analyses.

# **10. Heterogeneity analyses**

Using OLS regressions, we estimated three different models for each subgroup of interest based on patient characteristics (see Tables S18–S24). In every model, we included an indicator or mean-centered continuous measure for whether the patient belonged to the subgroup of interest (e.g., an indicator for “male patient” or a continuous measure for “age (mean-centered)”)^^[[2]](#footnote-2)^^. In the first model estimated (always labeled Model 1), we included eight treatment indicators for assignment to each of our megastudy’s eight intervention conditions (the holdout control condition is the omitted indicator), and we interacted each of these treatment condition indicators with the patient subgroup measure of interest (e.g., an indicator for “male patient”). In Model 2), we included an indicator for whether a patient received any of our reminder-only interventions (interventions 1 or 3–8) and an indicator for whether a patient received our free ride intervention (intervention 2), and we interacted each of these treatment condition indicators with the patient subgroup measure of interest (e.g., an indicator for “female patient”). In the third model estimated (always labeled Model 3), we included a single pooled treatment indicator for all of our megastudy’s eight intervention conditions, and we interacted this treatment condition indicator with the patient subgroup measure of interest (e.g., an indicator for “male patient”).

We followed this procedure to examine each subgroup of interest. Some subgroups of interest were determined by neighborhood-level data rather than individual-level data (see Tables S25–S57). As noted previously, some of the neighborhood-level data contained missing values (e.g., % Republican vote share in 2020 in a given county). In such cases, we replaced the missing value in our regression with the mean value of the variable among other patients in the same study condition, and we included a dummy variable in the regression of interest that took on a value of 1 whenever this value was missing. We also added an interaction between that missing variable dummy and all treatment indicators.

# **11. Robustness checks of analysis of experts’ and laypeople’s forecasts of our interventions’ effects**

As noted in the main manuscript, at the time of our lay and expert forecasting studies, we had not yet determined that data on patients’ vaccinations prior to November 3, 2022 was unstable (due to updating over time), so when calculating summary statistics to share with participants, we excluded data from patients who appeared to have received a vaccine before the November 3, 2022 launch of our experiment. We thus told survey participants that 5.31% of participants in our control condition had been vaccinated, but our current analysis reports that 5.09% of participants in our control condition were vaccinated (because we do not exclude any participants from our analysis who were randomly assigned to conditions, we include thousands of additional people in our study sample who did not get a November vaccine).

Our main manuscript compares the absolute predicted increase in vaccinations with regression-estimated increases in vaccinations across interventions (e.g., if a forecaster predicted a treatment would produce a 6.31% vaccination rate, we would compare that to the baseline vaccination rate provided to them of 5.31% and call their prediction a 1.00 percentage point increase). As a robustness check, we reran our analyses looking at forecasted relative percentage increases in vaccinations across interventions (e.g., if a forecaster predicted a treatment would produce a 6.31% vaccination rate, we would compare that to the baseline vaccination rate provided to them of 5.31% and call their prediction an 18.83% relative percentage increase). The results of our robustness checks support all of the analyses reported in our main manuscript. Specifically, using this alternative analysis strategy, we found that lay forecasters again predicted that the free ride intervention would significantly outperform all other interventions tested (Wilcoxon signed-rank tests, all p’s < 0.05). Lay forecasters were also dramatically too optimistic about the effects of both free rides and reminders relative to their actual, measured effects (their estimates were 6 to 20x too high for every single intervention). Further, the correlation between lay forecasters’ eight (median) forecasts and the actual regression-estimated % change in vaccinations caused by each intervention was 0.00. Turning to expert forecasts using this alternative analysis strategy, we find that experts predicted the free ride intervention would significantly outperform all other reminder-only interventions tested (Wilcoxon signed-rank test, all p’s < 0.01). Again, experts were overly optimistic about the effects of reminders relative to their actual, measured effects (their estimates were 2 to 7x too high for every single intervention), but they were significantly less optimistic about every intervention’s performance than lay forecasters (all p’s from Wilcoxon rank sum tests < 0.001). Further, the correlation between experts’ eight (median) forecasts and the actual regression-estimated % change in vaccinations caused by each intervention was 0.20.

As a further robustness test, we looked at the subset of forecasters who made relatively accurate estimates of our baseline intervention’s impact (among experts and non-experts, separately). We defined those who estimated that our baseline reminder would increase vaccination rates by 1 percentage point (which was the observed effect of this reminder) plus or minus 1 percentage point as “superior” forecasters. We then explored whether these “superior” forecasters were still miscalibrated regarding: (1) our eight interventions’ relative benefits and (2) the absolute impact of our free ride intervention. There were N=71 expert forecasters (out of 163) who met the inclusion criteria described above for “superior forecasters” and there were N=25 non-expert forecasters (out of 199) who met these inclusion criteria.

First, we focus on these superior forecasters’ efforts to estimate our interventions’ relative benefits. The correlation between the “superior” subset of expert forecasters’ estimates of intervention effects and actual intervention effects is 0.23 (the correlation between these quantities is 0.20 in the full expert sample). The correlation between the “superior” subset of lay forecasters’ estimates of intervention effects and actual intervention effects is -0.10 (the correlation between these quantities is 0.00 in the full layperson sample).

Second, we examine these superior forecasters’ efforts to estimate the absolute impact of our free ride intervention. Both the “superior” expert and lay forecasters expect free rides to have the largest effect of all interventions. Further, both groups vastly overestimate the impact of free rides (the median predicted increase in vaccinations from free rides was 3.31 percentage points among “superior” expert forecasters, and it was 5.19 percentage points among “superior” lay forecasters). Figures S3 and S4 plot superior forecasters’ average estimates of all intervention effects and show that superior forecasters remain remarkably miscalibrated regarding: (1) our eight interventions’ relative benefits and (2) the absolute impact of our free ride intervention.

Finally, we took a look at the average rankings of each intervention according to lay forecasters and expert forecasters as well as the fraction of each forecasting group that put the free ride intervention at the top of their list. These measures should be less susceptible to noise. As shown in Extended Data Tables 7 and 8, no matter how we cut our data, we see strong expectations from forecasters that free rides will outperform other interventions.

#

# **12. Forecasting study stimuli**

#

Screen 1: Welcome page and consent form

**
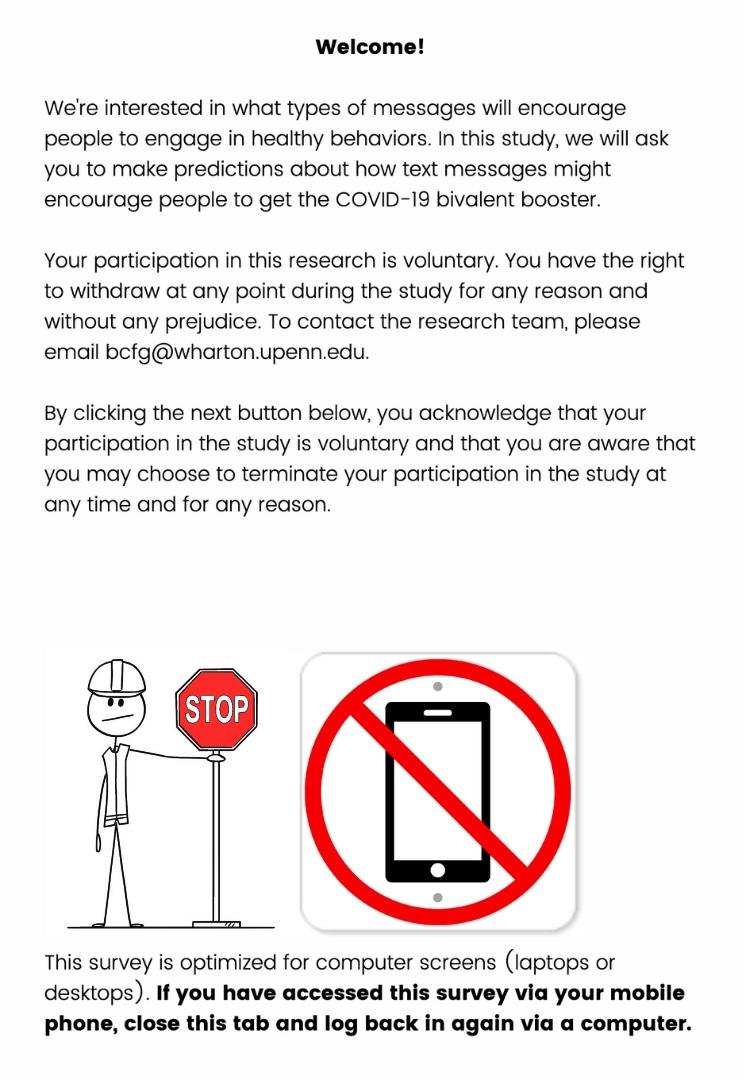
**

Screen 2: Screening question displayed to expert sample


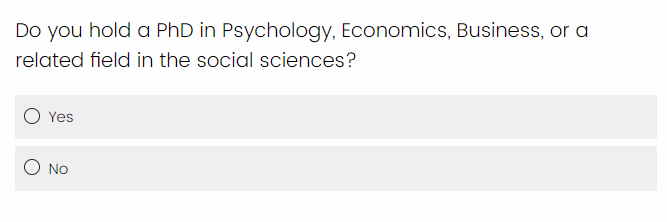


Screen 3: Task overview

**
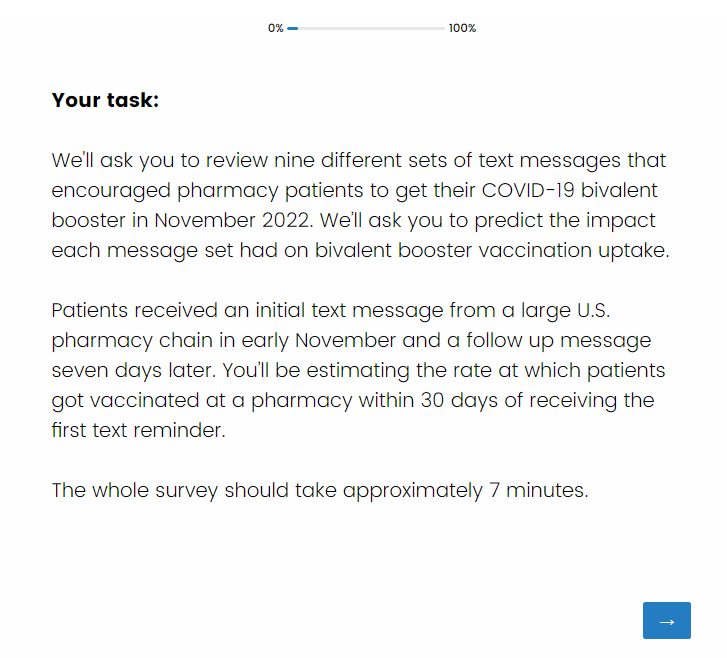

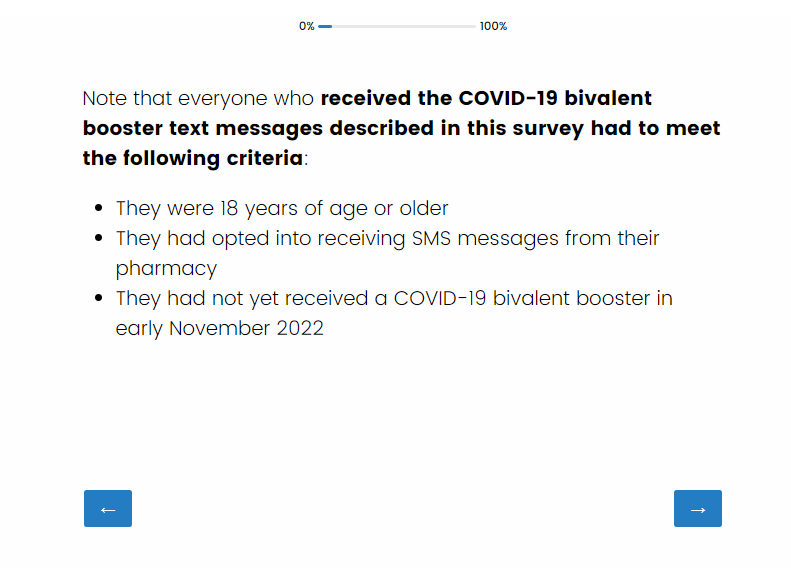
**

**
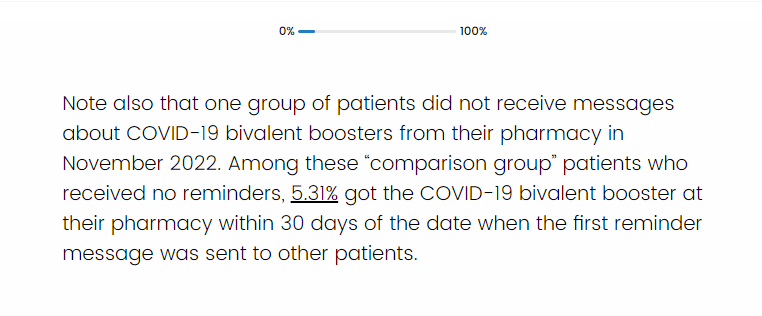
**

Screen 4: Attention checks


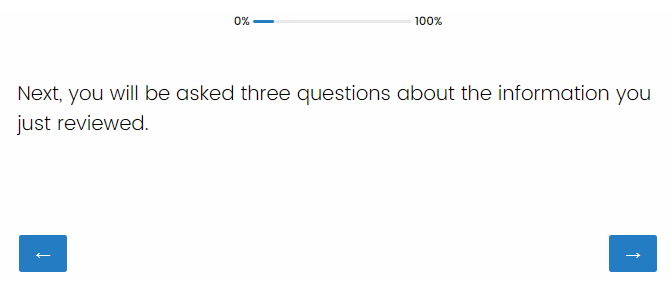


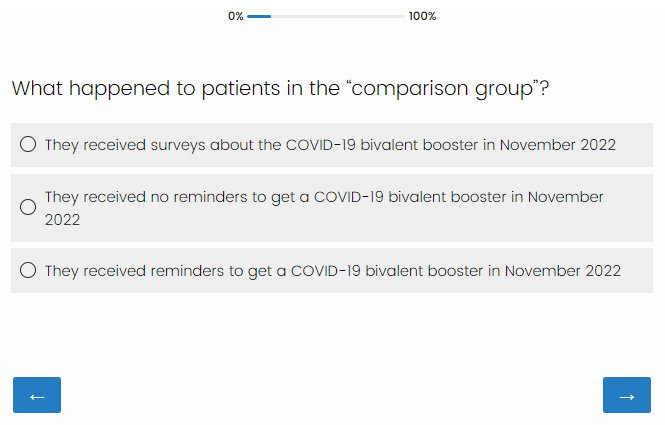


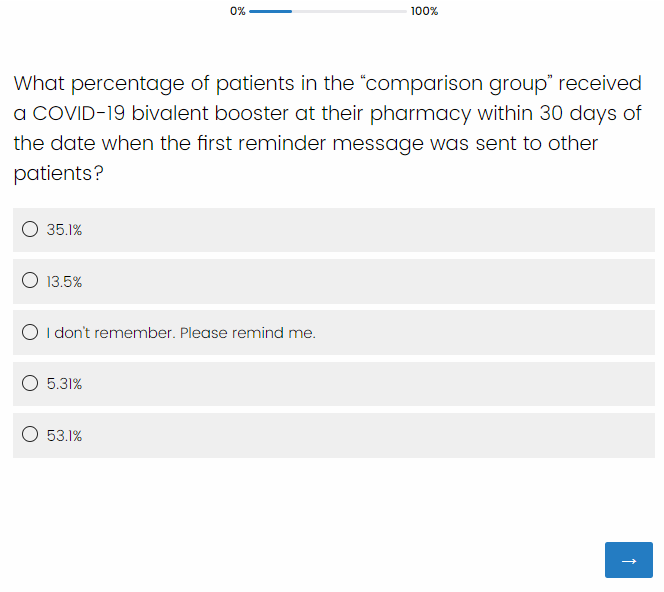


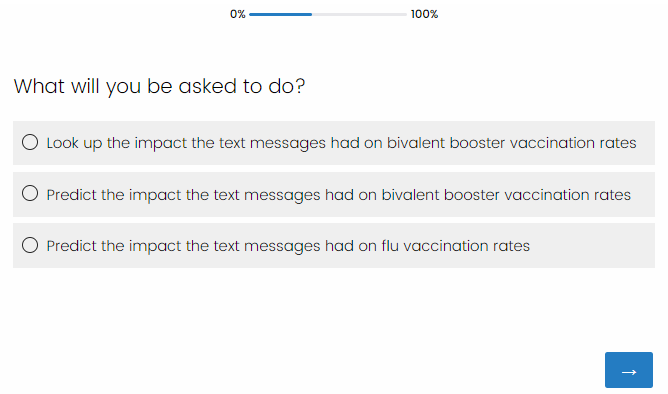


Screen 5: Transition to forecasting task


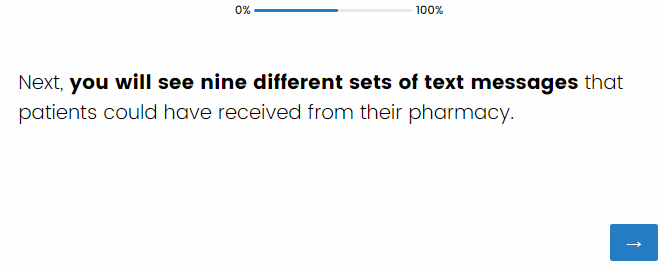


Screens 6–14: Baseline message forecasting task

*(Note that the order in which different intervention conditions were displayed was randomized. Patient names from CVS Pharmacy in the sample stimuli below are fictitious and only provided for illustrative purposes, as are pharmacy addresses and scheduler links.)*


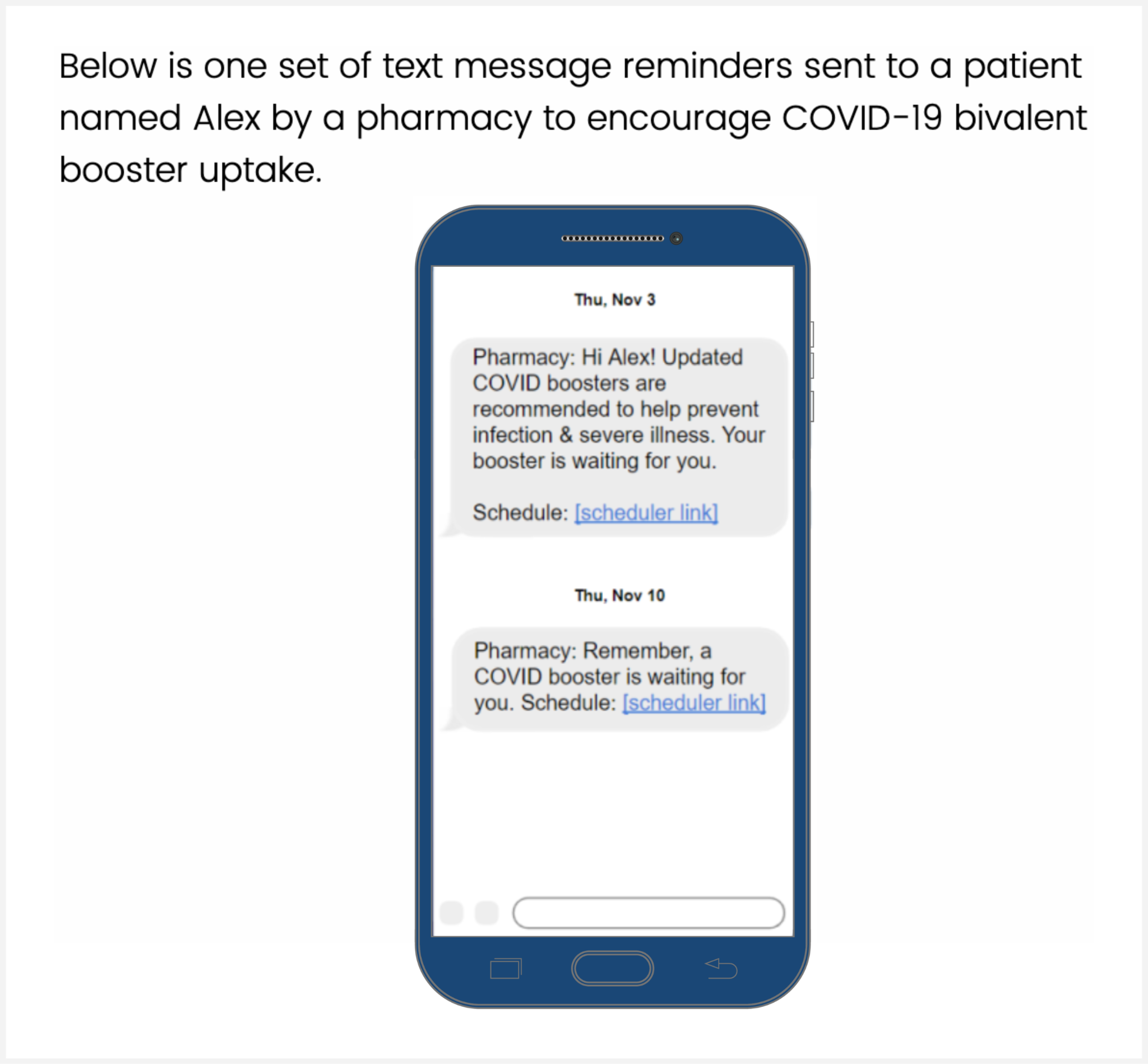


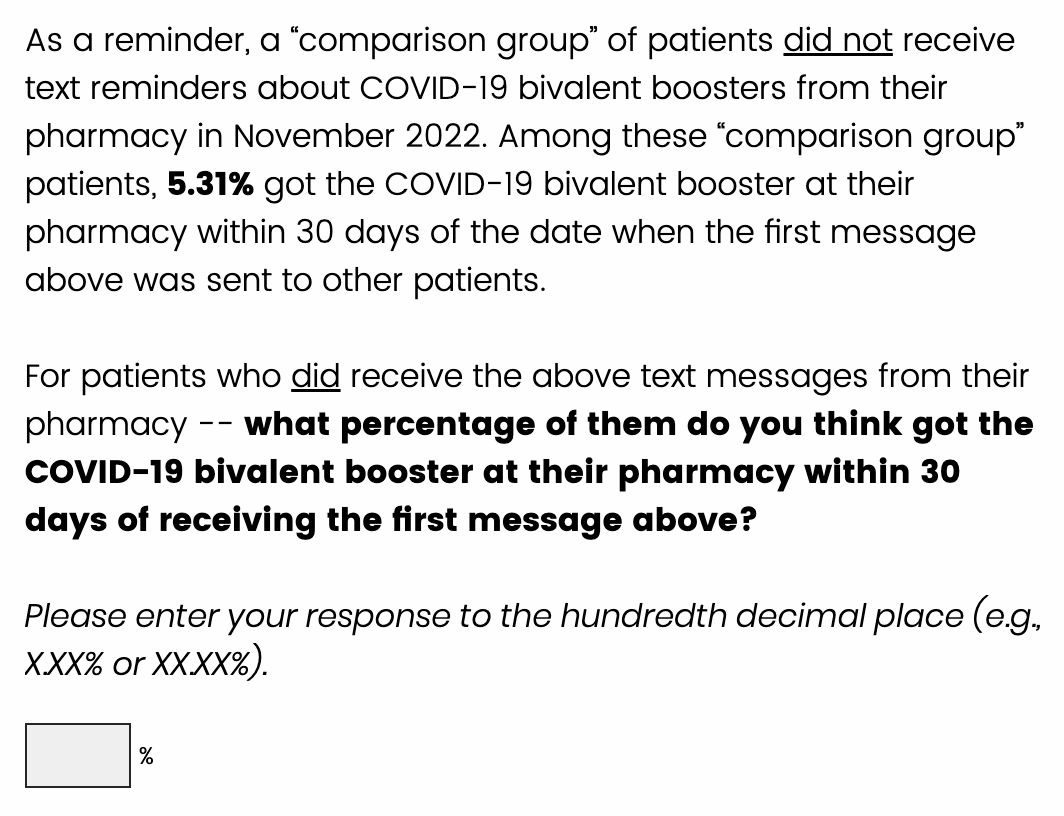


Free ride forecasting task^^[[3]](#footnote-3)^^


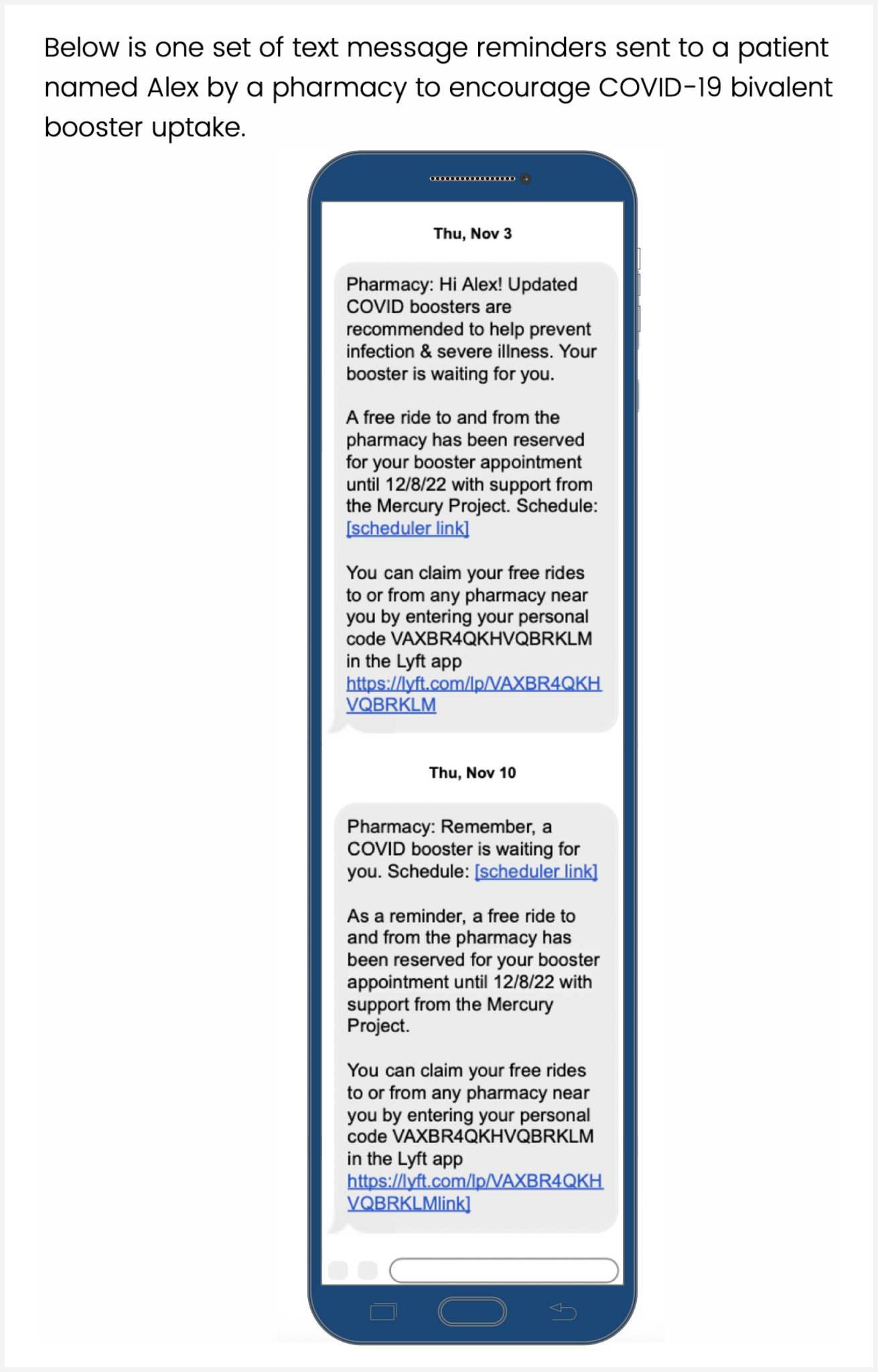


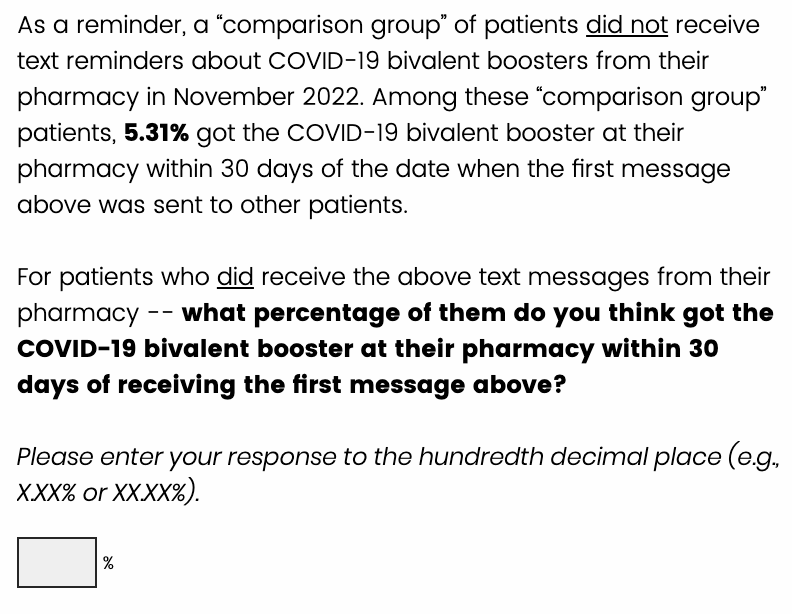


Suggested plan forecasting task^^[[4]](#footnote-4)^^


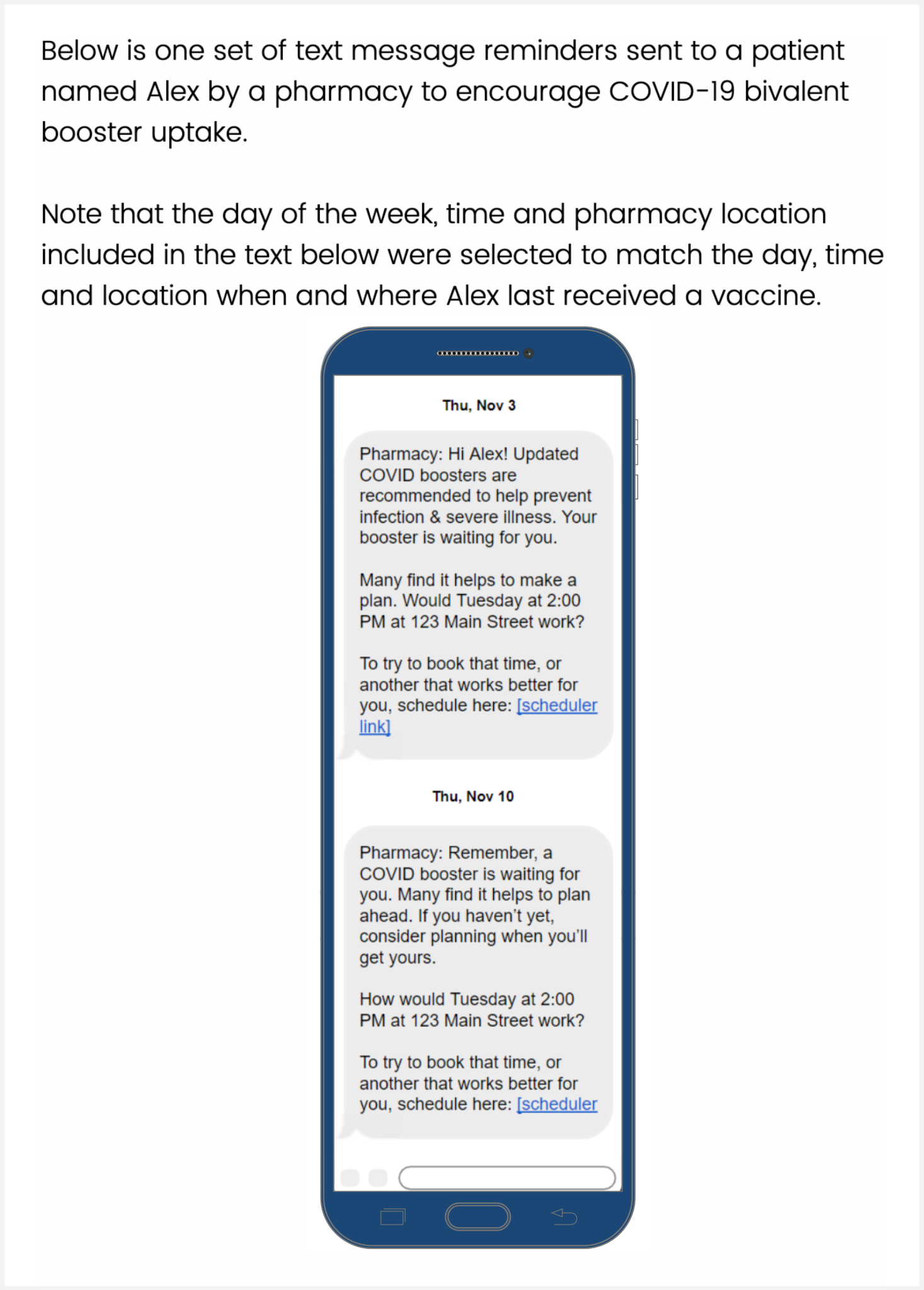


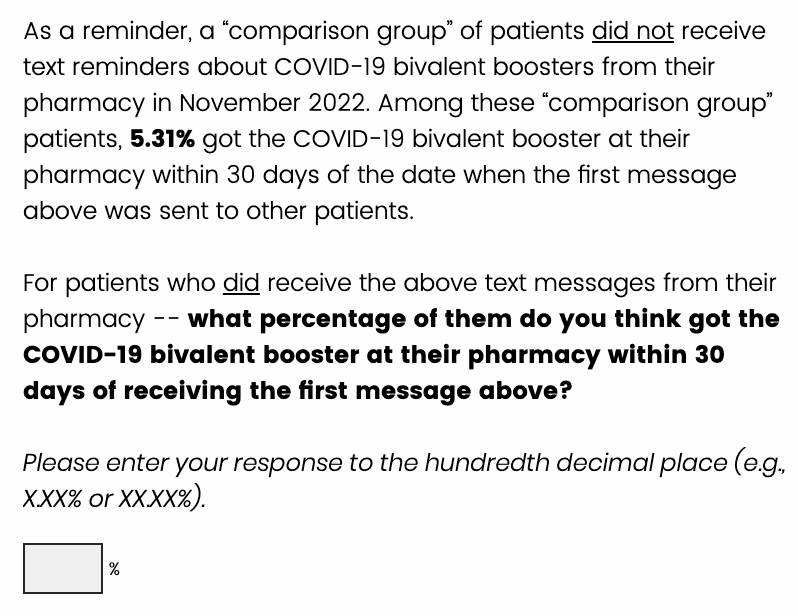


Infection rates forecasting task (Top 50%)^^[[5]](#footnote-5)^^


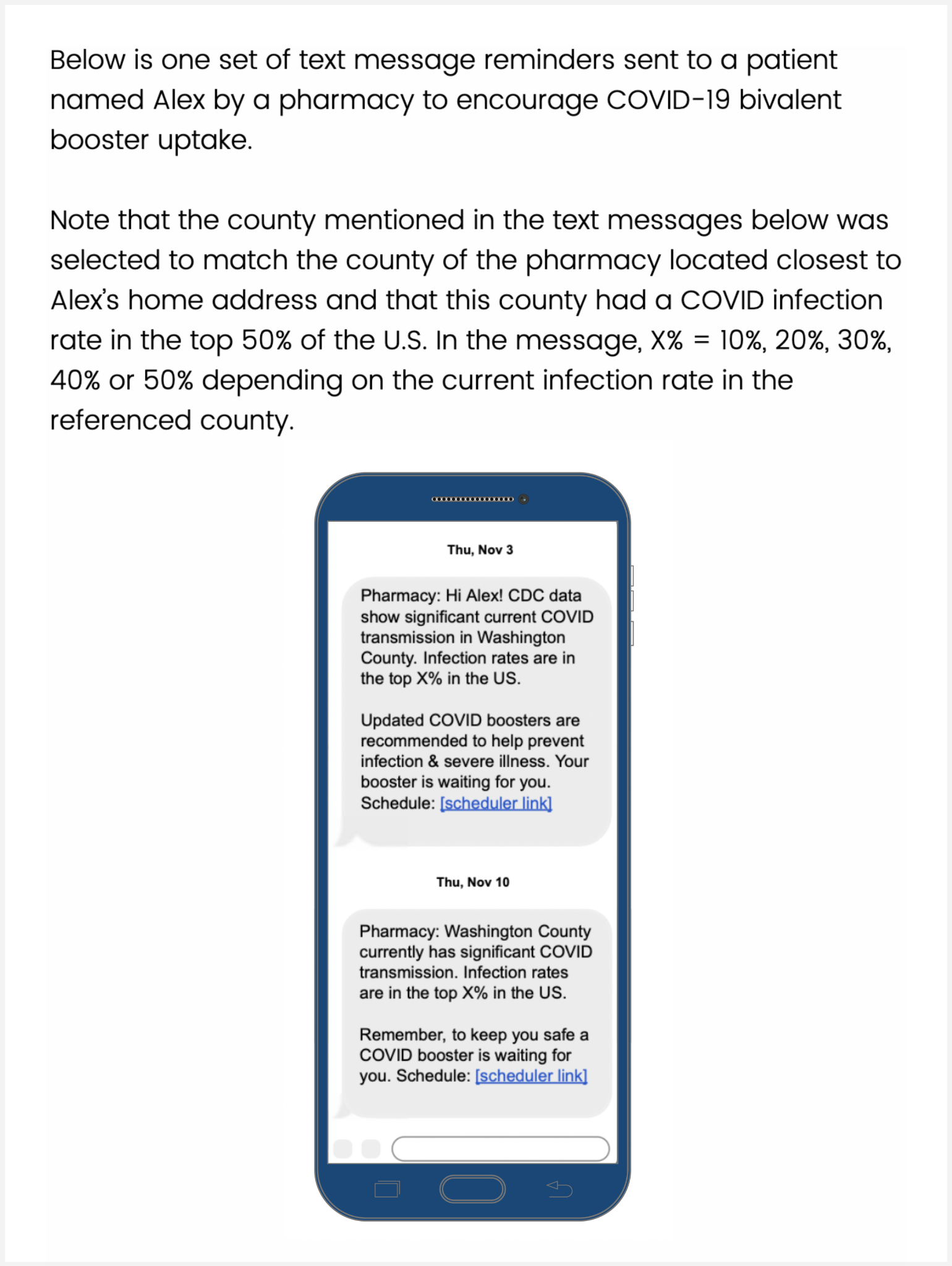


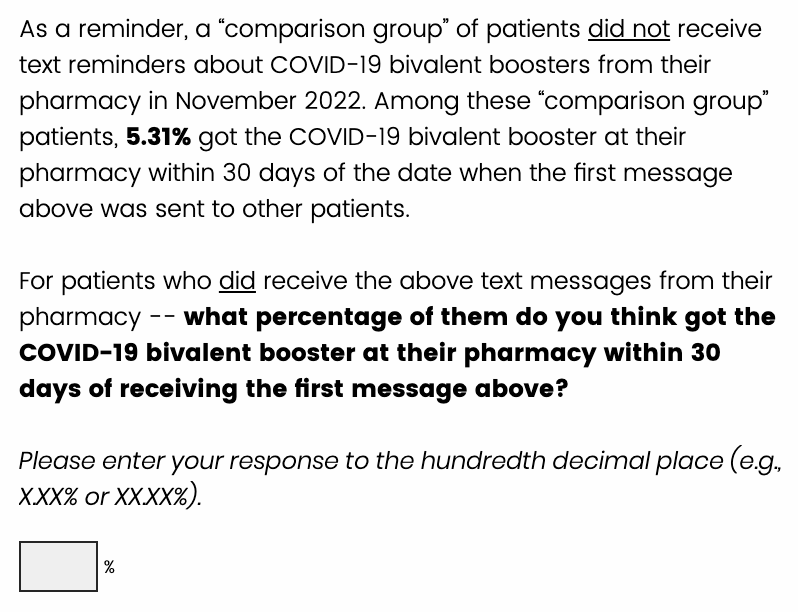


Infection rates forecasting task (Bottom 50%)^^[[6]](#footnote-6)^^


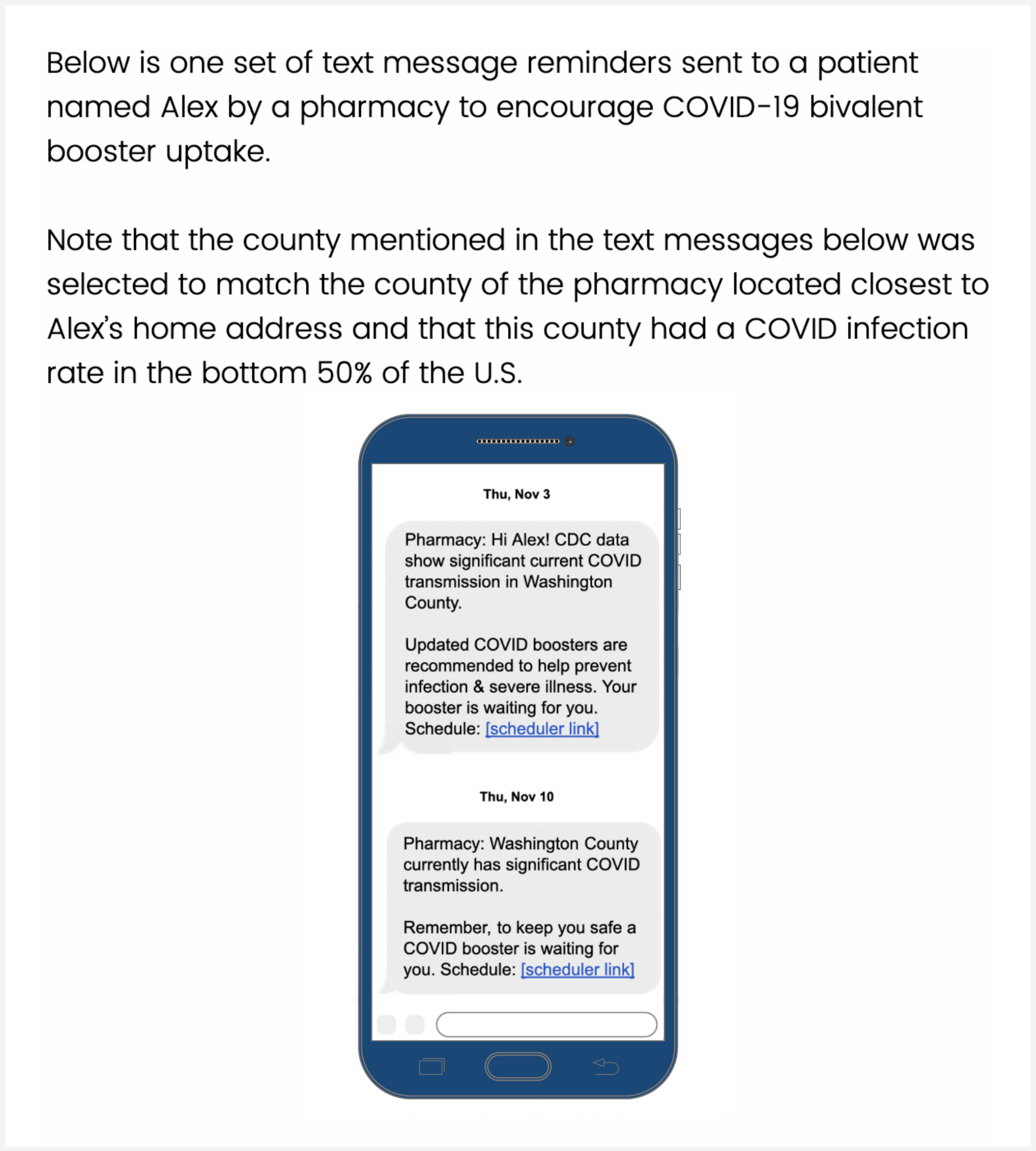


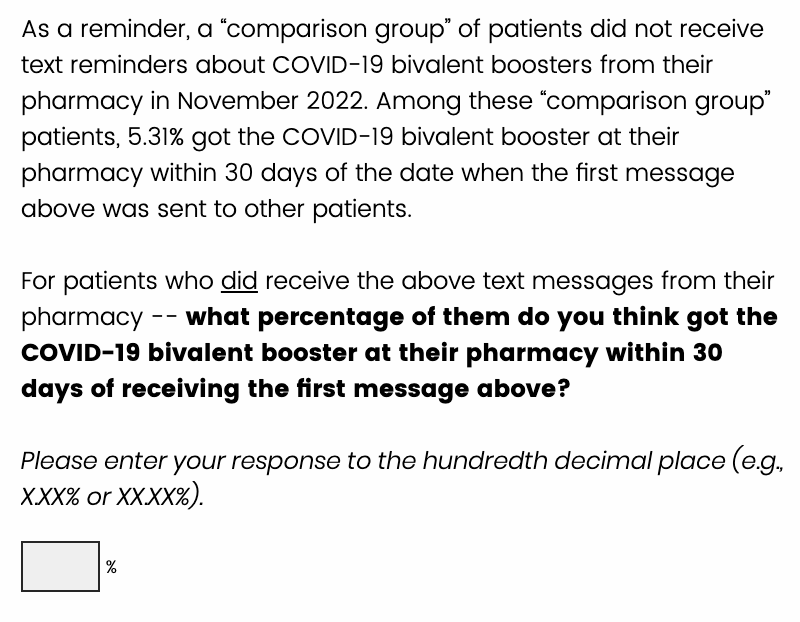


Pharmacy message forecasting task^^[[7]](#footnote-7)^^


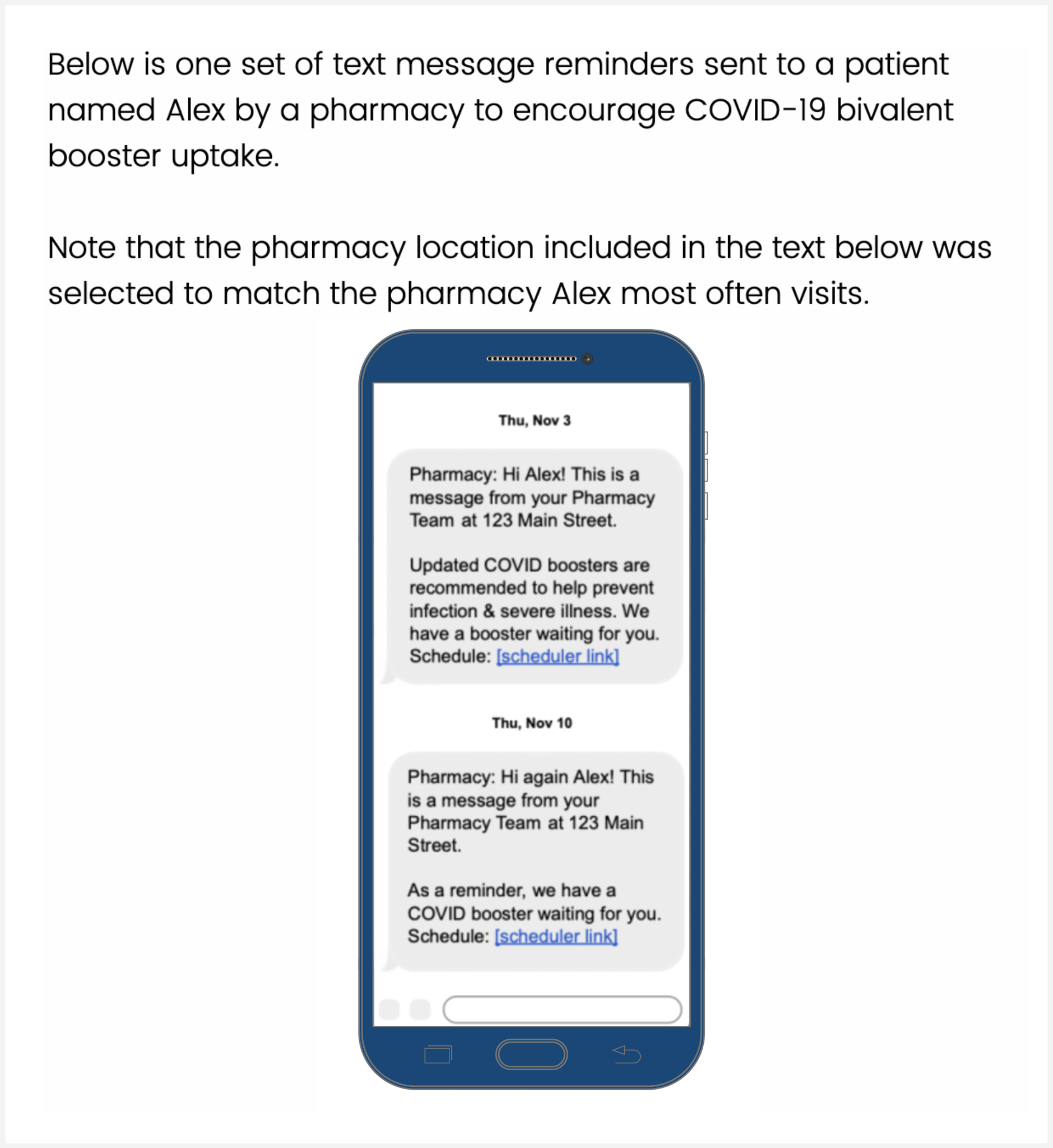


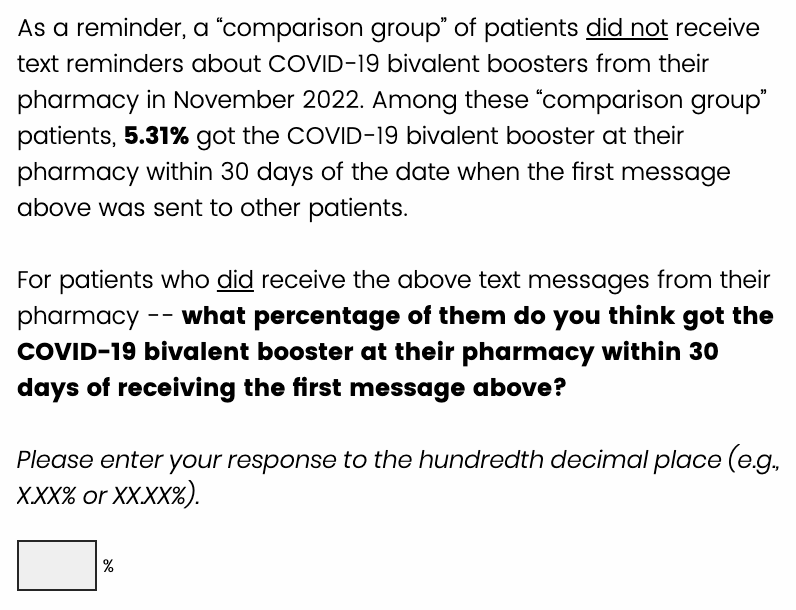


CDC recommended forecasting task^^[[8]](#footnote-8)^^


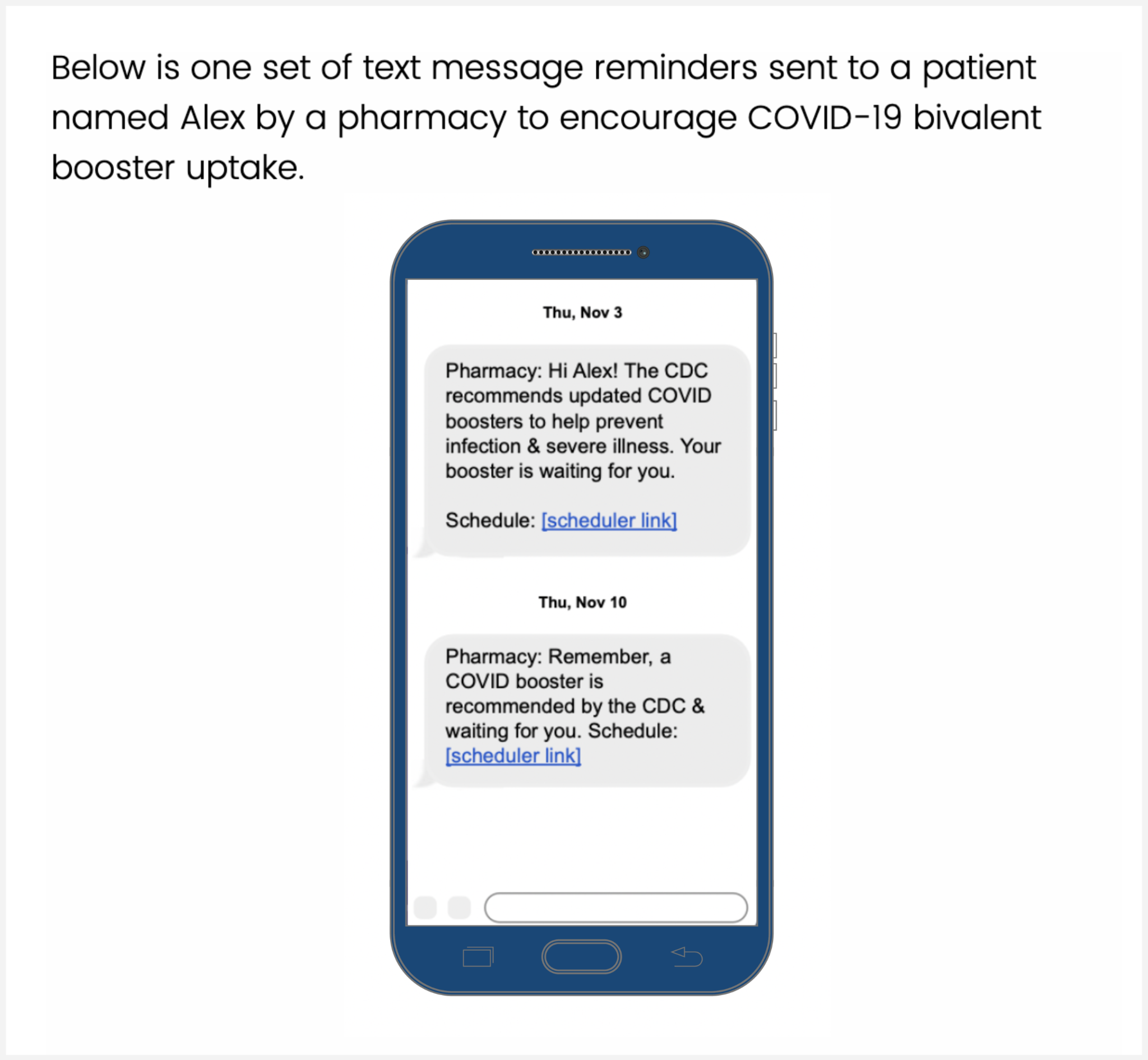


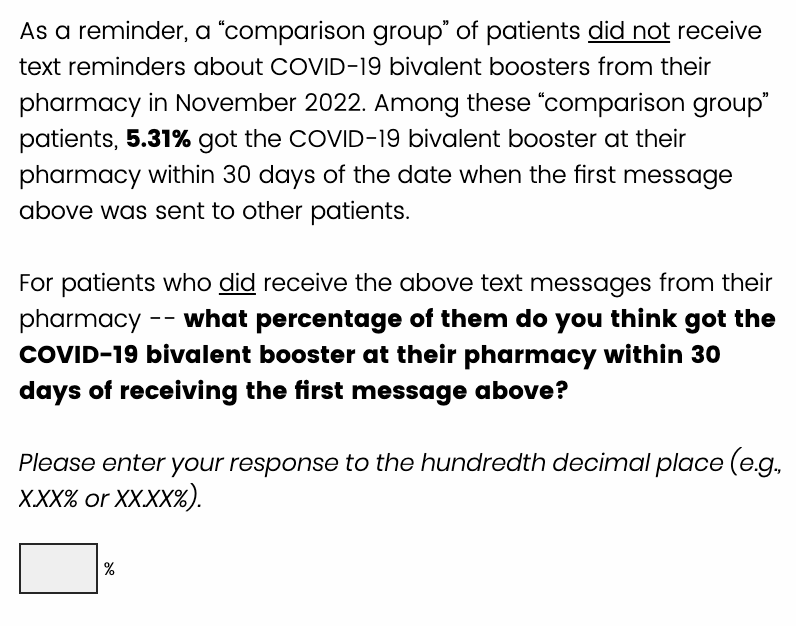


Holiday protection forecasting task^^[[9]](#footnote-9)^^


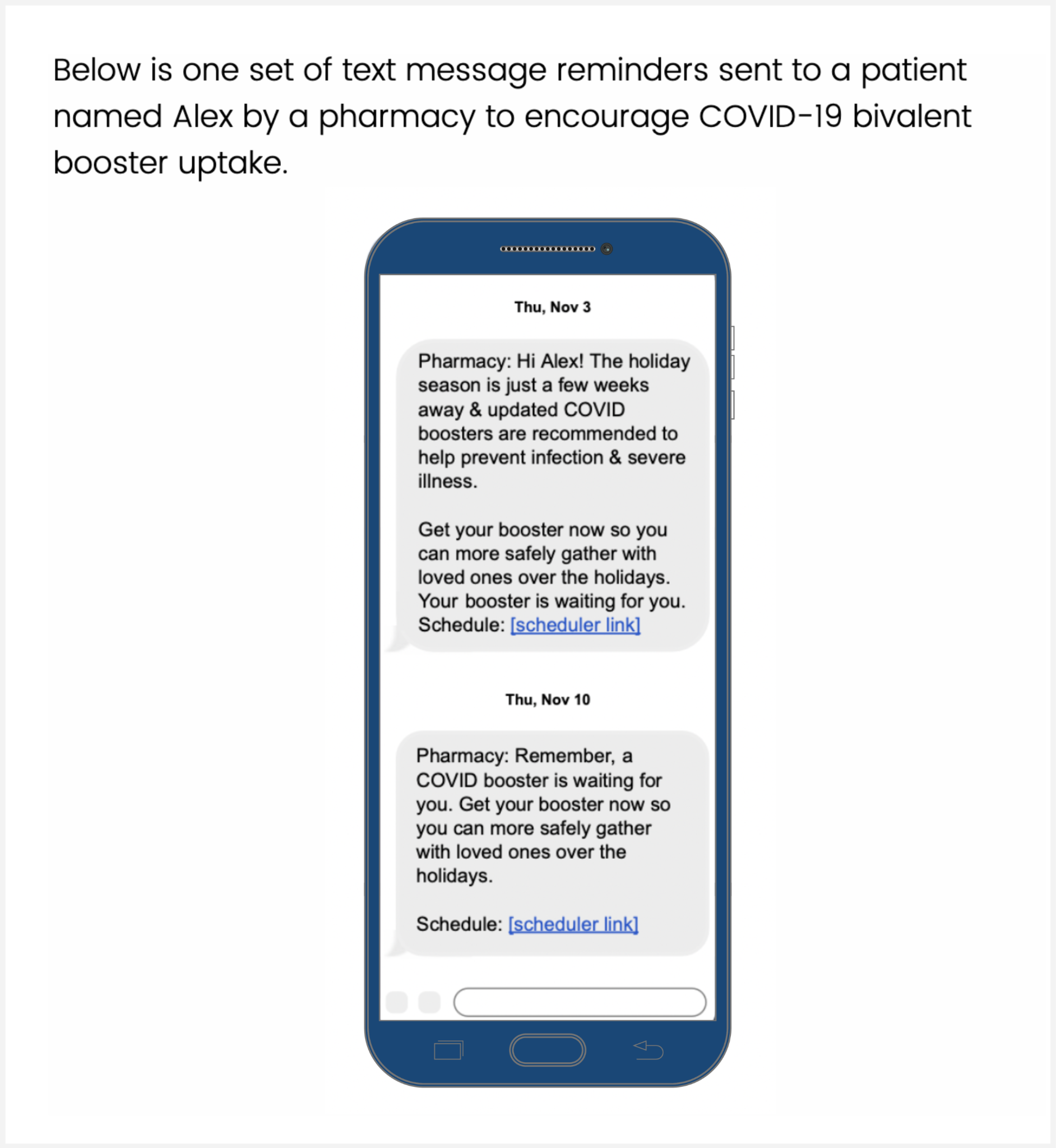


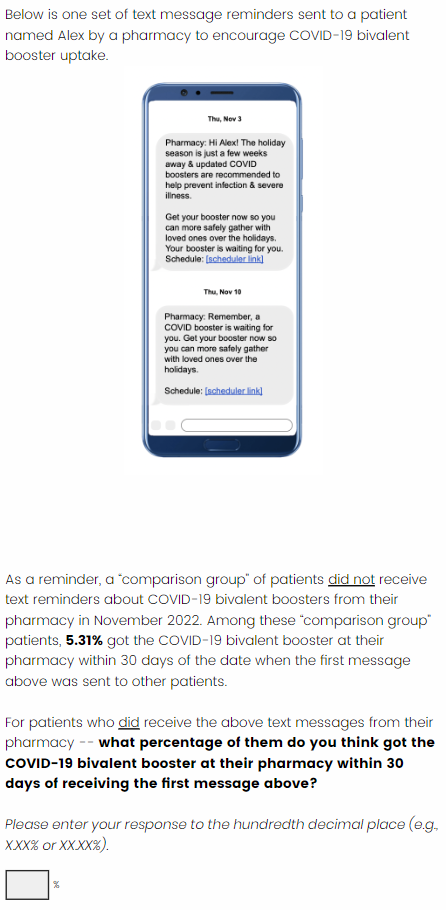


Misinformation resources forecasting task^^[[10]](#footnote-10)^^

# **
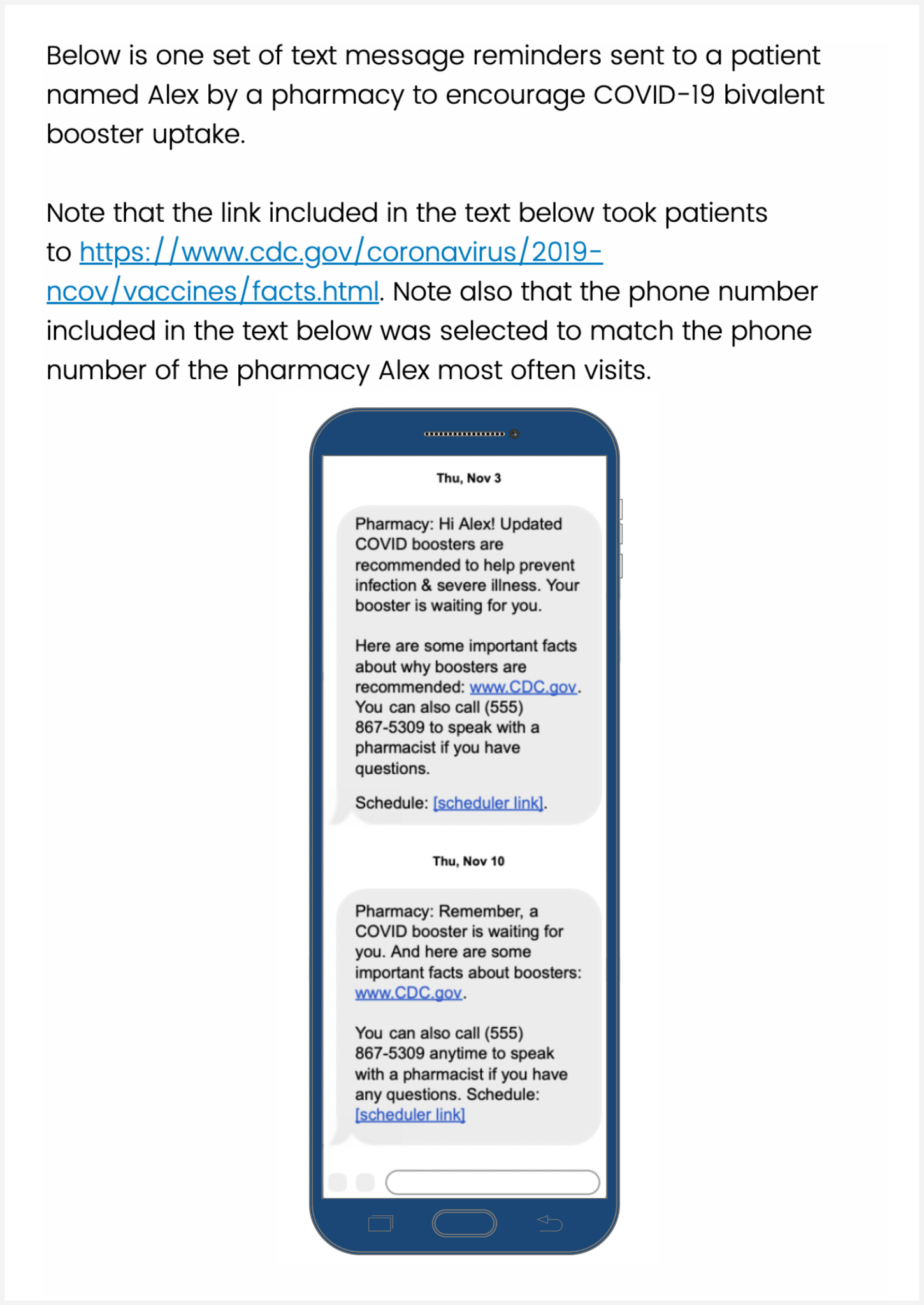
**

# **
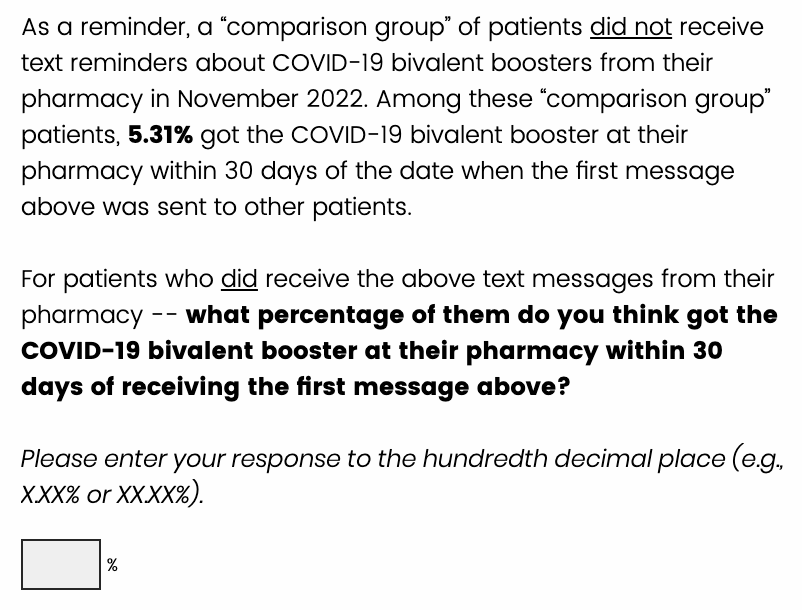
**

# **13. Figures**

**Figure S1. Regression-estimated percent of patients who received a bivalent COVID-19 booster at a CVS Pharmacy within 30 days of this megastudy’s launch, by condition (excluding data from launch day 1).** Error bars represent 95% confidence intervals.

*Note:* The points in this figure present the regression-estimated percent of patients who received a bivalent COVID-19 booster at a CVS Pharmacy by experimental condition. These estimates are derived from a variant of our supplemental regression model that excludes data from launch day 1 (see Extended Data Table 2, Model 1) in which we include an additional binary indicator for assignment to the holdout control condition and exclude the intercept. The number of patients in each megastudy condition depicted here are as follows: holdout control (328,119), baseline message (329,179), free ride (33,206), default plan (328,226), infection rates (328,112), pharmacy team message (328,979), CDC recommended (328,392), holiday protection (328,285), misinformation resources (328,826). Whiskers depict 95% confidence intervals.

*
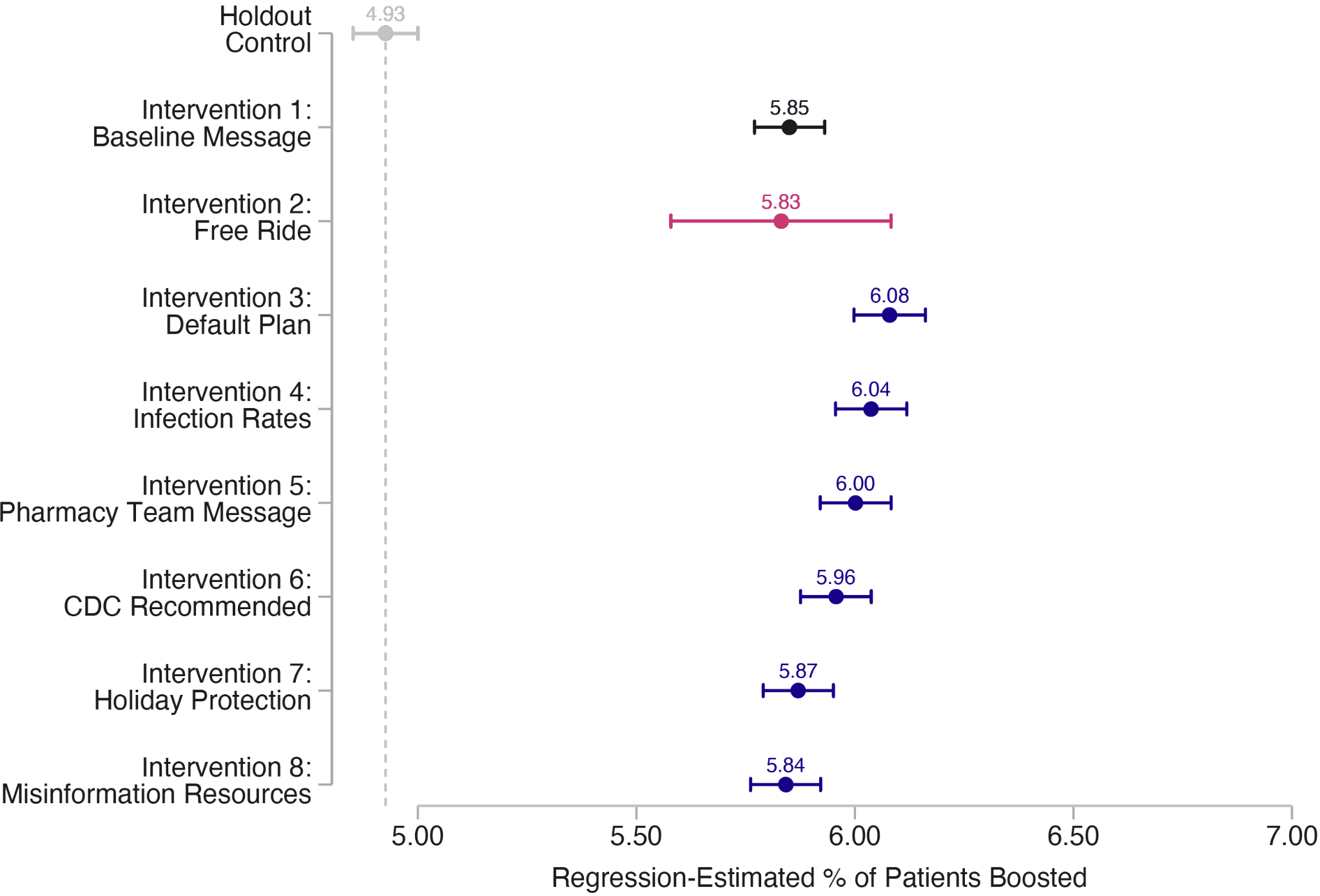
*

**Figure S2. Histogram showing the distribution of distances to the nearest CVS Pharmacy for “synthetic” patients living at randomly selected addresses in each zip code associated with real patients in our study**

**
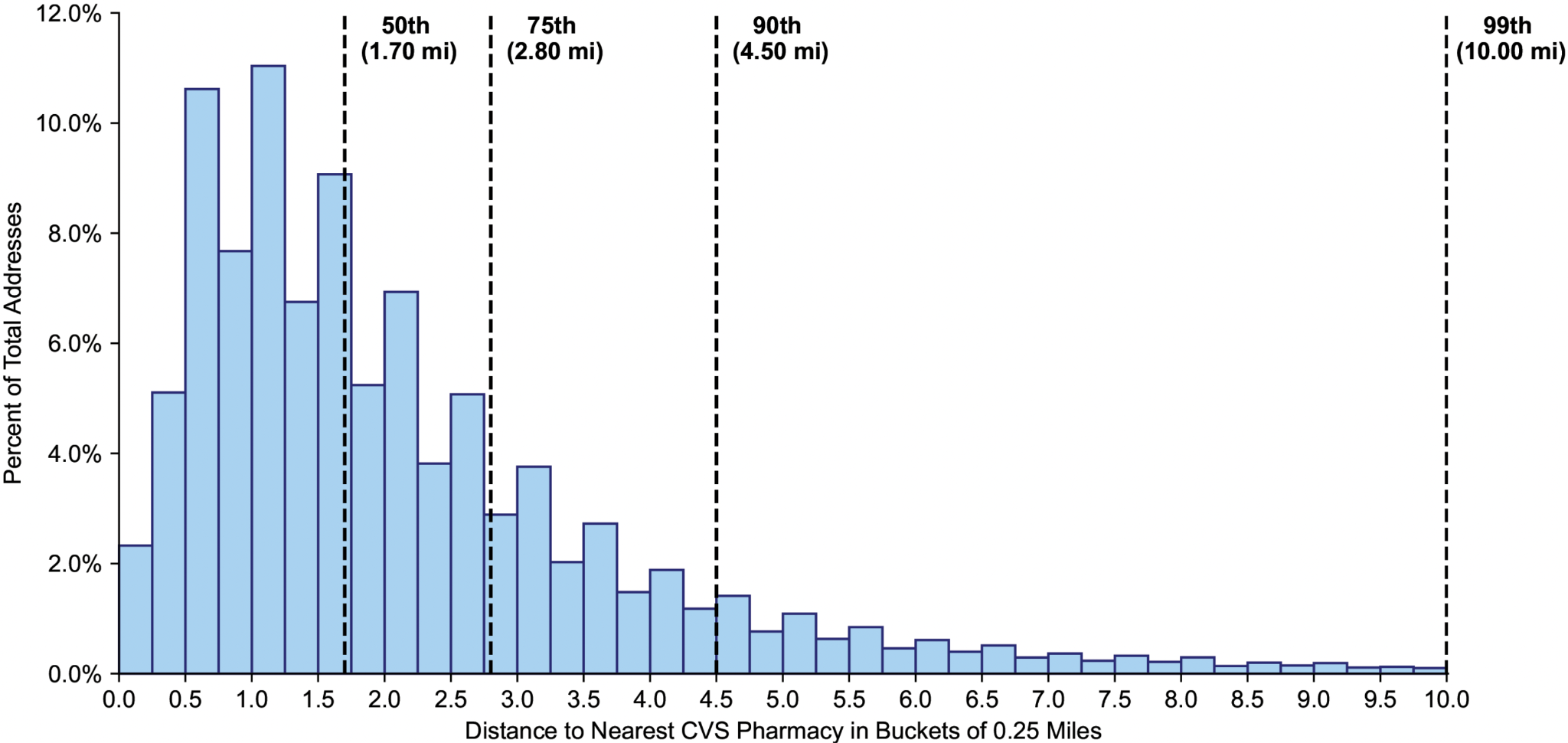
**

*Note.* This figure represents the distribution of distance to the nearest CVS Pharmacy for addresses from zip codes in our study. The bin width in this graph is 0.25 miles. The dotted lines represent 50th (median), 75th, 90th, and 99th percentile distances to a CVS Pharmacy at the zip code level.

**Figure S3. Scatterplot showing experts’ median *forecasts* of intervention effects on the x-axis and *actual* regression-estimated intervention effects on the y-axis, including only “superior” forecasters.
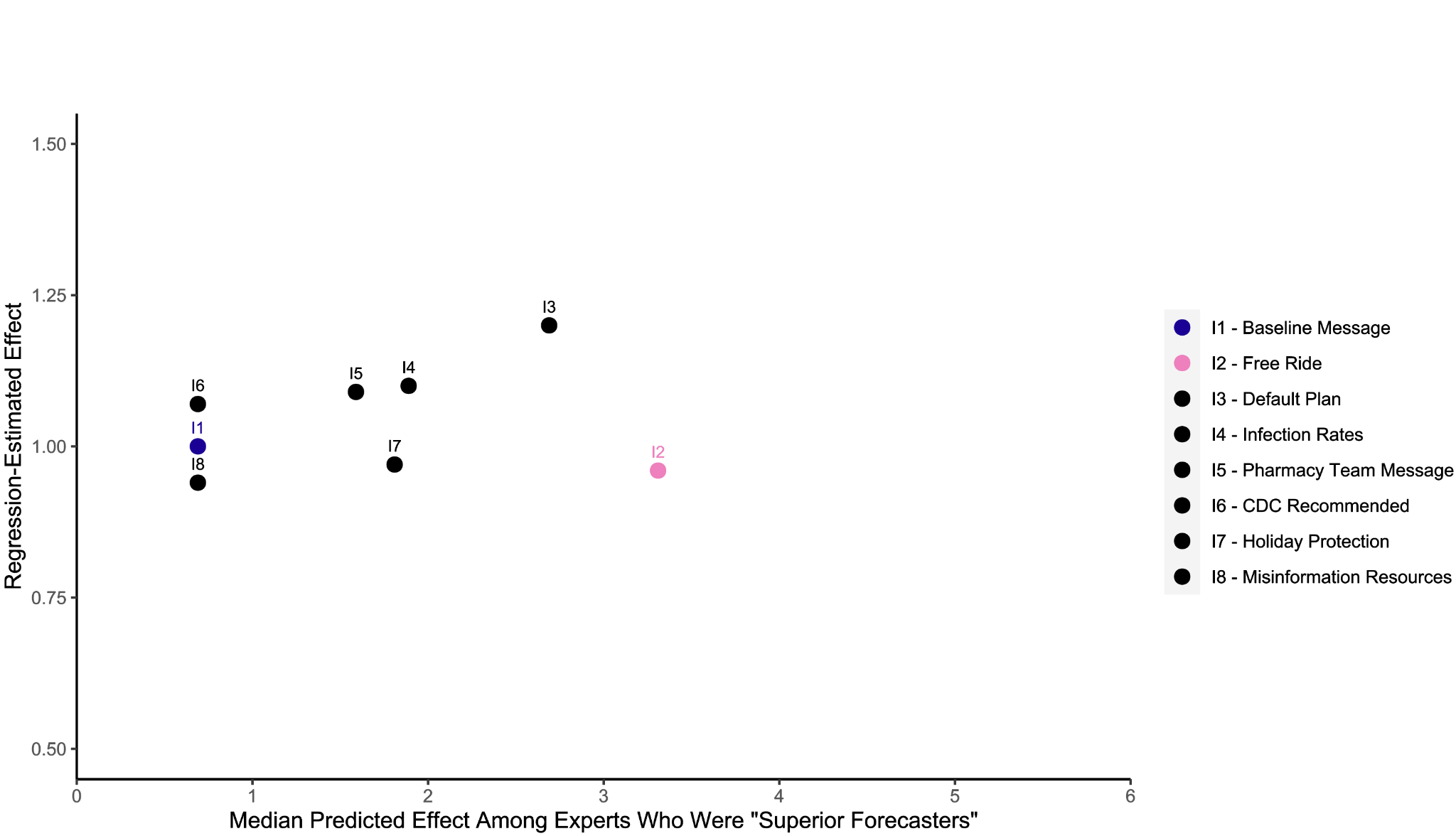
**

*Note:* “Superior” expert forecasters include only those experts who estimated that our baseline reminder would increase vaccination rates by 1 percentage point plus or minus 1 percentage point (N=71 out of our original 163 expert forecasters).

**Figure S4. Scatterplot showing laypeople’s median *forecasts* of intervention effects on the x-axis and *actual* regression-estimated intervention effects on the y-axis, including only “superior” forecasters**

**
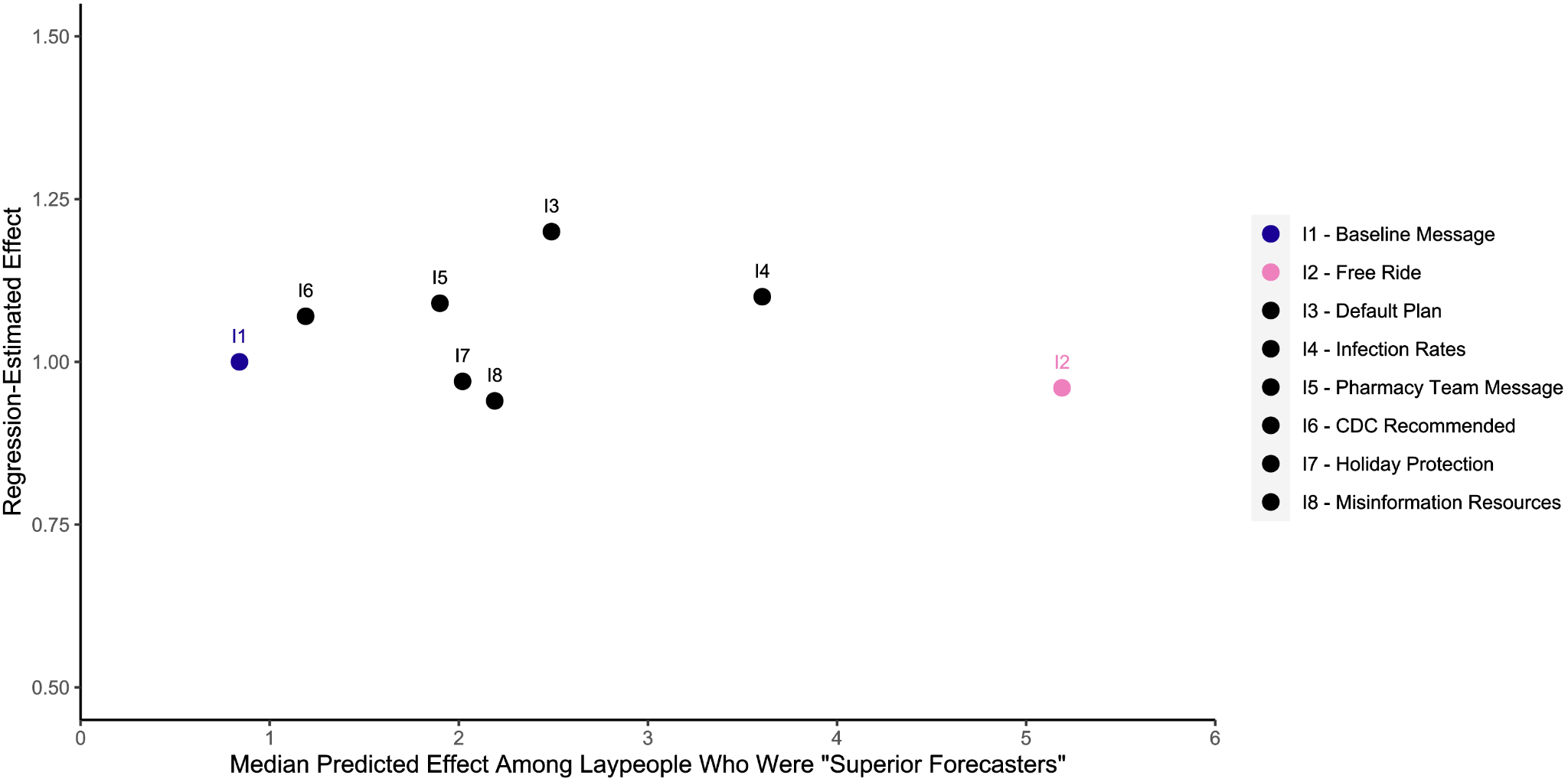
**

*Note:* “Superior” lay forecasters include only those laypeople who estimated that our baseline reminder would increase vaccination rates by 1 percentage point plus or minus 1 percentage point (N=25 out of our original 199 lay forecasters).

**Figure S5. eCDF of pairwise differences between forecasted effects of the Free Ride condition and each Reminder-Only condition, by forecaster type (expert vs. lay forecaster).** The following panels present empirical Cumulative Distribution Functions (eCDF) of the differences between the predicted effects of the Free Ride intervention and each other intervention, according to lay and expert forecasters (Baseline Message in **A**, Default Plan in **B**, Infection Rates in **C**, Pharmacy Team Message in **D**, CDC Recommended in **E**, Holiday Protection in **F**, Misinformation Resources in **G**).

**
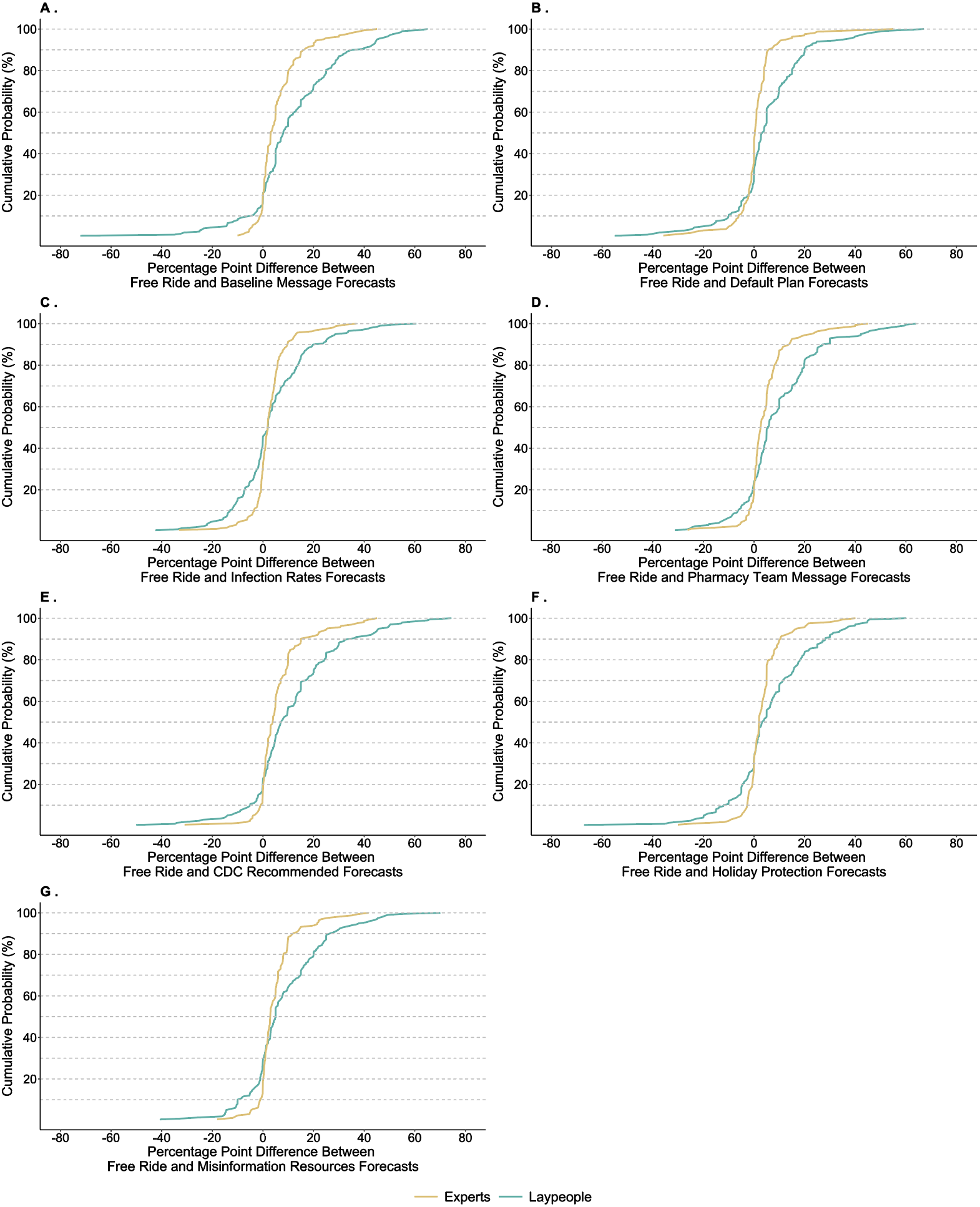
**

# **14. Tables**

**Table S1.** Balance tests of patient-level variables by condition assignment.


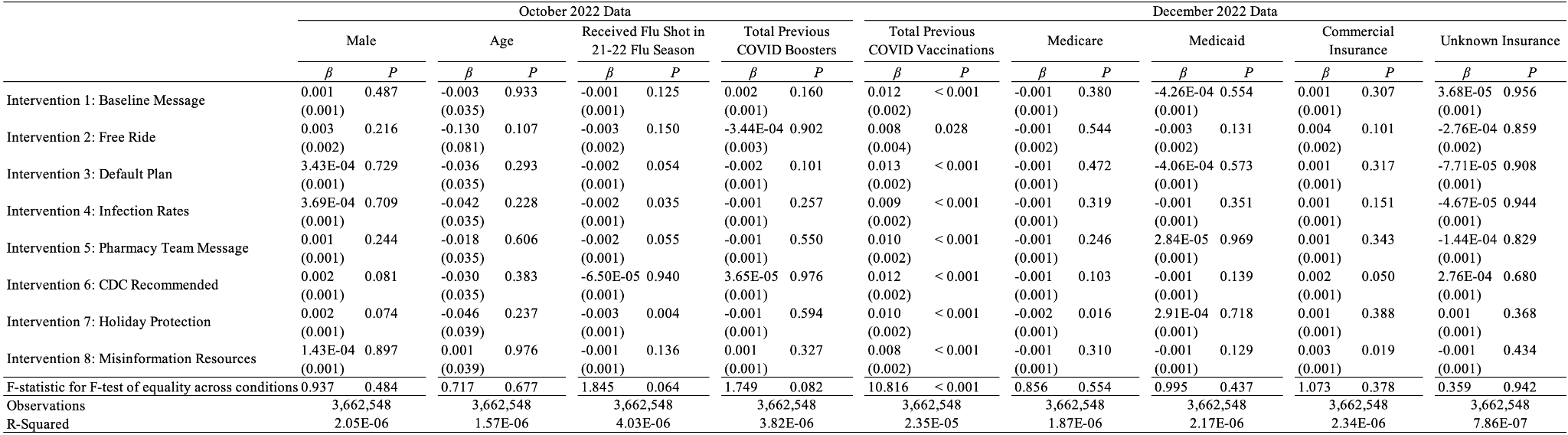


*Note.* This table reports the results of nine ordinary least squares (OLS) regressions that test whether patient-level variables are balanced across conditions. The predictor variables in each of these regressions are eight indicators for assignment to each of our megastudy’s eight intervention conditions. All regression coefficients and standard errors have been multiplied by 100 to improve interpretability. Statistical tests of whether an individual regression coefficient is zero are all two-sided. Statistical tests involving multiple regression coefficients are all undirected.

**Table S2. Balance tests of patient-level variables by condition assignment and launch day pairs.**

**
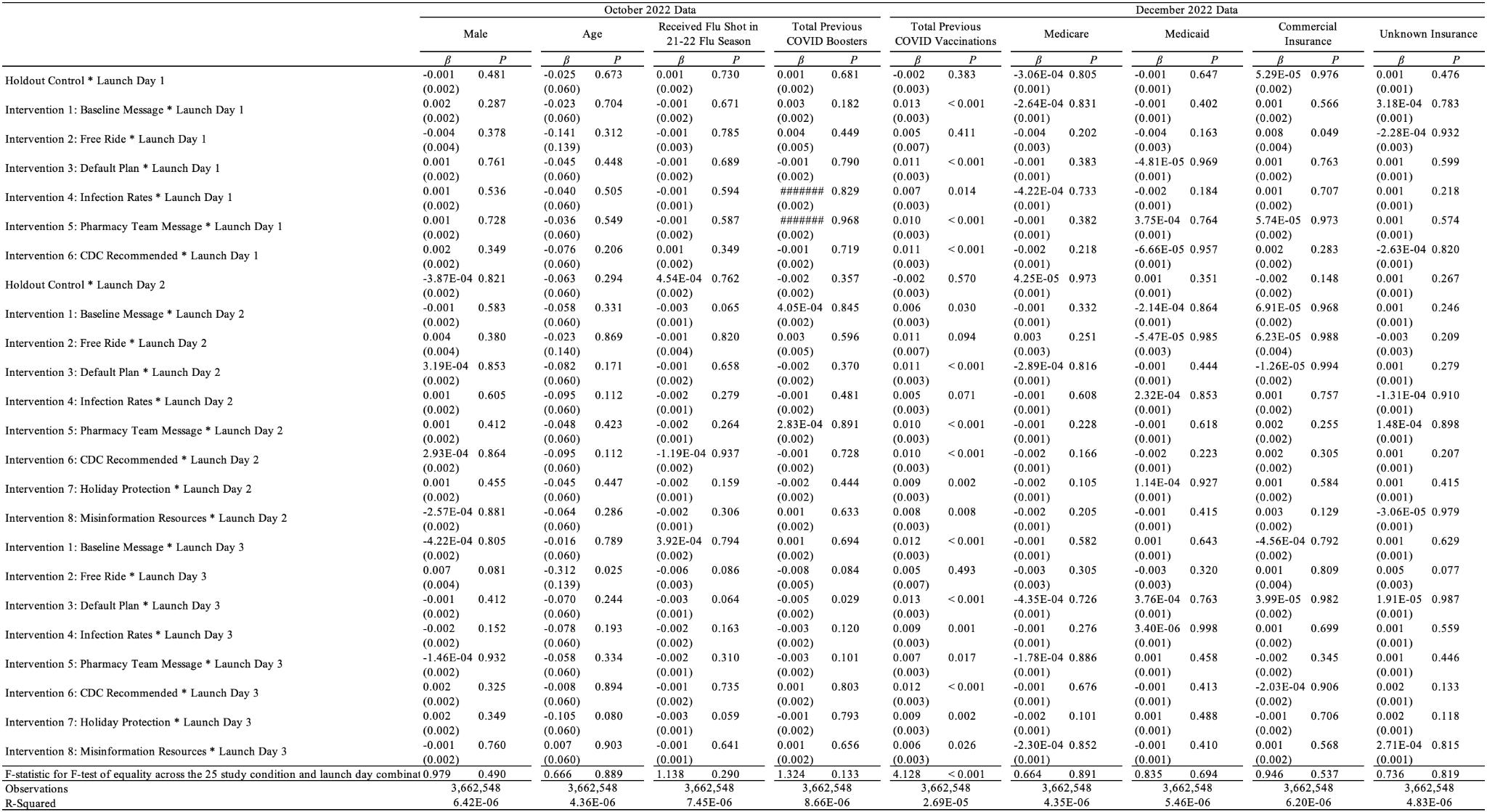
**

*Note.* This table reports the results of nine ordinary least squares (OLS) regressions that test whether patient-level variables are balanced across conditions and launch day combinations. The predictor variables in each of these regressions are indicators for assignment to each of our megastudy’s 25 intervention condition launch day pairs (Holdout Control, Launch Day 3 omitted). All regression coefficients and standard errors have been multiplied by 100 to improve interpretability. Statistical tests of whether an individual regression coefficient is zero are all two-sided. Statistical tests involving multiple regression coefficients are all undirected.

# **Table S3. Wald tests comparing the regression-estimated impacts of each of our megastudy’s intervention conditions to the regression-estimated impact of our megastudy’s baseline message (Intervention 1: Baseline Message) as well as Wald tests comparing the regression-estimated impact of our megastudy’s intervention with the smallest regression-estimated impact (Intervention 8: Misinformation Resources) to the regression-estimated impacts of each of our megastudy’s other intervention conditions.**


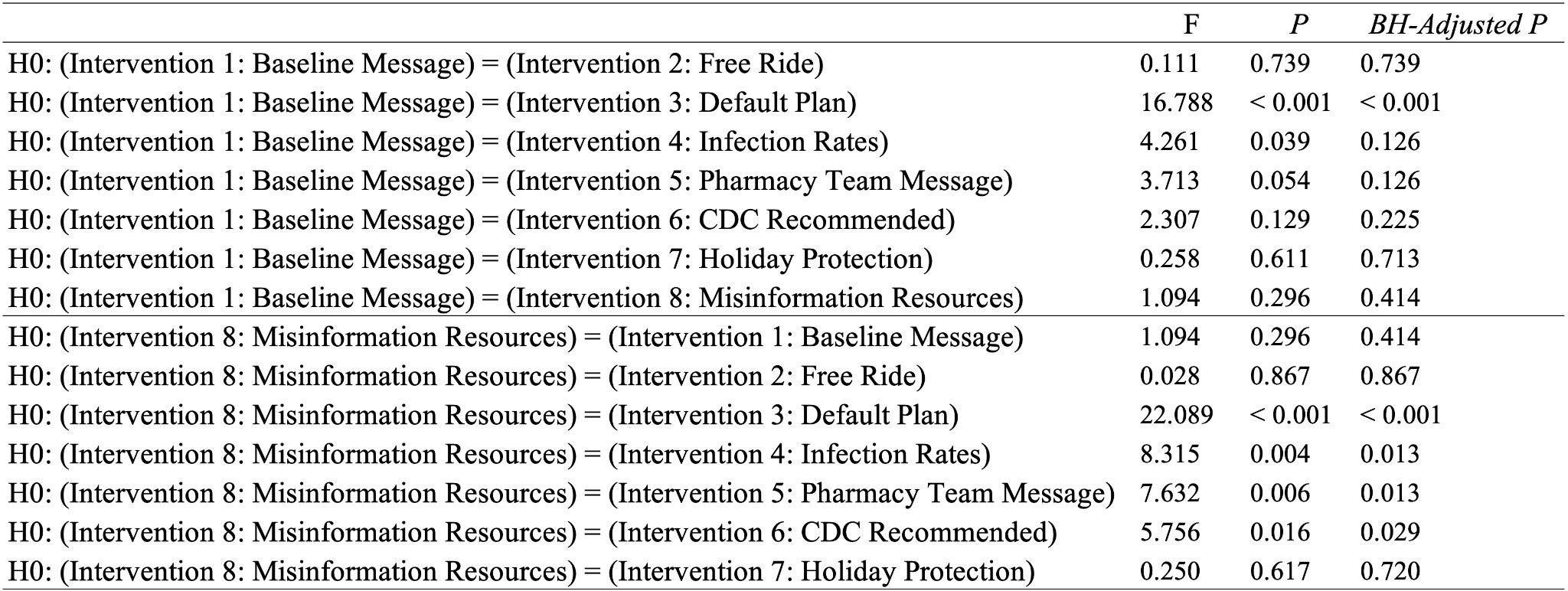


*Note.* The Wald tests presented in this table were performed following the estimation of our main regression model (see Table 2, Model 1) and are two-sided. P-values are reported both unadjusted and adjusted for multiple comparisons using the Benjamini-Hochberg (BH) procedure.**SUBGROUP ANALYSES BASED ON PATIENT CHARACTERISTICS**

**Table S4. Female subgroup analyses.** Regression-estimated impact of each of our megastudy’s eight intervention conditions on bivalent COVID-19 booster uptake at CVS Pharmacy within 30 days of a patient’s study launch day for female patients, either breaking out all interventions individually (Model 1), pooling the reminder-only interventions (Model 2), or pooling all interventions (Model 3).

**
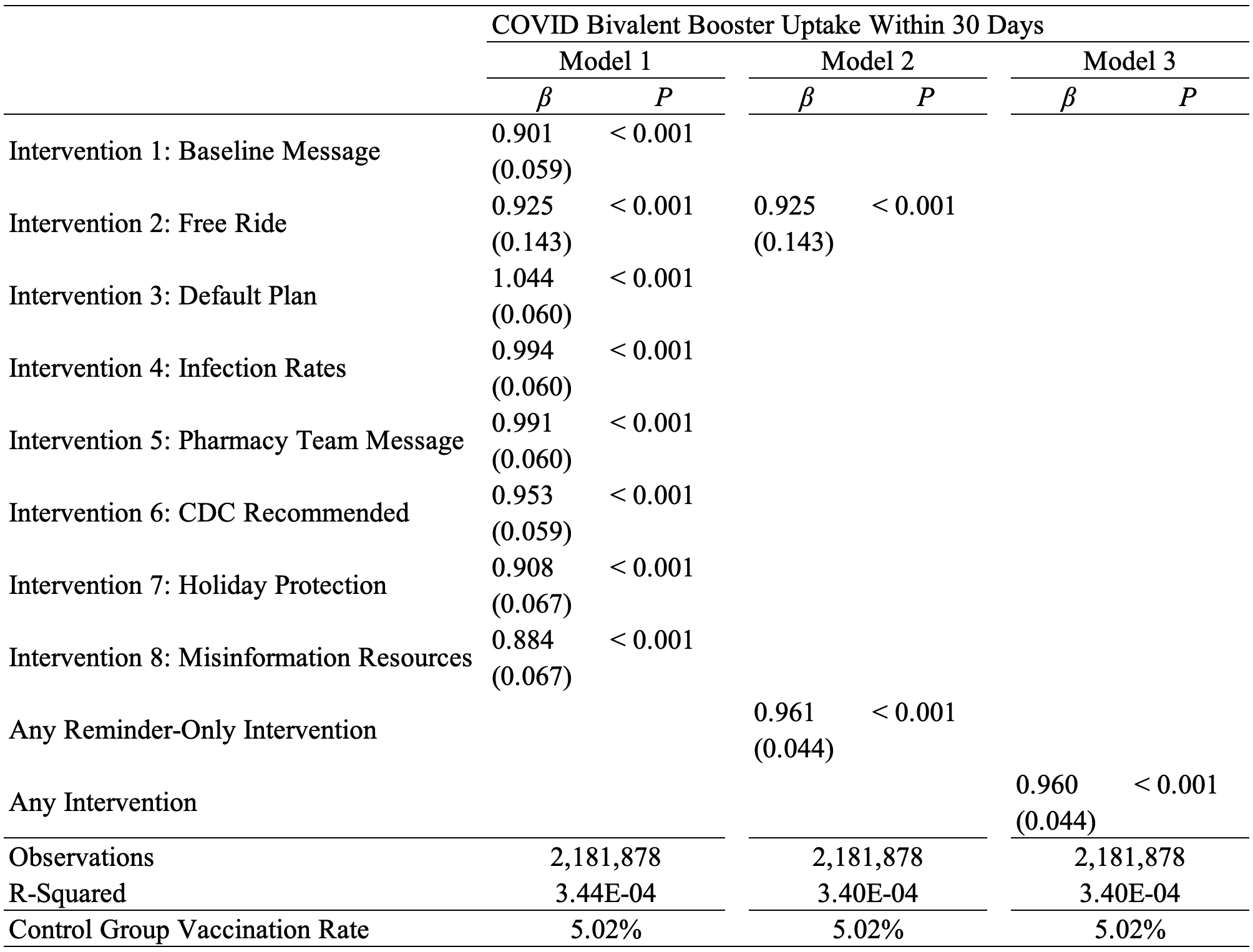
**

*Note:* This table reports the results of three ordinary least squares (OLS) regressions to predict whether a given female patient received a COVID-19 booster vaccine at a CVS Pharmacy within 30 days of a patient’s study launch day. Model 1 relies on the same specification as our main regression model (Table 2, Model 1). Models 2 and 3 include different primary predictors. In Model 2, we include two primary predictors: an indicator for whether a patient received any reminder-only intervention and an indicator for whether a patient received our free ride intervention. In Model 3, we include a single pooled treatment indicator for whether a patient received any of our megastudy’s eight intervention conditions. All three regression models include indicators for whether the patient received their first text message on launch day 1 or launch day 2 (an indicator for receiving a message on launch day 3 is omitted). The control variables in all models are mean-centered using the mean of the holdout control. All regression coefficients and standard errors have been multiplied by 100 to improve interpretability (and thus reflect percentage point change(s) induced in vaccination uptake). Standard errors reported in parentheses are estimated robustly using HC1. Statistical tests of whether an individual regression coefficient is zero are all two-sided.

**Table S5. Male subgroup analyses.** Regression-estimated impact of each of our megastudy’s eight intervention conditions on bivalent COVID-19 booster uptake at CVS Pharmacy within 30 days of a patient’s study launch day for male patients, either breaking out all interventions individually (Model 1), pooling the reminder-only interventions (Model 2), or pooling all interventions (Model 3).

**
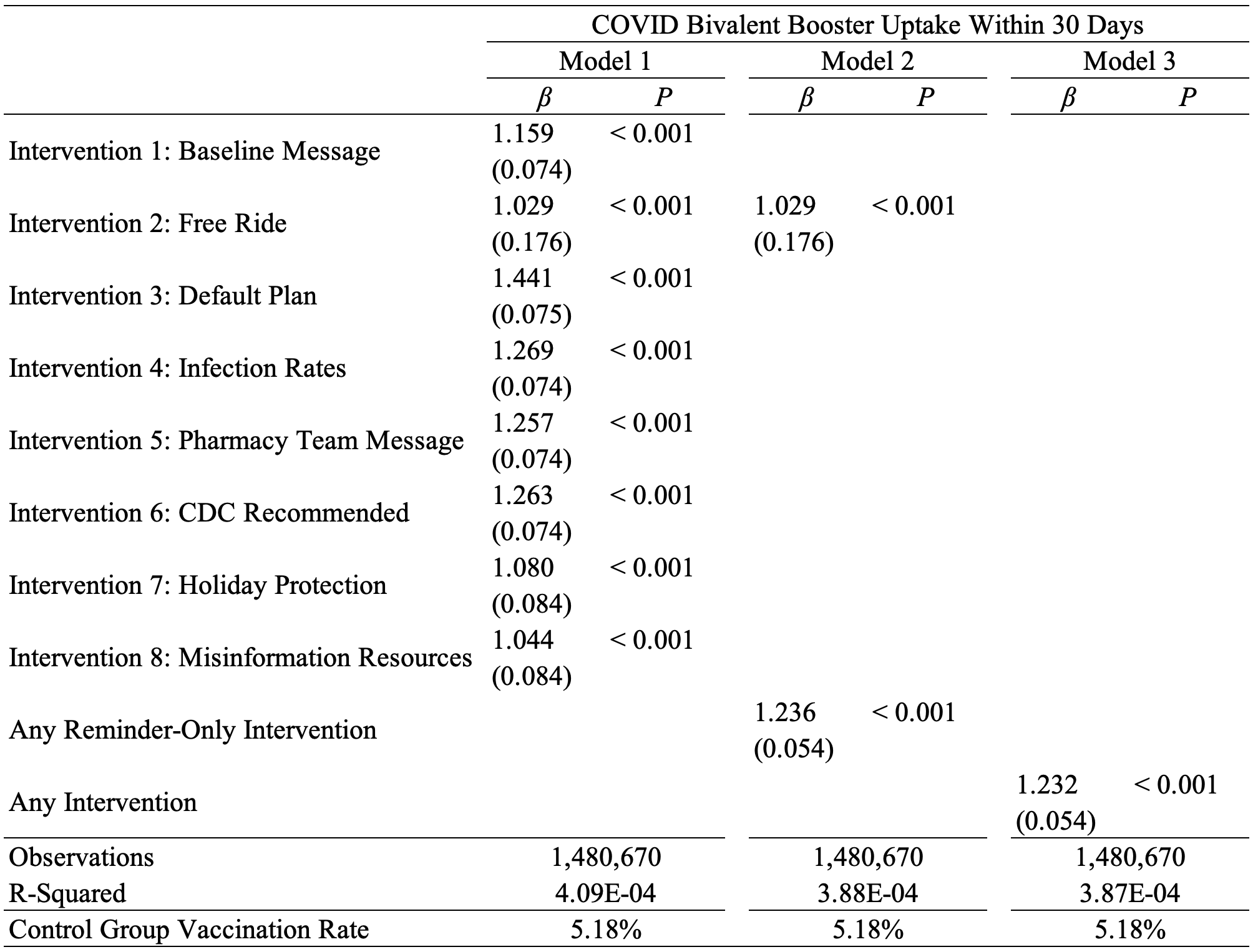
**

*Note:* This table reports the results of three ordinary least squares (OLS) regressions to predict whether a given male patient received a COVID-19 booster vaccine at a CVS Pharmacy within 30 days of a patient’s study launch day. Model 1 relies on the same specification as our main regression model (Table 2, Model 1). Models 2 and 3 include different primary predictors. In Model 2, we include two primary predictors: an indicator for whether a patient received any reminder-only intervention and an indicator for whether a patient received our free ride intervention. In Model 3, we include a single pooled treatment indicator for whether a patient received any of our megastudy’s eight intervention conditions. All three regression models include indicators for whether the patient received their first text message on launch day 1 or launch day 2 (an indicator for receiving a message on launch day 3 is omitted). The control variables in all models are mean-centered using the mean of the holdout control. All regression coefficients and standard errors have been multiplied by 100 to improve interpretability (and thus reflect percentage point change(s) induced in vaccination uptake). Standard errors reported in parentheses are estimated robustly using HC1. Statistical tests of whether an individual regression coefficient is zero are all two-sided.

**Table S6. Subgroup analyses for patients below median age (median age = 47).** Regression-estimated impact of each of our megastudy’s eight intervention conditions on bivalent COVID-19 booster uptake at CVS Pharmacy within 30 days of a patient’s study launch day for below median age patients, either breaking out all interventions individually (Model 1), pooling the reminder-only interventions (Model 2), or pooling all interventions (Model 3).

**
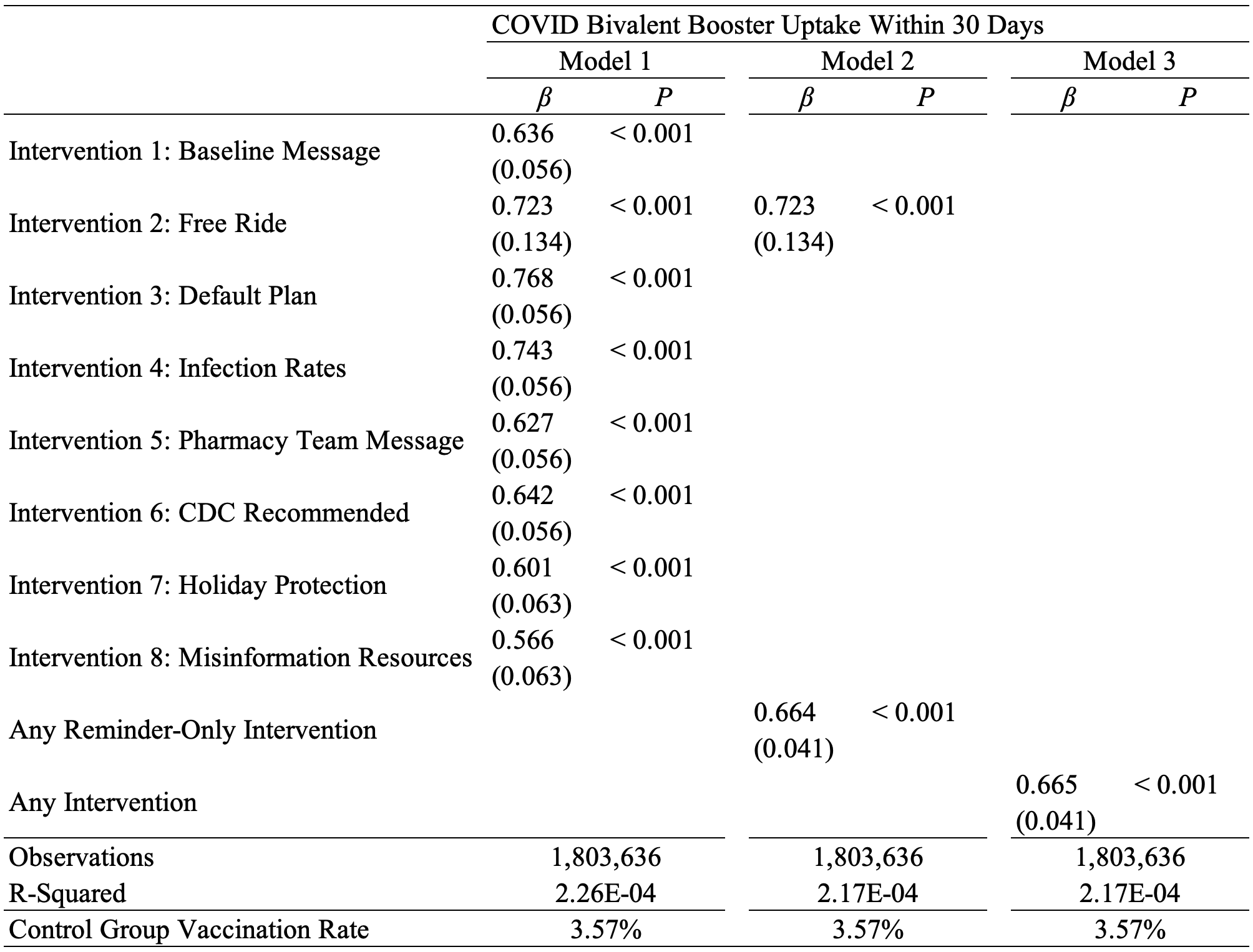
**

*Note:* This table reports the results of three ordinary least squares (OLS) regressions to predict whether a given below median age patient received a COVID-19 booster vaccine at a CVS Pharmacy within 30 days of a patient’s study launch day. Model 1 relies on the same specification as our main regression model (Table 2, Model 1). Models 2 and 3 include different primary predictors. In Model 2, we include two primary predictors: an indicator for whether a patient received any reminder-only intervention and an indicator for whether a patient received our free ride intervention. In Model 3, we include a single pooled treatment indicator for whether a patient received any of our megastudy’s eight intervention conditions. All three regression models include indicators for whether the patient received their first text message on launch day 1 or launch day 2 (an indicator for receiving a message on launch day 3 is omitted). The control variables in all models are mean-centered using the mean of the holdout control. All regression coefficients and standard errors have been multiplied by 100 to improve interpretability (and thus reflect percentage point change(s) induced in vaccination uptake). Standard errors reported in parentheses are estimated robustly using HC1. Statistical tests of whether an individual regression coefficient is zero are all two-sided.

**Table S7. Subgroup analyses for patients at or above median age (median age = 47).** Regression-estimated impact of each of our megastudy’s eight intervention conditions on bivalent COVID-19 booster uptake at CVS Pharmacy within 30 days of a patient’s study launch day for at or above median age patients, either breaking out all interventions individually (Model 1), pooling the reminder-only interventions (Model 2), or pooling all interventions (Model 3).

**
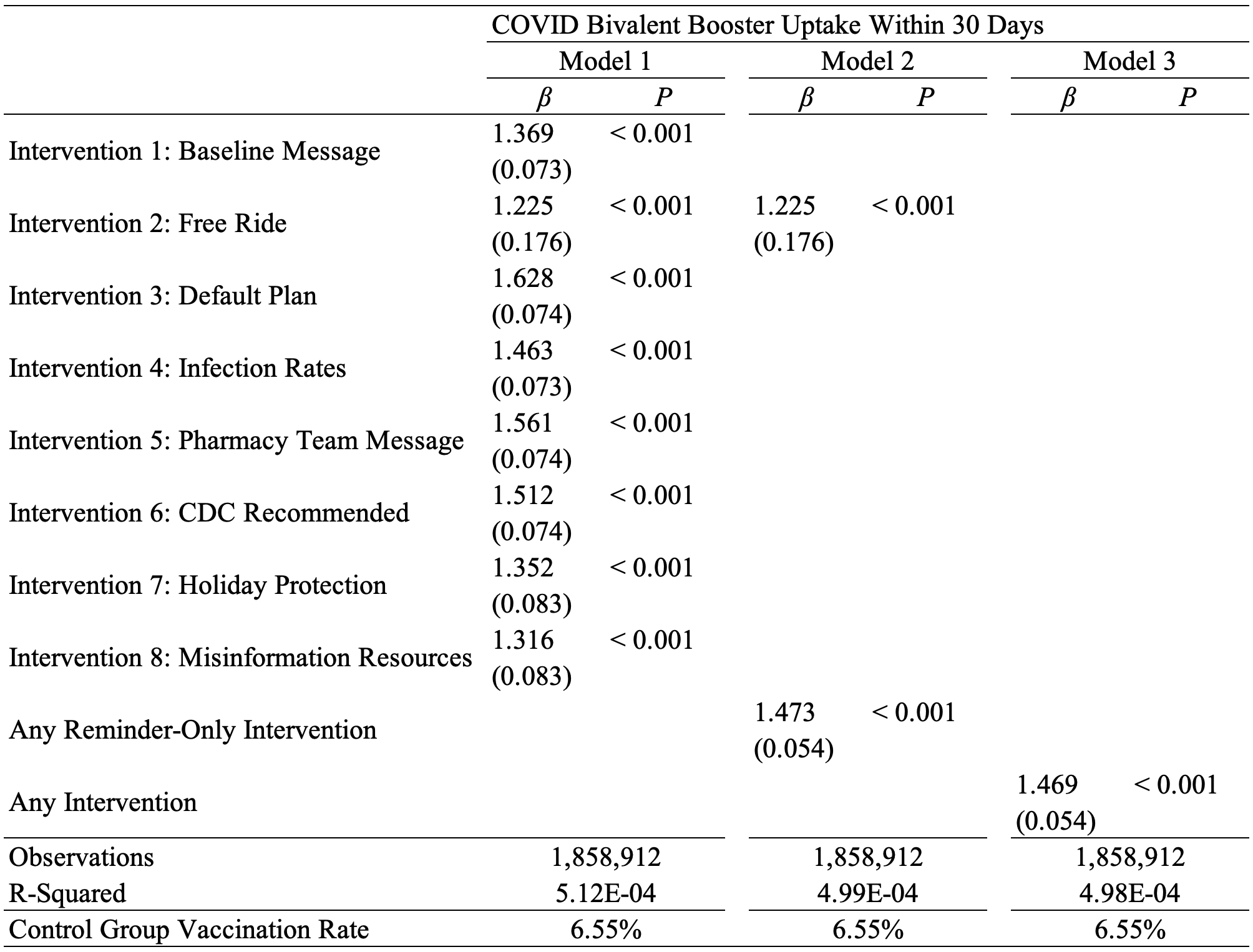
**

*Note:* This table reports the results of three ordinary least squares (OLS) regressions to predict whether a given at or above median age patient received a COVID-19 booster vaccine at a CVS Pharmacy within 30 days of a patient’s study launch day. Model 1 relies on the same specification as our main regression model (Table 2, Model 1). Models 2 and 3 include different primary predictors. In Model 2, we include two primary predictors: an indicator for whether a patient received any reminder-only intervention and an indicator for whether a patient received our free ride intervention. In Model 3, we include a single pooled treatment indicator for whether a patient received any of our megastudy’s eight intervention conditions. All three regression models include indicators for whether the patient received their first text message on launch day 1 or launch day 2 (an indicator for receiving a message on launch day 3 is omitted). The control variables in all models are mean-centered using the mean of the holdout control. All regression coefficients and standard errors have been multiplied by 100 to improve interpretability (and thus reflect percentage point change(s) induced in vaccination uptake). Standard errors reported in parentheses are estimated robustly using HC1. Statistical tests of whether an individual regression coefficient is zero are all two-sided.

**Table S8. Subgroup analyses for patients with no prior booster(s).** Regression-estimated impact of each of our megastudy’s eight intervention conditions on bivalent COVID-19 booster uptake at CVS Pharmacy within 30 days of a patient’s study launch day for patients with no prior booster(s), either breaking out all interventions individually (Model 1), pooling the reminder-only interventions (Model 2), or pooling all interventions (Model 3).

**
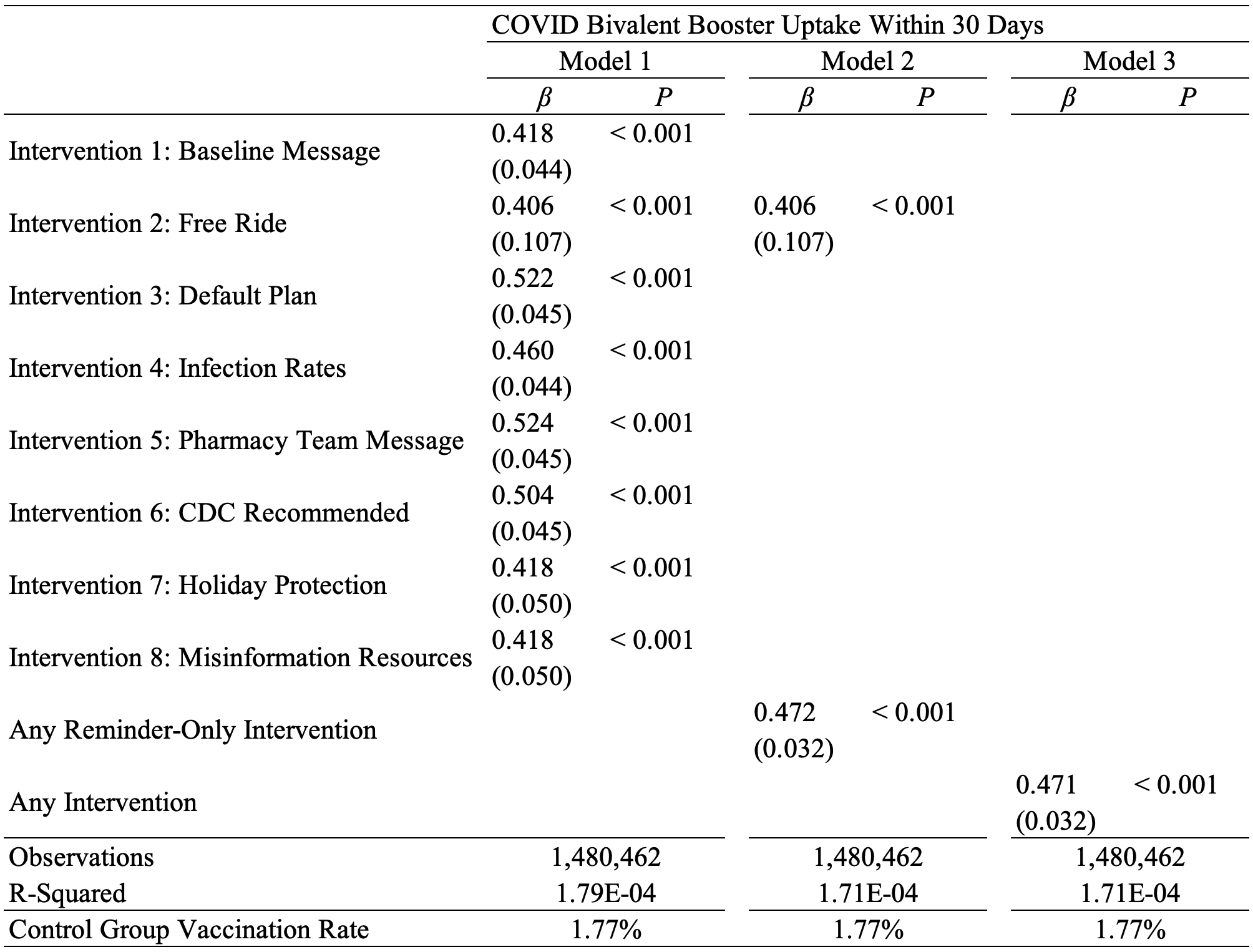
**

*Note:* This table reports the results of three ordinary least squares (OLS) regressions to predict whether a given patient with no prior booster(s) received a COVID-19 booster vaccine at a CVS Pharmacy within 30 days of a patient’s study launch day. Model 1 relies on the same specification as our main regression model (Table 2, Model 1). Models 2 and 3 include different primary predictors. In Model 2, we include two primary predictors: an indicator for whether a patient received any reminder-only intervention and an indicator for whether a patient received our free ride intervention. In Model 3, we include a single pooled treatment indicator for whether a patient received any of our megastudy’s eight intervention conditions. All three regression models include indicators for whether the patient received their first text message on launch day 1 or launch day 2 (an indicator for receiving a message on launch day 3 is omitted). The control variables in all models are mean-centered using the mean of the holdout control. All regression coefficients and standard errors have been multiplied by 100 to improve interpretability (and thus reflect percentage point change(s) induced in vaccination uptake). Standard errors reported in parentheses are estimated robustly using HC1. Statistical tests of whether an individual regression coefficient is zero are all two-sided.

**Table S9. Subgroup analyses for patients with one or more prior booster(s).** Regression-estimated impact of each of our megastudy’s eight intervention conditions on bivalent COVID-19 booster uptake at CVS Pharmacy within 30 days of a patient’s study launch day for patients with one or more prior booster(s), either breaking out all interventions individually (Model 1), pooling the reminder-only interventions (Model 2), or pooling all interventions (Model 3).

**
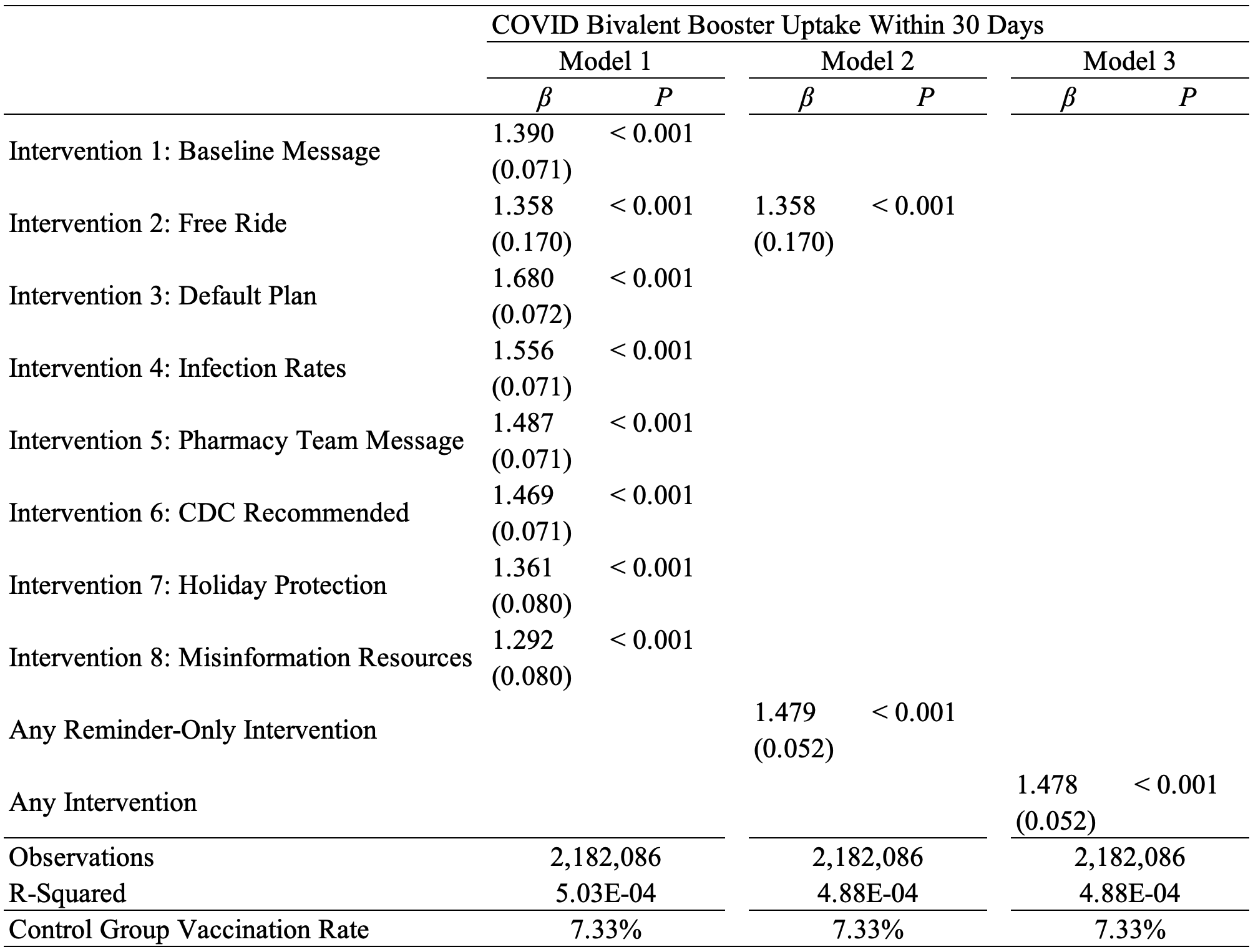
**

*Note:* This table reports the results of three ordinary least squares (OLS) regressions to predict whether a given patient with one or more prior booster(s) received a COVID-19 booster vaccine at a CVS Pharmacy within 30 days of a patient’s study launch day. Model 1 relies on the same specification as our main regression model (Table 2, Model 1). Models 2 and 3 include different primary predictors. In Model 2, we include two primary predictors: an indicator for whether a patient received any reminder-only intervention and an indicator for whether a patient received our free ride intervention. In Model 3, we include a single pooled treatment indicator for whether a patient received any of our megastudy’s eight intervention conditions. All three regression models include indicators for whether the patient received their first text message on launch day 1 or launch day 2 (an indicator for receiving a message on launch day 3 is omitted). The control variables in all models are mean-centered using the mean of the holdout control. All regression coefficients and standard errors have been multiplied by 100 to improve interpretability (and thus reflect percentage point change(s) induced in vaccination uptake). Standard errors reported in parentheses are estimated robustly using HC1. Statistical tests of whether an individual regression coefficient is zero are all two-sided.

**Table S10. Subgroup analyses for patients without Medicare coverage.** Regression-estimated impact of each of our megastudy’s eight intervention conditions on bivalent COVID-19 booster uptake at CVS Pharmacy within 30 days of a patient’s study launch day for non-Medicare patients, either breaking out all interventions individually (Model 1), pooling the reminder-only interventions (Model 2), or pooling all interventions (Model 3).

**
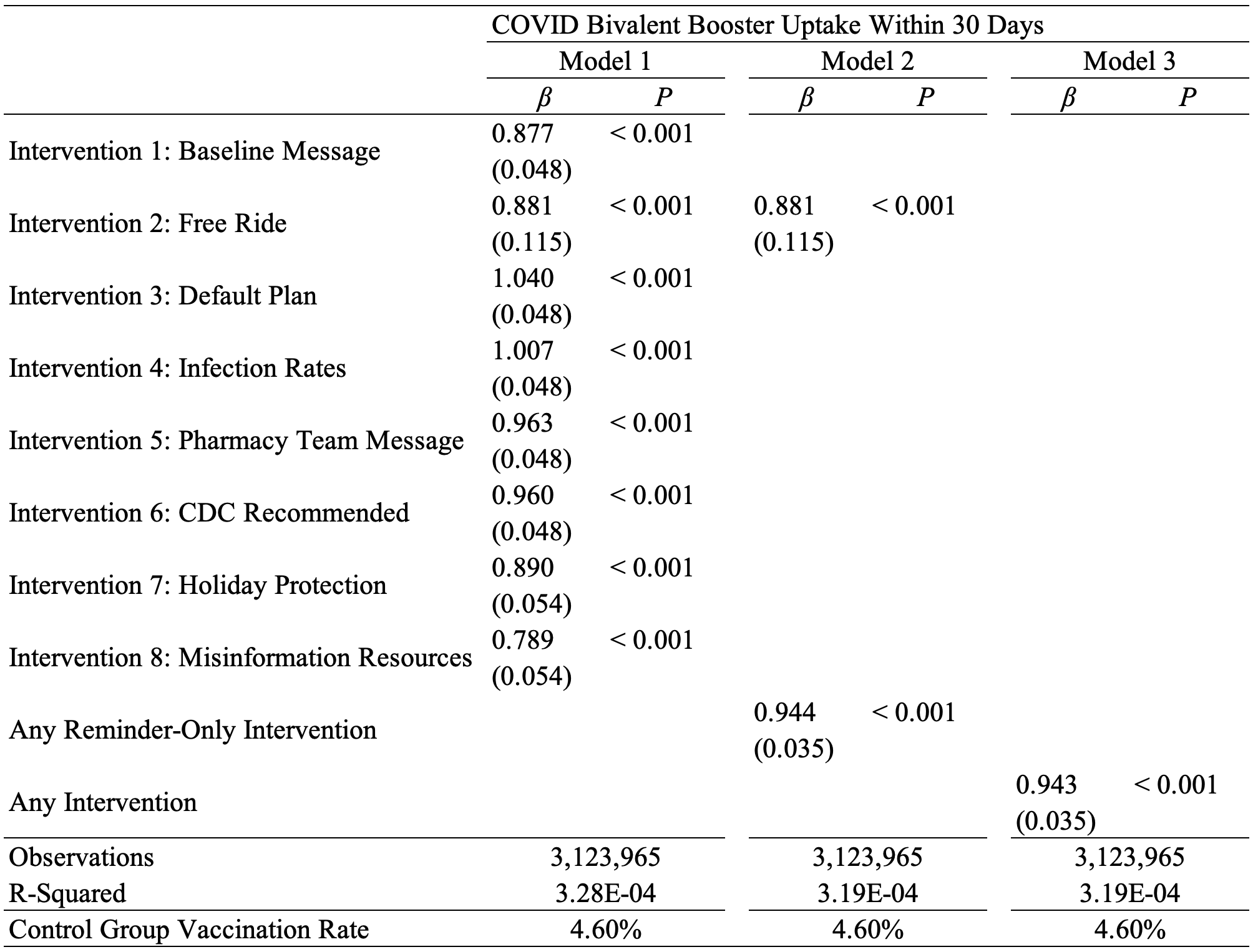
**

*Note:* This table reports the results of three ordinary least squares (OLS) regressions to predict whether a given non-Medicare patient received a COVID-19 booster vaccine at a CVS Pharmacy within 30 days of a patient’s study launch day. Model 1 relies on the same specification as our main regression model (Table 2, Model 1). Models 2 and 3 include different primary predictors. In Model 2, we include two primary predictors: an indicator for whether a patient received any reminder-only intervention and an indicator for whether a patient received our free ride intervention. In Model 3, we include a single pooled treatment indicator for whether a patient received any of our megastudy’s eight intervention conditions. All three regression models include indicators for whether the patient received their first text message on launch day 1 or launch day 2 (an indicator for receiving a message on launch day 3 is omitted). The control variables in all models are mean-centered using the mean of the holdout control. All regression coefficients and standard errors have been multiplied by 100 to improve interpretability (and thus reflect percentage point change(s) induced in vaccination uptake). Standard errors reported in parentheses are estimated robustly using HC1. Statistical tests of whether an individual regression coefficient is zero are all two-sided.

**Table S11. Subgroup analyses for patients with Medicare coverage.** Regression-estimated impact of each of our megastudy’s eight intervention conditions on bivalent COVID-19 booster uptake at CVS Pharmacy within 30 days of a patient’s study launch day for Medicare patients, either breaking out all interventions individually (Model 1), pooling the reminder-only interventions (Model 2), or pooling all interventions (Model 3).

**
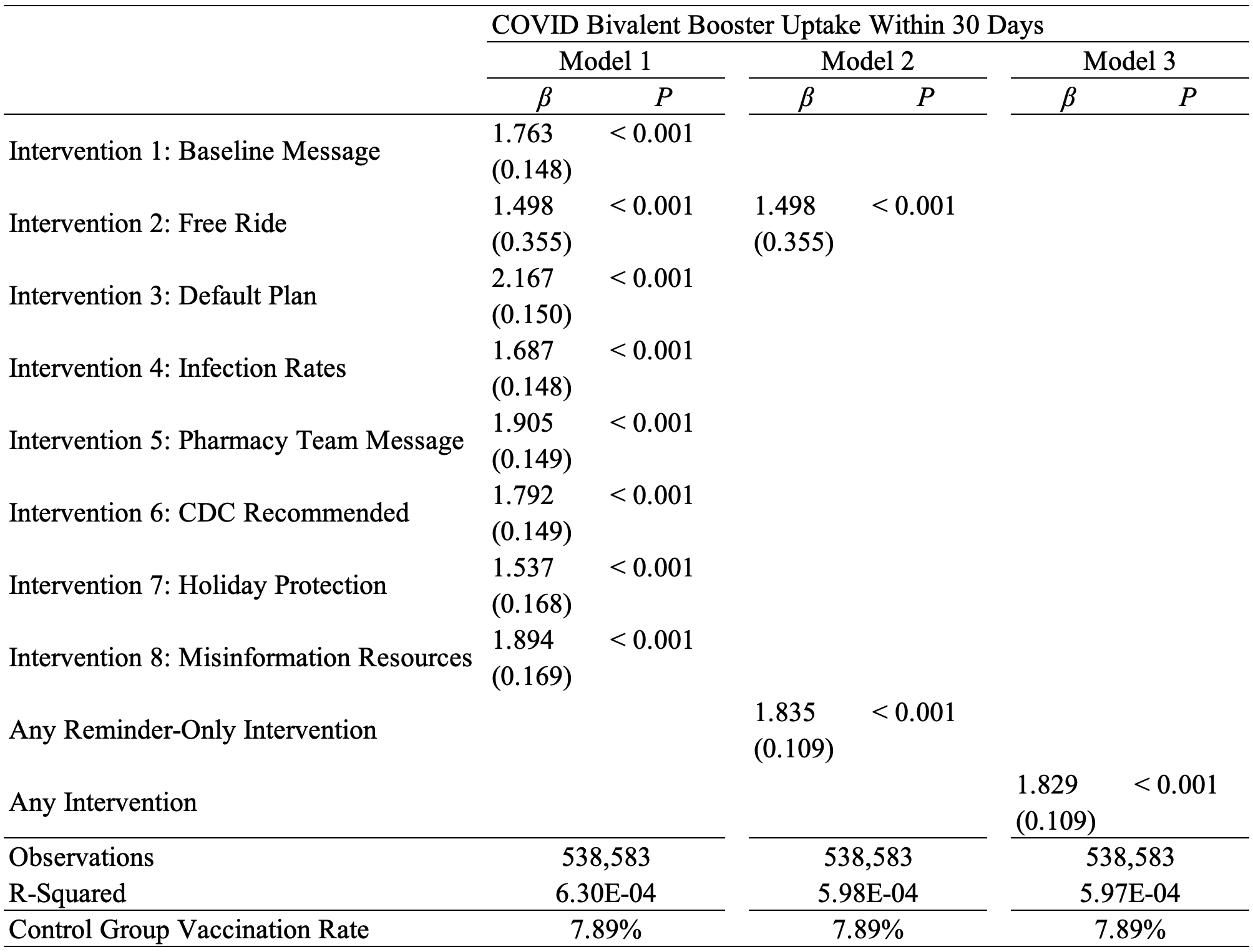
**

*Note:* This table reports the results of three ordinary least squares (OLS) regressions to predict whether a given Medicare patient received a COVID-19 booster vaccine at a CVS Pharmacy within 30 days of a patient’s study launch day. Model 1 relies on the same specification as our main regression model (Table 2, Model 1). Models 2 and 3 include different primary predictors. In Model 2, we include two primary predictors: an indicator for whether a patient received any reminder-only intervention and an indicator for whether a patient received our free ride intervention. In Model 3, we include a single pooled treatment indicator for whether a patient received any of our megastudy’s eight intervention conditions. All three regression models include indicators for whether the patient received their first text message on launch day 1 or launch day 2 (an indicator for receiving a message on launch day 3 is omitted). The control variables in all models are mean-centered using the mean of the holdout control. All regression coefficients and standard errors have been multiplied by 100 to improve interpretability (and thus reflect percentage point change(s) induced in vaccination uptake). Standard errors reported in parentheses are estimated robustly using HC1. Statistical tests of whether an individual regression coefficient is zero are all two-sided.

**Table S12. Subgroup analyses for patients without Medicaid coverage.** Regression-estimated impact of each of our megastudy’s eight intervention conditions on bivalent COVID-19 booster uptake at CVS Pharmacy within 30 days of a patient’s study launch day for non-Medicaid patients, either breaking out all interventions individually (Model 1), pooling the reminder-only interventions (Model 2), or pooling all interventions (Model 3).

**
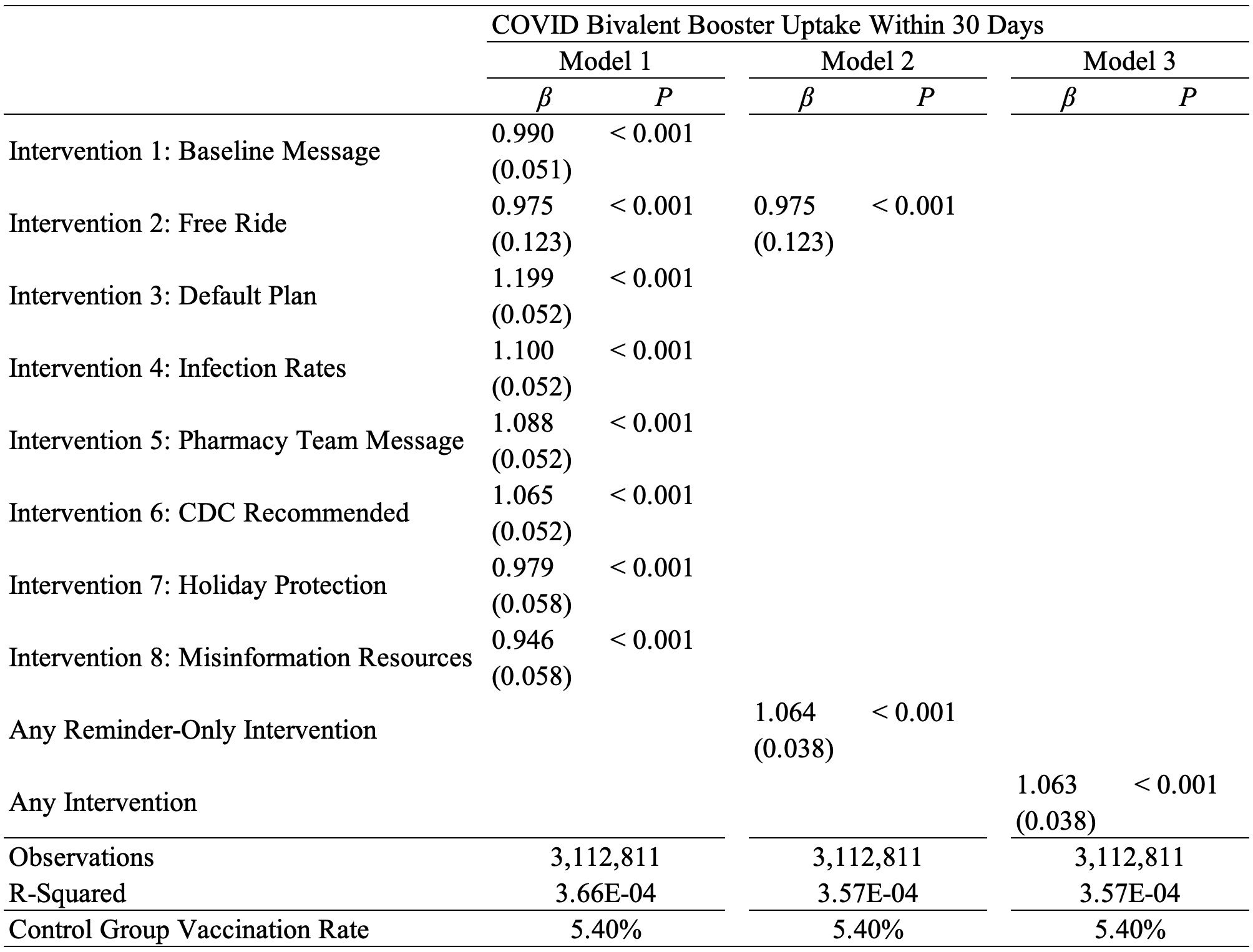
**

*Note:* This table reports the results of three ordinary least squares (OLS) regressions to predict whether a given non-Medicaid patient received a COVID-19 booster vaccine at a CVS Pharmacy within 30 days of a patient’s study launch day. Model 1 relies on the same specification as our main regression model (Table 2, Model 1). Models 2 and 3 include different primary predictors. In Model 2, we include two primary predictors: an indicator for whether a patient received any reminder-only intervention and an indicator for whether a patient received our free ride intervention. In Model 3, we include a single pooled treatment indicator for whether a patient received any of our megastudy’s eight intervention conditions. All three regression models include indicators for whether the patient received their first text message on launch day 1 or launch day 2 (an indicator for receiving a message on launch day 3 is omitted). The control variables in all models are mean-centered using the mean of the holdout control. All regression coefficients and standard errors have been multiplied by 100 to improve interpretability (and thus reflect percentage point change(s) induced in vaccination uptake). Standard errors reported in parentheses are estimated robustly using HC1. Statistical tests of whether an individual regression coefficient is zero are all two-sided.

**Table S13. Subgroup analyses for patients with Medicaid coverage.** Regression-estimated impact of each of our megastudy’s eight intervention conditions on bivalent COVID-19 booster uptake at CVS Pharmacy within 30 days of a patient’s study launch day for Medicaid patients, either breaking out all interventions individually (Model 1), pooling the reminder-only interventions (Model 2), or pooling all interventions (Model 3).

**
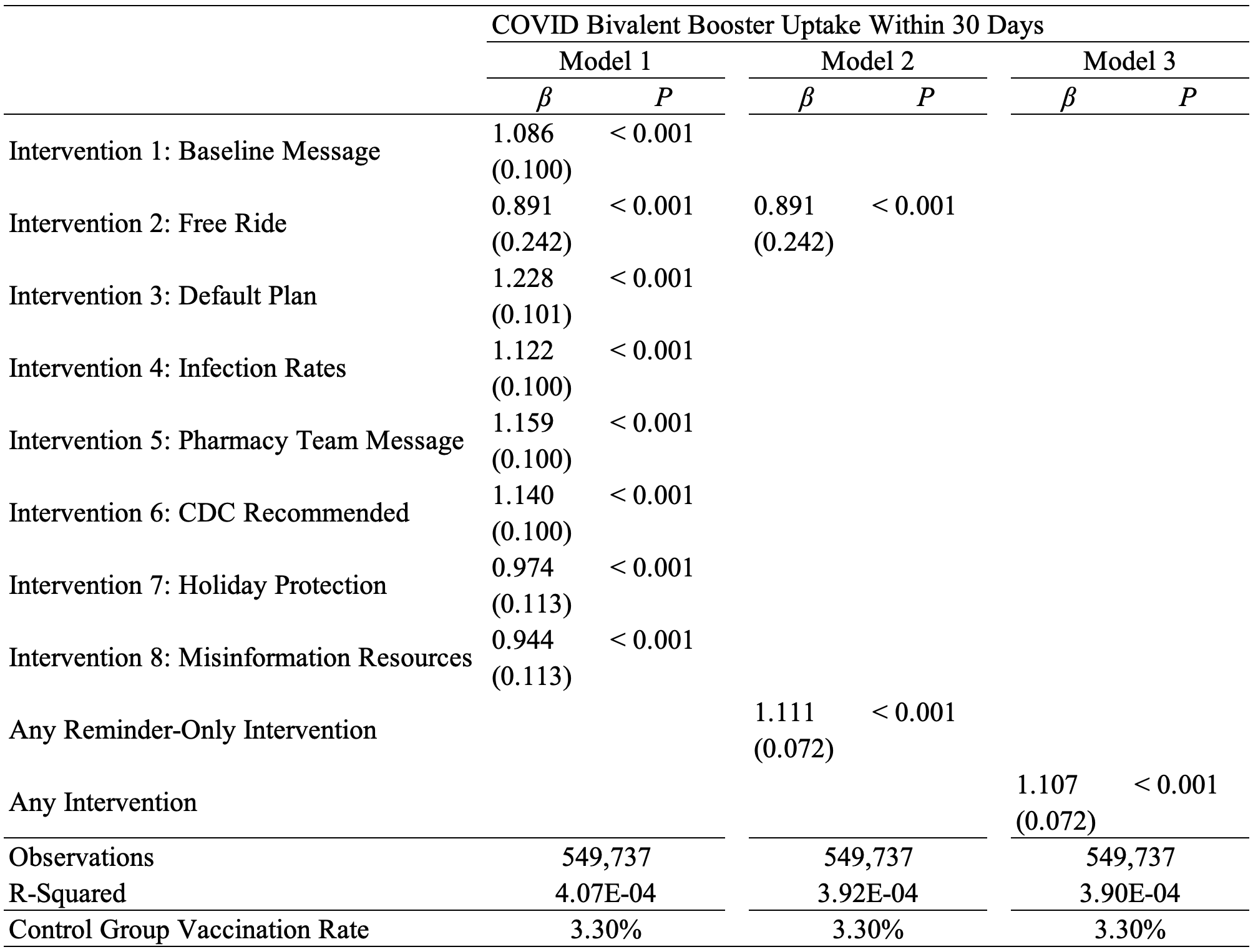
**

*Note:* This table reports the results of three ordinary least squares (OLS) regressions to predict whether a given Medicaid patient received a COVID-19 booster vaccine at a CVS Pharmacy within 30 days of a patient’s study launch day. Model 1 relies on the same specification as our main regression model (Table 2, Model 1). Models 2 and 3 include different primary predictors. In Model 2, we include two primary predictors: an indicator for whether a patient received any reminder-only intervention and an indicator for whether a patient received our free ride intervention. In Model 3, we include a single pooled treatment indicator for whether a patient received any of our megastudy’s eight intervention conditions. All three regression models include indicators for whether the patient received their first text message on launch day 1 or launch day 2 (an indicator for receiving a message on launch day 3 is omitted). The control variables in all models are mean-centered using the mean of the holdout control. All regression coefficients and standard errors have been multiplied by 100 to improve interpretability (and thus reflect percentage point change(s) induced in vaccination uptake). Standard errors reported in parentheses are estimated robustly using HC1. Statistical tests of whether an individual regression coefficient is zero are all two-sided.

**Table S14. Subgroup analyses for patients without commercial insurance.** Regression-estimated impact of each of our megastudy’s eight intervention conditions on bivalent COVID-19 booster uptake at CVS Pharmacy within 30 days of a patient’s study launch day for patients without commercial insurance, either breaking out all interventions individually (Model 1), pooling the reminder-only interventions (Model 2), or pooling all interventions (Model 3).

**
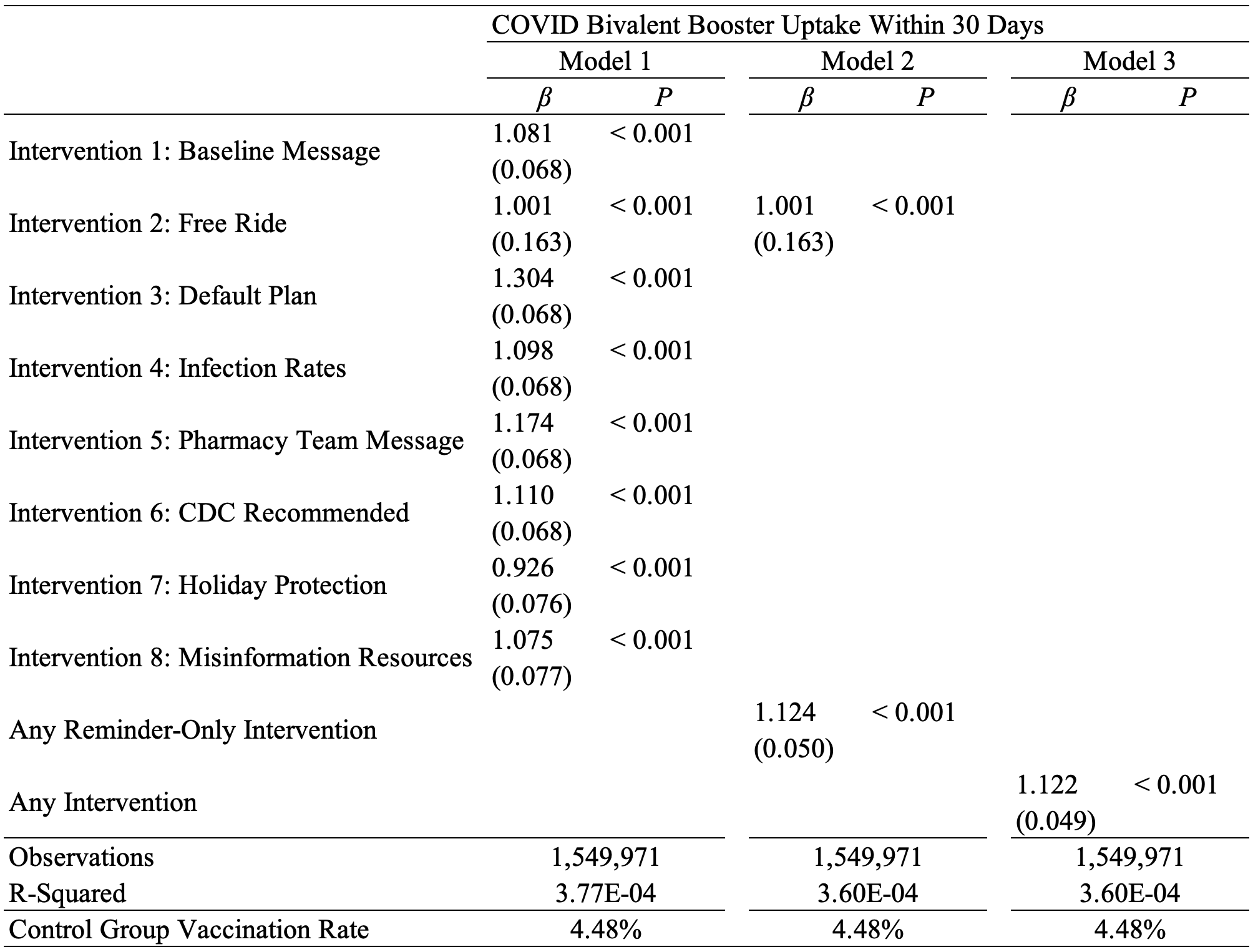
**

*Note:* This table reports the results of three ordinary least squares (OLS) regressions to predict whether a given patient without commercial insurance received a COVID-19 booster vaccine at a CVS Pharmacy within 30 days of a patient’s study launch day. Model 1 relies on the same specification as our main regression model (Table 2, Model 1). Models 2 and 3 include different primary predictors. In Model 2, we include two primary predictors: an indicator for whether a patient received any reminder-only intervention and an indicator for whether a patient received our free ride intervention. In Model 3, we include a single pooled treatment indicator for whether a patient received any of our megastudy’s eight intervention conditions. All three regression models include indicators for whether the patient received their first text message on launch day 1 or launch day 2 (an indicator for receiving a message on launch day 3 is omitted). The control variables in all models are mean-centered using the mean of the holdout control. All regression coefficients and standard errors have been multiplied by 100 to improve interpretability (and thus reflect percentage point change(s) induced in vaccination uptake). Standard errors reported in parentheses are estimated robustly using HC1. Statistical tests of whether an individual regression coefficient is zero are all two-sided.

**Table S15. Subgroup analyses for patients with commercial insurance.** Regression-estimated impact of each of our megastudy’s eight intervention conditions on bivalent COVID-19 booster uptake at CVS Pharmacy within 30 days of a patient’s study launch day for patients with commercial insurance, either breaking out all interventions individually (Model 1), pooling the reminder-only interventions (Model 2), or pooling all interventions (Model 3).

**
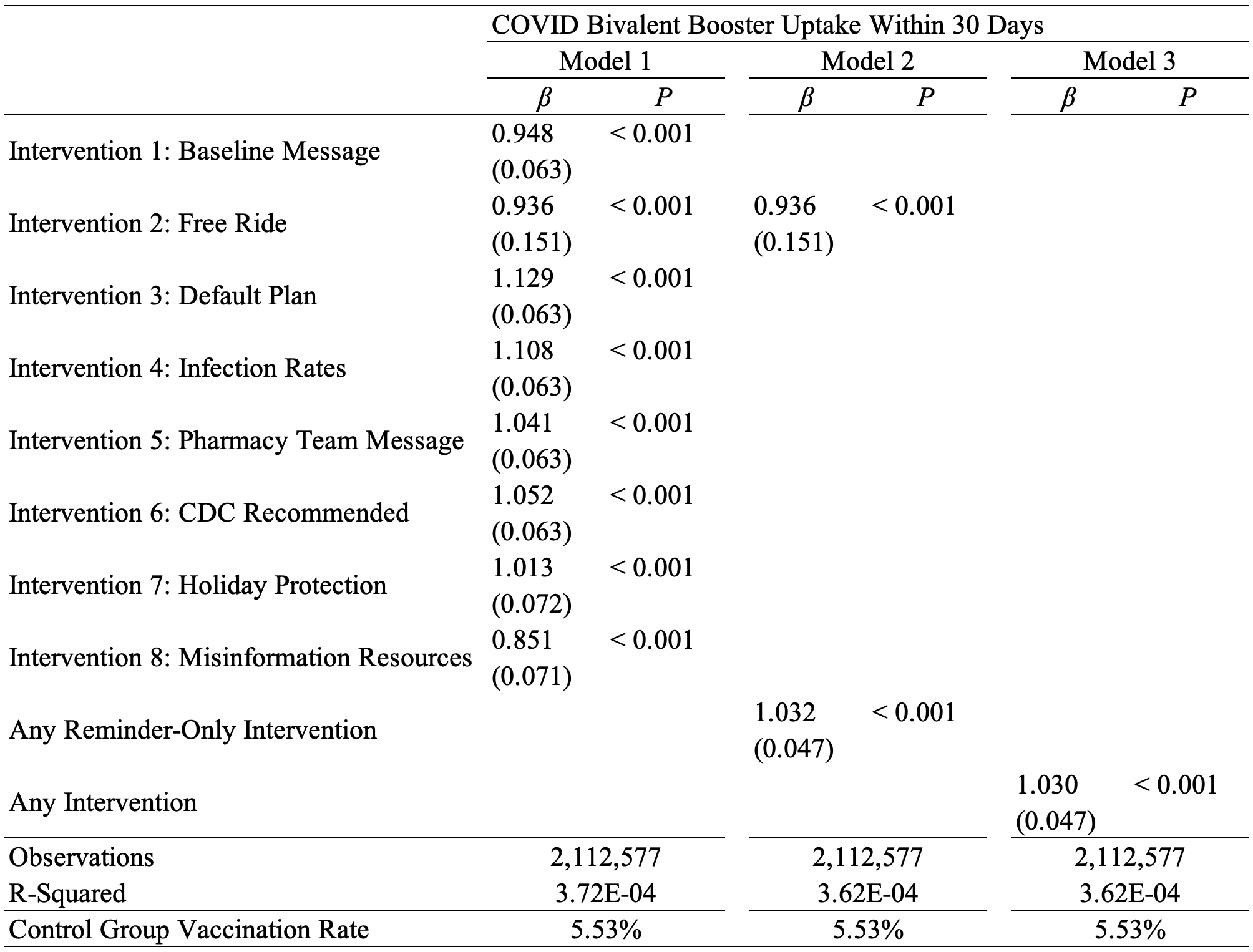
**

*Note:* This table reports the results of three ordinary least squares (OLS) regressions to predict whether a given patient with commercial insurance received a COVID-19 booster vaccine at a CVS Pharmacy within 30 days of a patient’s study launch day. Model 1 relies on the same specification as our main regression model (Table 2, Model 1). Models 2 and 3 include different primary predictors. In Model 2, we include two primary predictors: an indicator for whether a patient received any reminder-only intervention and an indicator for whether a patient received our free ride intervention. In Model 3, we include a single pooled treatment indicator for whether a patient received any of our megastudy’s eight intervention conditions. All three regression models include indicators for whether the patient received their first text message on launch day 1 or launch day 2 (an indicator for receiving a message on launch day 3 is omitted). The control variables in all models are mean-centered using the mean of the holdout control. All regression coefficients and standard errors have been multiplied by 100 to improve interpretability (and thus reflect percentage point change(s) induced in vaccination uptake). Standard errors reported in parentheses are estimated robustly using HC1. Statistical tests of whether an individual regression coefficient is zero are all two-sided.

**Table S16. Subgroup analyses for patients with known insurance.** Regression-estimated impact of each of our megastudy’s eight intervention conditions on bivalent COVID-19 booster uptake at CVS Pharmacy within 30 days of a patient’s study launch day for patients with known insurance, either breaking out all interventions individually (Model 1), pooling the reminder-only interventions (Model 2), or pooling all interventions (Model 3).

**
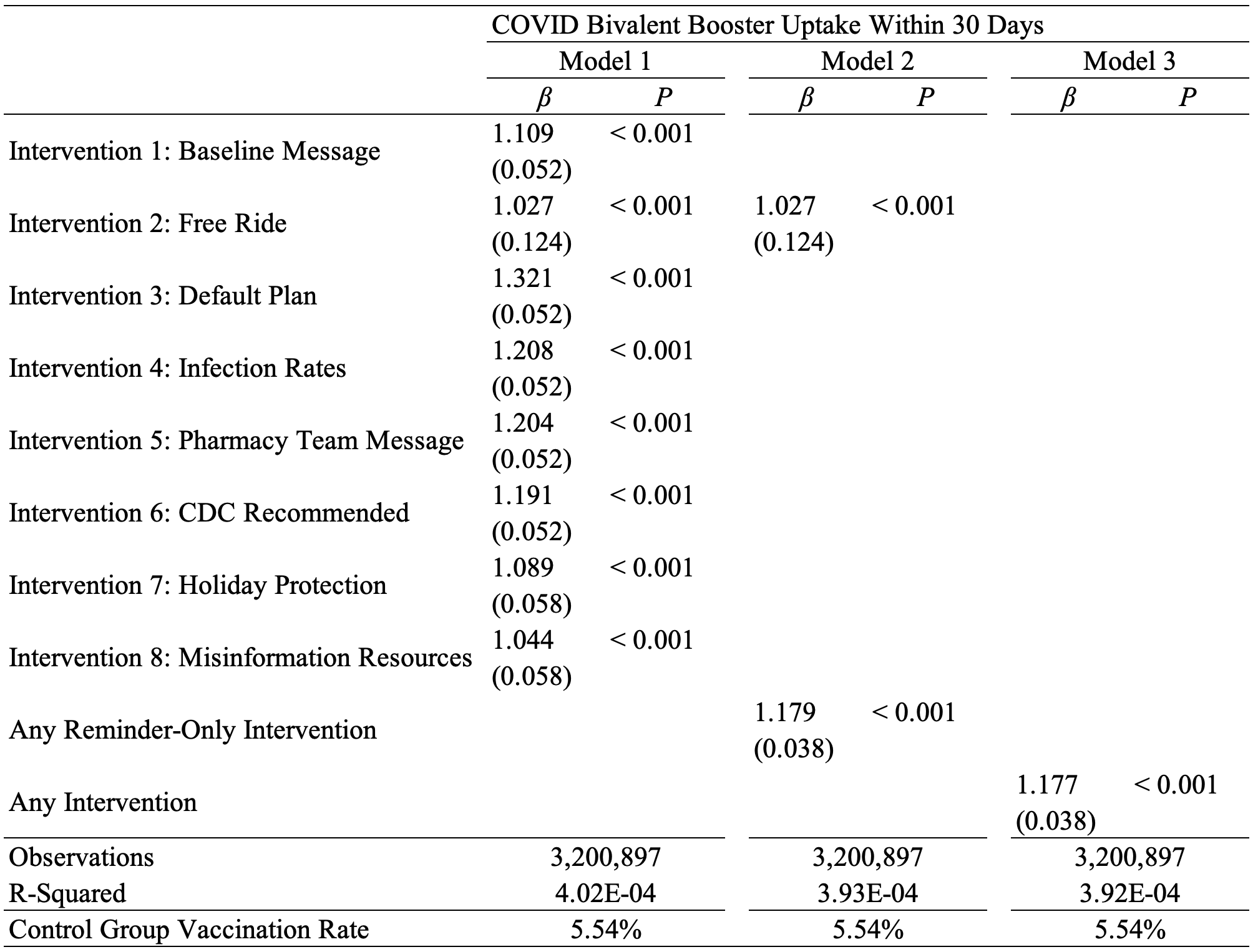
**

*Note:* This table reports the results of three ordinary least squares (OLS) regressions to predict whether a given patient with known insurance received a COVID-19 booster vaccine at a CVS Pharmacy within 30 days of a patient’s study launch day. Model 1 relies on the same specification as our main regression model (Table 2, Model 1). Models 2 and 3 include different primary predictors. In Model 2, we include two primary predictors: an indicator for whether a patient received any reminder-only intervention and an indicator for whether a patient received our free ride intervention. In Model 3, we include a single pooled treatment indicator for whether a patient received any of our megastudy’s eight intervention conditions. All three regression models include indicators for whether the patient received their first text message on launch day 1 or launch day 2 (an indicator for receiving a message on launch day 3 is omitted). The control variables in all models are mean-centered using the mean of the holdout control. All regression coefficients and standard errors have been multiplied by 100 to improve interpretability (and thus reflect percentage point change(s) induced in vaccination uptake). Standard errors reported in parentheses are estimated robustly using HC1. Statistical tests of whether an individual regression coefficient is zero are all two-sided.

**Table S17. Subgroup analyses for patients with unknown insurance.** Regression-estimated impact of each of our megastudy’s eight intervention conditions on bivalent COVID-19 booster uptake at CVS Pharmacy within 30 days of a patient’s study launch day for patients with unknown insurance, either breaking out all interventions individually (Model 1), pooling the reminder-only interventions (Model 2), or pooling all interventions (Model 3).

**
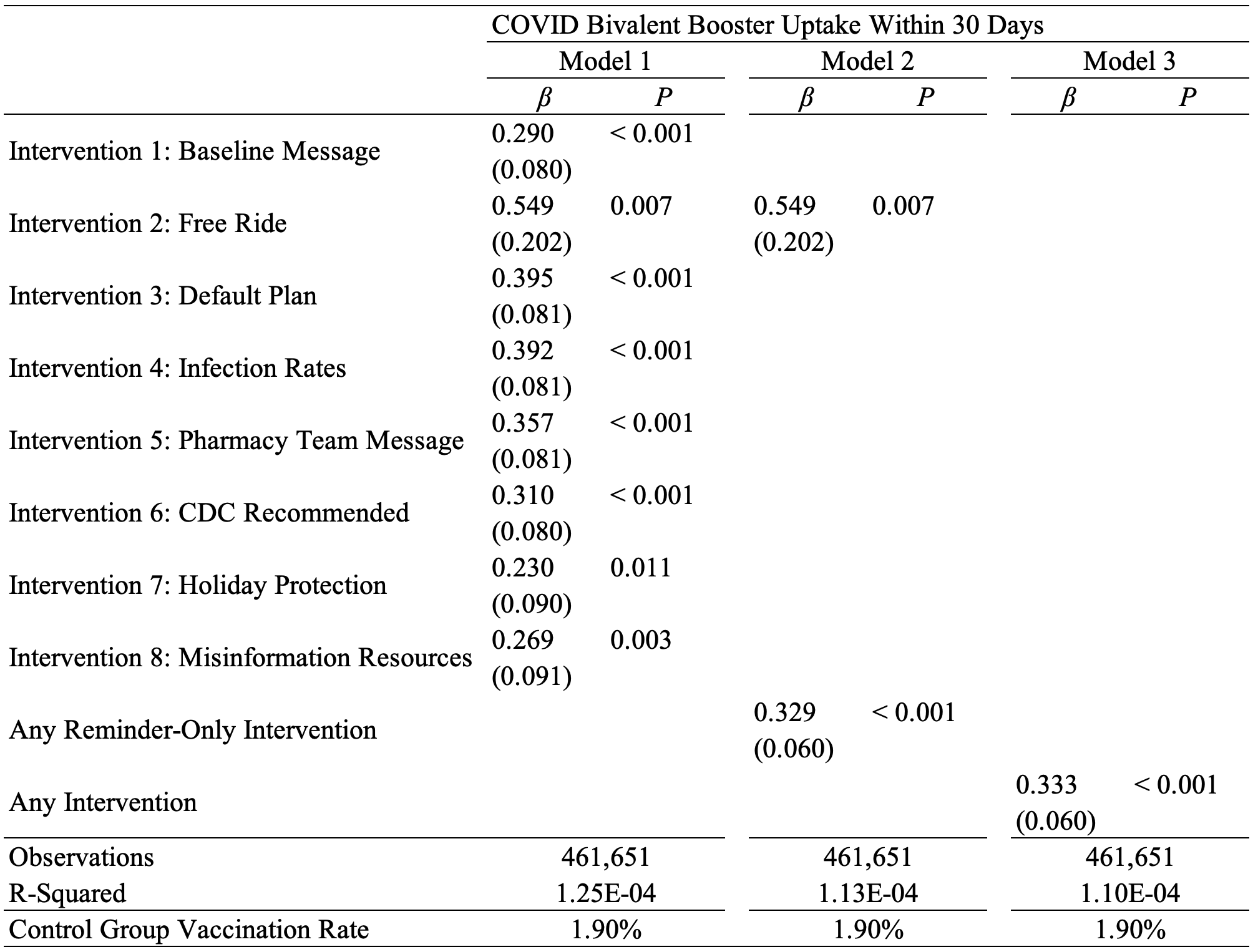
**

*Note:* This table reports the results of three ordinary least squares (OLS) regressions to predict whether a given patient with unknown insurance received a COVID-19 booster vaccine at a CVS Pharmacy within 30 days of a patient’s study launch day. Model 1 relies on the same specification as our main regression model (Table 2, Model 1). Models 2 and 3 include different primary predictors. In Model 2, we include two primary predictors: an indicator for whether a patient received any reminder-only intervention and an indicator for whether a patient received our free ride intervention. In Model 3, we include a single pooled treatment indicator for whether a patient received any of our megastudy’s eight intervention conditions. All three regression models include indicators for whether the patient received their first text message on launch day 1 or launch day 2 (an indicator for receiving a message on launch day 3 is omitted). The control variables in all models are mean-centered using the mean of the holdout control. All regression coefficients and standard errors have been multiplied by 100 to improve interpretability (and thus reflect percentage point change(s) induced in vaccination uptake). Standard errors reported in parentheses are estimated robustly using HC1. Statistical tests of whether an individual regression coefficient is zero are all two-sided.

**HETEROGENEITY ANALYSES BASED ON PATIENT CHARACTERISTICS**

**Table S18. Heterogeneity analyses by patient gender.** Regression-estimated impact of each of our megastudy’s eight intervention conditions on bivalent COVID-19 booster uptake at a CVS Pharmacy within 30 days of a patient’s study launch day as a function of patient gender, either breaking out all interventions individually (Model 1), pooling the reminder-only interventions (Model 2), or pooling all interventions (Model 3).

**
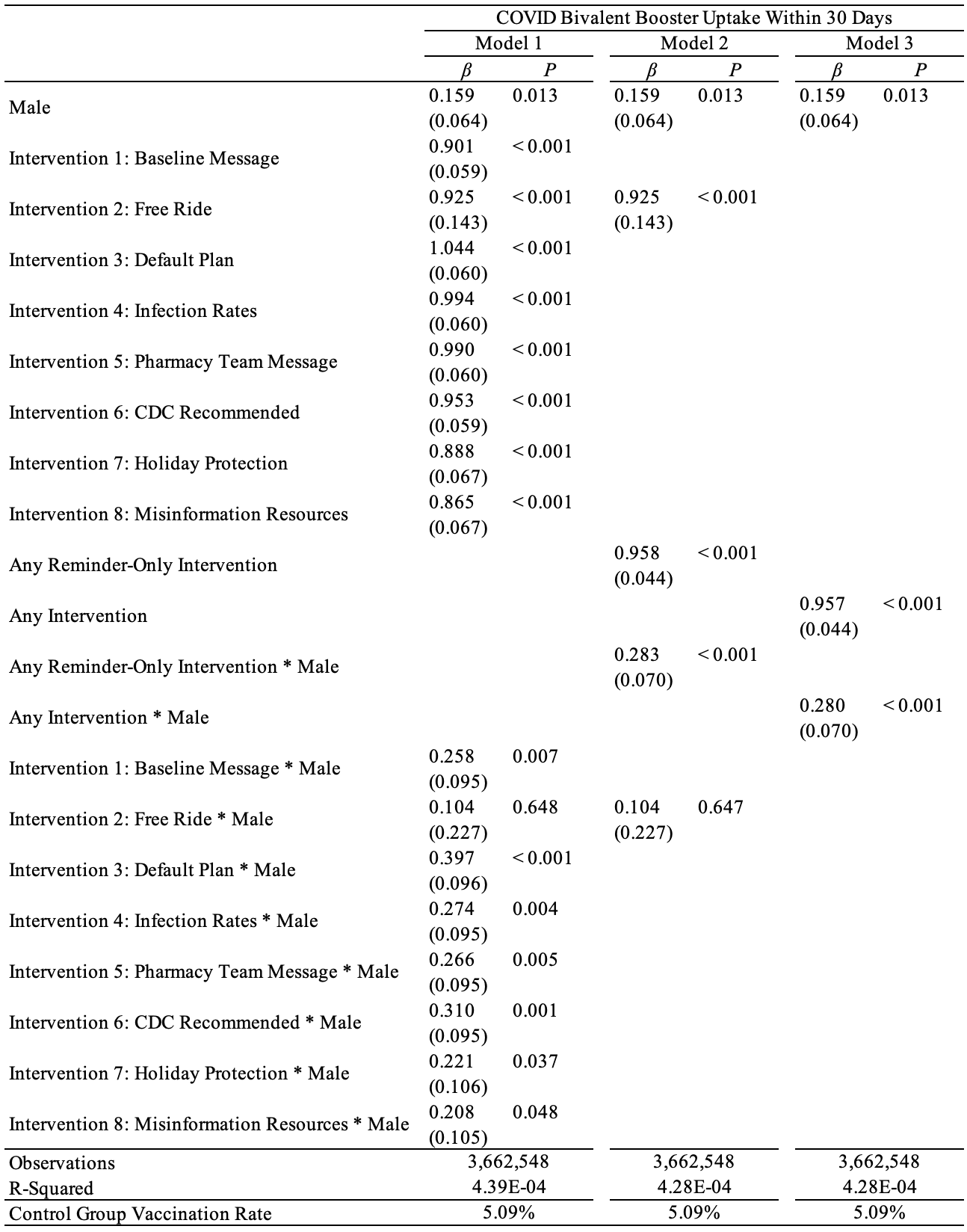
**

*Note:* This table reports the results of three ordinary least squares (OLS) regressions to predict whether a given patient received a COVID-19 booster vaccine at a CVS Pharmacy within 30 days of a patient’s study launch day. Model 1 relies on the same specification as our main regression model (Table 2, Model 1), while Model 2 and 3 include different primary predictors. In Model 2, we include two primary predictors: an indicator for whether a patient received any reminder-only intervention and an indicator for whether a patient received our free ride intervention. In Model 3, we include a single pooled treatment indicator for whether a patient received any of our megastudy’s eight intervention conditions. Each model includes an indicator for whether a patient is male and interactions between this male indicator and each intervention indicator. All three regression models also include indicators for whether the patient received their first text message on launch day 1 or launch day 2 (an indicator for receiving a message on launch day 3 is omitted). The control variables in all models are mean-centered using the mean of the holdout control. All regression coefficients and standard errors have been multiplied by 100 to improve interpretability (and thus reflect percentage point change(s) induced in vaccination uptake). Standard errors reported in parentheses are estimated robustly using HC1. Statistical tests of whether an individual regression coefficient is zero are all two-sided.

**Table S19. Heterogeneity analyses by patient age.** Regression-estimated impact of each of our megastudy’s eight intervention conditions on bivalent COVID-19 booster uptake at a CVS Pharmacy within 30 days of a patient’s study launch day as a function of patient age, either breaking out all interventions individually (Model 1), pooling the reminder-only interventions (Model 2), or pooling all interventions (Model 3).

**
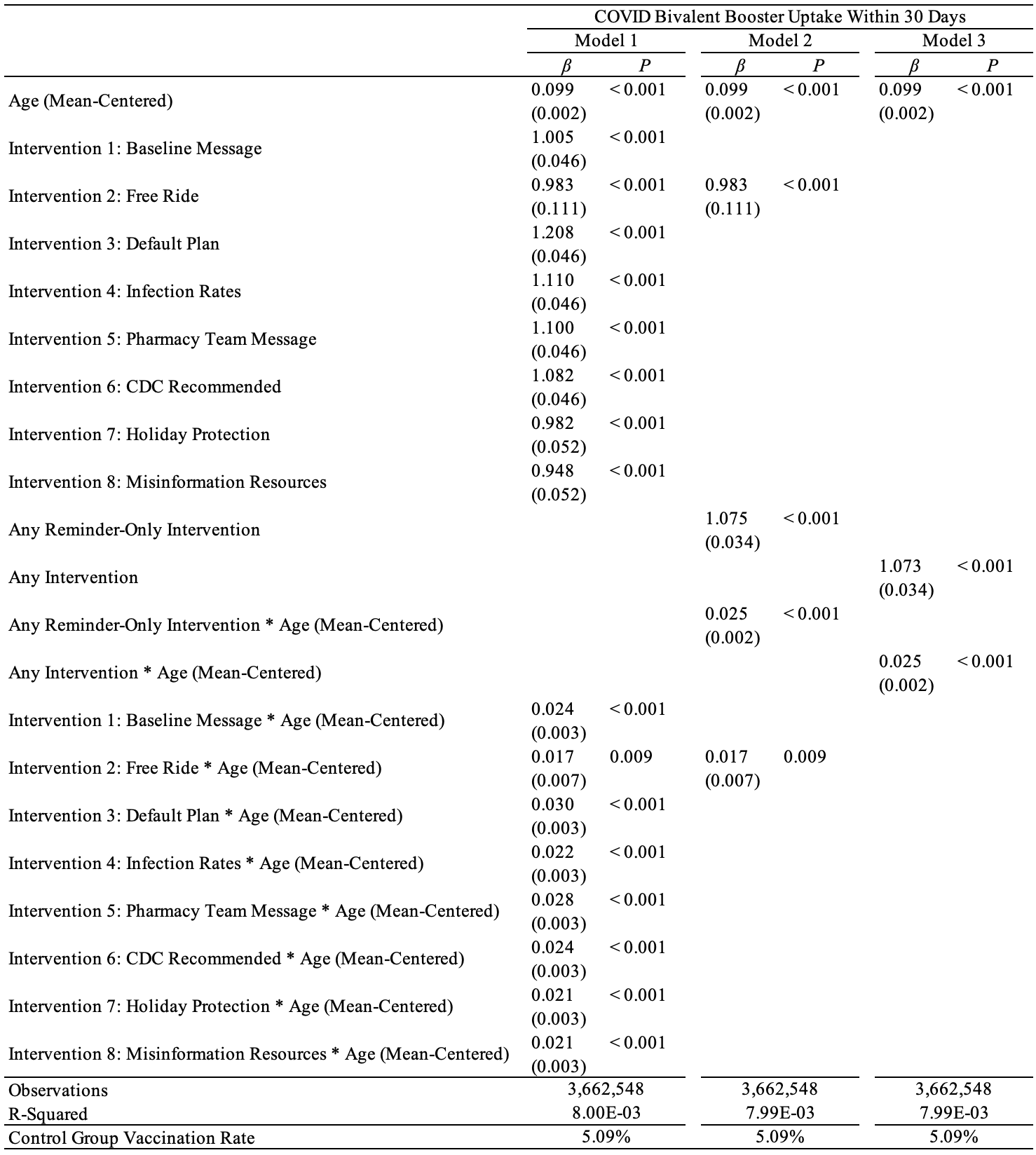
**

*Note:* This table reports the results of three ordinary least squares (OLS) regressions to predict whether a given patient received a COVID-19 booster vaccine at a CVS Pharmacy within 30 days of a patient’s study launch day. Model 1 relies on the same specification as our main regression model (Table 2, Model 1), while Model 2 and 3 include different primary predictors. In Model 2, we include two primary predictors: an indicator for whether a patient received any reminder-only intervention and an indicator for whether a patient received our free ride intervention. In Model 3, we include a single pooled treatment indicator for whether a patient received any of our megastudy’s eight intervention conditions. Each model includes a continuous mean-centered measure of patient age and interactions between this measure of patient age and each intervention indicator. All three regression models also include indicators for whether the patient received their first text message on launch day 1 or launch day 2 (an indicator for receiving a message on launch day 3 is omitted). The control variables in all models are mean-centered using the mean of the holdout control. All regression coefficients and standard errors have been multiplied by 100 to improve interpretability (and thus reflect percentage point change(s) induced in vaccination uptake). Standard errors reported in parentheses are estimated robustly using HC1. Statistical tests of whether an individual regression coefficient is zero are all two-sided.

**Table S20. Heterogeneity analyses by whether a patient has received one or more prior booster(s).** Regression-estimated impact of each of our megastudy’s eight intervention conditions on bivalent COVID-19 booster uptake at a CVS Pharmacy within 30 days of a patient’s study launch day as a function of a patient having received one or more prior booster(s), either breaking out all interventions individually (Model 1), pooling the reminder-only interventions (Model 2), or pooling all interventions (Model 3).

**
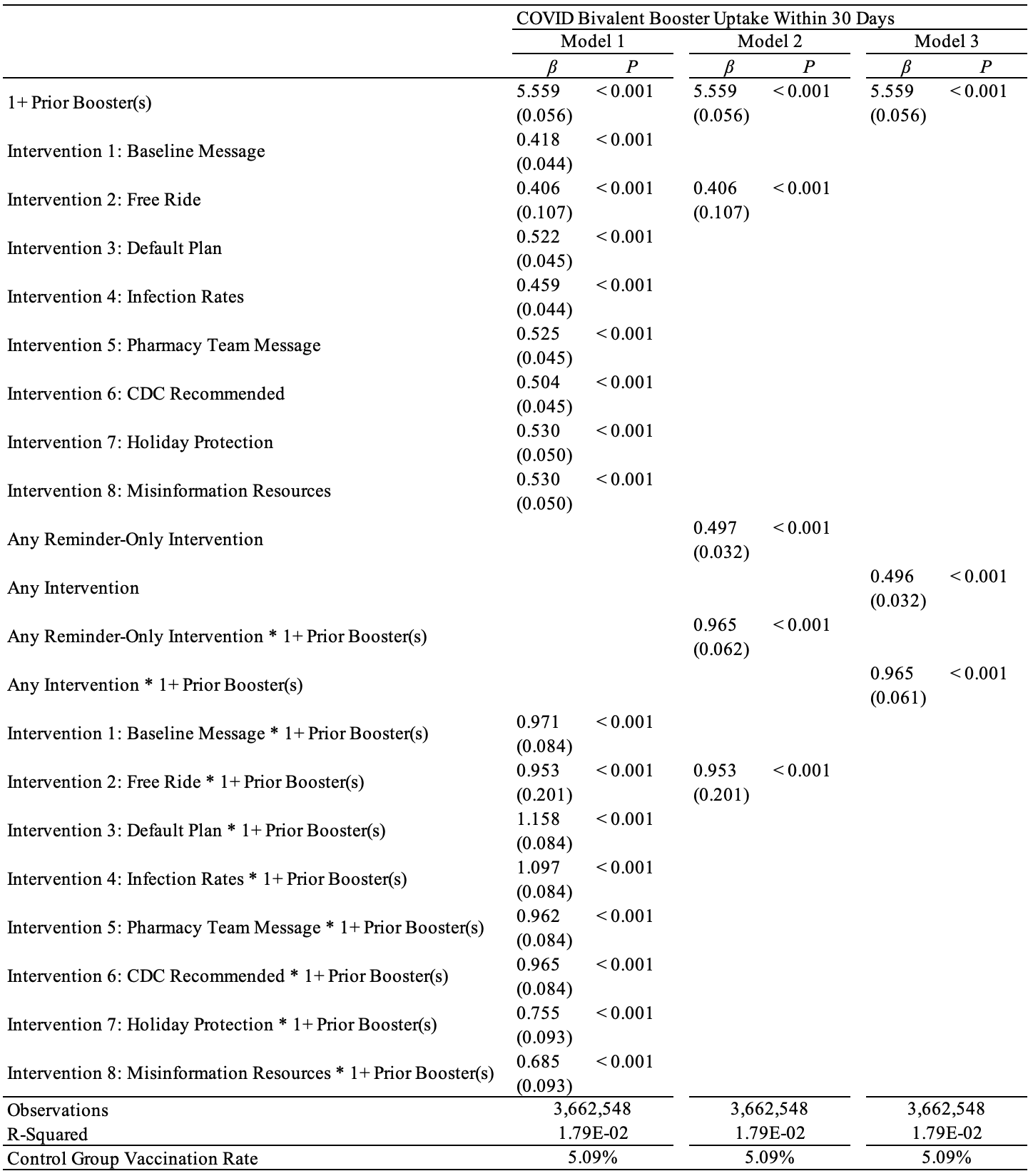
**

*Note:* This table reports the results of three ordinary least squares (OLS) regressions to predict whether a given patient received a COVID-19 booster vaccine at a CVS Pharmacy within 30 days of a patient’s study launch day. Model 1 relies on the same specification as our main regression model (Table 2, Model 1), while Model 2 and 3 include different primary predictors. In Model 2, we include two primary predictors: an indicator for whether a patient received any reminder-only intervention and an indicator for whether a patient received our free ride intervention. In Model 3, we include a single pooled treatment indicator for whether a patient received any of our megastudy’s eight intervention conditions. Each model includes an indicator for whether a patient has received one or more prior booster(s) and interactions between this received one or more prior booster(s) indicator and each intervention indicator. All three regression models also include indicators for whether the patient received their first text message on launch day 1 or launch day 2 (an indicator for receiving a message on launch day 3 is omitted). The control variables in all models are mean-centered using the mean of the holdout control. All regression coefficients and standard errors have been multiplied by 100 to improve interpretability (and thus reflect percentage point change(s) induced in vaccination uptake). Standard errors reported in parentheses are estimated robustly using HC1. Statistical tests of whether an individual regression coefficient is zero are all two-sided.

**Table S21. Heterogeneity analyses by whether a patient has Medicare coverage.** Regression-estimated impact of each of our megastudy’s eight intervention conditions on bivalent COVID-19 booster uptake at a CVS Pharmacy within 30 days of a patient’s study launch day as a function of whether a patient has Medicare coverage, either breaking out all interventions individually (Model 1), pooling the reminder-only interventions (Model 2), or pooling all interventions (Model 3).

**
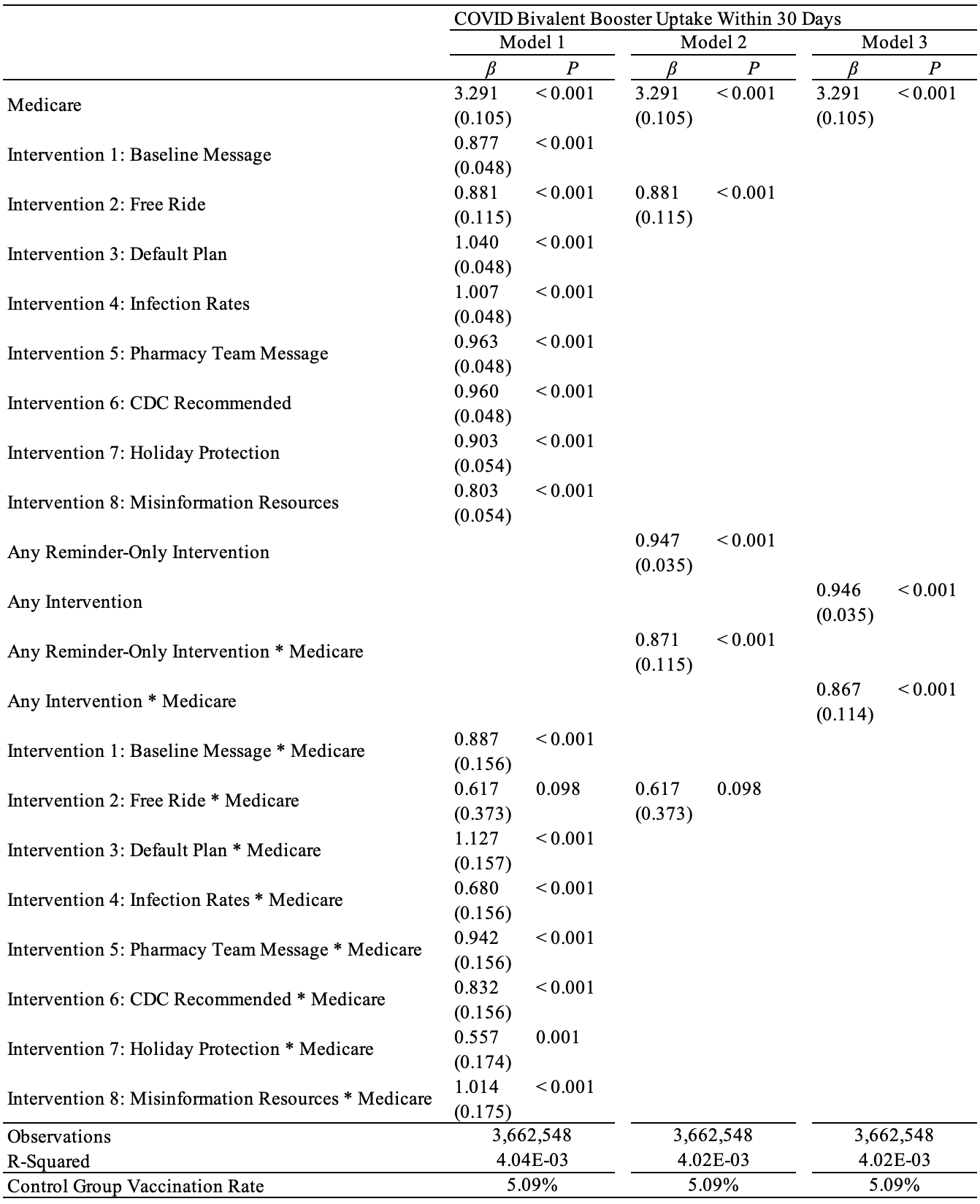
**

*Note:* This table reports the results of three ordinary least squares (OLS) regressions to predict whether a given patient received a COVID-19 booster vaccine at a CVS Pharmacy within 30 days of a patient’s study launch day. Model 1 relies on the same specification as our main regression model (Table 2, Model 1), while Model 2 and 3 include different primary predictors. In Model 2, we include two primary predictors: an indicator for whether a patient received any reminder-only intervention and an indicator for whether a patient received our free ride intervention. In Model 3, we include a single pooled treatment indicator for whether a patient received any of our megastudy’s eight intervention conditions. Each model includes an indicator for whether a patient has Medicare coverage and interactions between this Medicare coverage indicator and each intervention indicator. All three regression models also include indicators for whether the patient received their first text message on launch day 1 or launch day 2 (an indicator for receiving a message on launch day 3 is omitted). The control variables in all models are mean-centered using the mean of the holdout control. All regression coefficients and standard errors have been multiplied by 100 to improve interpretability (and thus reflect percentage point change(s) induced in vaccination uptake). Standard errors reported in parentheses are estimated robustly using HC1. Statistical tests of whether an individual regression coefficient is zero are all two-sided.

**Table S22. Heterogeneity analyses by whether a patient has Medicaid coverage.** Regression-estimated impact of each of our megastudy’s eight intervention conditions on bivalent COVID-19 booster uptake at a CVS Pharmacy within 30 days of a patient’s study launch day as a function of whether a patient has Medicaid coverage, either breaking out all interventions individually (Model 1), pooling the reminder-only interventions (Model 2), or pooling all interventions (Model 3).

**
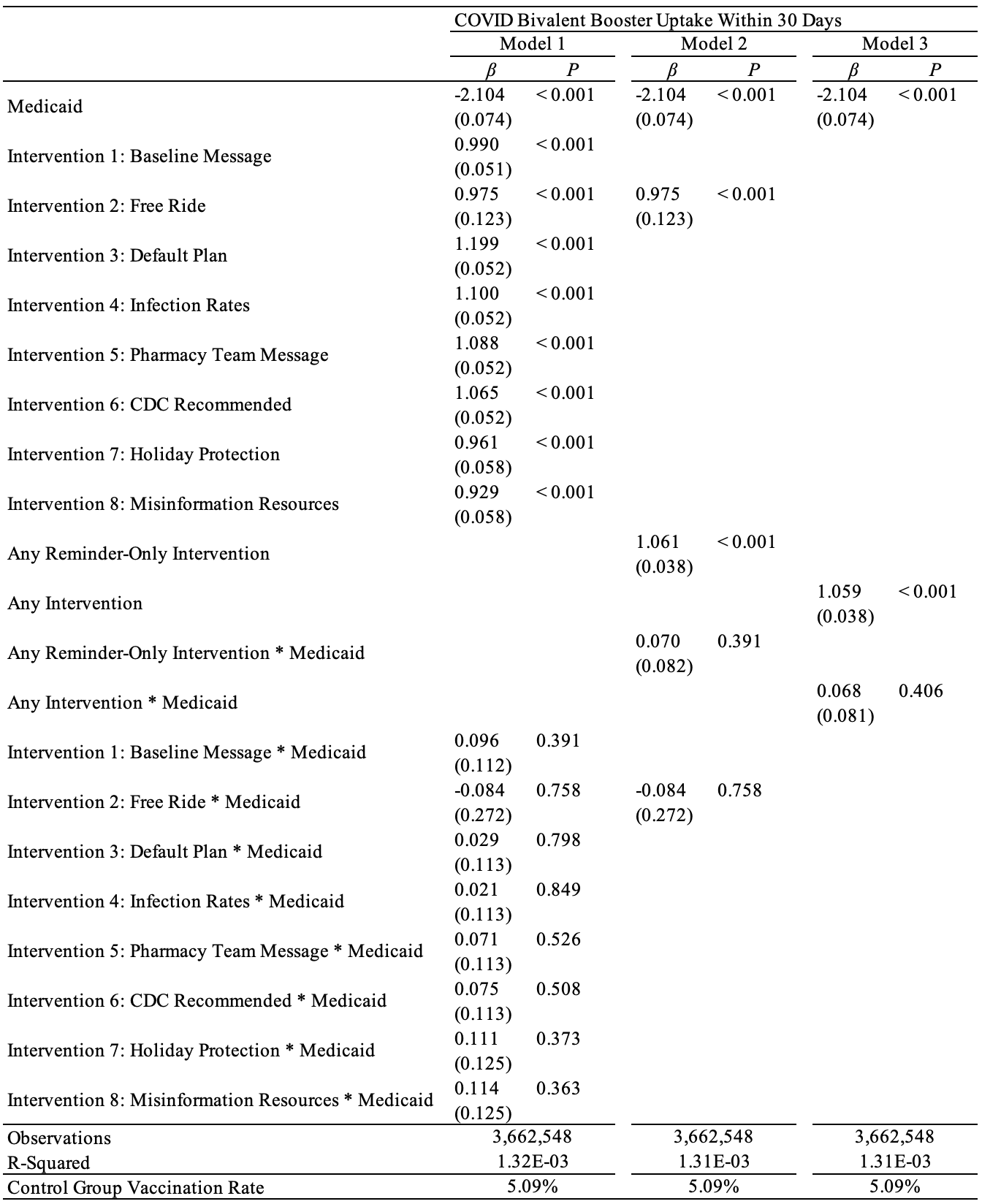
**

*Note:* This table reports the results of three ordinary least squares (OLS) regressions to predict whether a given patient received a COVID-19 booster vaccine at a CVS Pharmacy within 30 days of a patient’s study launch day. Model 1 relies on the same specification as our main regression model (Table 2, Model 1), while Model 2 and 3 include different primary predictors. In Model 2, we include two primary predictors: an indicator for whether a patient received any reminder-only intervention and an indicator for whether a patient received our free ride intervention. In Model 3, we include a single pooled treatment indicator for whether a patient received any of our megastudy’s eight intervention conditions. Each model includes an indicator for whether a patient has Medicaid coverage and interactions between this Medicaid coverage indicator and each intervention indicator. All three regression models also include indicators for whether the patient received their first text message on launch day 1 or launch day 2 (an indicator for receiving a message on launch day 3 is omitted). The control variables in all models are mean-centered using the mean of the holdout control. All regression coefficients and standard errors have been multiplied by 100 to improve interpretability (and thus reflect percentage point change(s) induced in vaccination uptake). Standard errors reported in parentheses are estimated robustly using HC1. Statistical tests of whether an individual regression coefficient is zero are all two-sided.

**Table S23. Heterogeneity analyses by whether a patient has unknown insurance.** Regression-estimated impact of each of our megastudy’s eight intervention conditions on bivalent COVID-19 booster uptake at a CVS Pharmacy within 30 days of a patient’s study launch day as a function of patient having unknown insurance coverage, either breaking out all interventions individually (Model 1), pooling the reminder-only interventions (Model 2), or pooling all interventions (Model 3).

**
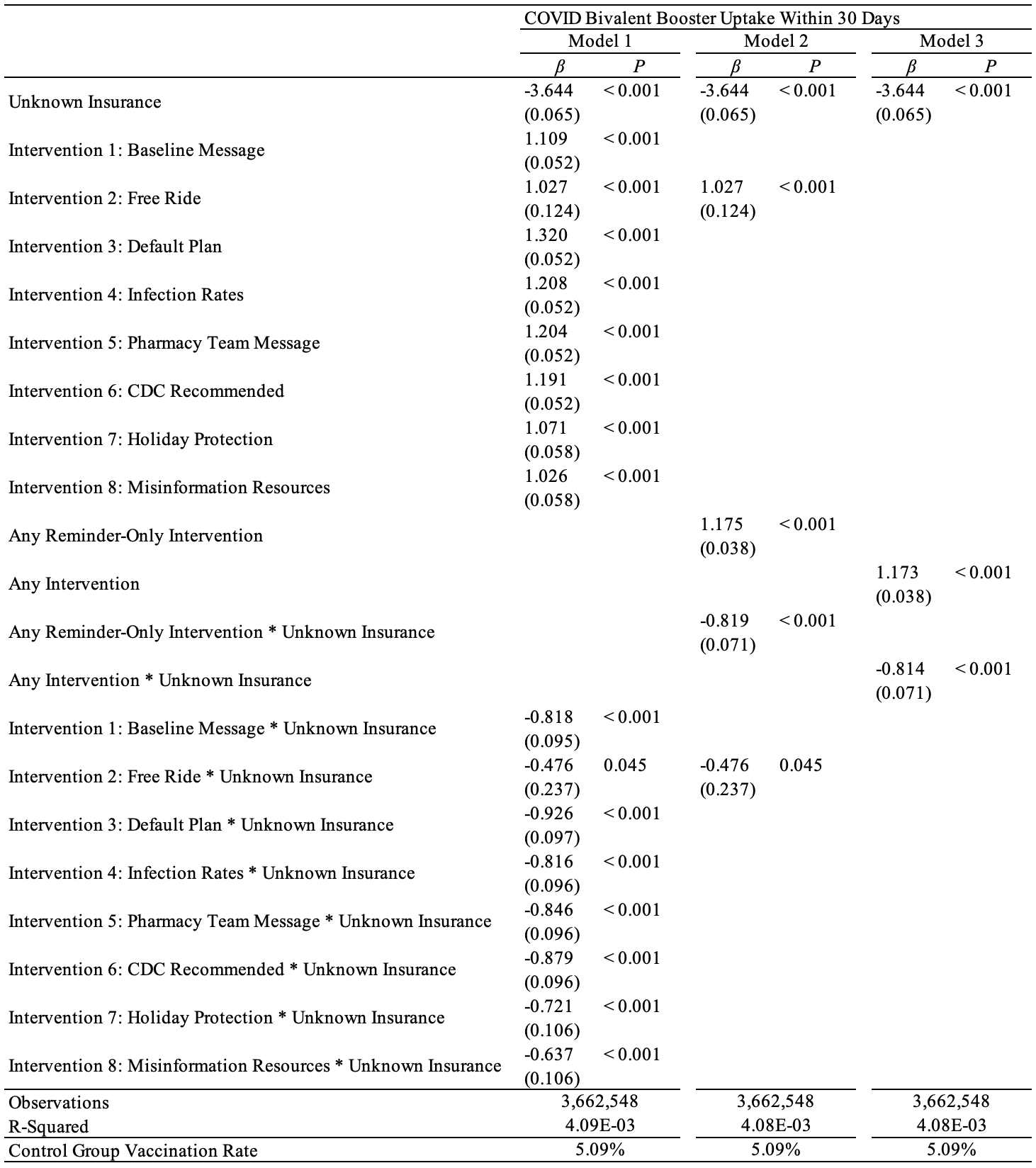
**

*Note:* This table reports the results of three ordinary least squares (OLS) regressions to predict whether a given patient received a COVID-19 booster vaccine at a CVS Pharmacy within 30 days of a patient’s study launch day. Model 1 relies on the same specification as our main regression model (Table 2, Model 1), while Model 2 and 3 include different primary predictors. In Model 2, we include two primary predictors: an indicator for whether a patient received any reminder-only intervention and an indicator for whether a patient received our free ride intervention. In Model 3, we include a single pooled treatment indicator for whether a patient received any of our megastudy’s eight intervention conditions. Each model includes an indicator for whether a patient has unknown insurance and interactions between this unknown insurance indicator and each intervention indicator. All three regression models also include indicators for whether the patient received their first text message on launch day 1 or launch day 2 (an indicator for receiving a message on launch day 3 is omitted). The control variables in all models are mean-centered using the mean of the holdout control. All regression coefficients and standard errors have been multiplied by 100 to improve interpretability (and thus reflect percentage point change(s) induced in vaccination uptake). Standard errors reported in parentheses are estimated robustly using HC1. Statistical tests of whether an individual regression coefficient is zero are all two-sided.

**Table S24. Heterogeneity analyses by whether a patient has commercial insurance.** Regression-estimated impact of each of our megastudy’s eight intervention conditions on bivalent COVID-19 booster uptake at a CVS Pharmacy within 30 days of a patient’s study launch day as a function of patient having commercial insurance coverage, either breaking out all interventions individually (Model 1), pooling the reminder-only interventions (Model 2), or pooling all interventions (Model 3).


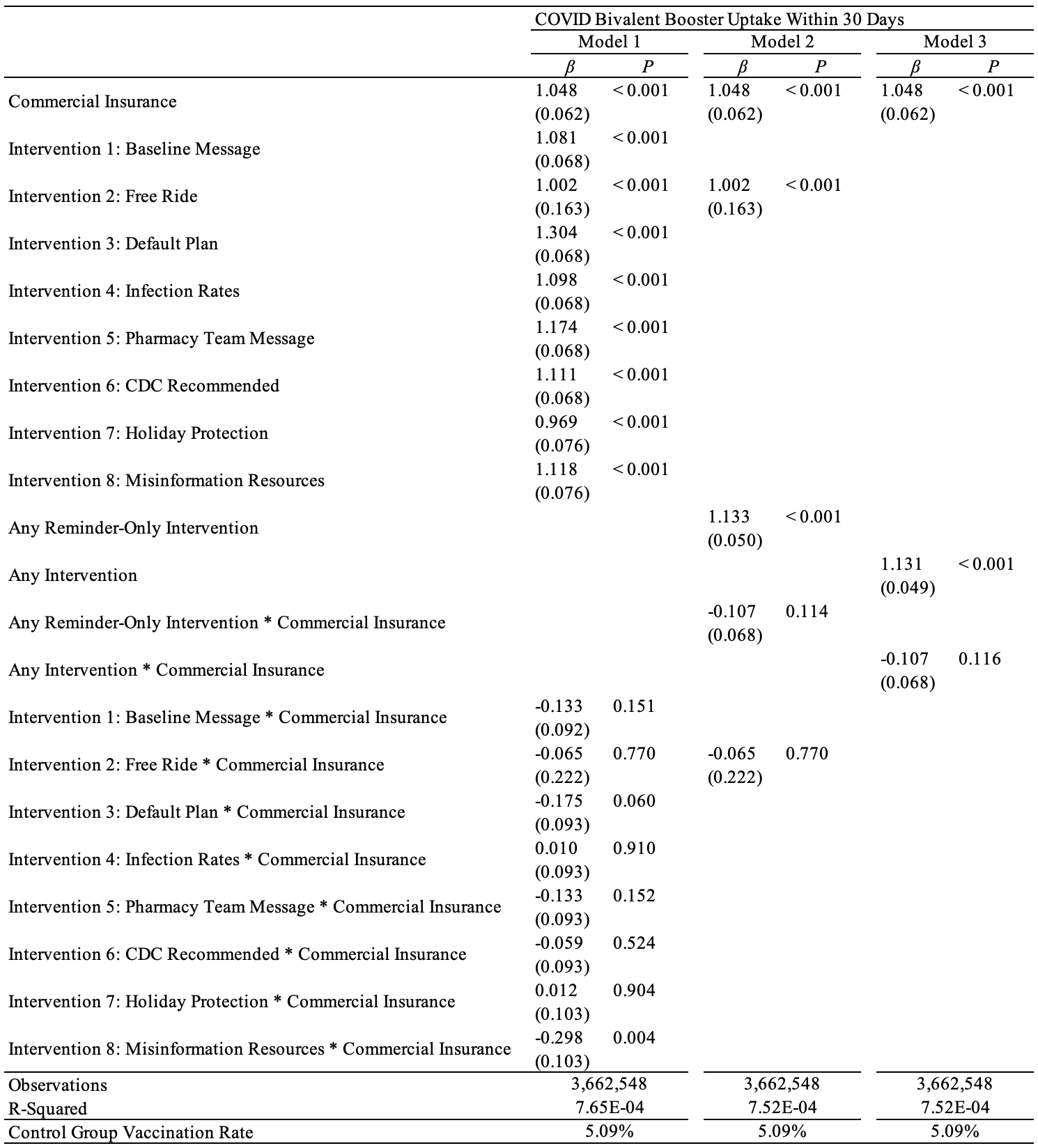


*Note:* This table reports the results of three ordinary least squares (OLS) regressions to predict whether a given patient received a COVID-19 booster vaccine at a CVS Pharmacy within 30 days of a patient’s study launch day. Model 1 relies on the same specification as our main regression model (Table 2, Model 1), while Model 2 and 3 include different primary predictors. In Model 2, we include two primary predictors: an indicator for whether a patient received any reminder-only intervention and an indicator for whether a patient received our free ride intervention. In Model 3, we include a single pooled treatment indicator for whether a patient received any of our megastudy’s eight intervention conditions. Each model includes an indicator for whether a patient has commercial insurance and interactions between this commercial insurance indicator and each intervention indicator. All three regression models also include indicators for whether the patient received their first text message on launch day 1 or launch day 2 (an indicator for receiving a message on launch day 3 is omitted). The control variables in all models are mean-centered using the mean of the holdout control. All regression coefficients and standard errors have been multiplied by 100 to improve interpretability (and thus reflect percentage point change(s) induced in vaccination uptake). Standard errors reported in parentheses are estimated robustly using HC1. Statistical tests of whether an individual regression coefficient is zero are all two-sided.

**SUBGROUP ANALYSES BASED ON PATIENT NEIGHBOURHOOD CHARACTERISTICS**

**Table S25. Subgroup analyses for patients whose closest CVS Pharmacy is in a zip code with a “low” level of White residents** (“low” levels are defined by a median split; observations with below median values in our data are included here; median percent of White residents = 62.09%). Regression-estimated impact of each of our megastudy’s eight intervention conditions on bivalent COVID-19 booster uptake at CVS Pharmacy within 30 days of a patient’s study launch day for patients whose closest CVS Pharmacy is in a zip code with a low level of White residents according to CVS Pharmacy, either breaking out all interventions individually (Model 1), pooling the reminder-only interventions (Model 2), or pooling all interventions (Model 3).


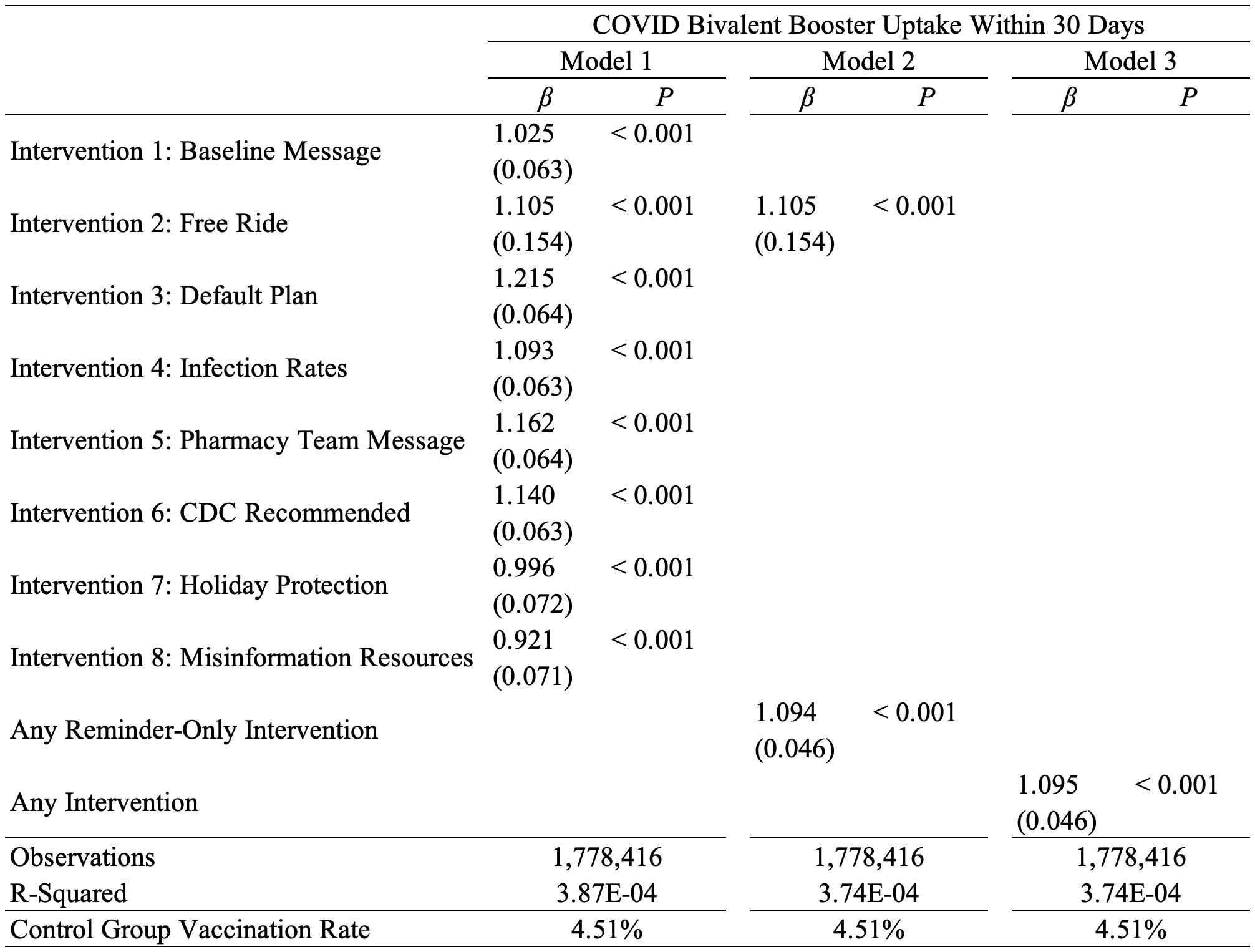


*Note:* This table reports the results of three ordinary least squares (OLS) regressions to predict whether a given patient whose closest CVS Pharmacy is in a zip code with a low level of White residents received a COVID-19 booster vaccine at a CVS Pharmacy within 30 days of a patient’s study launch day. Model 1 relies on the same specification as our main regression model (Table 2, Model 1). Models 2 and 3 include different primary predictors. In Model 2, we include two primary predictors: an indicator for whether a patient received any reminder-only intervention and an indicator for whether a patient received our free ride intervention. In Model 3, we include a single pooled treatment indicator for whether a patient received any of our megastudy’s eight intervention conditions. All three regression models include indicators for whether the patient received their first text message on launch day 1 or launch day 2 (an indicator for receiving a message on launch day 3 is omitted). The control variables in all models are mean-centered using the mean of the holdout control. All regression coefficients and standard errors have been multiplied by 100 to improve interpretability (and thus reflect percentage point change(s) induced in vaccination uptake). Standard errors reported in parentheses are estimated robustly using HC1. Statistical tests of whether an individual regression coefficient is zero are all two-sided.

**Table S26. Subgroup analyses for patients whose closest CVS Pharmacy is in a zip code with a “high” level of White residents** (“high” levels are defined by a median split; observations with at or above median values in our data are included here; median percent of White residents = 62.09%). Regression-estimated impact of each of our megastudy’s eight intervention conditions on bivalent COVID-19 booster uptake at CVS Pharmacy within 30 days of a patient’s study launch day for patients whose closest CVS Pharmacy is in a zip code with a high level of White residents according to CVS Pharmacy, either breaking out all interventions individually (Model 1), pooling the reminder-only interventions (Model 2), or pooling all interventions (Model 3).


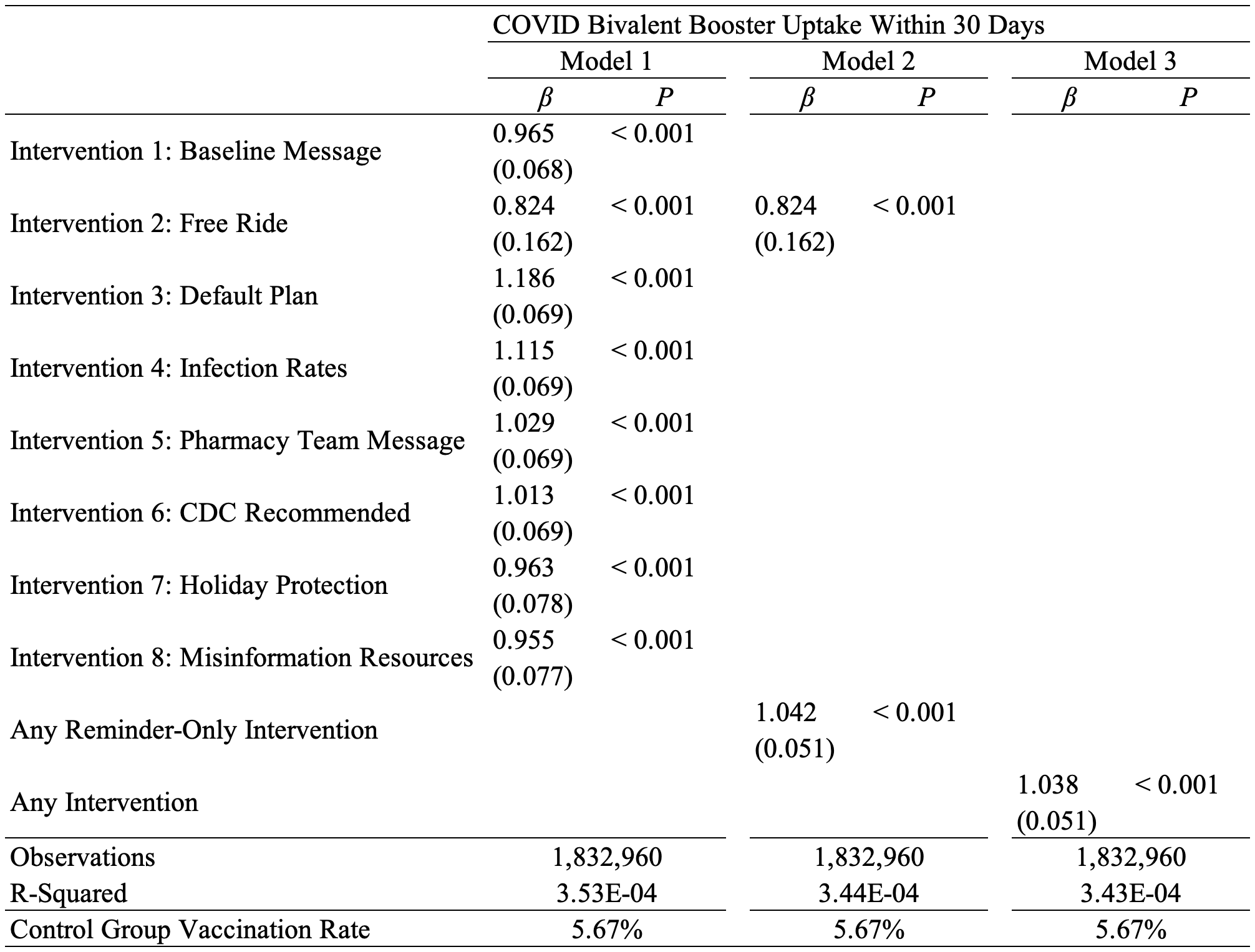


*Note:* This table reports the results of three ordinary least squares (OLS) regressions to predict whether a given patient whose closest CVS Pharmacy is in a zip code with a high level of White residents received a COVID-19 booster vaccine at a CVS Pharmacy within 30 days of a patient’s study launch day. Model 1 relies on the same specification as our main regression model (Table 2, Model 1). Models 2 and 3 include different primary predictors. In Model 2, we include two primary predictors: an indicator for whether a patient received any reminder-only intervention and an indicator for whether a patient received our free ride intervention. In Model 3, we include a single pooled treatment indicator for whether a patient received any of our megastudy’s eight intervention conditions. All three regression models include indicators for whether the patient received their first text message on launch day 1 or launch day 2 (an indicator for receiving a message on launch day 3 is omitted). The control variables in all models are mean-centered using the mean of the holdout control. All regression coefficients and standard errors have been multiplied by 100 to improve interpretability (and thus reflect percentage point change(s) induced in vaccination uptake). Standard errors reported in parentheses are estimated robustly using HC1. Statistical tests of whether an individual regression coefficient is zero are all two-sided.

**Table S27. Subgroup analyses for patients whose closest CVS Pharmacy is in a zip code with a “low” level of Black residents** (“low” levels are defined by a median split; observations with below median values in our data are included here; median percent of Black residents = 5.53%). Regression-estimated impact of each of our megastudy’s eight intervention conditions on bivalent COVID-19 booster uptake at CVS Pharmacy within 30 days of a patient’s study launch day for patients whose closest CVS Pharmacy is in a zip code with a low level of Black residents according to CVS Pharmacy, either breaking out all interventions individually (Model 1), pooling the reminder-only interventions (Model 2), or pooling all interventions (Model 3).


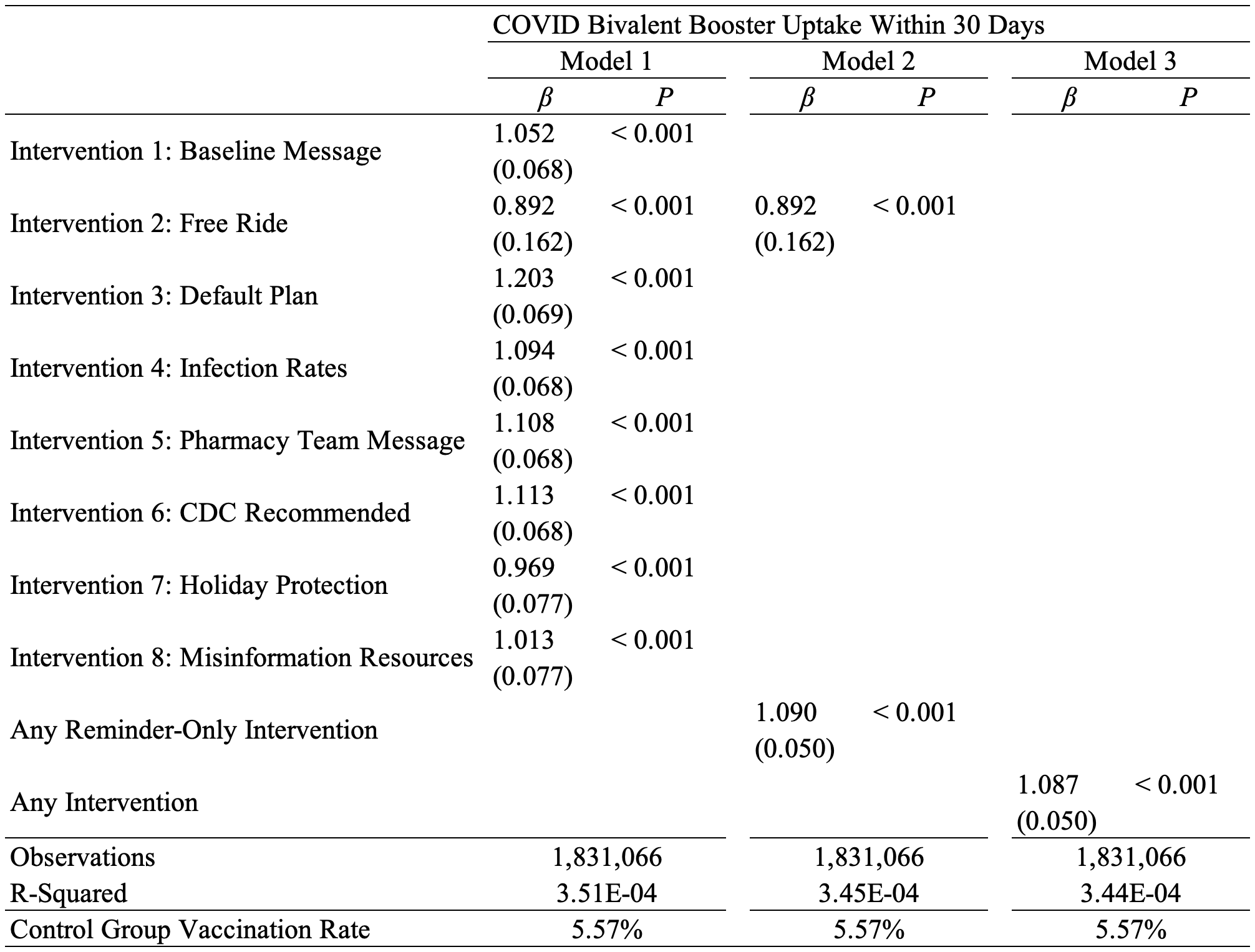


*Note:* This table reports the results of three ordinary least squares (OLS) regressions to predict whether a given patient whose closest CVS Pharmacy is in a zip code with a low level of Black residents received a COVID-19 booster vaccine at a CVS Pharmacy within 30 days of a patient’s study launch day. Model 1 relies on the same specification as our main regression model (Table 2, Model 1). Models 2 and 3 include different primary predictors. In Model 2, we include two primary predictors: an indicator for whether a patient received any reminder-only intervention and an indicator for whether a patient received our free ride intervention. In Model 3, we include a single pooled treatment indicator for whether a patient received any of our megastudy’s eight intervention conditions. All three regression models include indicators for whether the patient received their first text message on launch day 1 or launch day 2 (an indicator for receiving a message on launch day 3 is omitted). The control variables in all models are mean-centered using the mean of the holdout control. All regression coefficients and standard errors have been multiplied by 100 to improve interpretability (and thus reflect percentage point change(s) induced in vaccination uptake). Standard errors reported in parentheses are estimated robustly using HC1. Statistical tests of whether an individual regression coefficient is zero are all two-sided.

**Table S28. Subgroup analyses for patients whose closest CVS Pharmacy is in a zip code with a “high” level of Black residents** (“high” levels are defined by a median split; observations with at or above median values in our data are included here; median percent of Black residents = 5.53%). Regression-estimated impact of each of our megastudy’s eight intervention conditions on bivalent COVID-19 booster uptake at CVS Pharmacy within 30 days of a patient’s study launch day for patients whose closest CVS Pharmacy is in a zip code with a high level of Black residents according to CVS Pharmacy, either breaking out all interventions individually (Model 1), pooling the reminder-only interventions (Model 2), or pooling all interventions (Model 3).


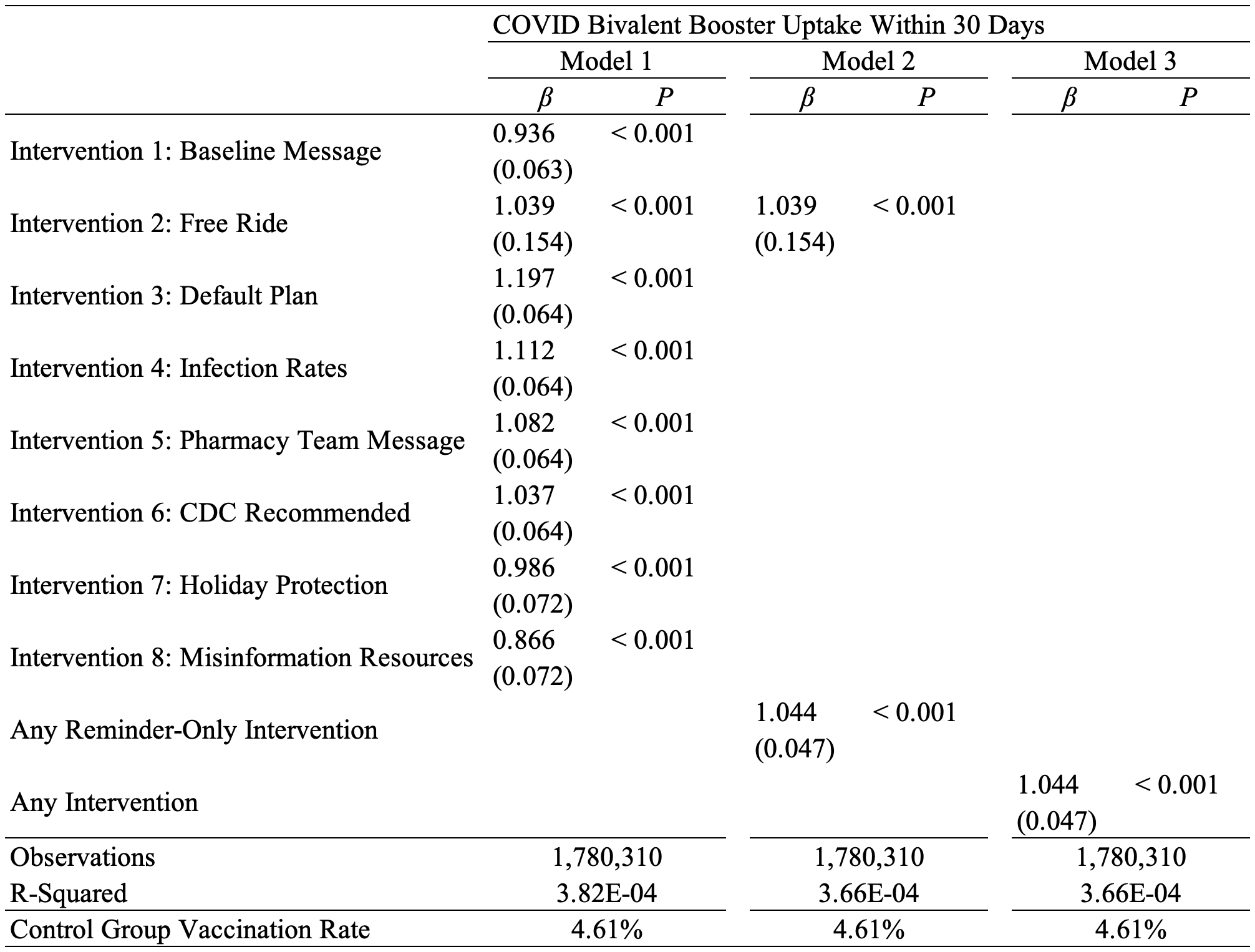


*Note:* This table reports the results of three ordinary least squares (OLS) regressions to predict whether a given patient whose closest CVS Pharmacy is in a zip code with a high level of Black residents received a COVID-19 booster vaccine at a CVS Pharmacy within 30 days of a patient’s study launch day. Model 1 relies on the same specification as our main regression model (Table 2, Model 1). Models 2 and 3 include different primary predictors. In Model 2, we include two primary predictors: an indicator for whether a patient received any reminder-only intervention and an indicator for whether a patient received our free ride intervention. In Model 3, we include a single pooled treatment indicator for whether a patient received any of our megastudy’s eight intervention conditions. All three regression models include indicators for whether the patient received their first text message on launch day 1 or launch day 2 (an indicator for receiving a message on launch day 3 is omitted). The control variables in all models are mean-centered using the mean of the holdout control. All regression coefficients and standard errors have been multiplied by 100 to improve interpretability (and thus reflect percentage point change(s) induced in vaccination uptake). Standard errors reported in parentheses are estimated robustly using HC1. Statistical tests of whether an individual regression coefficient is zero are all two-sided.

**Table S29. Subgroup analyses for patients whose closest CVS Pharmacy is in a zip code with a “low” level of Asian residents** (“low” levels are defined by a median split; observations with below median values in our data are included here; median percent of Asian residents = 4.59%). Regression-estimated impact of each of our megastudy’s eight intervention conditions on bivalent COVID-19 booster uptake at CVS Pharmacy within 30 days of a patient’s study launch day for patients whose closest CVS Pharmacy is in a zip code with a low level of Asian residents according to CVS Pharmacy, either breaking out all interventions individually (Model 1), pooling the reminder-only interventions (Model 2), or pooling all interventions (Model 3).


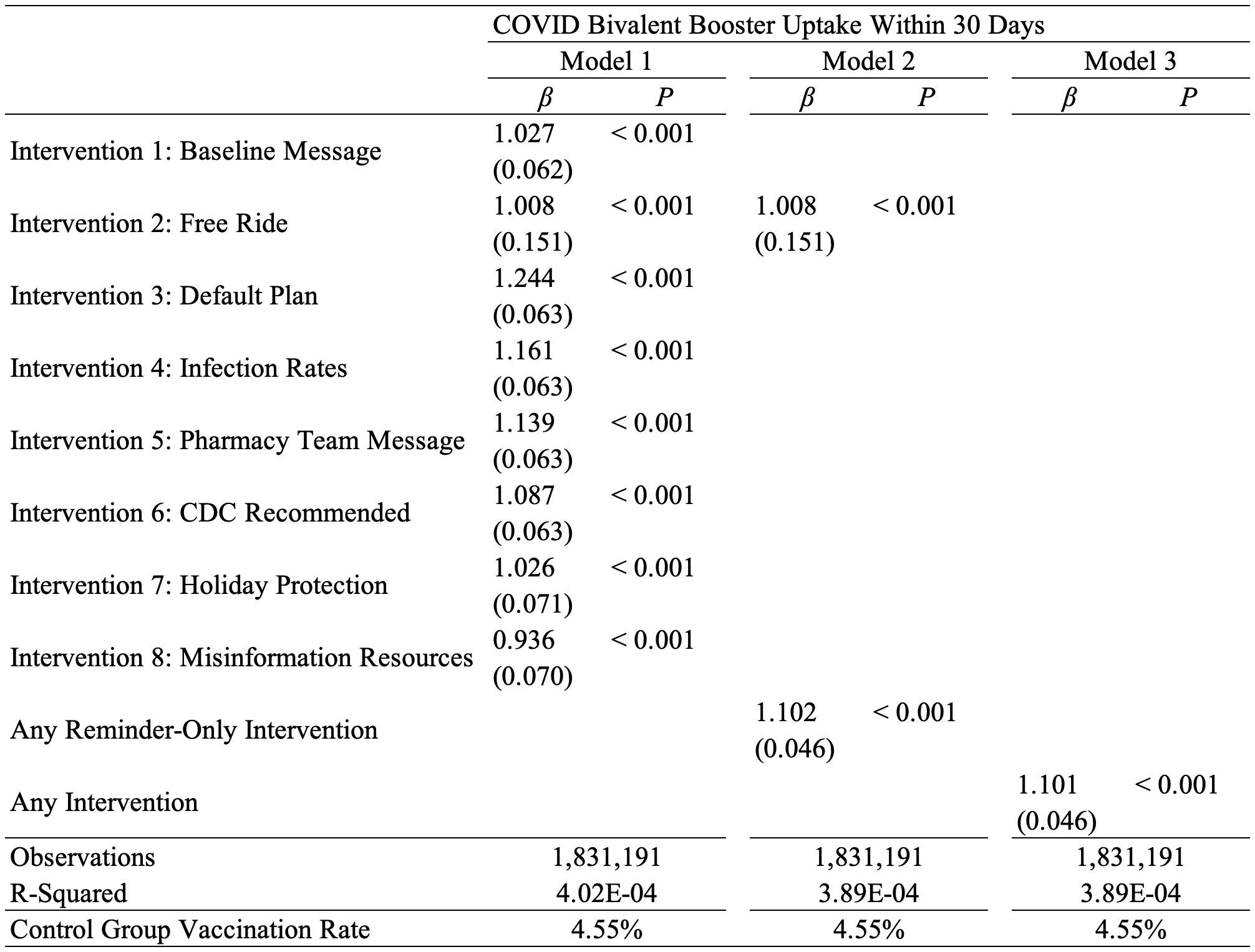


*Note:* This table reports the results of three ordinary least squares (OLS) regressions to predict whether a given patient whose closest CVS Pharmacy is in a zip code with a low level of Asian residents received a COVID-19 booster vaccine at a CVS Pharmacy within 30 days of a patient’s study launch day. Model 1 relies on the same specification as our main regression model (Table 2, Model 1). Models 2 and 3 include different primary predictors. In Model 2, we include two primary predictors: an indicator for whether a patient received any reminder-only intervention and an indicator for whether a patient received our free ride intervention. In Model 3, we include a single pooled treatment indicator for whether a patient received any of our megastudy’s eight intervention conditions. All three regression models include indicators for whether the patient received their first text message on launch day 1 or launch day 2 (an indicator for receiving a message on launch day 3 is omitted). The control variables in all models are mean-centered using the mean of the holdout control. All regression coefficients and standard errors have been multiplied by 100 to improve interpretability (and thus reflect percentage point change(s) induced in vaccination uptake). Standard errors reported in parentheses are estimated robustly using HC1. Statistical tests of whether an individual regression coefficient is zero are all two-sided.

**Table S30. Subgroup analyses for patients whose closest CVS Pharmacy is in a zip code with a “high” level of Asian residents** (“high” levels are defined by a median split; observations with at or above median values in our data are included here; median percent of Asian residents = 4.59%). Regression-estimated impact of each of our megastudy’s eight intervention conditions on bivalent COVID-19 booster uptake at CVS Pharmacy within 30 days of a patient’s study launch day for patients whose closest CVS Pharmacy is in a zip code with a high level of Asian residents according to CVS Pharmacy, either breaking out all interventions individually (Model 1), pooling the reminder-only interventions (Model 2), or pooling all interventions (Model 3).


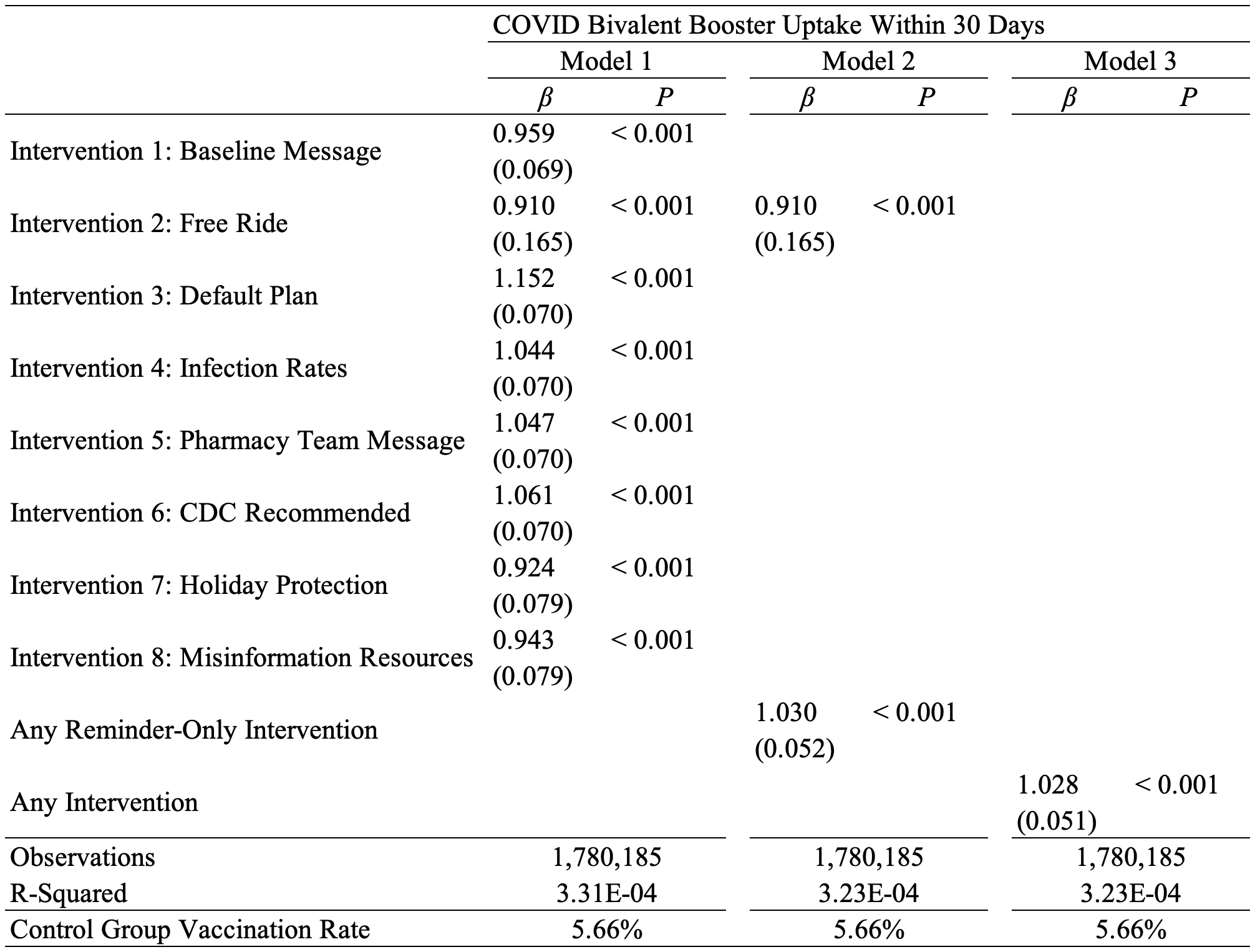


*Note:* This table reports the results of three ordinary least squares (OLS) regressions to predict whether a given patient whose closest CVS Pharmacy is in a zip code with a high level of Asian residents received a COVID-19 booster vaccine at a CVS Pharmacy within 30 days of a patient’s study launch day. Model 1 relies on the same specification as our main regression model (Table 2, Model 1). Models 2 and 3 include different primary predictors. In Model 2, we include two primary predictors: an indicator for whether a patient received any reminder-only intervention and an indicator for whether a patient received our free ride intervention. In Model 3, we include a single pooled treatment indicator for whether a patient received any of our megastudy’s eight intervention conditions. All three regression models include indicators for whether the patient received their first text message on launch day 1 or launch day 2 (an indicator for receiving a message on launch day 3 is omitted). The control variables in all models are mean-centered using the mean of the holdout control. All regression coefficients and standard errors have been multiplied by 100 to improve interpretability (and thus reflect percentage point change(s) induced in vaccination uptake). Standard errors reported in parentheses are estimated robustly using HC1. Statistical tests of whether an individual regression coefficient is zero are all two-sided.

**Table S31. Subgroup analyses for patients whose closest CVS Pharmacy is in a zip code with a “low” level of Hispanic residents** (“low” levels are defined by a median split; observations with below median values in our data are included here; median percent of Hispanic residents = 11.94%). Regression-estimated impact of each of our megastudy’s eight intervention conditions on bivalent COVID-19 booster uptake at CVS Pharmacy within 30 days of a patient’s study launch day for patients whose closest CVS Pharmacy is in a zip code with a low level of Hispanic residents according to CVS Pharmacy, either breaking out all interventions individually (Model 1), pooling the reminder-only interventions (Model 2), or pooling all interventions (Model 3).


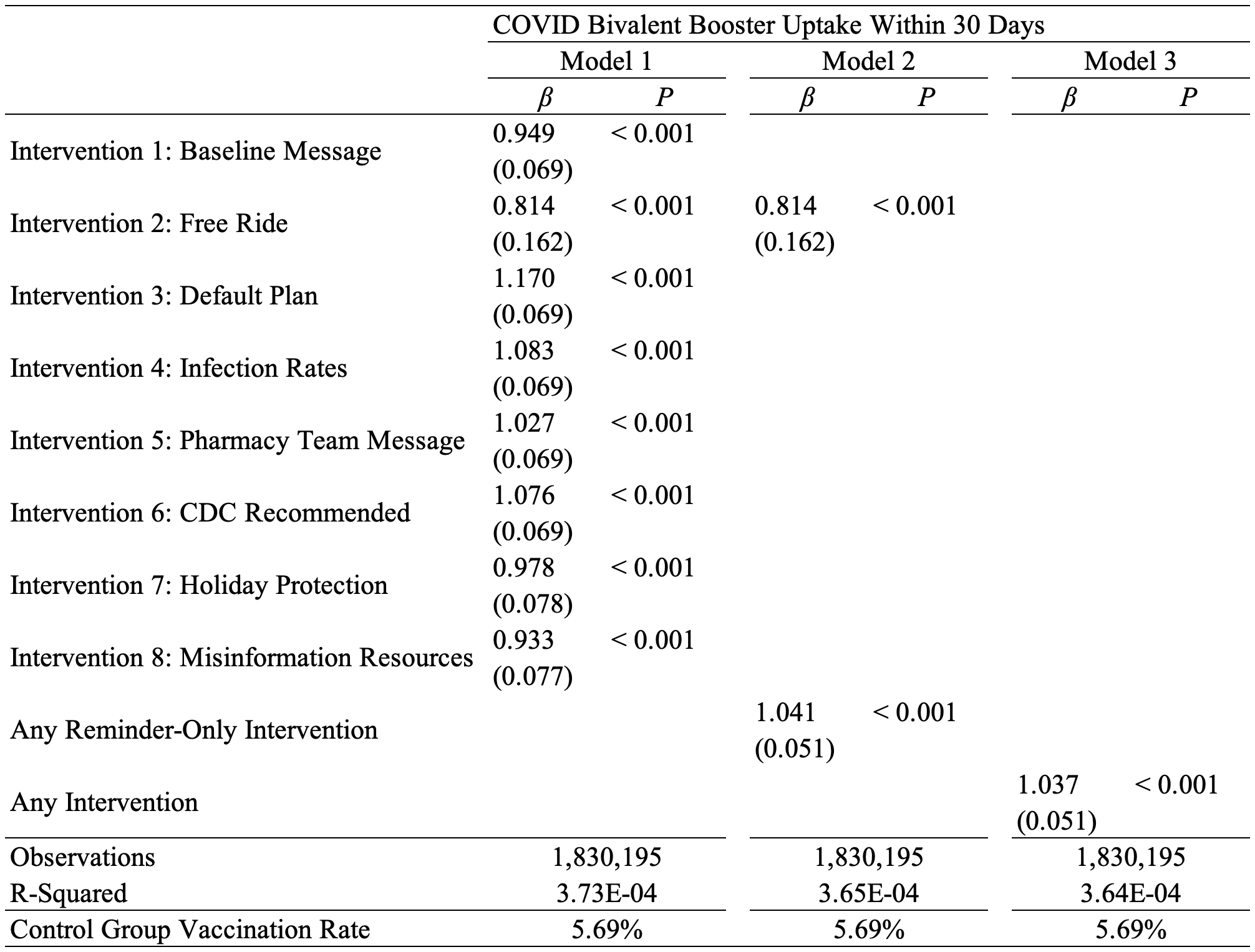


*Note:* This table reports the results of three ordinary least squares (OLS) regressions to predict whether a given patient whose closest CVS Pharmacy is in a zip code with a low level of Hispanic residents received a COVID-19 booster vaccine at a CVS Pharmacy within 30 days of a patient’s study launch day. Model 1 relies on the same specification as our main regression model (Table 2, Model 1). Models 2 and 3 include different primary predictors. In Model 2, we include two primary predictors: an indicator for whether a patient received any reminder-only intervention and an indicator for whether a patient received our free ride intervention. In Model 3, we include a single pooled treatment indicator for whether a patient received any of our megastudy’s eight intervention conditions. All three regression models include indicators for whether the patient received their first text message on launch day 1 or launch day 2 (an indicator for receiving a message on launch day 3 is omitted). The control variables in all models are mean-centered using the mean of the holdout control. All regression coefficients and standard errors have been multiplied by 100 to improve interpretability (and thus reflect percentage point change(s) induced in vaccination uptake). Standard errors reported in parentheses are estimated robustly using HC1. Statistical tests of whether an individual regression coefficient is zero are all two-sided.

**Table S32. Subgroup analyses for patients whose closest CVS Pharmacy is in a zip code with a “high” level of Hispanic residents** (“high” levels are defined by a median split; observations with at or above median values in our data are included here; median percent of Hispanic residents = 11.94%). Regression-estimated impact of each of our megastudy’s eight intervention conditions on bivalent COVID-19 booster uptake at CVS Pharmacy within 30 days of a patient’s study launch day for patients whose closest CVS Pharmacy is in a zip code with a high level of Hispanic residents according to CVS Pharmacy, either breaking out all interventions individually (Model 1), pooling the reminder-only interventions (Model 2), or pooling all interventions (Model 3).


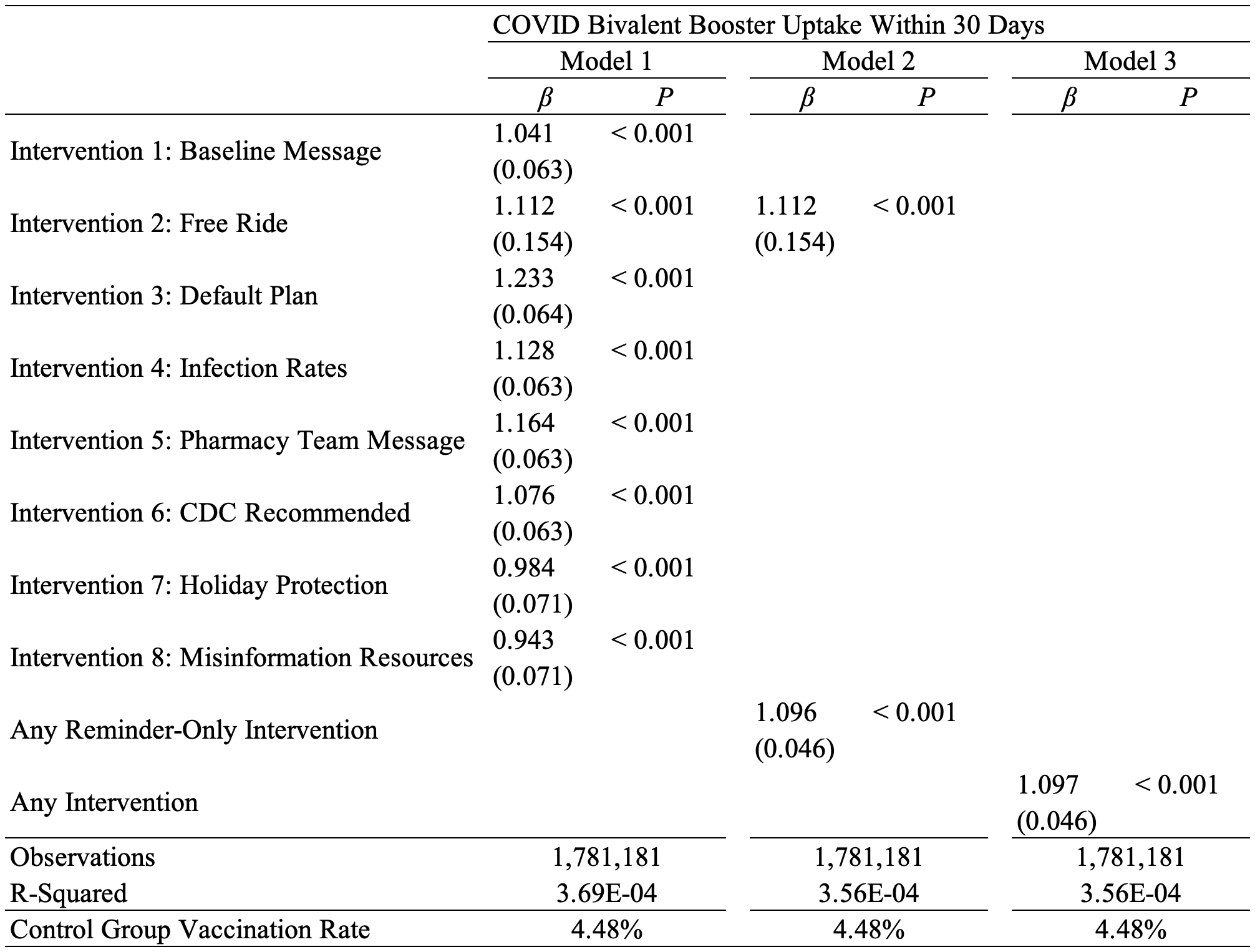


*Note:* This table reports the results of three ordinary least squares (OLS) regressions to predict whether a given patient whose closest CVS Pharmacy is in a zip code with a high level of Hispanic residents received a COVID-19 booster vaccine at a CVS Pharmacy within 30 days of a patient’s study launch day. Model 1 relies on the same specification as our main regression model (Table 2, Model 1). Models 2 and 3 include different primary predictors. In Model 2, we include two primary predictors: an indicator for whether a patient received any reminder-only intervention and an indicator for whether a patient received our free ride intervention. In Model 3, we include a single pooled treatment indicator for whether a patient received any of our megastudy’s eight intervention conditions. All three regression models include indicators for whether the patient received their first text message on launch day 1 or launch day 2 (an indicator for receiving a message on launch day 3 is omitted). The control variables in all models are mean-centered using the mean of the holdout control. All regression coefficients and standard errors have been multiplied by 100 to improve interpretability (and thus reflect percentage point change(s) induced in vaccination uptake). Standard errors reported in parentheses are estimated robustly using HC1. Statistical tests of whether an individual regression coefficient is zero are all two-sided.

**Table S33. Subgroup analyses for patients whose closest CVS Pharmacy is in a zip code with a “low” population density (residents per square mile)** (“low” densities are defined by a median split; observations with below median values in our data are included here; median population density = 3,006.93 residents per square mile). Regression-estimated impact of each of our megastudy’s eight intervention conditions on bivalent COVID-19 booster uptake at CVS Pharmacy within 30 days of a patient’s study launch day for patients whose closest CVS Pharmacy is in a zip code with a low density of residents per square mile according to the U.S. Census, either breaking out all interventions individually (Model 1), pooling the reminder-only interventions (Model 2), or pooling all interventions (Model 3).


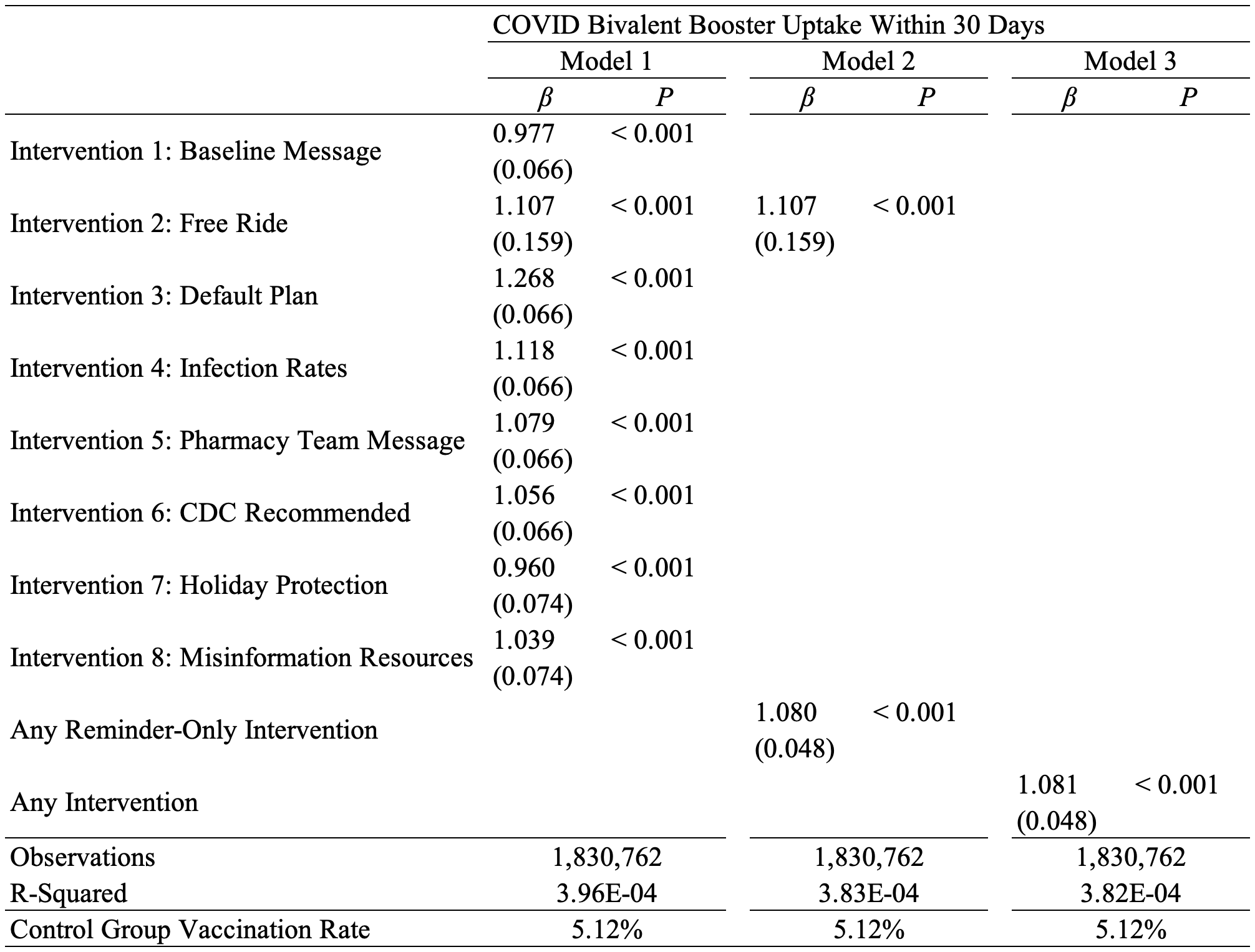


*Note:* This table reports the results of three ordinary least squares (OLS) regressions to predict whether a given patient whose closest CVS Pharmacy is in a zip code with a low density of residents per square mile received a COVID-19 booster vaccine at a CVS Pharmacy within 30 days of a patient’s study launch day. Model 1 relies on the same specification as our main regression model (Table 2, Model 1). Models 2 and 3 include different primary predictors. In Model 2, we include two primary predictors: an indicator for whether a patient received any reminder-only intervention and an indicator for whether a patient received our free ride intervention. In Model 3, we include a single pooled treatment indicator for whether a patient received any of our megastudy’s eight intervention conditions. All three regression models include indicators for whether the patient received their first text message on launch day 1 or launch day 2 (an indicator for receiving a message on launch day 3 is omitted). The control variables in all models are mean-centered using the mean of the holdout control. All regression coefficients and standard errors have been multiplied by 100 to improve interpretability (and thus reflect percentage point change(s) induced in vaccination uptake). Standard errors reported in parentheses are estimated robustly using HC1. Statistical tests of whether an individual regression coefficient is zero are all two-sided.

**Table S34. Subgroup analyses for patients whose closest CVS Pharmacy is in a zip code with a “high” population density (residents per square mile)** (“high” densities are defined by a median split; observations with at or above median values in our data are included here; median population density = 3,006.93 residents per square mile). Regression-estimated impact of each of our megastudy’s eight intervention conditions on bivalent COVID-19 booster uptake at CVS Pharmacy within 30 days of a patient’s study launch day for patients whose closest CVS Pharmacy is in a zip code with a high density of residents per square mile according to the U.S. Census, either breaking out all interventions individually (Model 1), pooling the reminder-only interventions (Model 2), or pooling all interventions (Model 3).


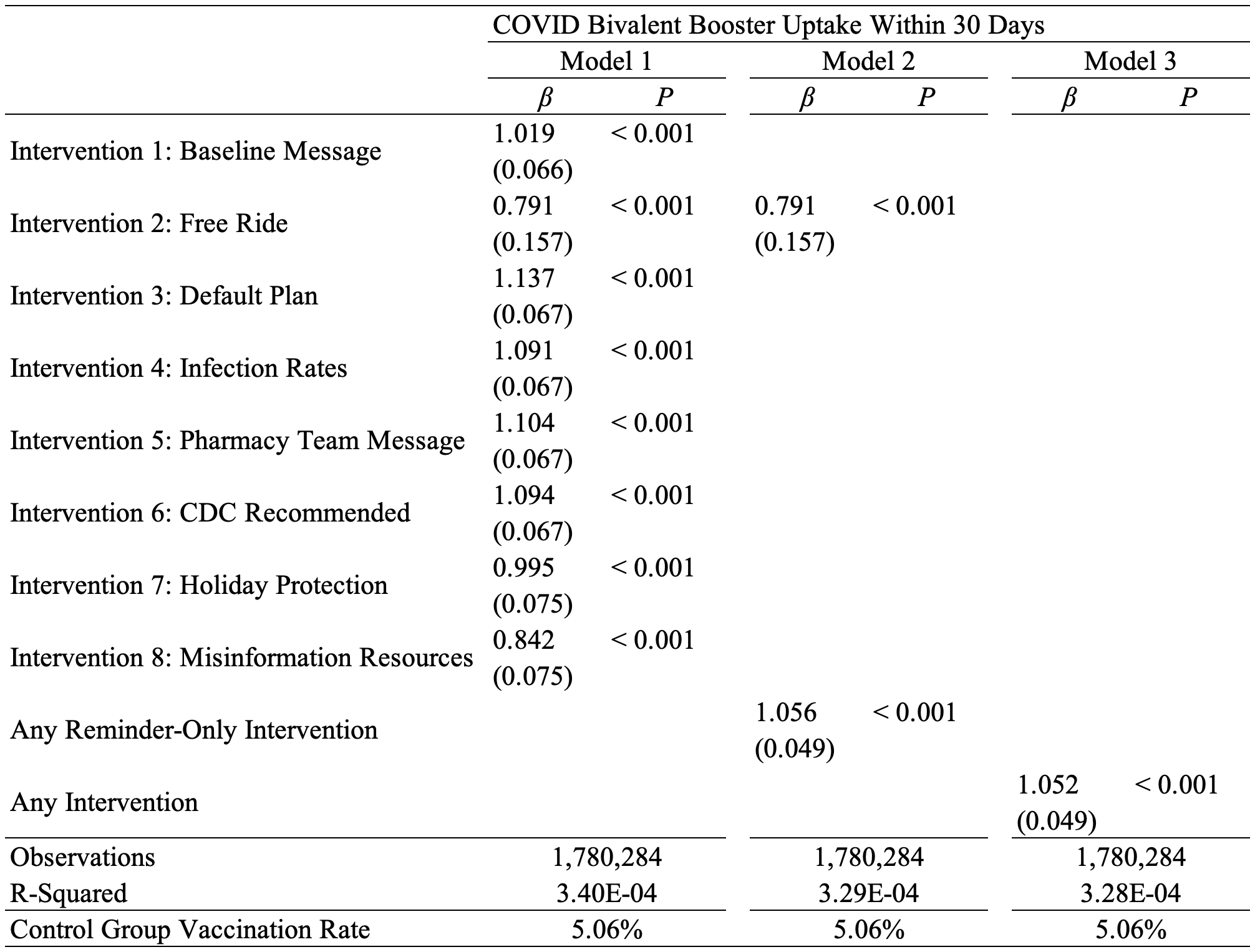


*Note:* This table reports the results of three ordinary least squares (OLS) regressions to predict whether a given patient whose closest CVS Pharmacy is in a zip code with a high density of residents per square mile received a COVID-19 booster vaccine at a CVS Pharmacy within 30 days of a patient’s study launch day. Model 1 relies on the same specification as our main regression model (Table 2, Model 1). Models 2 and 3 include different primary predictors. In Model 2, we include two primary predictors: an indicator for whether a patient received any reminder-only intervention and an indicator for whether a patient received our free ride intervention. In Model 3, we include a single pooled treatment indicator for whether a patient received any of our megastudy’s eight intervention conditions. All three regression models include indicators for whether the patient received their first text message on launch day 1 or launch day 2 (an indicator for receiving a message on launch day 3 is omitted). The control variables in all models are mean-centered using the mean of the holdout control. All regression coefficients and standard errors have been multiplied by 100 to improve interpretability (and thus reflect percentage point change(s) induced in vaccination uptake). Standard errors reported in parentheses are estimated robustly using HC1. Statistical tests of whether an individual regression coefficient is zero are all two-sided.

**Table S35. Subgroup analyses for patients whose closest CVS Pharmacy is in a zip code with a “low” level of median income** (“low” levels are defined by a median split; observations with below median values in our data are included here; median average income = $80,474). Regression-estimated impact of each of our megastudy’s eight intervention conditions on bivalent COVID-19 booster uptake at CVS Pharmacy within 30 days of a patient’s study launch day for patients whose closest CVS Pharmacy is in a zip code with a low level of average income according to the U.S. Census, either breaking out all interventions individually (Model 1), pooling the reminder-only interventions (Model 2), or pooling all interventions (Model 3).


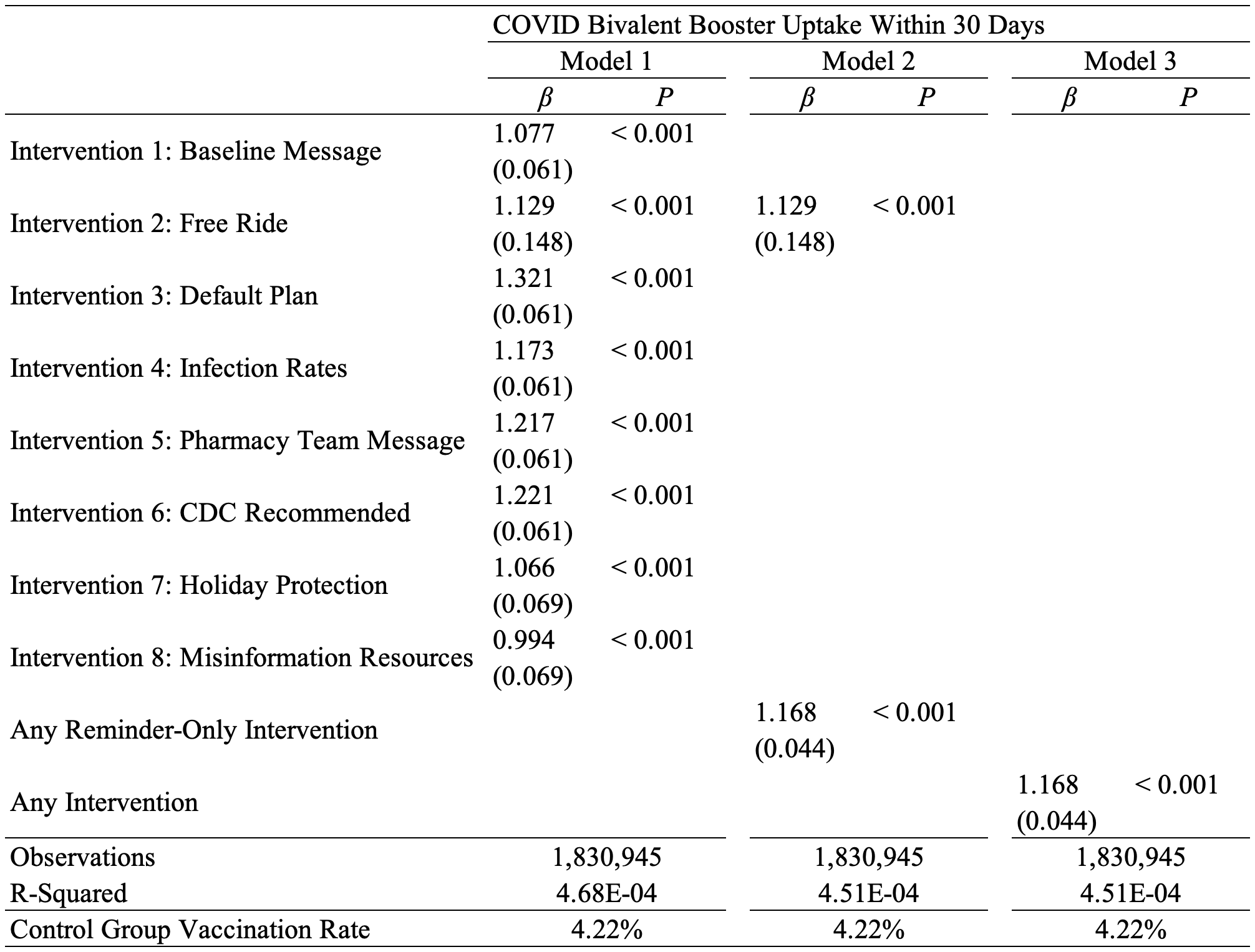


*Note:* This table reports the results of three ordinary least squares (OLS) regressions to predict whether a given patient whose closest CVS Pharmacy is in a zip code with a low level of average income received a COVID-19 booster vaccine at a CVS Pharmacy within 30 days of a patient’s study launch day. Model 1 relies on the same specification as our main regression model (Table 2, Model 1). Models 2 and 3 include different primary predictors. In Model 2, we include two primary predictors: an indicator for whether a patient received any reminder-only intervention and an indicator for whether a patient received our free ride intervention. In Model 3, we include a single pooled treatment indicator for whether a patient received any of our megastudy’s eight intervention conditions. All three regression models include indicators for whether the patient received their first text message on launch day 1 or launch day 2 (an indicator for receiving a message on launch day 3 is omitted). The control variables in all models are mean-centered using the mean of the holdout control. All regression coefficients and standard errors have been multiplied by 100 to improve interpretability (and thus reflect percentage point change(s) induced in vaccination uptake). Standard errors reported in parentheses are estimated robustly using HC1. Statistical tests of whether an individual regression coefficient is zero are all two-sided.

**Table S36. Subgroup analyses for patients whose closest CVS Pharmacy is in a zip code with a “high” level of median income** (“high” levels are defined by a median split; observations with at or above median values in our data are included here; median average income = $80,474). Regression-estimated impact of each of our megastudy’s eight intervention conditions on bivalent COVID-19 booster uptake at CVS Pharmacy within 30 days of a patient’s study launch day for patients whose closest CVS Pharmacy is in a zip code with a high level of average income according to the U.S. Census, either breaking out all interventions individually (Model 1), pooling the reminder-only interventions (Model 2), or pooling all interventions (Model 3).


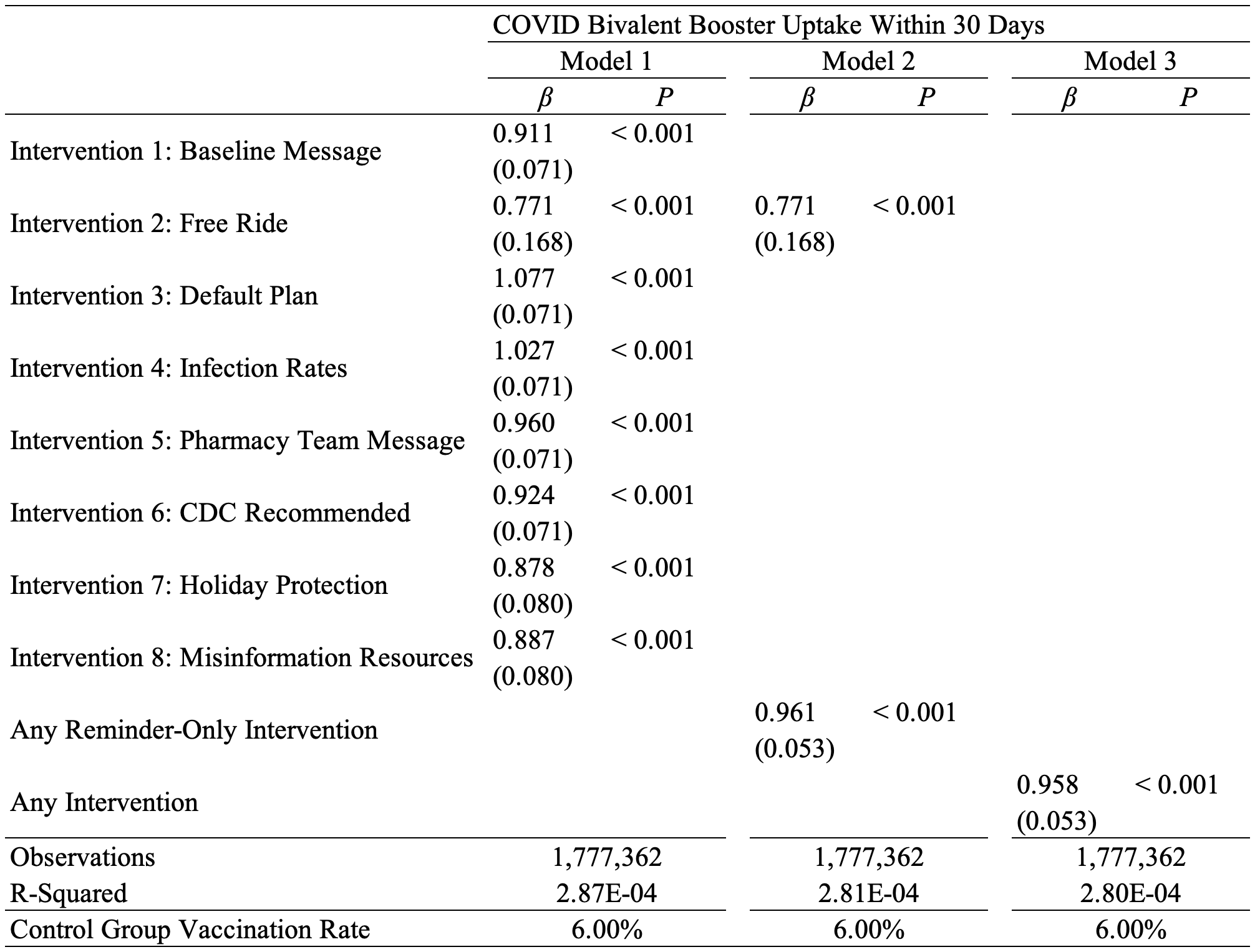


*Note:* This table reports the results of three ordinary least squares (OLS) regressions to predict whether a given patient whose closest CVS Pharmacy is in a zip code with a high level of average income received a COVID-19 booster vaccine at a CVS Pharmacy within 30 days of a patient’s study launch day. Model 1 relies on the same specification as our main regression model (Table 2, Model 1). Models 2 and 3 include different primary predictors. In Model 2, we include two primary predictors: an indicator for whether a patient received any reminder-only intervention and an indicator for whether a patient received our free ride intervention. In Model 3, we include a single pooled treatment indicator for whether a patient received any of our megastudy’s eight intervention conditions. All three regression models include indicators for whether the patient received their first text message on launch day 1 or launch day 2 (an indicator for receiving a message on launch day 3 is omitted). The control variables in all models are mean-centered using the mean of the holdout control. All regression coefficients and standard errors have been multiplied by 100 to improve interpretability (and thus reflect percentage point change(s) induced in vaccination uptake). Standard errors reported in parentheses are estimated robustly using HC1. Statistical tests of whether an individual regression coefficient is zero are all two-sided.

**Table S37. Subgroup analyses for patients whose closest CVS Pharmacy is in a zip code with a “low” level of residents with a Bachelor’s degree** (“low” levels are defined by a median split; observations with below median values in our data are included here; median percent of residents with a Bachelor’s degree = 29.08%). Regression-estimated impact of each of our megastudy’s eight intervention conditions on bivalent COVID-19 booster uptake at CVS Pharmacy within 30 days of a patient’s study launch day for patients whose closest CVS Pharmacy is in a zip code with a low level of residents with a Bachelor’s degree according to the U.S. Census, either breaking out all interventions individually (Model 1), pooling the reminder-only interventions (Model 2), or pooling all interventions (Model 3).


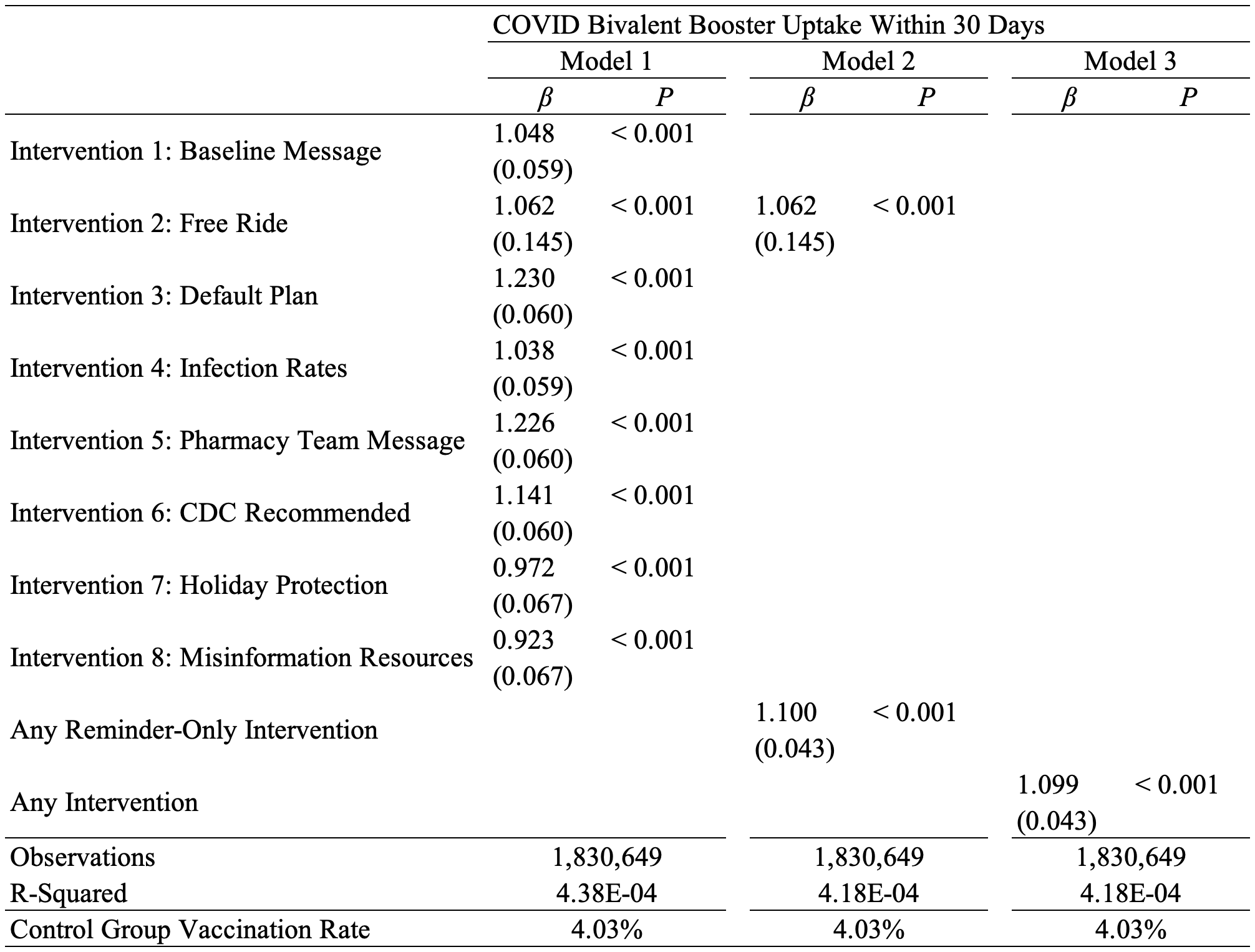


*Note:* This table reports the results of three ordinary least squares (OLS) regressions to predict whether a given patient whose closest CVS Pharmacy is in a zip code with a low level of residents with a Bachelor’s degree received a COVID-19 booster vaccine at a CVS Pharmacy within 30 days of a patient’s study launch day. Model 1 relies on the same specification as our main regression model (Table 2, Model 1). Models 2 and 3 include different primary predictors. In Model 2, we include two primary predictors: an indicator for whether a patient received any reminder-only intervention and an indicator for whether a patient received our free ride intervention. In Model 3, we include a single pooled treatment indicator for whether a patient received any of our megastudy’s eight intervention conditions. All three regression models include indicators for whether the patient received their first text message on launch day 1 or launch day 2 (an indicator for receiving a message on launch day 3 is omitted). The control variables in all models are mean-centered using the mean of the holdout control. All regression coefficients and standard errors have been multiplied by 100 to improve interpretability (and thus reflect percentage point change(s) induced in vaccination uptake). Standard errors reported in parentheses are estimated robustly using HC1. Statistical tests of whether an individual regression coefficient is zero are all two-sided.

**Table S38. Subgroup analyses for patients whose closest CVS Pharmacy is in a zip code with a “high” level of residents with a Bachelor’s degree** (“high” levels are defined by a median split; observations with at or above median values in our data are included here; median percent of residents with a Bachelor’s degree = 29.08%). Regression-estimated impact of each of our megastudy’s eight intervention conditions on bivalent COVID-19 booster uptake at CVS Pharmacy within 30 days of a patient’s study launch day for patients whose closest CVS Pharmacy is in a zip code with a high level of residents with a Bachelor’s degree according to the U.S. Census, either breaking out all interventions individually (Model 1), pooling the reminder-only interventions (Model 2), or pooling all interventions (Model 3).


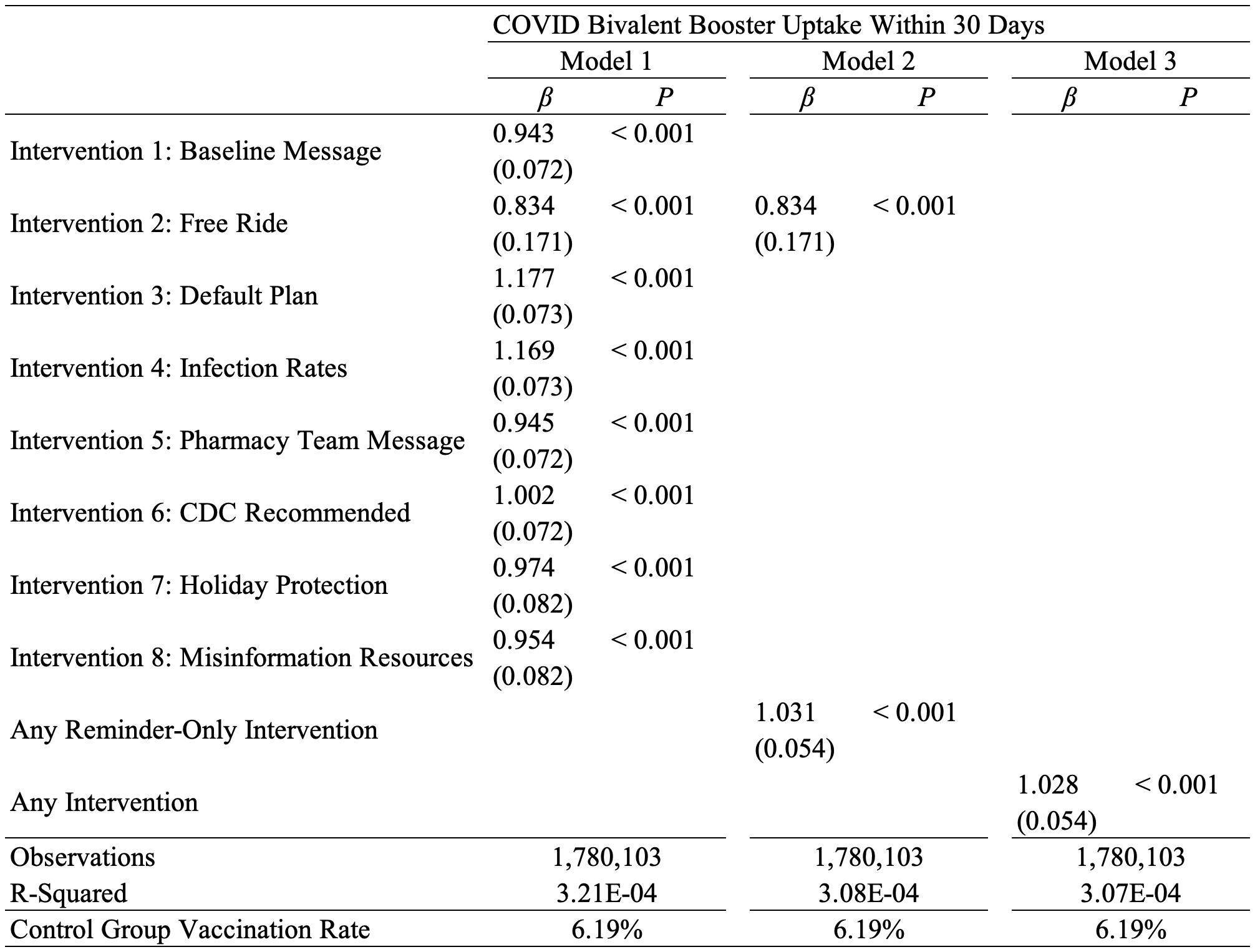


*Note:* This table reports the results of three ordinary least squares (OLS) regressions to predict whether a given patient whose closest CVS Pharmacy is in a zip code with a high level of residents with a Bachelor’s degree received a COVID-19 booster vaccine at a CVS Pharmacy within 30 days of a patient’s study launch day. Model 1 relies on the same specification as our main regression model (Table 2, Model 1). Models 2 and 3 include different primary predictors. In Model 2, we include two primary predictors: an indicator for whether a patient received any reminder-only intervention and an indicator for whether a patient received our free ride intervention. In Model 3, we include a single pooled treatment indicator for whether a patient received any of our megastudy’s eight intervention conditions. All three regression models include indicators for whether the patient received their first text message on launch day 1 or launch day 2 (an indicator for receiving a message on launch day 3 is omitted). The control variables in all models are mean-centered using the mean of the holdout control. All regression coefficients and standard errors have been multiplied by 100 to improve interpretability (and thus reflect percentage point change(s) induced in vaccination uptake). Standard errors reported in parentheses are estimated robustly using HC1. Statistical tests of whether an individual regression coefficient is zero are all two-sided.

**Table S39. Subgroup analyses for patients whose closest CVS Pharmacy is in a county with a “low” level of Republican presidential votes in 2020** (“low” levels are defined by a median split; observations with below median values in our data are included here; median percent of Republican votes = 39.47%). Regression-estimated impact of each of our megastudy’s eight intervention conditions on bivalent COVID-19 booster uptake at CVS Pharmacy within 30 days of a patient’s study launch day for patients whose closest CVS Pharmacy is in a zip code with a low level of Republican presidential votes in 2020 according to the MIT Election Data + Science Lab, either breaking out all interventions individually (Model 1), pooling the reminder-only interventions (Model 2), or pooling all interventions (Model 3).


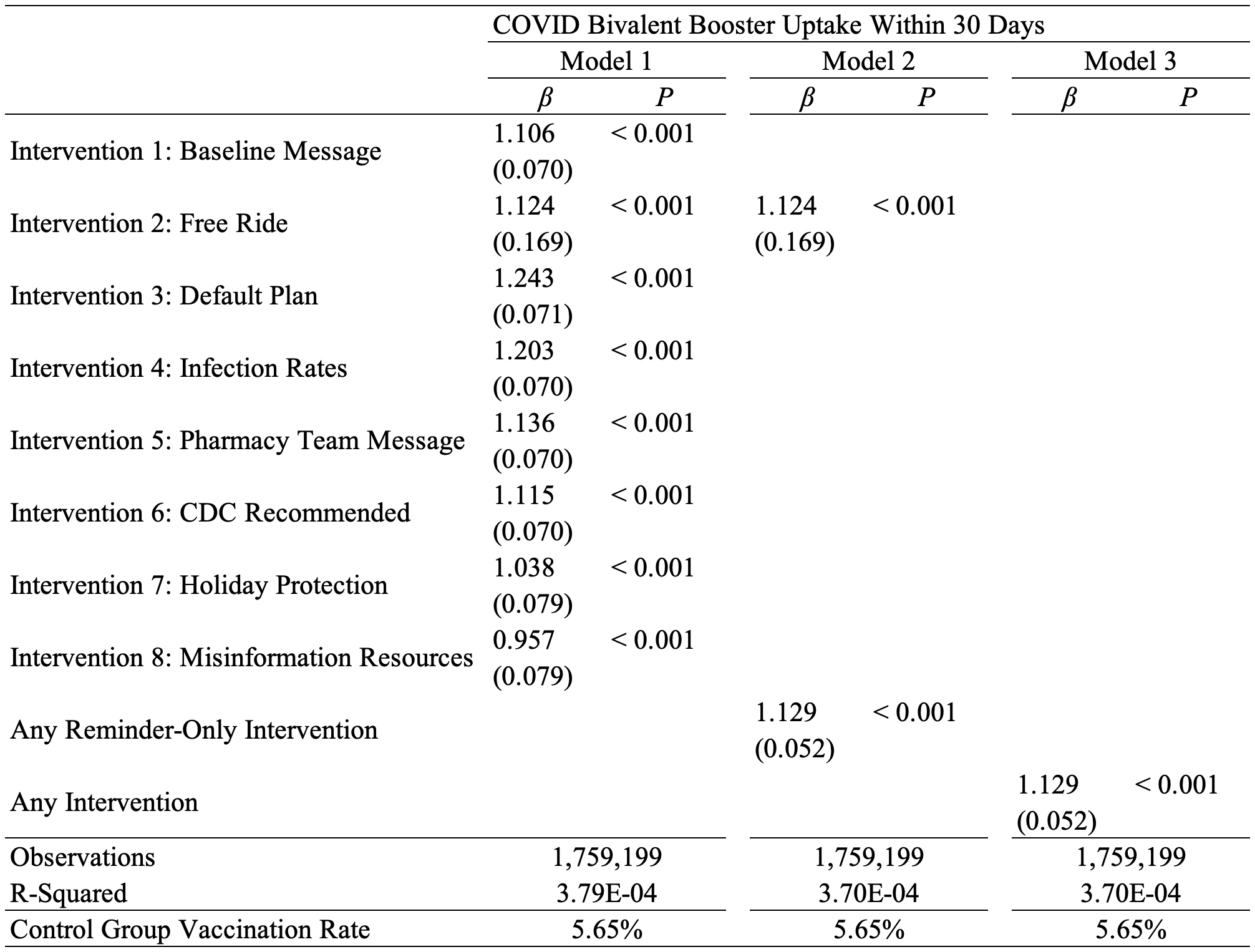


*Note:* This table reports the results of three ordinary least squares (OLS) regressions to predict whether a given patient whose closest CVS Pharmacy is in a zip code with a low level of Republican presidential votes in 2020 received a COVID-19 booster vaccine at a CVS Pharmacy within 30 days of a patient’s study launch day. Model 1 relies on the same specification as our main regression model (Table 2, Model 1). Models 2 and 3 include different primary predictors. In Model 2, we include two primary predictors: an indicator for whether a patient received any reminder-only intervention and an indicator for whether a patient received our free ride intervention. In Model 3, we include a single pooled treatment indicator for whether a patient received any of our megastudy’s eight intervention conditions. All three regression models include indicators for whether the patient received their first text message on launch day 1 or launch day 2 (an indicator for receiving a message on launch day 3 is omitted). The control variables in all models are mean-centered using the mean of the holdout control. All regression coefficients and standard errors have been multiplied by 100 to improve interpretability (and thus reflect percentage point change(s) induced in vaccination uptake). Standard errors reported in parentheses are estimated robustly using HC1. Statistical tests of whether an individual regression coefficient is zero are all two-sided.

**Table S40. Subgroup analyses for patients whose closest CVS Pharmacy is in a county with a “high” level of Republican presidential votes in 2020** (“high” levels are defined by a median split; observations with at or above median values in our data are included here; median percent of Republican votes = 39.47%). Regression-estimated impact of each of our megastudy’s eight intervention conditions on bivalent COVID-19 booster uptake at CVS Pharmacy within 30 days of a patient’s study launch day for patients whose closest CVS Pharmacy is in a zip code with a high level of Republican presidential votes in 2020 according to the MIT Election Data + Science Lab, either breaking out all interventions individually (Model 1), pooling the reminder-only interventions (Model 2), or pooling all interventions (Model 3).


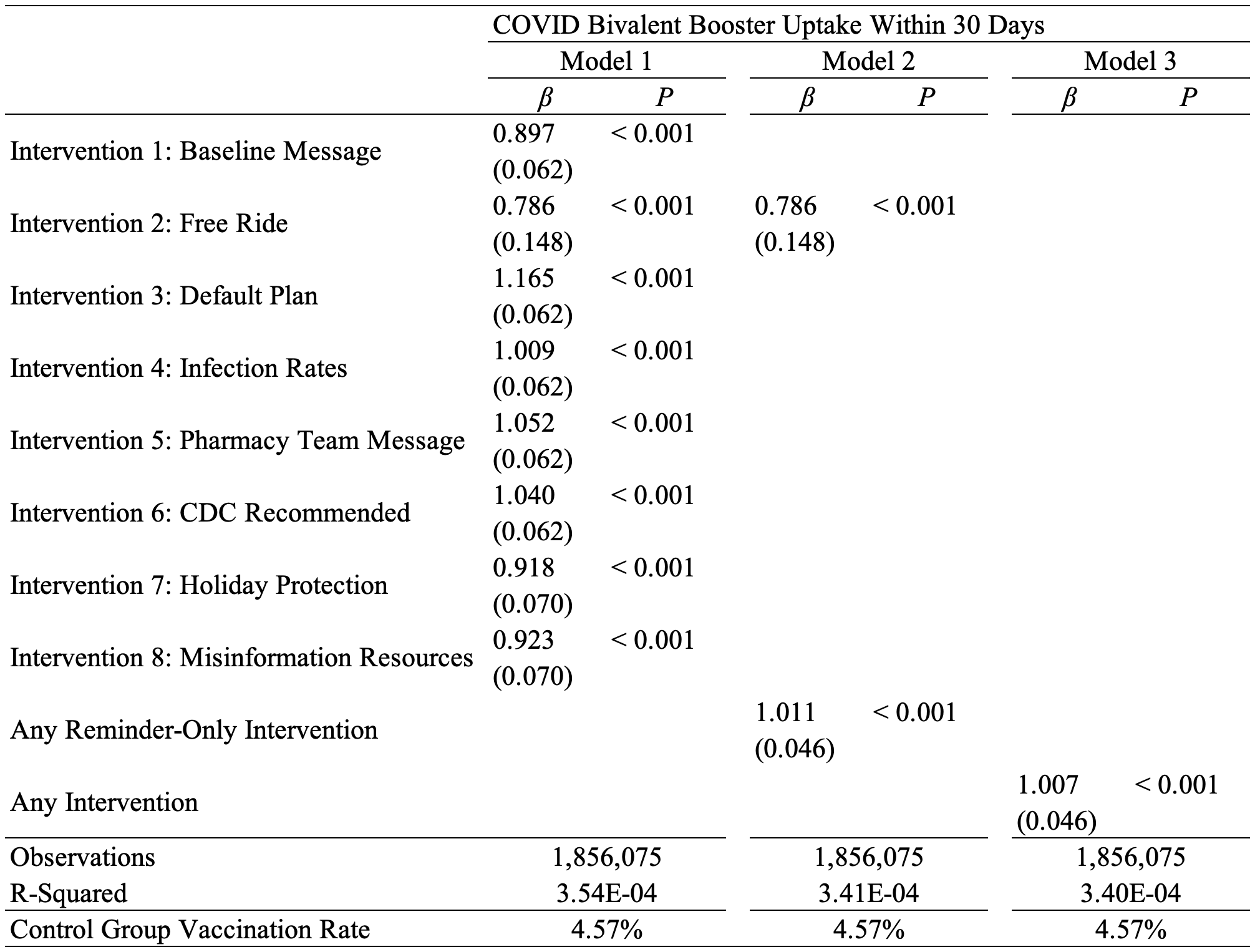


*Note:* This table reports the results of three ordinary least squares (OLS) regressions to predict whether a given patient whose closest CVS Pharmacy is in a zip code with a high level of Republican presidential votes in 2020 received a COVID-19 booster vaccine at a CVS Pharmacy within 30 days of a patient’s study launch day. Model 1 relies on the same specification as our main regression model (Table 2, Model 1). Models 2 and 3 include different primary predictors. In Model 2, we include two primary predictors: an indicator for whether a patient received any reminder-only intervention and an indicator for whether a patient received our free ride intervention. In Model 3, we include a single pooled treatment indicator for whether a patient received any of our megastudy’s eight intervention conditions. All three regression models include indicators for whether the patient received their first text message on launch day 1 or launch day 2 (an indicator for receiving a message on launch day 3 is omitted). The control variables in all models are mean-centered using the mean of the holdout control. All regression coefficients and standard errors have been multiplied by 100 to improve interpretability (and thus reflect percentage point change(s) induced in vaccination uptake). Standard errors reported in parentheses are estimated robustly using HC1. Statistical tests of whether an individual regression coefficient is zero are all two-sided.

**Table S41. Subgroup analyses for patients whose closest CVS Pharmacy is in a zip code with a “low” CVS Pharmacy density (CVS Pharmacies per square mile)** (“low” density are defined by a median split; observations with below median values in our data are included here; median CVS Pharmacy density = 0.15 CVS Pharmacies per square mile). Regression-estimated impact of each of our megastudy’s eight intervention conditions on bivalent COVID-19 booster uptake at CVS Pharmacy within 30 days of a patient’s study launch day for patients whose closest CVS Pharmacy is in a zip code with a low level of CVS Pharmacies per square mile, either breaking out all interventions individually (Model 1), pooling the reminder-only interventions (Model 2), or pooling all interventions (Model 3).


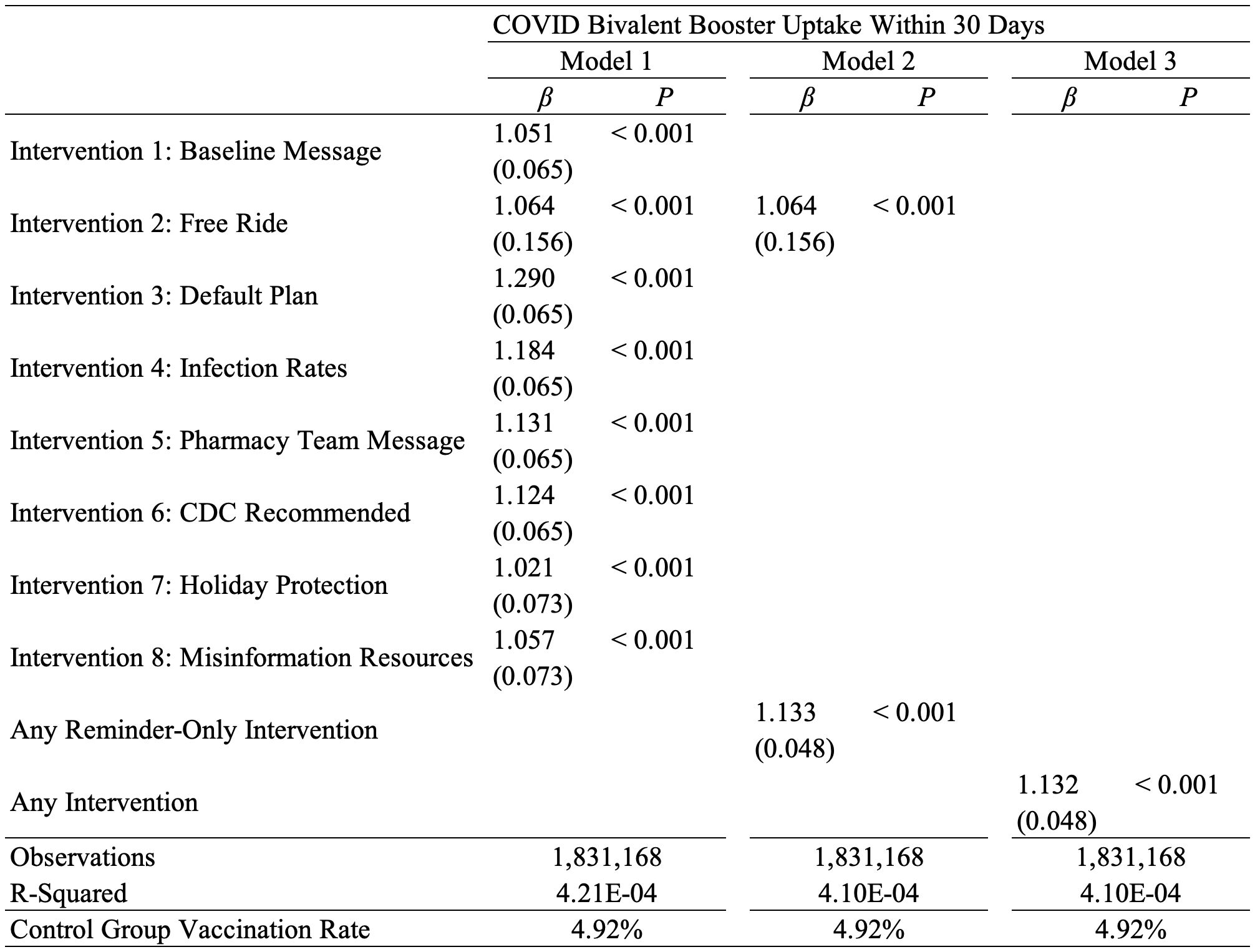


*Note:* This table reports the results of three ordinary least squares (OLS) regressions to predict whether a given patient whose closest CVS Pharmacy is in a zip code with a low density of CVS Pharmacies per square mile received a COVID-19 booster vaccine at a CVS Pharmacy within 30 days of a patient’s study launch day. Model 1 relies on the same specification as our main regression model (Table 2, Model 1). Models 2 and 3 include different primary predictors. In Model 2, we include two primary predictors: an indicator for whether a patient received any reminder-only intervention and an indicator for whether a patient received our free ride intervention. In Model 3, we include a single pooled treatment indicator for whether a patient received any of our megastudy’s eight intervention conditions. All three regression models include indicators for whether the patient received their first text message on launch day 1 or launch day 2 (an indicator for receiving a message on launch day 3 is omitted). The control variables in all models are mean-centered using the mean of the holdout control. All regression coefficients and standard errors have been multiplied by 100 to improve interpretability (and thus reflect percentage point change(s) induced in vaccination uptake). Standard errors reported in parentheses are estimated robustly using HC1. Statistical tests of whether an individual regression coefficient is zero are all two-sided.

**Table S42. Subgroup analyses for patients whose closest CVS Pharmacy is in a zip code with a “high” CVS Pharmacy density (CVS Pharmacies per square mile)** (“high” densities are defined by a median split; observations with at or above median values in our data are included here; median CVS Pharmacy density = 0.15 CVS Pharmacies per square mile). Regression-estimated impact of each of our megastudy’s eight intervention conditions on bivalent COVID-19 booster uptake at CVS Pharmacy within 30 days of a patient’s study launch day for patients whose closest CVS Pharmacy is in a zip code with a high level of CVS Pharmacies per square mile, either breaking out all interventions individually (Model 1), pooling the reminder-only interventions (Model 2), or pooling all interventions (Model 3).


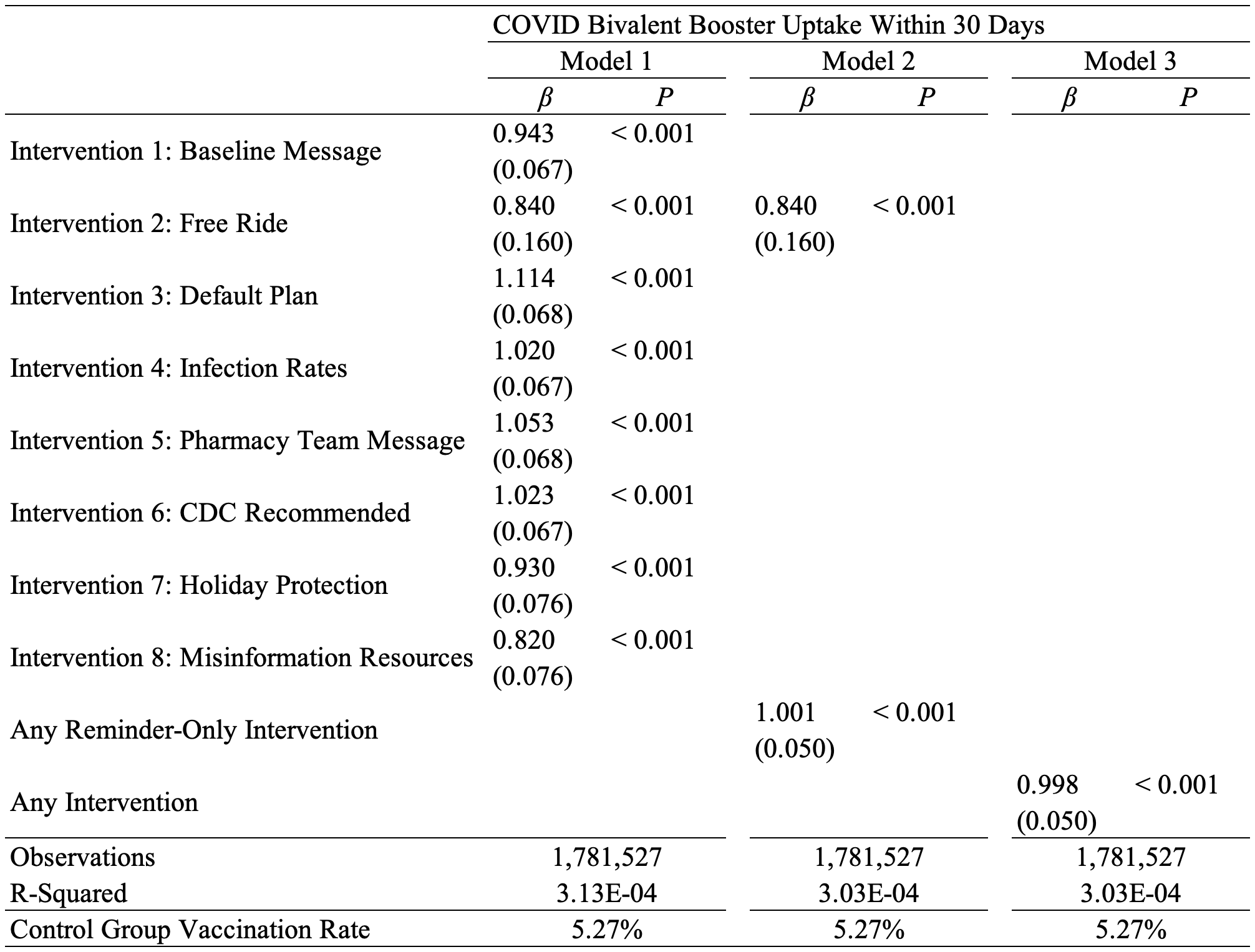


*Note:* This table reports the results of three ordinary least squares (OLS) regressions to predict whether a given patient whose closest CVS Pharmacy is in a zip code with a low level of CVS Pharmacies per square mile received a COVID-19 booster vaccine at a CVS Pharmacy within 30 days of a patient’s study launch day. Model 1 relies on the same specification as our main regression model (Table 2, Model 1). Models 2 and 3 include different primary predictors. In Model 2, we include two primary predictors: an indicator for whether a patient received any reminder-only intervention and an indicator for whether a patient received our free ride intervention. In Model 3, we include a single pooled treatment indicator for whether a patient received any of our megastudy’s eight intervention conditions. All three regression models include indicators for whether the patient received their first text message on launch day 1 or launch day 2 (an indicator for receiving a message on launch day 3 is omitted). The control variables in all models are mean-centered using the mean of the holdout control. All regression coefficients and standard errors have been multiplied by 100 to improve interpretability (and thus reflect percentage point change(s) induced in vaccination uptake). Standard errors reported in parentheses are estimated robustly using HC1. Statistical tests of whether an individual regression coefficient is zero are all two-sided.

**Table S43. Subgroup analyses for patients whose closest CVS Pharmacy is in a county with a “low” level COVID-19 primary series vaccination rate** (“low” levels are defined by a median split; observations with below median values in our data are included here; median COVID-19 vaccination rate = 73.30%). Regression-estimated impact of each of our megastudy’s eight intervention conditions on bivalent COVID-19 booster uptake at CVS Pharmacy within 30 days of a patient’s study launch day for patients whose closest CVS Pharmacy is in a county with a low level COVID-19 primary series vaccination rate according to the CDC, either breaking out all interventions individually (Model 1), pooling the reminder-only interventions (Model 2), or pooling all interventions (Model 3).


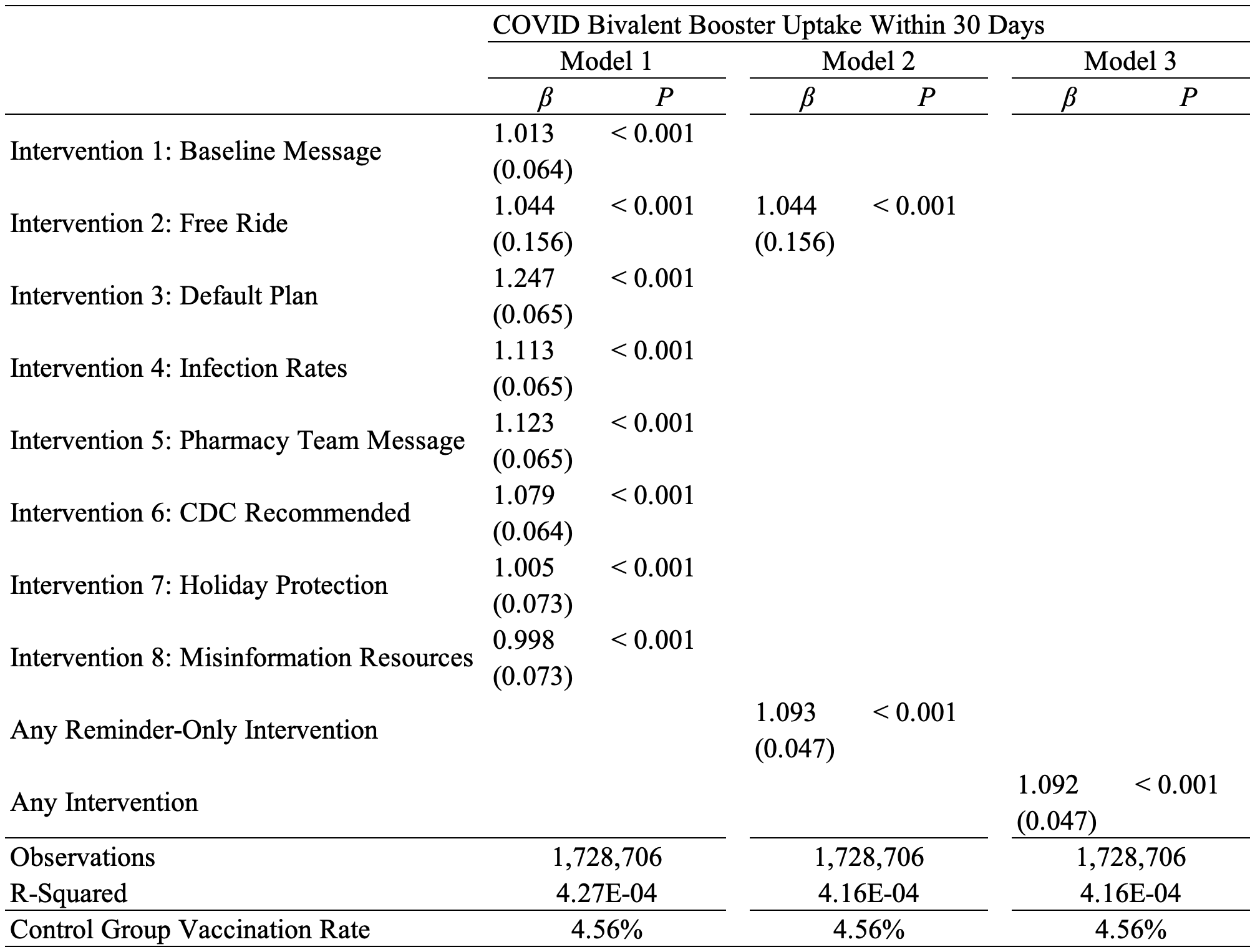


*Note:* This table reports the results of three ordinary least squares (OLS) regressions to predict whether a given patient whose closest CVS Pharmacy is in a county with a low level COVID-19 primary series vaccination rate received a COVID-19 booster vaccine at a CVS Pharmacy within 30 days of a patient’s study launch day. Model 1 relies on the same specification as our main regression model (Table 2, Model 1). Models 2 and 3 include different primary predictors. In Model 2, we include two primary predictors: an indicator for whether a patient received any reminder-only intervention and an indicator for whether a patient received our free ride intervention. In Model 3, we include a single pooled treatment indicator for whether a patient received any of our megastudy’s eight intervention conditions. All three regression models include indicators for whether the patient received their first text message on launch day 1 or launch day 2 (an indicator for receiving a message on launch day 3 is omitted). The control variables in all models are mean-centered using the mean of the holdout control. All regression coefficients and standard errors have been multiplied by 100 to improve interpretability (and thus reflect percentage point change(s) induced in vaccination uptake). Standard errors reported in parentheses are estimated robustly using HC1. Statistical tests of whether an individual regression coefficient is zero are all two-sided.

**Table S44. Subgroup analyses for patients whose closest CVS Pharmacy is in a county with a “high” level COVID-19 primary series vaccination rate** (“high” levels are defined by a median split; observations with at or above median values in our data are included here; median COVID-19 vaccination rate = 73.30%). Regression-estimated impact of each of our megastudy’s eight intervention conditions on bivalent COVID-19 booster uptake at CVS Pharmacy within 30 days of a patient’s study launch day for patients whose closest CVS Pharmacy is in a county with a high level COVID-19 primary series vaccination rate according to the CDC, either breaking out all interventions individually (Model 1), pooling the reminder-only interventions (Model 2), or pooling all interventions (Model 3).


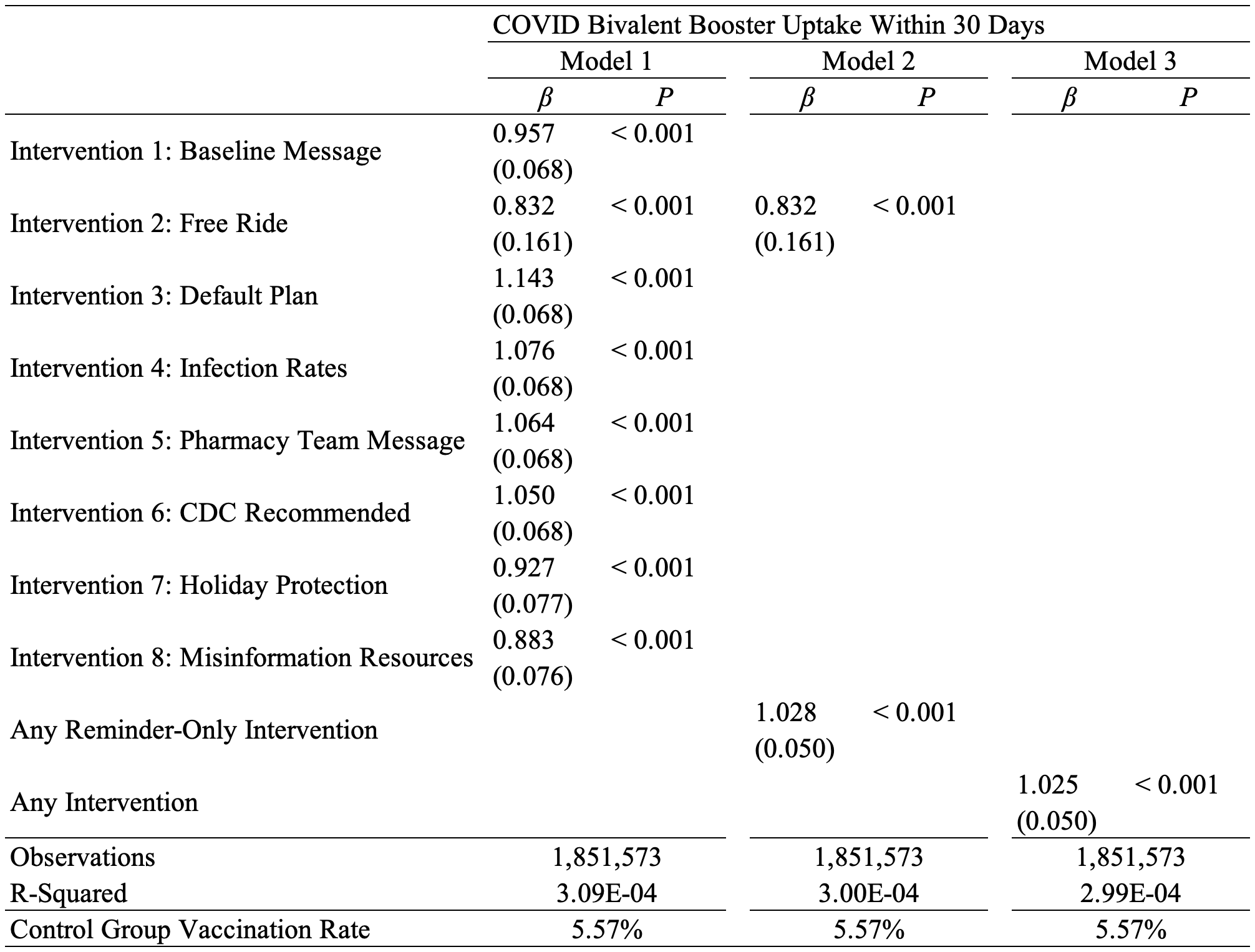


*Note:* This table reports the results of three ordinary least squares (OLS) regressions to predict whether a given patient whose closest CVS Pharmacy is in a county with a high level COVID-19 primary series vaccination rate received a COVID-19 booster vaccine at a CVS Pharmacy within 30 days of a patient’s study launch day. Model 1 relies on the same specification as our main regression model (Table 2, Model 1). Models 2 and 3 include different primary predictors. In Model 2, we include two primary predictors: an indicator for whether a patient received any reminder-only intervention and an indicator for whether a patient received our free ride intervention. In Model 3, we include a single pooled treatment indicator for whether a patient received any of our megastudy’s eight intervention conditions. All three regression models include indicators for whether the patient received their first text message on launch day 1 or launch day 2 (an indicator for receiving a message on launch day 3 is omitted). The control variables in all models are mean-centered using the mean of the holdout control. All regression coefficients and standard errors have been multiplied by 100 to improve interpretability (and thus reflect percentage point change(s) induced in vaccination uptake). Standard errors reported in parentheses are estimated robustly using HC1. Statistical tests of whether an individual regression coefficient is zero are all two-sided.

**Table S45. Subgroup analyses for patients whose closest CVS Pharmacy is in a county with a “low” level initial COVID-19 booster vaccination rate** (“low” levels are defined by a median split; observations with below median values in our data are included here; median COVID-19 booster rate = 50.00%). Regression-estimated impact of each of our megastudy’s eight intervention conditions on bivalent COVID-19 booster uptake at CVS Pharmacy within 30 days of a patient’s study launch day for patients whose closest CVS Pharmacy is in a county with a low level initial COVID-19 booster vaccination rate according to the CDC, either breaking out all interventions individually (Model 1), pooling the reminder-only interventions (Model 2), or pooling all interventions (Model 3).


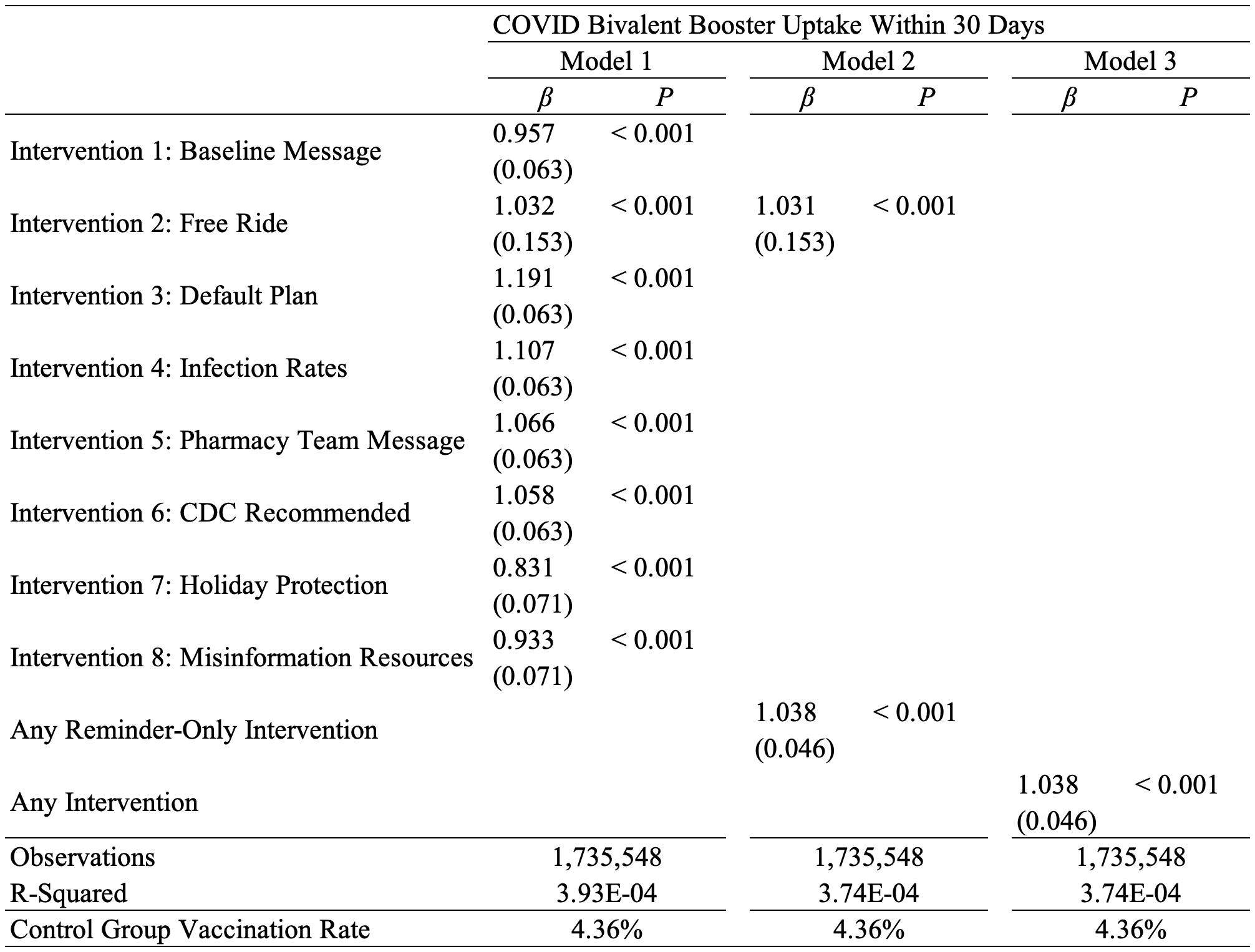


*Note:* This table reports the results of three ordinary least squares (OLS) regressions to predict whether a given patient whose closest CVS Pharmacy is in a county with a low initial COVID-19 booster vaccination rate received a COVID-19 booster vaccine at a CVS Pharmacy within 30 days of a patient’s study launch day. Model 1 relies on the same specification as our main regression model (Table 2, Model 1). Models 2 and 3 include different primary predictors. In Model 2, we include two primary predictors: an indicator for whether a patient received any reminder-only intervention and an indicator for whether a patient received our free ride intervention. In Model 3, we include a single pooled treatment indicator for whether a patient received any of our megastudy’s eight intervention conditions. All three regression models include indicators for whether the patient received their first text message on launch day 1 or launch day 2 (an indicator for receiving a message on launch day 3 is omitted). The control variables in all models are mean-centered using the mean of the holdout control. All regression coefficients and standard errors have been multiplied by 100 to improve interpretability (and thus reflect percentage point change(s) induced in vaccination uptake). Standard errors reported in parentheses are estimated robustly using HC1. Statistical tests of whether an individual regression coefficient is zero are all two-sided.

**Table S46. Subgroup analyses for patients whose closest CVS Pharmacy is in a county with a “high” level initial COVID-19 booster vaccination rate** (“high” levels are defined by a median split; observations with at or above median values in our data are included here; median COVID-19 booster rate = 50.00%). Regression-estimated impact of each of our megastudy’s eight intervention conditions on bivalent COVID-19 booster uptake at CVS Pharmacy within 30 days of a patient’s study launch day for patients whose closest CVS Pharmacy is in a county with a high level initial COVID-19 booster vaccination rate according to the CDC, either breaking out all interventions individually (Model 1), pooling the reminder-only interventions (Model 2), or pooling all interventions (Model 3).


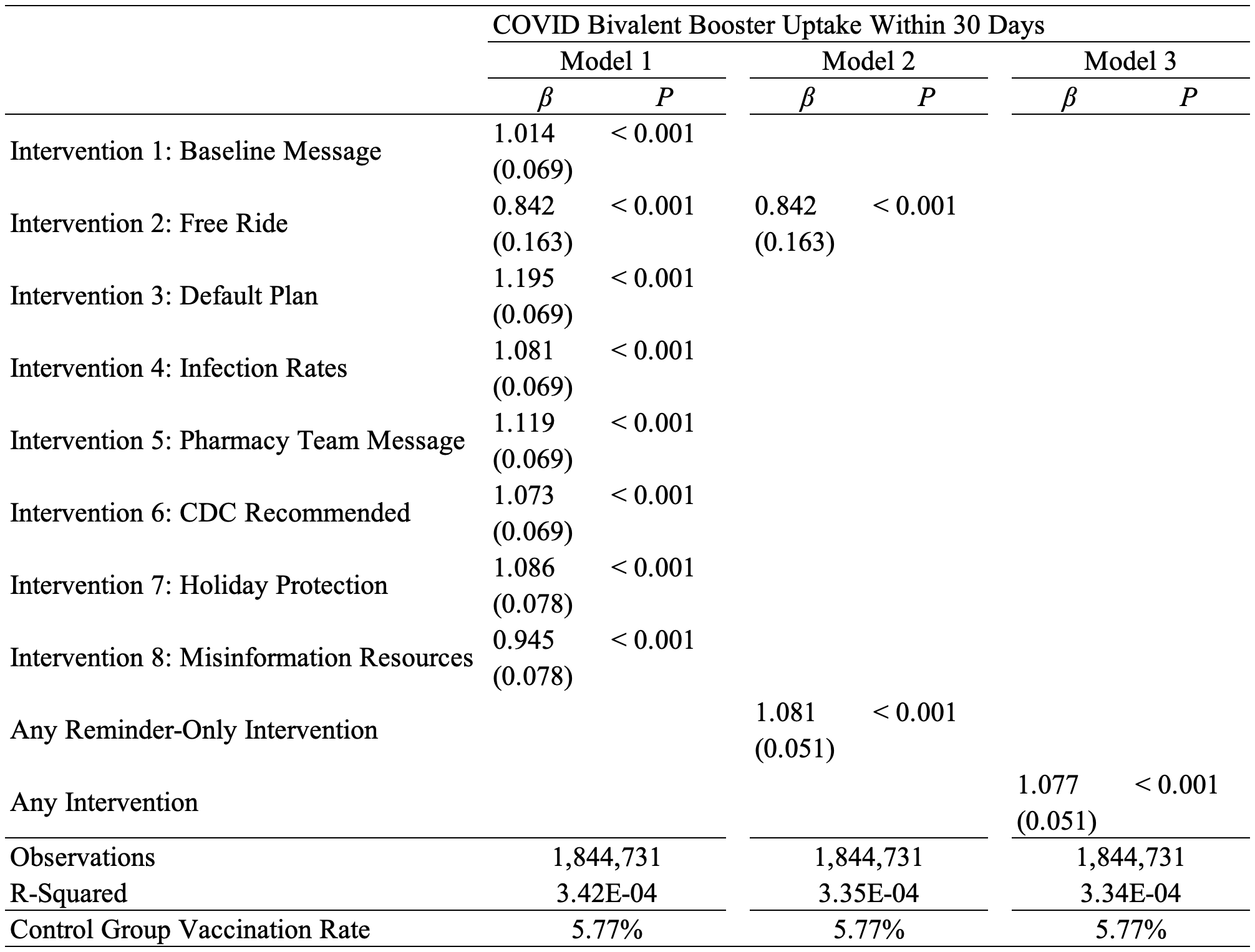


*Note:* This table reports the results of three ordinary least squares (OLS) regressions to predict whether a given patient whose closest CVS Pharmacy is in a county with a high initial COVID-19 booster vaccination rate received a COVID-19 booster vaccine at a CVS Pharmacy within 30 days of a patient’s study launch day. Model 1 relies on the same specification as our main regression model (Table 2, Model 1). Models 2 and 3 include different primary predictors. In Model 2, we include two primary predictors: an indicator for whether a patient received any reminder-only intervention and an indicator for whether a patient received our free ride intervention. In Model 3, we include a single pooled treatment indicator for whether a patient received any of our megastudy’s eight intervention conditions. All three regression models include indicators for whether the patient received their first text message on launch day 1 or launch day 2 (an indicator for receiving a message on launch day 3 is omitted). The control variables in all models are mean-centered using the mean of the holdout control. All regression coefficients and standard errors have been multiplied by 100 to improve interpretability (and thus reflect percentage point change(s) induced in vaccination uptake). Standard errors reported in parentheses are estimated robustly using HC1. Statistical tests of whether an individual regression coefficient is zero are all two-sided.

**HETEROGENEITY ANALYSES BASED ON PATIENT NEIGHBOURHOOD CHARACTERISTICS**

**Table S47. Heterogeneity analyses by percentage of White residents in the zip code of a patient’s closest CVS Pharmacy**. Regression-estimated impact of each of our megastudy’s eight intervention conditions on bivalent COVID-19 booster uptake at CVS Pharmacy within 30 days of a patient’s study launch day as a function of the percentage of White residents in the zip code of a patient’s closest CVS Pharmacy according to CVS Pharmacy, either breaking out all interventions individually (Model 1), pooling the reminder-only interventions (Model 2), or pooling all interventions (Model 3).

**
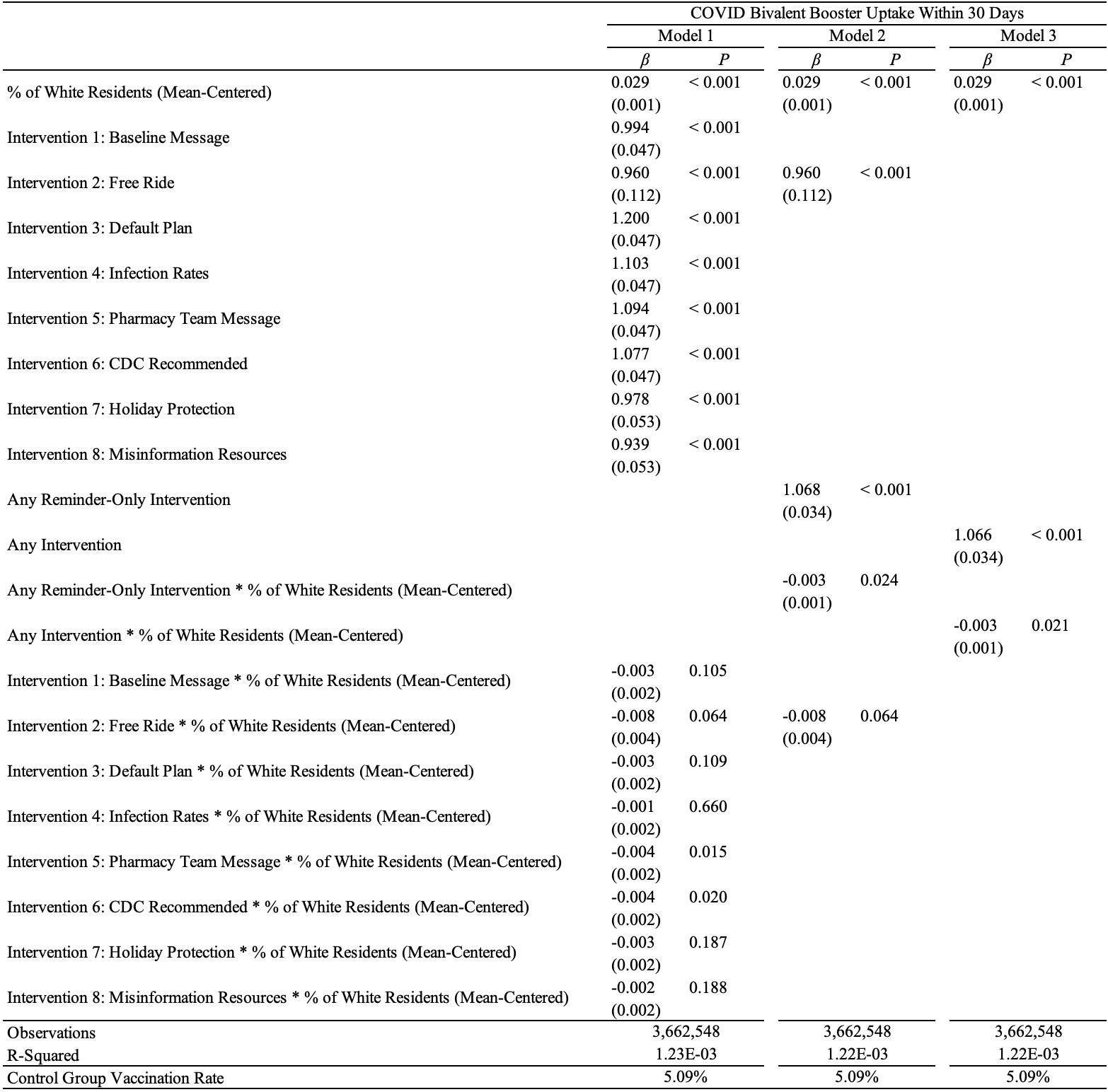
**

*Note:* This table reports the results of three ordinary least squares (OLS) regressions to predict whether a given patient received a COVID-19 booster vaccine at a CVS Pharmacy within 30 days of a patient’s study launch day. Model 1 relies on the same specification as our main regression model (Table 2, Model 1), while Model 2 and 3 include different primary predictors. In Model 2, we include two primary predictors: an indicator for whether a patient received any reminder-only intervention and an indicator for whether a patient received our free ride intervention. In Model 3, we include a single pooled treatment indicator for whether a patient received any of our megastudy’s eight intervention conditions. Each model includes a mean-centered measure for the percentage of White residents in the zip code of a patient’s closest CVS Pharmacy according to CVS Pharmacy, an indicator for whether this metric has a missing value, and interactions between these variables and each intervention indicator. All three regression models also include indicators for whether the patient received their first text message on launch day 1 or launch day 2 (an indicator for receiving a message on launch day 3 is omitted). The control variables in all models are mean-centered using the mean of the holdout control. All regression coefficients and standard errors have been multiplied by 100 to improve interpretability (and thus reflect percentage point change(s) induced in vaccination uptake). Standard errors reported in parentheses are estimated robustly using HC1. Statistical tests of whether an individual regression coefficient is zero are all two-sided.

**Table S48. Heterogeneity analyses by percentage of Black residents in the zip code of a patient’s closest CVS Pharmacy**. Regression-estimated impact of each of our megastudy’s eight intervention conditions on bivalent COVID-19 booster uptake at CVS Pharmacy within 30 days of a patient’s study launch day as a function of the percentage of Black residents in the zip code of a patient’s closest CVS Pharmacy according to CVS Pharmacy, either breaking out all interventions individually (Model 1), pooling the reminder-only interventions (Model 2), or pooling all interventions (Model 3).

**
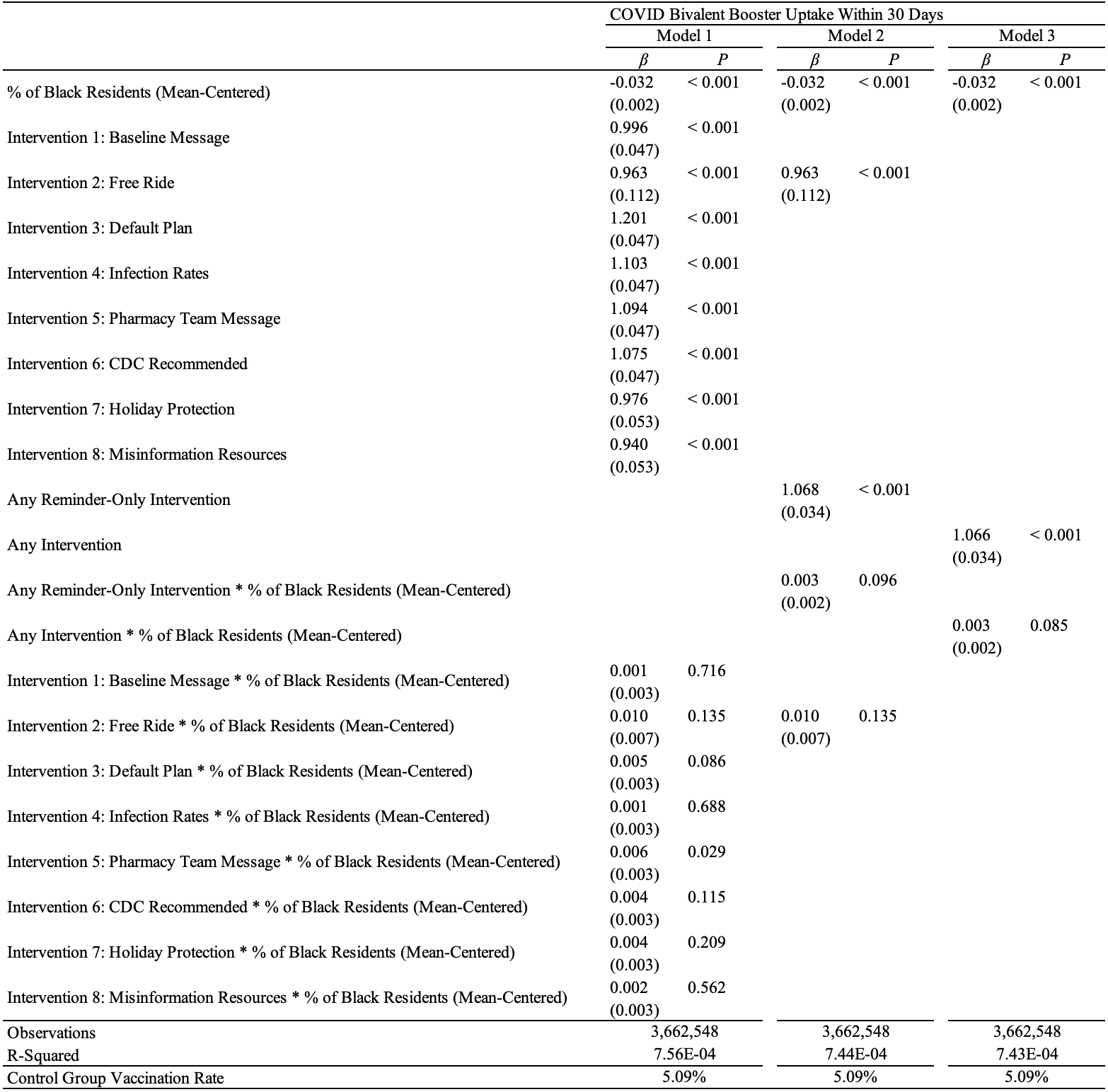
**

*Note:* This table reports the results of three ordinary least squares (OLS) regressions to predict whether a given patient received a COVID-19 booster vaccine at a CVS Pharmacy within 30 days of a patient’s study launch day. Model 1 relies on the same specification as our main regression model (Table 2, Model 1), while Model 2 and 3 include different primary predictors. In Model 2, we include two primary predictors: an indicator for whether a patient received any reminder-only intervention and an indicator for whether a patient received our free ride intervention. In Model 3, we include a single pooled treatment indicator for whether a patient received any of our megastudy’s eight intervention conditions. Each model includes a mean-centered measure for the percentage of Black residents in the zip code of a patient’s closest CVS Pharmacy according to CVS Pharmacy, an indicator for whether this metric has a missing value, and interactions between these variables and each intervention indicator. All three regression models also include indicators for whether the patient received their first text message on launch day 1 or launch day 2 (an indicator for receiving a message on launch day 3 is omitted). The control variables in all models are mean-centered using the mean of the holdout control. All regression coefficients and standard errors have been multiplied by 100 to improve interpretability (and thus reflect percentage point change(s) induced in vaccination uptake). Standard errors reported in parentheses are estimated robustly using HC1. Statistical tests of whether an individual regression coefficient is zero are all two-sided.

**Table S49. Heterogeneity analyses by percentage of Asian residents in the zip code of a patient’s closest CVS Pharmacy**. Regression-estimated impact of each of our megastudy’s eight intervention conditions on bivalent COVID-19 booster uptake at CVS Pharmacy within 30 days of a patient’s study launch day as a function of the percentage of Asian residents in the zip code of a patient’s closest CVS Pharmacy according to CVS Pharmacy, either breaking out all interventions individually (Model 1), pooling the reminder-only interventions (Model 2), or pooling all interventions (Model 3).

**
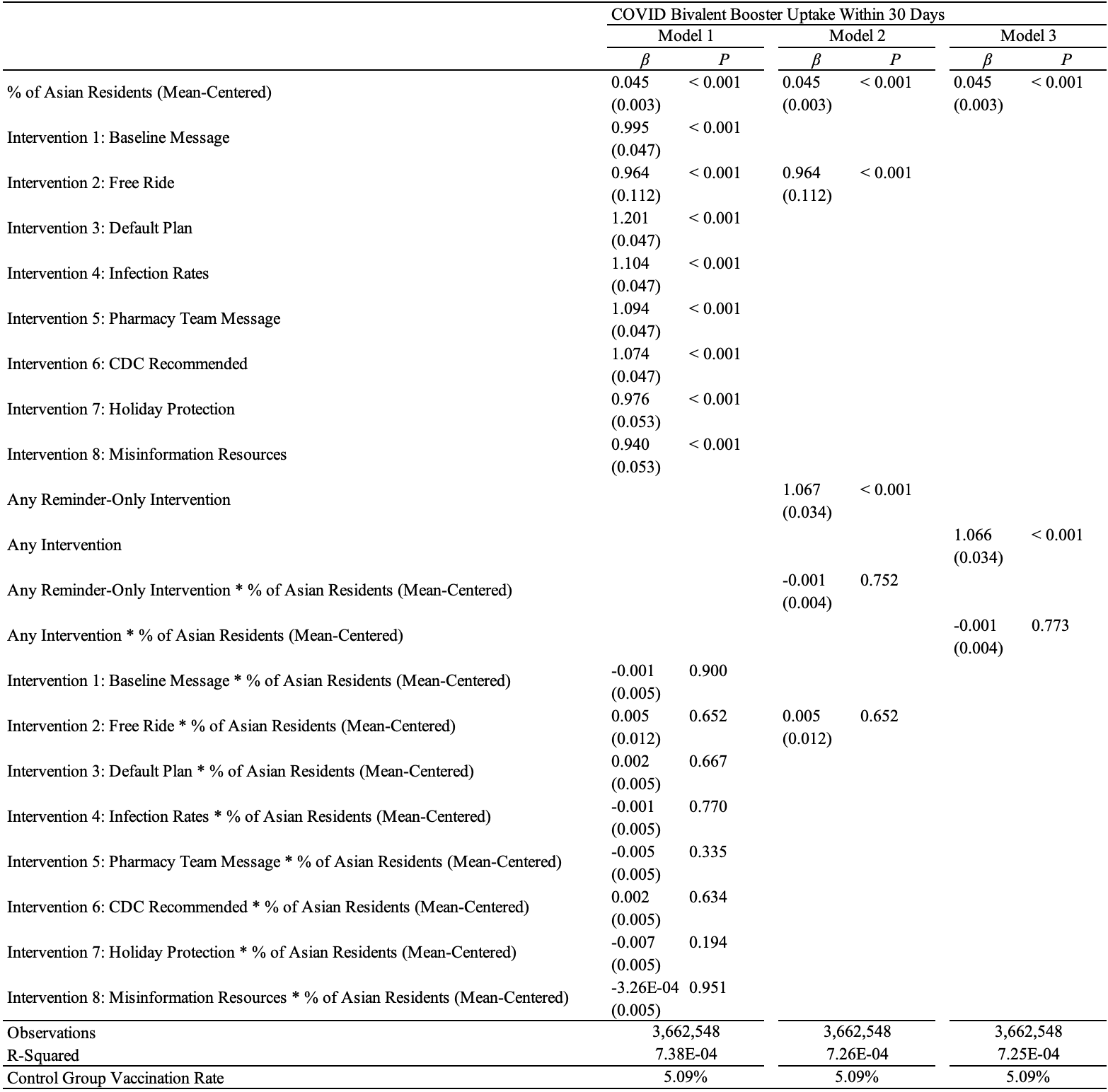
**

*Note:* This table reports the results of three ordinary least squares (OLS) regressions to predict whether a given patient received a COVID-19 booster vaccine at a CVS Pharmacy within 30 days of a patient’s study launch day. Model 1 relies on the same specification as our main regression model (Table 2, Model 1), while Model 2 and 3 include different primary predictors. In Model 2, we include two primary predictors: an indicator for whether a patient received any reminder-only intervention and an indicator for whether a patient received our free ride intervention. In Model 3, we include a single pooled treatment indicator for whether a patient received any of our megastudy’s eight intervention conditions. Each model includes a mean-centered measure for the percentage of Asian residents in the zip code of a patient’s closest CVS Pharmacy according to CVS Pharmacy, an indicator for whether this metric has a missing value, and interactions between these variables and each intervention indicator. All three regression models also include indicators for whether the patient received their first text message on launch day 1 or launch day 2 (an indicator for receiving a message on launch day 3 is omitted). The control variables in all models are mean-centered using the mean of the holdout control. All regression coefficients and standard errors have been multiplied by 100 to improve interpretability (and thus reflect percentage point change(s) induced in vaccination uptake). Standard errors reported in parentheses are estimated robustly using HC1. Statistical tests of whether an individual regression coefficient is zero are all two-sided.

**Table S50. Heterogeneity analyses by percentage of Hispanic residents in the zip code of a patient’s closest CVS Pharmacy**. Regression-estimated impact of each of our megastudy’s eight intervention conditions on bivalent COVID-19 booster uptake at CVS Pharmacy within 30 days of a patient’s study launch day as a function of the percentage of Hispanic residents in the zip code of a patient’s closest CVS Pharmacy according to CVS Pharmacy, either breaking out all interventions individually (Model 1), pooling the reminder-only interventions (Model 2), or pooling all interventions (Model 3).

**
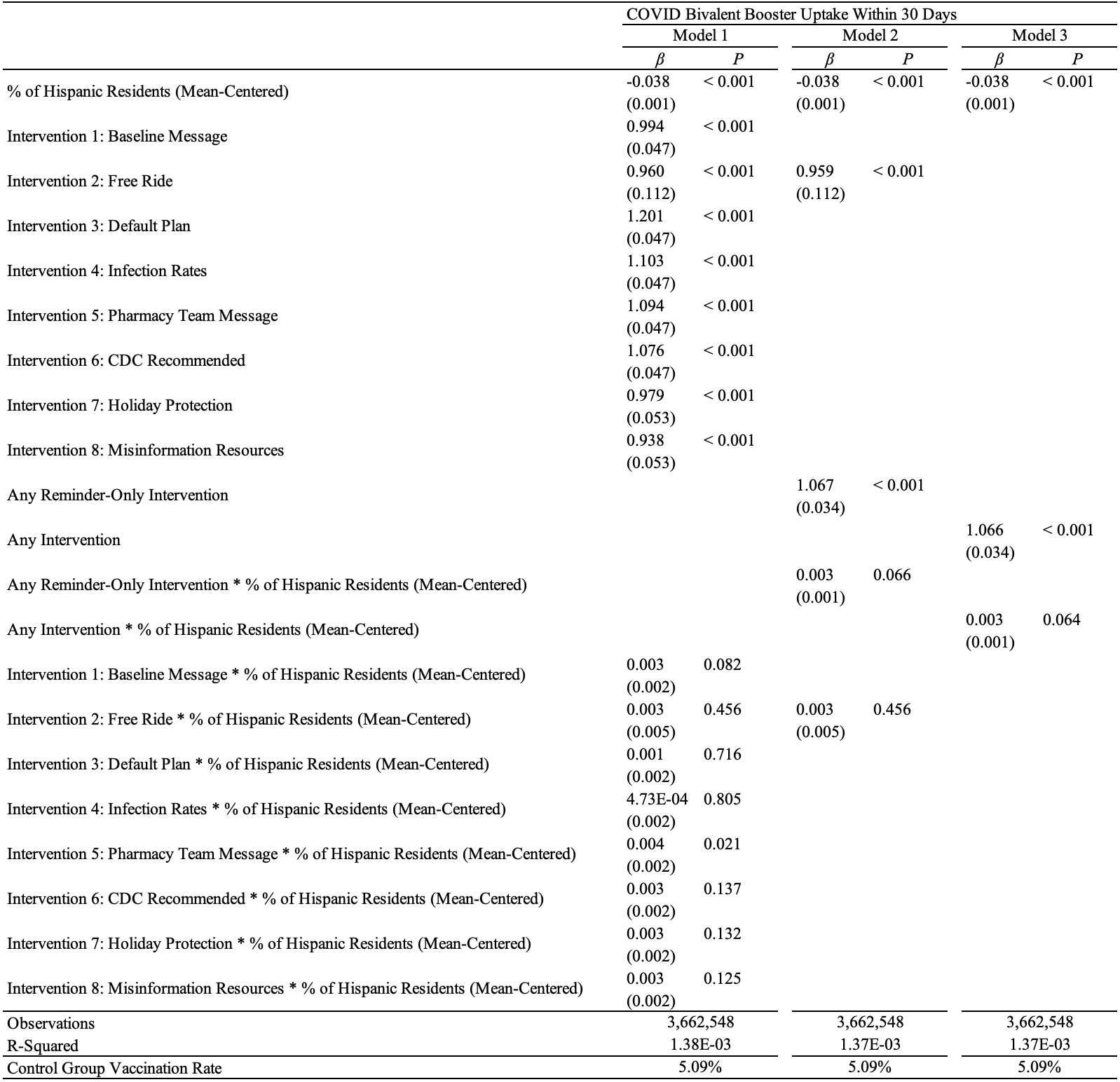
**

*Note:* This table reports the results of three ordinary least squares (OLS) regressions to predict whether a given patient received a COVID-19 booster vaccine at a CVS Pharmacy within 30 days of a patient’s study launch day. Model 1 relies on the same specification as our main regression model (Table 2, Model 1), while Model 2 and 3 include different primary predictors. In Model 2, we include two primary predictors: an indicator for whether a patient received any reminder-only intervention and an indicator for whether a patient received our free ride intervention. In Model 3, we include a single pooled treatment indicator for whether a patient received any of our megastudy’s eight intervention conditions. Each model includes a mean-centered measure for the percentage of Hispanic residents in the zip code of a patient’s closest CVS Pharmacy according to CVS Pharmacy, an indicator for whether this metric has a missing value, and interactions between these variables and each intervention indicator. All three regression models also include indicators for whether the patient received their first text message on launch day 1 or launch day 2 (an indicator for receiving a message on launch day 3 is omitted). The control variables in all models are mean-centered using the mean of the holdout control. All regression coefficients and standard errors have been multiplied by 100 to improve interpretability (and thus reflect percentage point change(s) induced in vaccination uptake). Standard errors reported in parentheses are estimated robustly using HC1. Statistical tests of whether an individual regression coefficient is zero are all two-sided.

**Table S51. Heterogeneity analyses by median income in the zip code of a patient’s closest CVS Pharmacy**. Regression-estimated impact of each of our megastudy’s eight intervention conditions on bivalent COVID-19 booster uptake at CVS Pharmacy within 30 days of a patient’s study launch day as a function of median income in the zip code of a patient’s closest CVS Pharmacy according to the U.S. Census, either breaking out all interventions individually (Model 1), pooling the reminder-only interventions (Model 2), or pooling all interventions (Model 3).

**
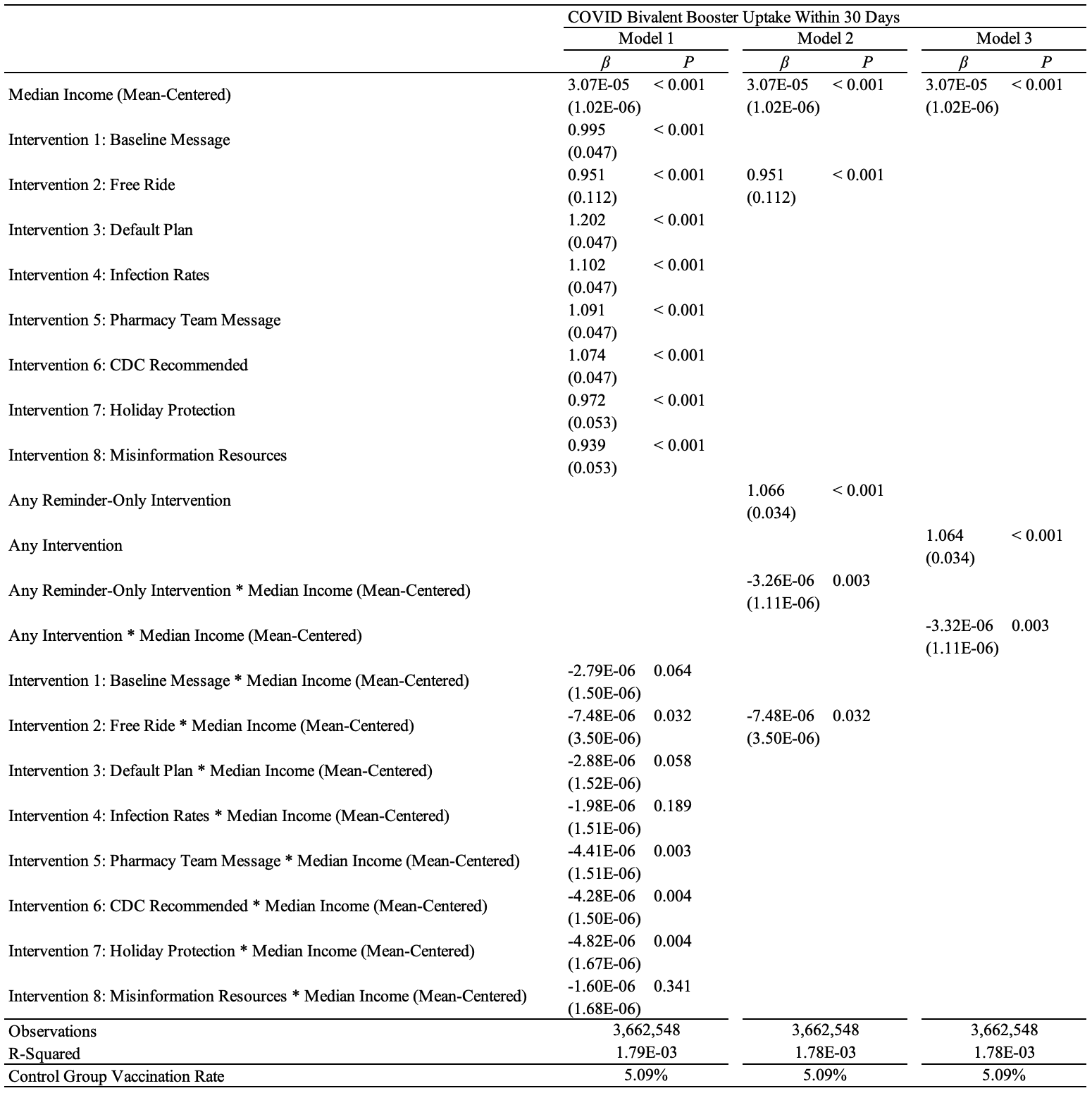
**

*Note:* This table reports the results of three ordinary least squares (OLS) regressions to predict whether a given patient received a COVID-19 booster vaccine at a CVS Pharmacy within 30 days of a patient’s study launch day. Model 1 relies on the same specification as our main regression model (Table 2, Model 1), while Model 2 and 3 include different primary predictors. In Model 2, we include two primary predictors: an indicator for whether a patient received any reminder-only intervention and an indicator for whether a patient received our free ride intervention. In Model 3, we include a single pooled treatment indicator for whether a patient received any of our megastudy’s eight intervention conditions. Each model includes a mean-centered measure for median income in the zip code of a patient’s closest CVS Pharmacy according to the U.S. Census, an indicator for whether this metric has a missing value, and interactions between these variables and each intervention indicator. All three regression models also include indicators for whether the patient received their first text message on launch day 1 or launch day 2 (an indicator for receiving a message on launch day 3 is omitted). The control variables in all models are mean-centered using the mean of the holdout control. All regression coefficients and standard errors have been multiplied by 100 to improve interpretability (and thus reflect percentage point change(s) induced in vaccination uptake). Standard errors reported in parentheses are estimated robustly using HC1. Statistical tests of whether an individual regression coefficient is zero are all two-sided.

**Table S52. Heterogeneity analyses by percentage of residents with a Bachelor’s degree in the zip code of a patient’s closest CVS Pharmacy**. Regression-estimated impact of each of our megastudy’s eight intervention conditions on bivalent COVID-19 booster uptake at CVS Pharmacy within 30 days of a patient’s study launch day as a function of the percentage of residents with a Bachelor’s degree in the zip code of a patient’s closest CVS Pharmacy according to the U.S. Census, either breaking out all interventions individually (Model 1), pooling the reminder-only interventions (Model 2), or pooling all interventions (Model 3).

**
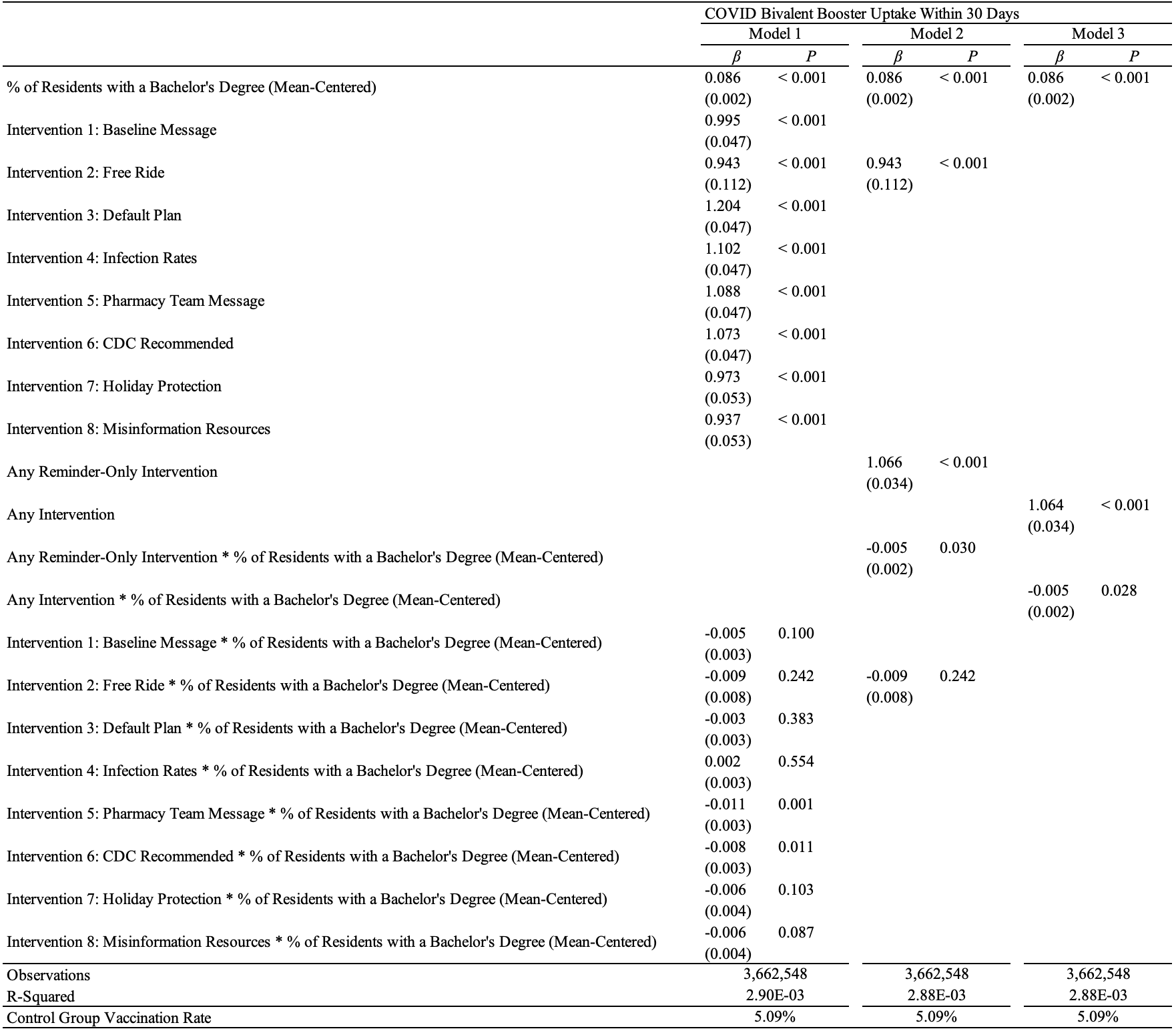
**

*Note:* This table reports the results of three ordinary least squares (OLS) regressions to predict whether a given patient received a COVID-19 booster vaccine at a CVS Pharmacy within 30 days of a patient’s study launch day. Model 1 relies on the same specification as our main regression model (Table 2, Model 1), while Model 2 and 3 include different primary predictors. In Model 2, we include two primary predictors: an indicator for whether a patient received any reminder-only intervention and an indicator for whether a patient received our free ride intervention. In Model 3, we include a single pooled treatment indicator for whether a patient received any of our megastudy’s eight intervention conditions. Each model includes a mean-centered measure of the percentage of residents with a Bachelor’s degree in the zip code of a patient’s closest CVS Pharmacy according to the U.S. Census, an indicator for whether this metric has a missing value, and interactions between these variables and each intervention indicator. All three regression models also include indicators for whether the patient received their first text message on launch day 1 or launch day 2 (an indicator for receiving a message on launch day 3 is omitted). The control variables in all models are mean-centered using the mean of the holdout control. All regression coefficients and standard errors have been multiplied by 100 to improve interpretability (and thus reflect percentage point change(s) induced in vaccination uptake). Standard errors reported in parentheses are estimated robustly using HC1. Statistical tests of whether an individual regression coefficient is zero are all two-sided.

**Table S53. Heterogeneity analyses by Republican share of 2020 Presidential votes in the county of a patient’s closest CVS Pharmacy**. Regression-estimated impact of each of our megastudy’s eight intervention conditions on bivalent COVID-19 booster uptake at CVS Pharmacy within 30 days of a patient’s study launch day as a function of Republican share of 2020 Presidential votes in the county of a patient’s closest CVS Pharmacy according to the MIT Election Data + Science Lab, either breaking out all interventions individually (Model 1), pooling the reminder-only interventions (Model 2), or pooling all interventions (Model 3).

**
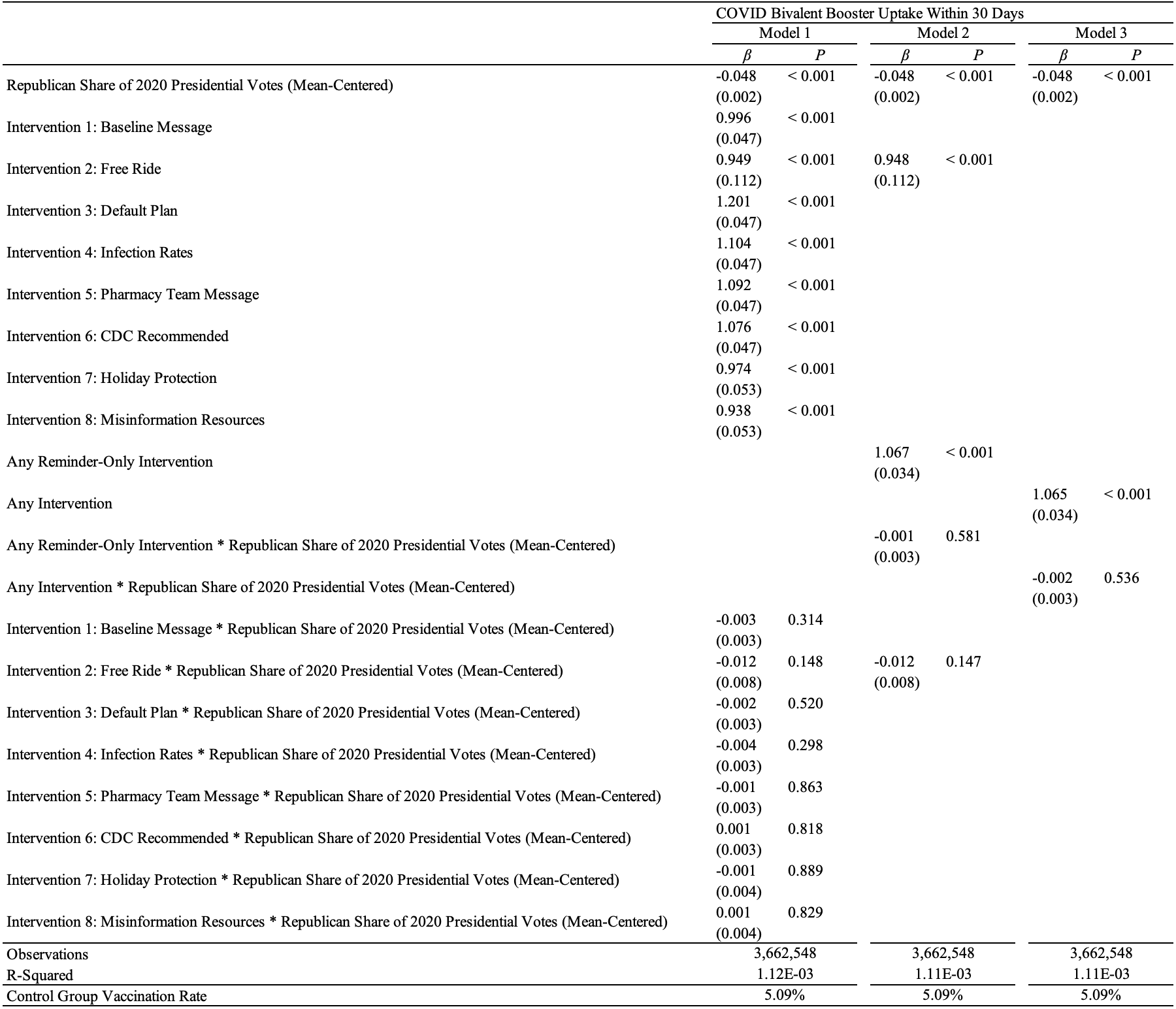
**

*Note:* This table reports the results of three ordinary least squares (OLS) regressions to predict whether a given patient received a COVID-19 booster vaccine at a CVS Pharmacy within 30 days of a patient’s study launch day. Model 1 relies on the same specification as our main regression model (Table 2, Model 1), while Model 2 and 3 include different primary predictors. In Model 2, we include two primary predictors: an indicator for whether a patient received any reminder-only intervention and an indicator for whether a patient received our free ride intervention. In Model 3, we include a single pooled treatment indicator for whether a patient received any of our megastudy’s eight intervention conditions. Each model includes a mean-centered measure of the Republican share of 2020 Presidential votes in the county of a patient’s closest CVS Pharmacy according to MIT Election Data + Science Lab, an indicator for whether this metric has a missing value, and interactions between these variables and each intervention indicator. All three regression models also include indicators for whether the patient received their first text message on launch day 1 or launch day 2 (an indicator for receiving a message on launch day 3 is omitted). The control variables in all models are mean-centered using the mean of the holdout control. All regression coefficients and standard errors have been multiplied by 100 to improve interpretability (and thus reflect percentage point change(s) induced in vaccination uptake). Standard errors reported in parentheses are estimated robustly using HC1. Statistical tests of whether an individual regression coefficient is zero are all two-sided.

**Table S54. Heterogeneity analyses by COVID primary series vaccination rate in the county of a patient’s closest CVS Pharmacy**. Regression-estimated impact of each of our megastudy’s eight intervention conditions on bivalent COVID-19 booster uptake at CVS Pharmacy within 30 days of a patient’s study launch day as a function of the COVID primary series vaccination rate in the county of a patient’s closest CVS Pharmacy according to the CDC, either breaking out all interventions individually (Model 1), pooling the reminder-only interventions (Model 2), or pooling all interventions (Model 3).

**
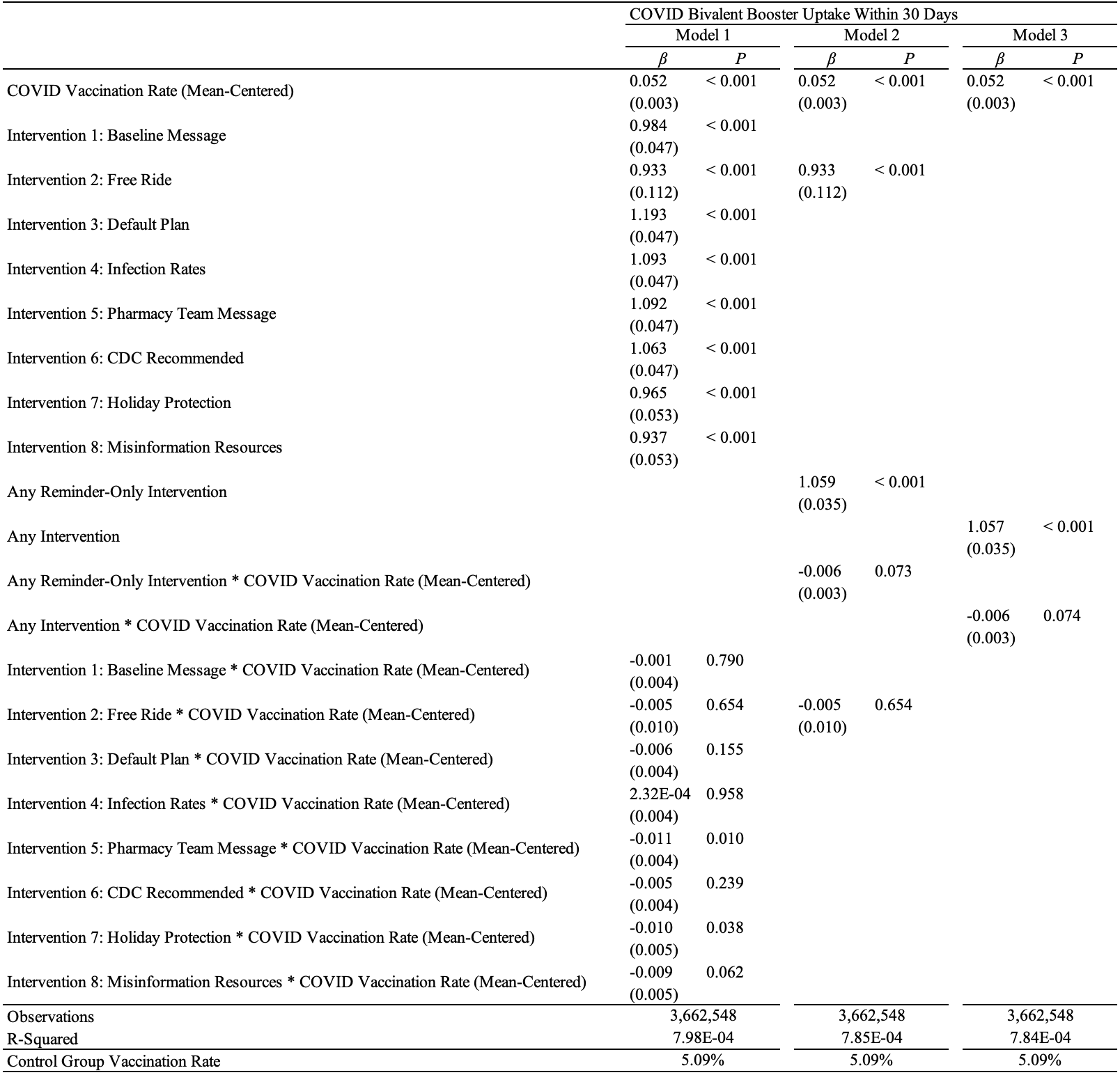
**

*Note:* This table reports the results of three ordinary least squares (OLS) regressions to predict whether a given patient received a COVID-19 booster vaccine at a CVS Pharmacy within 30 days of a patient’s study launch day. Model 1 relies on the same specification as our main regression model (Table 2, Model 1), while Model 2 and 3 include different primary predictors. In Model 2, we include two primary predictors: an indicator for whether a patient received any reminder-only intervention and an indicator for whether a patient received our free ride intervention. In Model 3, we include a single pooled treatment indicator for whether a patient received any of our megastudy’s eight intervention conditions. Each model includes a mean-centered measure for the COVID primary series vaccination rate in the county of a patient’s closest CVS Pharmacy according to the CDC, an indicator for whether this metric has a missing value, and interactions between these variables and each intervention indicator. All three regression models also include indicators for whether the patient received their first text message on launch day 1 or launch day 2 (an indicator for receiving a message on launch day 3 is omitted). The control variables in all models are mean-centered using the mean of the holdout control. All regression coefficients and standard errors have been multiplied by 100 to improve interpretability (and thus reflect percentage point change(s) induced in vaccination uptake). Standard errors reported in parentheses are estimated robustly using HC1. Statistical tests of whether an individual regression coefficient is zero are all two-sided.

**Table S55. Heterogeneity analyses by initial COVID booster vaccination rate in the county of a patient’s closest CVS Pharmacy**. Regression-estimated impact of each of our megastudy’s eight intervention conditions on bivalent COVID-19 booster uptake at CVS Pharmacy within 30 days of a patient’s study launch day as a function of initial COVID booster vaccination rate in the county of a patient’s closest CVS Pharmacy according to the CDC, either breaking out all interventions individually (Model 1), pooling the reminder-only interventions (Model 2), or pooling all interventions (Model 3).

**
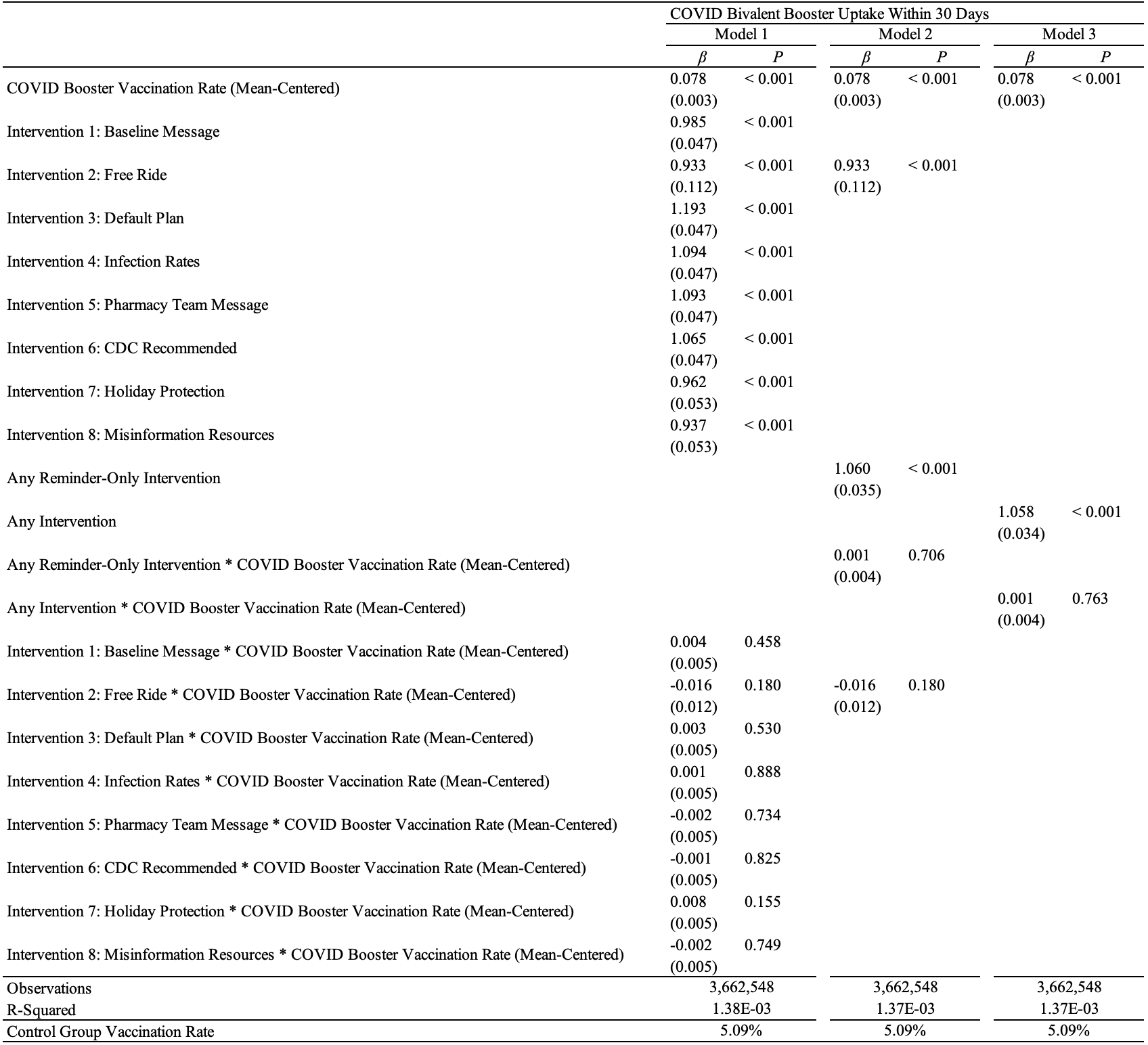
**

*Note:* This table reports the results of three ordinary least squares (OLS) regressions to predict whether a given patient received a COVID-19 booster vaccine at a CVS Pharmacy within 30 days of a patient’s study launch day. Model 1 relies on the same specification as our main regression model (Table 2, Model 1), while Model 2 and 3 include different primary predictors. In Model 2, we include two primary predictors: an indicator for whether a patient received any reminder-only intervention and an indicator for whether a patient received our free ride intervention. In Model 3, we include a single pooled treatment indicator for whether a patient received any of our megastudy’s eight intervention conditions. Each model includes a mean-centered measure of the initial COVID booster vaccination rate in the county of a patient’s closest CVS Pharmacy according to the CDC, an indicator for whether this metric has a missing value, and interactions between these variables and each intervention indicator. All three regression models also include indicators for whether the patient received their first text message on launch day 1 or launch day 2 (an indicator for receiving a message on launch day 3 is omitted). The control variables in all models are mean-centered using the mean of the holdout control. All regression coefficients and standard errors have been multiplied by 100 to improve interpretability (and thus reflect percentage point change(s) induced in vaccination uptake). Standard errors reported in parentheses are estimated robustly using HC1. Statistical tests of whether an individual regression coefficient is zero are all two-sided.

**Table S56. Heterogeneity analyses by population density (residents per square mile) in the zip code of a patient’s closest CVS Pharmacy**. Regression-estimated impact of each of our megastudy’s eight intervention conditions on bivalent COVID-19 booster uptake at CVS Pharmacy within 30 days of a patient’s study launch day as a function of residents per square mile in the zip code of a patient’s closest CVS Pharmacy according to the U.S. Census, either breaking out all interventions individually (Model 1), pooling the reminder-only interventions (Model 2), or pooling all interventions (Model 3).


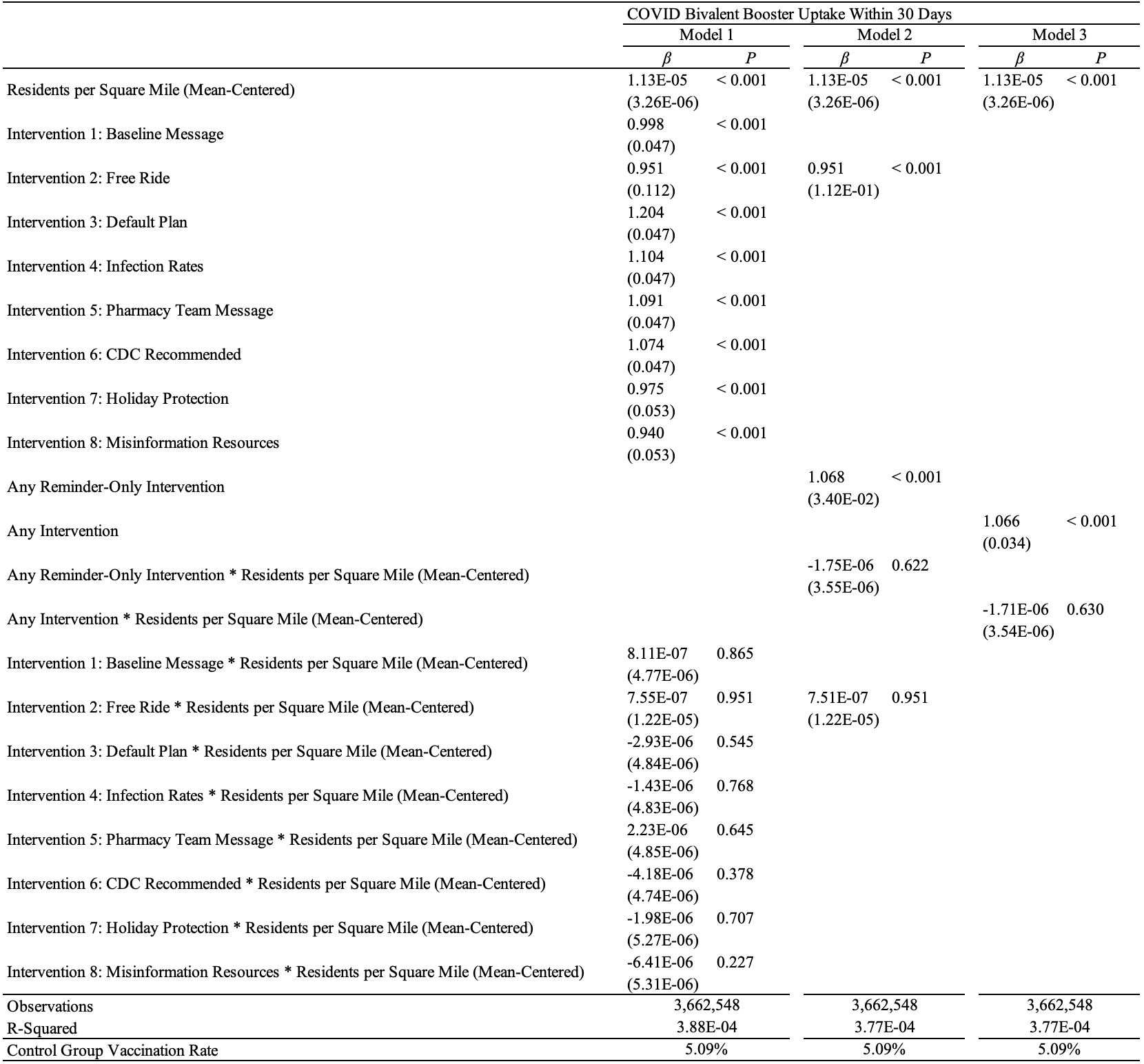


*Note:* This table reports the results of three ordinary least squares (OLS) regressions to predict whether a given patient received a COVID-19 booster vaccine at a CVS Pharmacy within 30 days of a patient’s study launch day. Model 1 relies on the same specification as our main regression model (Table 2, Model 1), while Model 2 and 3 include different primary predictors. In Model 2, we include two primary predictors: an indicator for whether a patient received any reminder-only intervention and an indicator for whether a patient received our free ride intervention. In Model 3, we include a single pooled treatment indicator for whether a patient received any of our megastudy’s eight intervention conditions. Each model includes a mean-centered measure of residents per square mile in the zip code of a patient’s closest CVS Pharmacy according to the U.S. Census, an indicator for whether this metric has a missing value, and interactions between these variables and each intervention indicator. All three regression models also include indicators for whether the patient received their first text message on launch day 1 or launch day 2 (an indicator for receiving a message on launch day 3 is omitted). The control variables in all models are mean-centered using the mean of the holdout control. All regression coefficients and standard errors have been multiplied by 100 to improve interpretability (and thus reflect percentage point change(s) induced in vaccination uptake). Standard errors reported in parentheses are estimated robustly using HC1. Statistical tests of whether an individual regression coefficient is zero are all two-sided.

**Table S57. Heterogeneity analyses by CVS Pharmacy density (CVS Pharmacies per square mile) in the zip code of a patient’s closest CVS Pharmacy**. Regression-estimated impact of each of our megastudy’s eight intervention conditions on bivalent COVID-19 booster uptake at CVS Pharmacy within 30 days of a patient’s study launch day as a function of CVS Pharmacies per square mile in the zip code of a patient’s closest CVS Pharmacy according to the U.S. Census, either breaking out all interventions individually (Model 1), pooling the reminder-only interventions (Model 2), or pooling all interventions (Model 3).

**
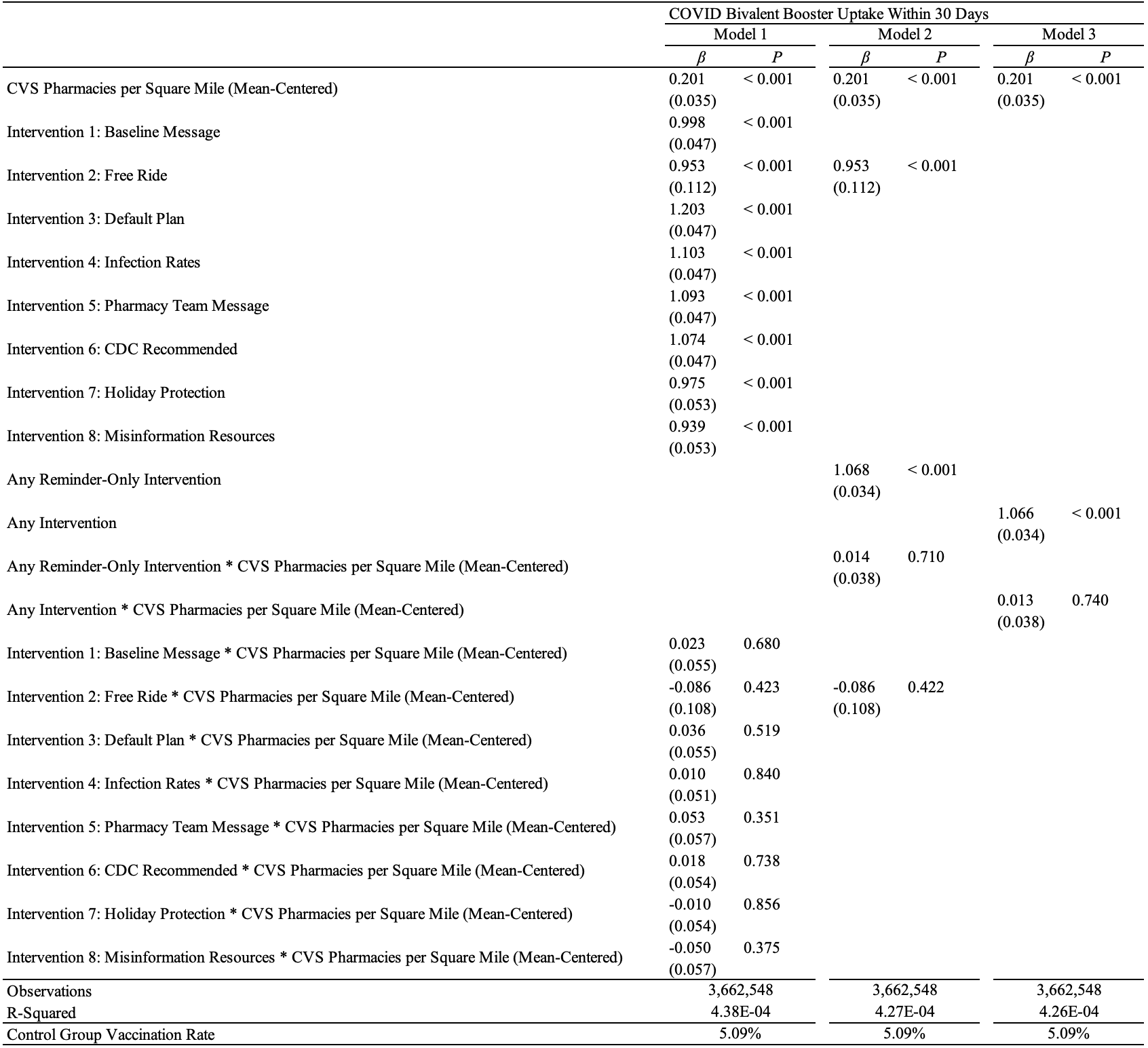
**

*Note:* This table reports the results of three ordinary least squares (OLS) regressions to predict whether a given patient received a COVID-19 booster vaccine at a CVS Pharmacy within 30 days of a patient’s study launch day. Model 1 relies on the same specification as our main regression model (Table 2, Model 1), while Model 2 and 3 include different primary predictors. In Model 2, we include two primary predictors: an indicator for whether a patient received any reminder-only intervention and an indicator for whether a patient received our free ride intervention. In Model 3, we include a single pooled treatment indicator for whether a patient received any of our megastudy’s eight intervention conditions. Each model includes a mean-centered measure of CVS Pharmacies per square mile in the zip code of a patient’s closest CVS Pharmacy according to the U.S. Census, an indicator for whether this metric has a missing value, and interactions between these variables and each intervention indicator. All three regression models also include indicators for whether the patient received their first text message on launch day 1 or launch day 2 (an indicator for receiving a message on launch day 3 is omitted). The control variables in all models are mean-centered using the mean of the holdout control. All regression coefficients and standard errors have been multiplied by 100 to improve interpretability (and thus reflect percentage point change(s) induced in vaccination uptake). Standard errors reported in parentheses are estimated robustly using HC1. Statistical tests of whether an individual regression coefficient is zero are all two-sided.

**Table S58.** Means (and standard deviations) by study condition of patients’ reported total previous COVID-19 boosters and total previous COVID-19 vaccinations across the three data pulls received from CVS Pharmacy (October 2022, December 2022, February 2023).


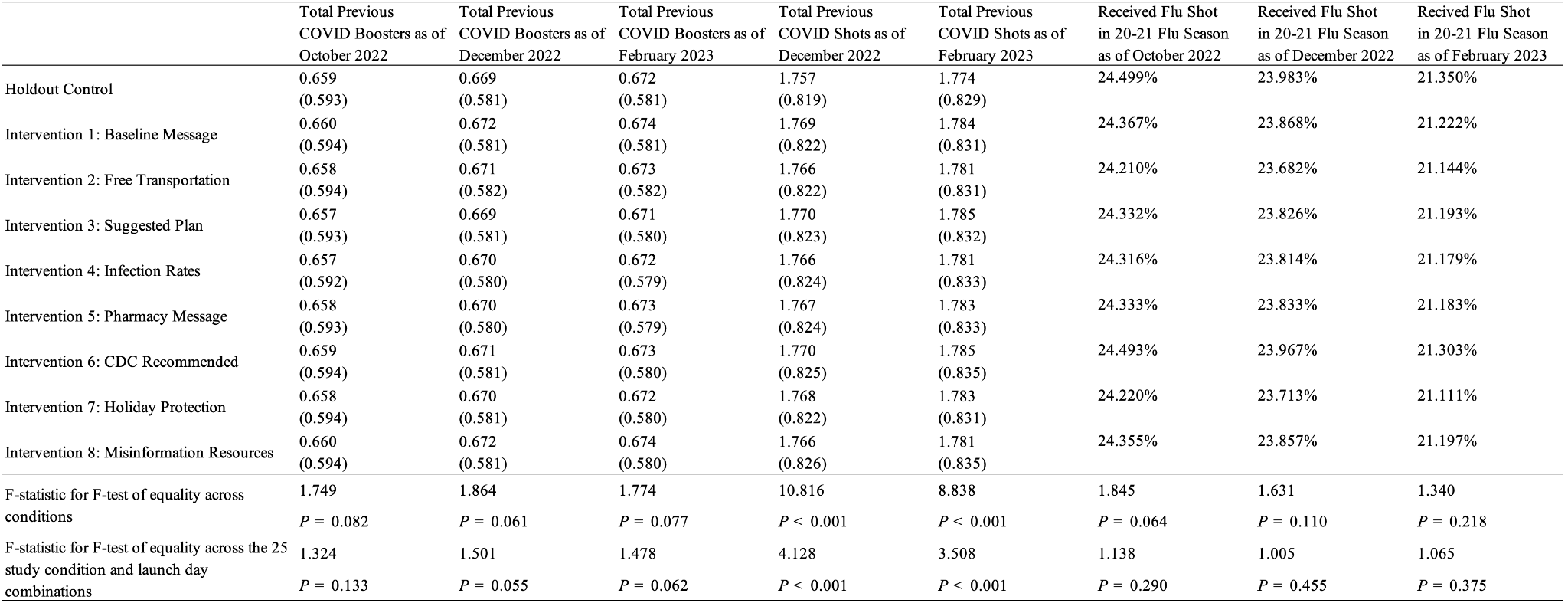


*Note:* Standard deviations are reported in parenthesis. The total previous COVID-19 shots covariate was not included in the October 2022 data provided to us. Statistical tests involving multiple regression coefficients are all undirected.

**Table S59.** Sample size, total clicks on the CVS Pharmacy vaccine scheduler link sent in reminder texts, and CVS Pharmacy vaccine scheduler links clicked per participant by intervention condition within 30 days of launch.

**
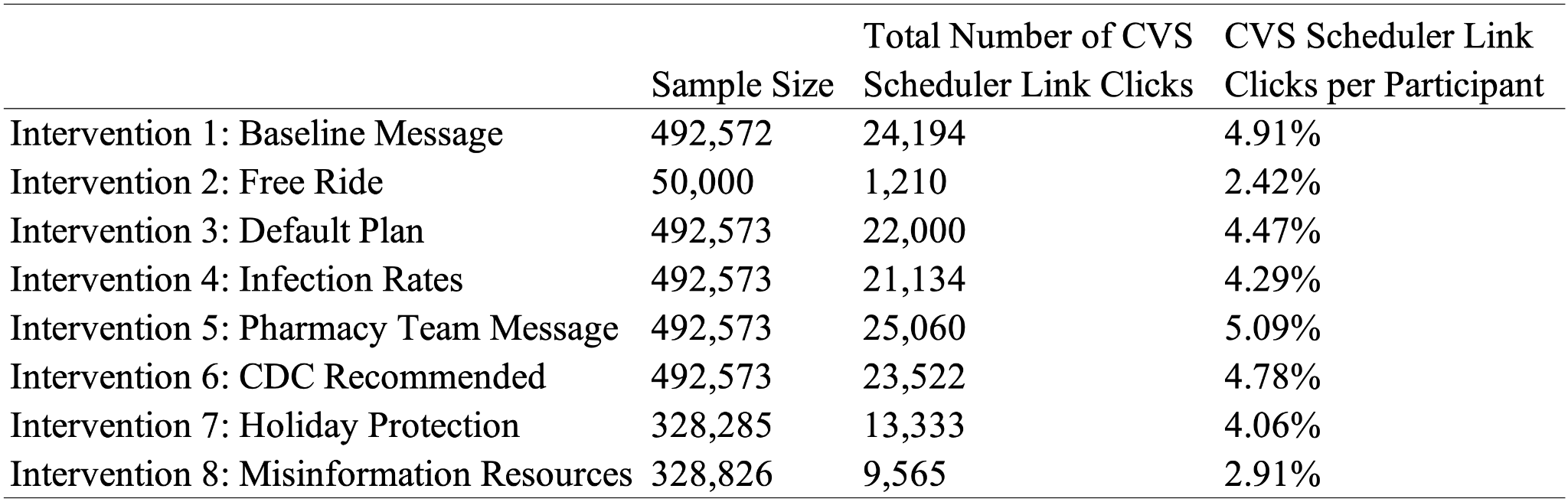
**

**Table S60.** The estimated non-surge price for the median ride, the 75th percentile ride, the 90th percentile ride, and the 99th percentile ride in each of the Metropolitan Statistical Areas included in our study based on pricing as of November 2023.


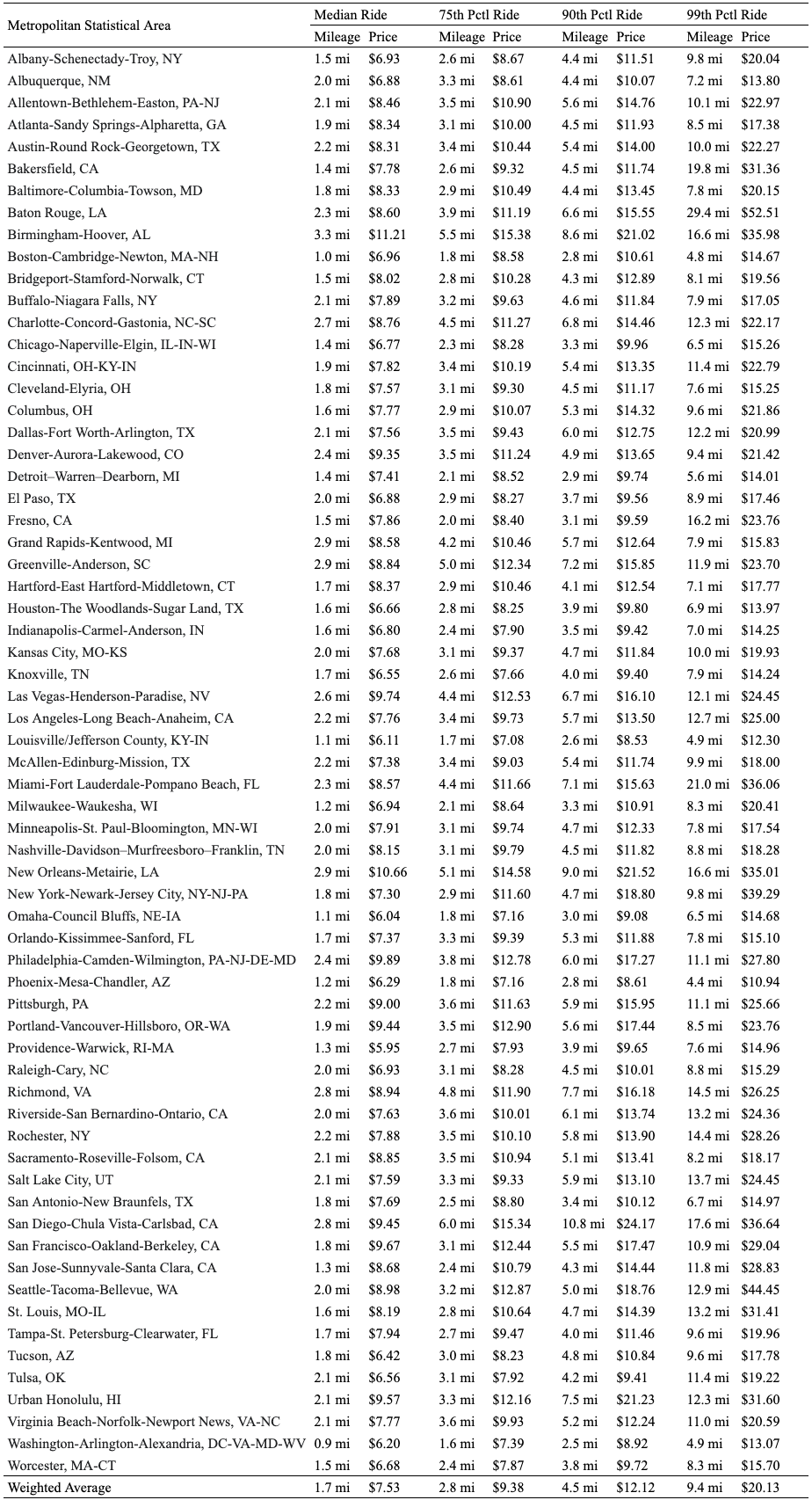


*Note.* This table reports the median, 75th percentile, 90th percentile, and 99th percentile distance and cost for rides from randomly selected addresses in each Metropolitan Statistical Area (MSA), and the weighted average of these quantities across all MSAs in our study weighted by the number of CVS Pharmacy patients in each MSA. All Lyft costs were calculated using the following Lyft equation: Base Fare + Service Fee + Cost Per Mile * estimated mileage + Cost Per Minute * estimated minutes (Lyft, 2023). Estimated mileage was calculated to one decimal point of precision using the average distance to a CVS Pharmacy for a patient living at a randomly selected address in a given patient’s zip code (note that a patient’s zip code is determined by the zip code of their most frequently visited CVS Pharmacy). Estimated minutes were calculated by pairing estimated mileage with an assumption of 20 miles per hour. Base Fare, Service Fee, Cost Per Minute, and Cost Per Mile are published by Lyft for each metropolitan area (Lyft, 2023). Each reported median ride cost was subject to the Minimum Ride Fare for its corresponding metropolitan area.

**Table S61.** Logistic regression-estimated impact of each of our megastudy’s eight intervention conditions on bivalent COVID-19 booster uptake at a CVS Pharmacy within 30 days of a patient’s study launch day (Models 1–2), bivalent COVID-19 booster uptake at a CVS Pharmacy within 90 days of a patient’s study launch day (Models 3–4), and flu shot uptake at a CVS Pharmacy within 30 days of a patient’s study launch day (Models 5–6).

**
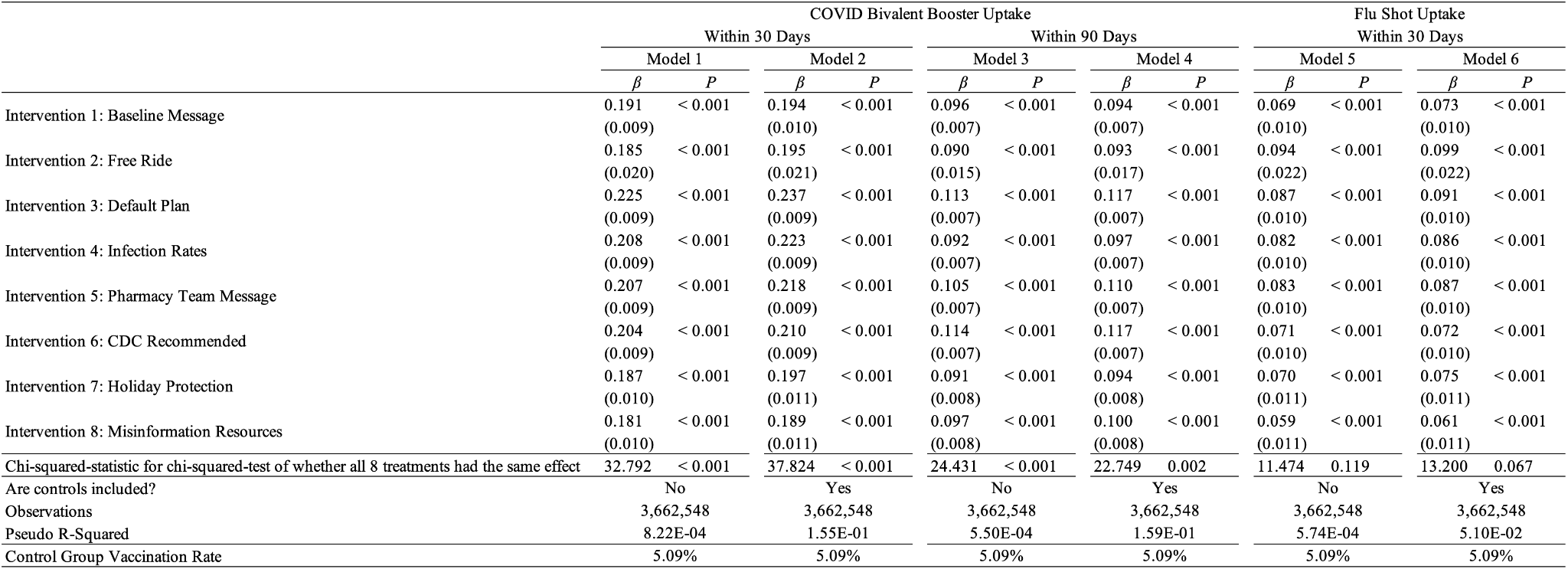
**

*Note:* This table reports the results of six logistic regressions to predict whether a given patient received a given vaccine at a CVS Pharmacy. The primary predictor variables in these regressions are eight indicators for assignment to each of our megastudy’s eight intervention conditions (the holdout control condition is the comparison group). All regression models also include indicators for whether the patient received their first text message on launch day 1 or launch day 2 (an indicator for receiving a first message on launch day 3 is omitted). In Models 2, 4 and 6, additional controls are also included for the patient’s age as of October 2022; an indicator for whether the patient’s age was greater than or equal to 50 in October 2022; an indicator for whether a patient is male; and indicators for the patient’s insurance status (Medicare, Medicaid, or unknown; commercial insurance is omitted) as of December 2022. Models 2 and 4 also control for the patient’s number of previous COVID-19 boosters prior to the start of the study according to CVS Pharmacy’s records as of October 2022 and their number of previous COVID-19 vaccinations prior to the start of the study according to CVS Pharmacy’s records as of December 2022 (which were potentially affected by our interventions, as reported in Section 3). Model 6 includes an indicator for whether a patient received a flu shot at any CVS Pharmacy during the 2021-2022 flu season. The control variables in all models are mean-centered using the mean of the holdout control. Standard errors reported in parentheses are estimated robustly using HC1 and p-values are adjusted for multiple comparisons using the Benjamini-Hochberg procedure. Statistical tests of whether an individual regression coefficient is zero are all two-sided. Statistical tests involving multiple regression coefficients are all undirected.

**Table S62.** Regression-estimated impact of each of our megastudy’s eight intervention conditions on bivalent COVID-19 booster uptake at a CVS Pharmacy within 30 days of a patient’s study launch day (Model 1) and bivalent COVID-19 booster uptake at a CVS Pharmacy within 90 days of a patient’s study launch day (Model 2) including pre-registered patient covariate controls except those potentially affected by our interventions (i.e., information on prior vaccinations).


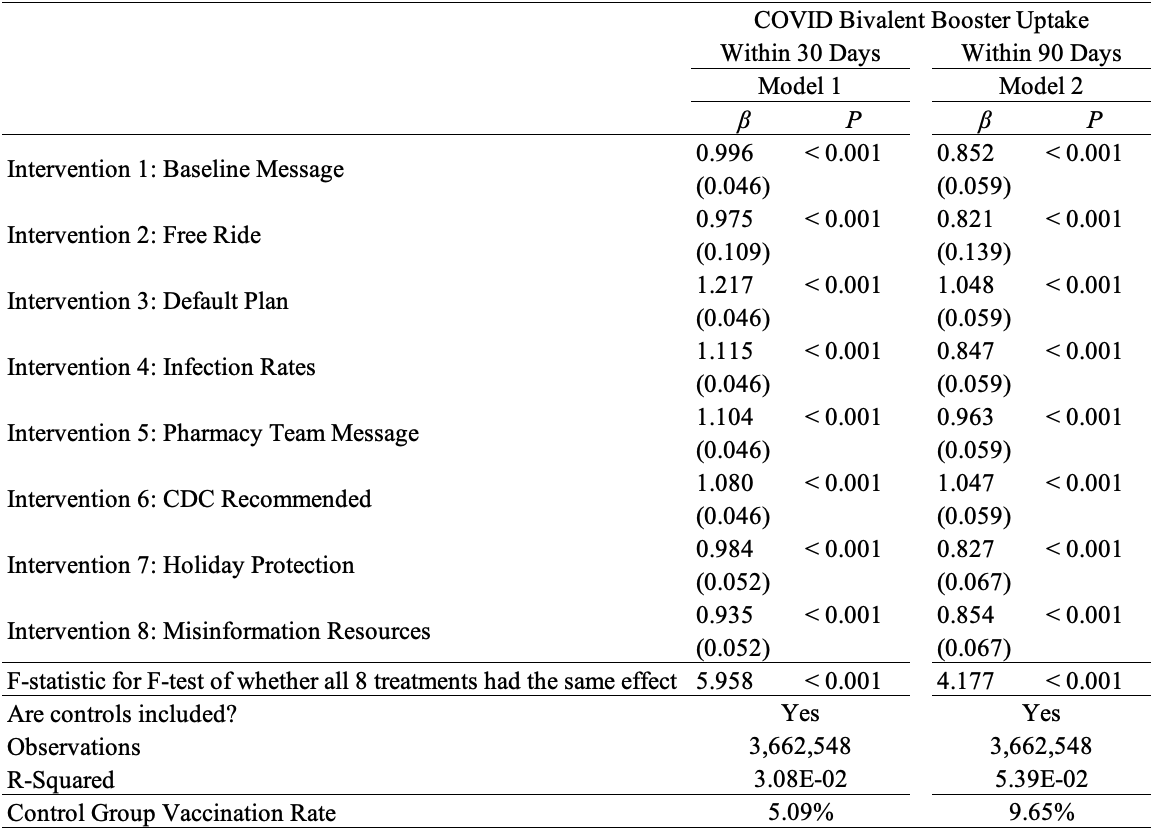


*Note.* This table reports the results of two ordinary least squares (OLS) regressions to predict whether a given patient received a given vaccine at a CVS Pharmacy. The primary predictor variables in these regressions are eight indicators for assignment to each of our megastudy’s eight intervention conditions (the holdout control condition is the comparison group). All regression models also include indicators for whether the patient received their first text message on launch day 1 or launch day 2 (an indicator for receiving a first message on launch day 3 is omitted); the patient’s age as of October 2022; an indicator for whether the patient’s age was greater than or equal to 50 in October 2022; an indicator for whether a patient is male; the patient’s number of previous COVID-19 boosters prior to the start of the study according to CVS Pharmacy’s records as of October 2022; and indicators for the patient’s insurance status (Medicare, Medicaid, or unknown; commercial insurance is omitted) as of December 2022. Control variables are mean-centered using the mean of the holdout control. All regression coefficients and standard errors have been multiplied by 100 to improve interpretability (and thus reflect percentage point change(s) induced in vaccination uptake). Standard errors reported in parentheses are estimated robustly using HC1 and p-values are adjusted for multiple comparisons using the Benjamini-Hochberg procedure. Statistical tests of whether an individual regression coefficient is zero are all two-sided. Statistical tests involving multiple regression coefficients are all undirected.

# **15. Supplementary Materials References**

1. U.S. Census Bureau. About. *United States Census Bureau* https://www.census.gov/programs-surveys/metro-micro/about.html (2021).

2. Office of Management and Budget. *OMB BULLETIN NO. 20-01*. https://www.whitehouse.gov/wp-content/uploads/2020/03/Bulletin-20-01.pdf? (2020).

3. Lyft. Area Ride Costs. lyft.com/pricing/[ALB; ABQ; ABE; ATL; AUS; BFL; BWI; BTR; BHM; BOS; BDR; BUF; CLT; CHI; CVG; CLE; CMH; DFW; DEN; DTW; ELP; FAT; GRR; GSP; BDL; IAH; IND; MCI; TYS; LAS; LAX; SDF; MFE; MIA; MKE; MSP; BNA; MSY; BKN; OMA; MCO; PHI; PHX; PIT; PDX; PVD; RDU; ICC; SBD; RST; SMF; SLC; SAT; SAN; SFO; SJC; SEA; STL; TPA; TUS; TUL; HNL; ORF; DCA; ORH] (2023).

4. CDC. Rates of COVID-19 Cases and Deaths by Vaccination Status. *Centers for Disease Control and Prevention* https://covid.cdc.gov/covid-data-tracker/#rates-by-vaccine-status (2023).

5. CDC. Estimated COVID-19 Burden. *Centers for Disease Control and Prevention* https://stacks.cdc.gov/view/cdc/117147 (2022).

6. Link-Gelles, R. *et al.* Effectiveness of Bivalent mRNA Vaccines in Preventing Symptomatic SARS-CoV-2 Infection — Increasing Community Access to Testing Program, United States, September–November 2022. *Morb. Mortal. Wkly. Rep.* **71**, 1526–1530 (2022).

7. Mogstad, M., Romano, J. P., Shaikh, A. M. & Wilhelm, D. Inference for Ranks With Applications to Mobility Across Neighbourhoods and Academic Achievement Across Countries. *Rev. Econ. Stud.* rdad006 (2023) doi:10.1093/restud/rdad006.

8. U.S. Census Bureau. American Community Survey Income in the Past 12 Months (In 2021 Inflation-Adjusted Dollars). (2021).

9. U.S. Census Bureau. American Community Survey Educational Attainment. (2021).

10. U.S. Census Bureau. American Community Survey Demographic and Housing Estimates. (2020).

11. U.S. Census Bureau. Gazetteer Files. https://www.census.gov/geographies/reference-files/time-series/geo/gazetteer-files.html (2020).

12. CDC. COVID-19 Vaccinations in the United States, County. *Centers for Disease Control and Prevention* https://data.cdc.gov/Vaccinations/COVID-19-Vaccinations-in-the-United-States-County/8xkx-amqh (2023).

13. MIT Election Data + Science Lab. Data. https://electionlab.mit.edu/data (2023).

14. *Lyft Second Quarter 2023 Earnings Call*. (2023).

1. If an address from the National Address Database was not recognizable to the Google Maps API, we manually checked it first with Google Maps, and then if necessary with Bing Maps. If in a given zip code, all the addresses from the National Address Database were unrecognizable by Google Maps’ API, Google Maps, and Bing Maps, we reverted to following the randomly generated latitude and longitude method described above for generating addresses in the zip code. [↑](#footnote-ref-1)
2. For age, residents per square mile, and CVS pharmacies per square mile, we also estimate a version of the heterogeneity analyses using the continuous version of the variables instead of the dichotomized version. [↑](#footnote-ref-2)
3. Patient name, Lyft link and scheduler link are fictitious and only provided for illustrative purposes. [↑](#footnote-ref-3)
4. Patient name, pharmacy address, and scheduler link are fictitious and only provided for illustrative purposes. [↑](#footnote-ref-4)
5. Patient name, county and scheduler link are fictitious and only provided for illustrative purposes. [↑](#footnote-ref-5)
6. Patient name, county and scheduler link are fictitious and only provided for illustrative purposes. [↑](#footnote-ref-6)
7. Patient name, pharmacy address, and scheduler link are fictitious and only provided for illustrative purposes. [↑](#footnote-ref-7)
8. Patient name and scheduler link are fictitious and only provided for illustrative purposes. [↑](#footnote-ref-8)
9. Patient name and scheduler link are fictitious and only provided for illustrative purposes. [↑](#footnote-ref-9)
10. Patient name, pharmacy phone number, and scheduler link are fictitious and only provided for illustrative purposes. [↑](#footnote-ref-10)
