# Supplementary material for: Megastudy shows that reminders boost vaccination but adding free rides does not
Source: Nature. 2024 Jun 26;631(8019):179–88. doi: 10.1038/s41586-024-07591-x (PMC11222156; doi:10.1038/s41586-024-07591-x)
Supplement: Supplementary file 2 — Reporting Summary [file 41586_2024_7591_MOESM2_ESM.pdf]

nature portfolio

Corresponding author(s): Katherine L. Milkman

Last updated by author(s): 03/06/2024

Reporting Summary

Nature Portfolio wishes to improve the reproducibility of the work that we publish. This form provides structure for consistency and transparency in reporting. For further information on Nature Portfolio policies, see our Editorial Policies and the Editorial Policy Checklist.

Statistics

For all statistical analyses, confirm that the following items are present in the figure legend, table legend, main text, or Methods section.

n/a	Confirmed	
☐	☒	The exact sample size (*n*) for each experimental group/condition, given as a discrete number and unit of measurement
☒	☐	A statement on whether measurements were taken from distinct samples or whether the same sample was measured repeatedly
☐	☒	The statistical test(s) used AND whether they are one- or two-sided<br>*Only common tests should be described solely by name; describe more complex techniques in the Methods section.*
☐	☒	A description of all covariates tested
☐	☒	A description of any assumptions or corrections, such as tests of normality and adjustment for multiple comparisons
☐	☒	A full description of the statistical parameters including central tendency (e.g. means) or other basic estimates (e.g. regression coefficient) AND variation (e.g. standard deviation) or associated estimates of uncertainty (e.g. confidence intervals)
☐	☒	For null hypothesis testing, the test statistic (e.g. *F*, *t*, *r*) with confidence intervals, effect sizes, degrees of freedom and *P* value noted<br>*Give P values as exact values whenever suitable.*
☐	☒	For Bayesian analysis, information on the choice of priors and Markov chain Monte Carlo settings
☒	☐	For hierarchical and complex designs, identification of the appropriate level for tests and full reporting of outcomes
☐	☒	Estimates of effect sizes (e.g. Cohen's *d*, Pearson's *r*), indicating how they were calculated

Our web collection on statistics for biologists contains articles on many of the points above.

Software and code

Policy information about availability of computer code

Data collection	The experimental megastudy data analyzed in this paper were provided by CVS Pharmacy. Publicly available supplementary data was retrieved from the CDC, U.S. Census Bureau, the Massachusetts Institute of Technology Election Data + Science Lab, and the Department of Transportation's National Address Database. Additional supplementary data was generated using Google Maps API, Google Maps, and Bing Maps.<br><br>The prediction study data was collected via Qualtrics from a layperson sample recruited via Prolific and an expert sample recruited through professional listserves and social media.<br><br>Fully anonymized and de-identified data are available on the Open Science Framework as are aggregated summary statistics (https://bit.ly/3MhRHgm). The majority of the data was cleaned using Stata 17.0, with some of the supplementary data being cleaned in Python.
Data analysis	The majority of the data analysis was performed in Stata 17.0, with supplementary analyses being performed in R and Python. All data analysis code is available on the Open Science Framework (https://bit.ly/3MhRHgm).

For manuscripts utilizing custom algorithms or software that are central to the research but not yet described in published literature, software must be made available to editors and reviewers. We strongly encourage code deposition in a community repository (e.g. GitHub). See the Nature Portfolio guidelines for submitting code & software for further information.

Data

Policy information about availability of data

All manuscripts must include a data availability statement. This statement should provide the following information, where applicable:

- Accession codes, unique identifiers, or web links for publicly available datasets
- A description of any restrictions on data availability
- For clinical datasets or third party data, please ensure that the statement adheres to our policy

Provide your data availability statement here.

Research involving human participants, their data, or biological material

Policy information about studies with human participants or human data. See also policy information about sex, gender (identity/presentation), and sexual orientation and race, ethnicity and racism.

Reporting on sex and gender	Our interventions were generally more effective for older recipients and men.
Reporting on race, ethnicity, or other socially relevant groupings	Information on patient's race and/or ethnicity was not available. However, we did conduct heterogeneity and subgroup analyses using the racial composition of the zip code of a patient's nearest CVS Pharmacy. We see that our interventions were generally more effective in neighborhoods where a lower proportion of residents were White. We do not observe substantial or systematic heterogeneity in treatment effects based on patients' neighborhoods' proportion of Black, Hispanic, or Asian residents.
Population characteristics	In our megastudy, patients had an average age of 47.30 years and were were 40.43% male.<br><br>In our prediction study, the lay sample had an average age of 35.69 years and was 52.26% male. The expert sample had an average age of 41.86 years and was 50.00% male.
Recruitment	Our megastudy was conducted with over 3.66 million patients of CVS Pharmacy. Only patients who had completed their primary COVID-19 vaccination series were eligible for a bivalent booster according to the FDA. We further restricted our sample to the subset of patients who had consented in writing to receive text communications to comply with the Federal Communication Commission (FCC)'s Telephone Consumer Protection Act, which outlaws sending communications via text without a patient's consent.<br><br>For our prediction study, we recruited 216 U.S. residents from Prolific and 215 participants who held a PhD in psychology, economics, business, or a related field in the social sciences via social media and the listserves of the Society for Judgement and Decision Making and the Economic Science Association.
Ethics oversight	This study has been approved by the Institutional Review Board of the University of Pennsylvania.

Note that full information on the approval of the study protocol must also be provided in the manuscript.

Field-specific reporting

Please select the one below that is the best fit for your research. If you are not sure, read the appropriate sections before making your selection.

☐ Life sciences ☒ Behavioural & social sciences ☐ Ecological, evolutionary & environmental sciences

For a reference copy of the document with all sections, see nature.com/documents/nr-reporting-summary-flat.pdf

Behavioural & social sciences study design

All studies must disclose on these points even when the disclosure is negative.

Study description	We conducted a megastudy with 3.66 million pharmacy patients testing the effect of free round-trip Lyft rides to pharmacies for COVID-19 booster vaccination appointments as well as seven other behaviorally-informed interventions aimed at encouraging takeup of COVID-19 boosters. We also conducted a prediction study with both laypeople and experts to see if they could accurately predict which interventions would perform best and what effect they would have on vaccination uptake.
Research sample	Our megastudy was conducted with over 3.66 million patients of CVS Pharmacy. Our prediction study recruited 216 U.S. residents from Prolific and 215 participants who held a PhD in psychology, economics, business, or a related field in the social sciences
Sampling strategy	Our megastudy was a randomized control trial conducted with a large, national pharmacy chain. Our prediction study recruited lay participants via Prolific and expert participants via professional listserves and social media.
Data collection	The experimental megastudy data analyzed in this paper were provided by CVS Pharmacy. The prediction study data was collected using Qualtrics.

Timing	For the megastudy, data collection started on the day patients received their first text message and ended 90 days after. Patients received their first text messages on 11/3/2022, 11/5/2022, or 11/8/2022.<br><br>For the prediction survey, data was collected in early 2023.
Data exclusions	In the megastudy, 985,145 patients were excluded after assignment to additional treatment conditions that failed to launch. An additional 328,034 patients from interventions 7 and 8 were excluded because of an error sending their messages on launch day 1.<br><br>In the prediction survey, 17 participants from the layperson sample and 52 participants from the expert sample were excluded for failing attention and comprehension checks.
Non-participation	There was no attrition.
Randomization	Participants in the megastudy were randomized to one of eight experimental conditions or a control group. The prediction study did not have separate conditions, thus no randomization took place.

Reporting for specific materials, systems and methods

We require information from authors about some types of materials, experimental systems and methods used in many studies. Here, indicate whether each material, system or method listed is relevant to your study. If you are not sure if a list item applies to your research, read the appropriate section before selecting a response.

Materials & experimental systems

n/a	Involved in the study
☒	☐ Antibodies
☒	☐ Eukaryotic cell lines
☒	☐ Palaeontology and archaeology
☒	☐ Animals and other organisms
☒	☐ Clinical data
☒	☐ Dual use research of concern
☒	☐ Plants

Methods

n/a	Involved in the study
☒	☐ ChIP-seq
☒	☐ Flow cytometry
☒	☐ MRI-based neuroimaging

Plants

Seed stocks	N/A
Novel plant genotypes	N/A
Authentication	N/A
